# Early diagnostic biomarkers for acute myocardial infarction unveiled by metabolomics, Mendelian randomization, and machine learning

**DOI:** 10.1186/s43556-025-00387-z

**Published:** 2026-01-13

**Authors:** Hao Fan, Xiaoya Fu, Qingqing Guo, Feifan Jia, Xiao-Yu Wei, Jun Liu, Ningxuan Zhang, Chenglin Zhu, Jiujin Shi, Lei Zhang, Ji-Cheng Li

**Affiliations:** 1 School of Basic Medical Sciences, Henan University, Kaifeng, 475004 Henan China; 2 Department of Laboratory Medicine, Dongguan Hospital of Guangzhou University of Chinese Medicine, Dongguan, China

**Keywords:** Metabolomics, Acute myocardial infarction, Diagnostic Markers, Mendelian Randomization, Machine Learning

## Abstract

Acute myocardial infarction (AMI) remains a leading cause of global cardiovascular morbidity and mortality. Limitations in current diagnostic methods hinder early detection and intervention, creating an urgent need for novel early diagnostic biomarkers. This study employed an integrated multi-omics approach, combining metabolomics, Mendelian randomization (MR), and transcriptomics data to identify potential AMI biomarkers. Plasma metabolomic profiling revealed 174 differentially abundant metabolites. Subsequent MR analysis pinpointed a key causal metabolite, L-arachidoyl carnitine (carnitine C20:0). Genes associated with this metabolite were retrieved from the GeneCards database and cross-referenced with differentially expressed genes from the GEO database, leading to the identification of 10 candidate biomarker genes: *ACSL1,* *PYGL,* *DYSF,* *MGAM,* *SLC7A7,* *SULF2,* *KCNJ2,* *CYP1B1,* *NCF2,* and *SLC22A4*. By constructing and evaluating 80 machine learning models, the Enet[alpha = 0.1] model was determined to have the optimal diagnostic performance. The diagnostic potential of these ten genes was further corroborated by logistic regression with tenfold cross-validation. Additionally, immune cell infiltration analysis using the CIBERSORT algorithm uncovered potential associations between the candidate genes and specific immune cell subpopulations. In conclusion, this sequential multi-omics investigation successfully identifies and validates 10 gene biomarkers related to AMI, offering new perspectives for early precision diagnosis and insights into the disease’s pathogenesis, alongside potential therapeutic targets.

**Supplementary Information:**

The online version contains supplementary material available at 10.1186/s43556-025-00387-z.

## Introduction

Acute myocardial infarction (AMI) is a fatal cardiovascular disease based on the pathology of coronary atherosclerosis. It is classified into ST-segment elevation myocardial infarction (STEMI) and non-ST-segment elevation myocardial infarction (NSTEMI) based on the presence or absence of ST-segment elevation at presentation [1].Epidemiological data shows that approximately two-thirds of 28-day deaths in AMI patients occur before the patient arrives at the hospital, resulting in a higher mortality rate [2]. Early diagnosis of AMI is significant to improving the success rate of life-saving treatment. Current methods of early diagnosis of AMI include patient's clinical manifestations, imaging examinations, echocardiography, and serum biomarkers. Cardiac troponin (cTn) is currently the most widely used early serum biomarker in clinical practice. According to relevant discussions in the American Journal of Cardiology, the dynamic changes in cTn levels serve as a crucial basis for determining acute myocardial injury [3]. However, alterations in cTn levels lack causative specificity; besides AMI, elevated levels may also occur in various non-ischemic disease states, such as chronic kidney disease [4]. Despite its high sensitivity, cTn testing requires approximately 12 h post-AMI onset to reach peak concentrations. This time lag constitutes a critical limitation for this marker in clinical scenarios demanding rapid diagnosis [5].Therefore exploring more robust early biomarkers has become a focus of current research.

Mendelian randomization (MR) estimates causality between exposure factors and study outcomes by using genetic variation as an instrumental variable, which can be biomarkers, anthropometric measures, and any other risk factors that may affect outcomes [6]. Metabolomics directly maps physiological and pathological states, providing highly timely and information-rich molecular evidence for mechanism elucidation and biomarker discovery [7]. Elevated levels of o-aminobenzoic acid and tryptophan, along with decreased levels of xanthine and 3-OH-kynurenine, may play significant roles in MI [8]. However, fluctuations in these metabolite levels are not AMI-specific biomarkers, as they are also observed in diseases such as avascular necrosis of the femoral head and rheumatoid arthritis [9, 10].This non-specificity limits the diagnostic value of individual metabolites. To address this challenge, this study employs an integrated analysis strategy combining MR with metabolomics techniques. MR analysis provides a more robust evidence chain than simple association studies through multiple MR methods and robustness tests (e.g., MR-PRESSO, Egger's intercept, heterogeneity tests, and leave-one-out validation) [11].

Machine learning algorithms demonstrate significant advantages over conventional risk scores in predicting and assessing cardiovascular disease risk [12]. When integrated with omics data, they provide additional insights into disease pathological pathways, enabling early preventive interventions or prognostic assessments across multiple stages of disease progression [13, 14].Building upon the aforementioned research foundation, we integrated metabolomics, MR, and machine learning to screen candidate biomarkers, thereby significantly enhancing biological interpretability and result robustness. Overall, our objective is to identify differentially expressed genes (DEGs) associated with causally implicated differential metabolites and construct machine learning models, thereby providing novel approaches for the early identification of AMI and the elucidation of its biological mechanisms.

We screened AMI-related differential metabolites based on the quantitative analysis of 1400 plasma metabolites and verified their causal associations by MR, targeted key metabolite CarnitineC20:0, and combining with mining its regulated genes in the GeneCards database, and taking the intersection with the differential genes of AMI screened with the Genomics Expression Omnibus(GEO) data, and finally obtained 10 AMI genes that are related to metabolites and are differential. Subsequently, we built a machine learning diagnostic model stemmed from the above genes, further more, combined with the immune infiltration score to realize accurate stratified diagnosis of AMI, which provides a new option for more accurate diagnosis of AMI at the gene level.

## Results

### Metabolomics analysis of differential metabolites

**Fig.1:**
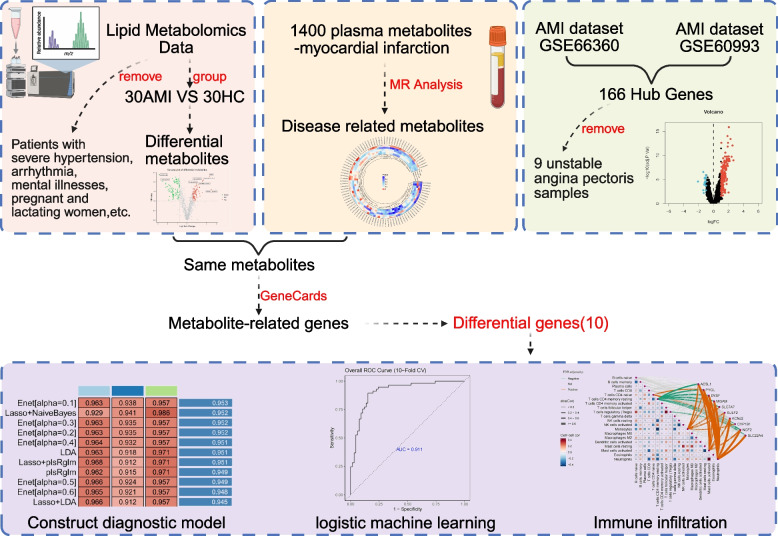
Flowchart We combined metabolomics, Mendelian randomization (MR), and Genomics Expression Omnibus(GEO) database analysis to identify 10 gene biomarkers for early Acute myocardial infarction (AMI), followed by further evaluation through machine learning and immune infiltration. Our approach comprised four main steps: 1) Screening for differentially expressed metabolites via metabolomics. 2) Identifying causally relevant core metabolites via MR Analysis. 3) Screening differentially expressed genes in the AMI context using the GEO database. 4) Constructing machine learning diagnostic models and performing immune infiltration analysis for the 10 core gene biomarkers (Created by BioRender)

**Fig. 2:**
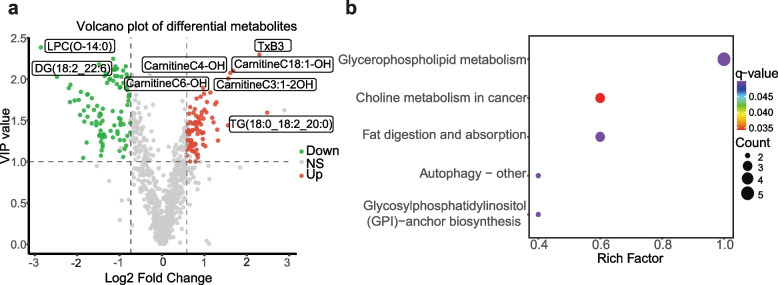
Differential metabolites in metabolomics **a** Volcano plot revealing 92 downregulated and 82 upregulated lipid metabolites in the AMI group. **b** Kyoto Encyclopedia of Genes and Genomes (KEGG) enrichment diagram showing differential lipid metabolites significantly enriched in multiple biological processes and signaling pathways, particularly those related to glycerophospholipid metabolism, choline metabolism in cancer, and fat digestion and absorption

Our workflow is illustrated in Fig. 1. To identify differential lipid metabolites, we conducted metabolomics analysis on the data. The baseline clinical characteristics of the participants are presented in Table. S1.Volcano plots revealed that 174 differential lipid metabolites were identified in the AMI and healthy control(HC) groups based on the criteria: VIP > 1.0; Benjamin–Hochberg (BH) corrected q-value < 0.05; Fold Change (FC) > 1.5 or < 0.67. A total of 174 differential lipid metabolites were identified (Table. S2). Among these, 92 lipid metabolites were downregulated in the AMI group, while 82 were upregulated (Fig. S1). A heatmap visually displays the measurement differences of the 174 differentially expressed metabolites between the AMI and HC groups through color variations (Fig. 2a). A correlation heatmap visually illustrates the correlations among the 174 differentially expressed lipid metabolites in the AMI and HC groups through color variations (Fig. S2). The Kyoto Encyclopedia of Genes and Genomes (KEGG) enrichment plot revealed that the differentially expressed lipid metabolites were significantly enriched in multiple biological processes and signaling pathways, particularly those related to glycerophospholipid metabolism, choline metabolism in cancer, and fat digestion and absorption (Fig. 2b). Overall, we identified 174 differentially expressed metabolites and conducted preliminary biological functional exploration.

### MR probes differential metabolites with causal relationships

**Fig. 3:**
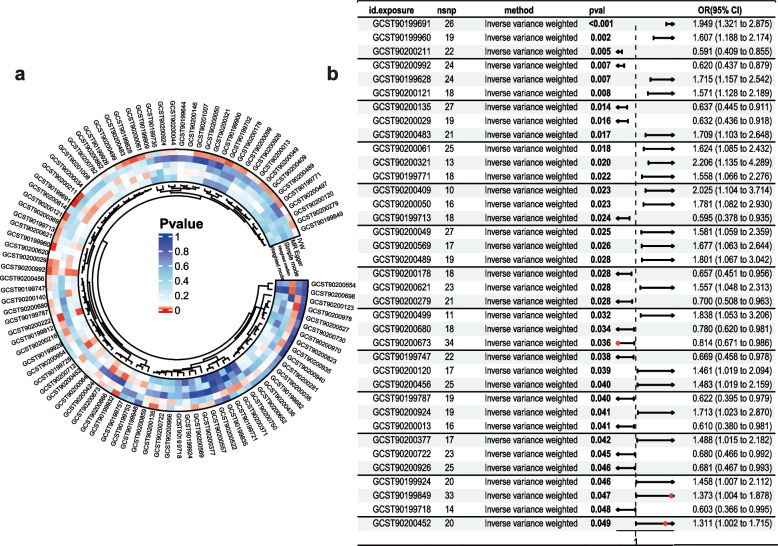
MR screening for potential metabolic markers of AMI **a** Circle plot displaying 37 plasma metabolites identified through five methods: MR-Egger, Weighted median, Simple mode, Weighted mode, and inverse variance weighting (IVW). **b** Forest plot showing *p*-values from IVW analysis for the screened plasma metabolites

To further filter and identify causally relevant core plasma metabolites, we conducted a MR analysis using 1,400 plasma metabolites as exposures and AMI as the outcome. The circle plot displays the p-values generated by five methods—MR-Egger, Weighted median, Simple mode, Weighted mode, and inverse variance weighting (IVW)—for the 1,400 plasma metabolites (Fig. 3a). Using the IVW method with P < 0.05 and consistent OR directions across all five methods, while excluding cases of multivariat non-independence and heterogeneity, we ultimately identified 37 plasma metabolites (Fig. 3b) (Table. S3).

**Fig. 4:**
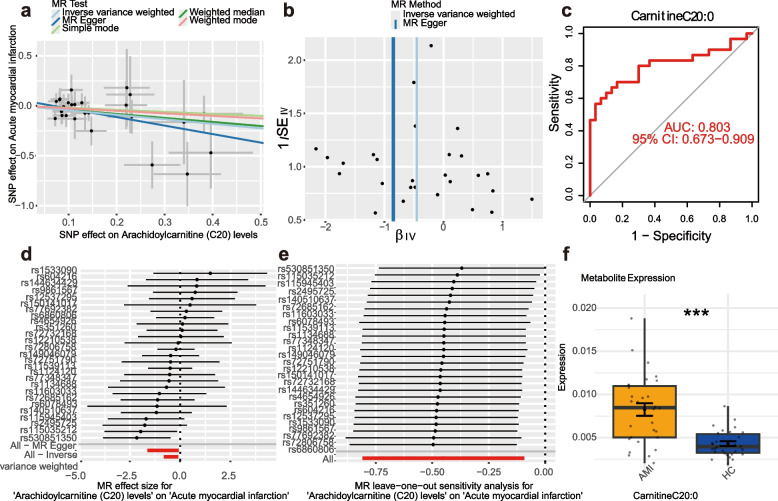
Results of Mendelian analyzes (**a**) Mendelian scatter plot of CarnitineC20:0 levels versus risk of AMI (**b**) Mendelian funnel plot of CarnitineC20:0 levels versus risk of AMI (**c**) receiver operating characteristic (ROC) curves for the prediction of the risk of AMI by CarnitineC20:0 showing statistical significance and reliability of model performance (**d**) Mendelian forest plot of CarnitineC20:0 versus AMI (**e**) CarnitineC20:0 versus AMI Mendelian outcome leave-one-out plots (**f**) Significant difference in expression levels of CarnitineC20:0 in AMI group versus control group ***, *p*<0.001

Through the analysis of metabolomics and MR, we found that CarnitineC20:0 (Arachidic acid carnitine) was a potential metabolic marker for AMI screened by the two methods together.In order to assess whether CarnitineC20:0 can be used as a diagnostic basis for AMI, In the current investigation, we used MR to assess the causal relationship between CarnitineC20:0 levels and AMI. By analyzing the effect of single nucleotide polymorphisms (SNPs) associated with CarnitineC20:0 levels on the risk of AMI, we could infer whether CarnitineC20:0 might have a causal effect on the risk of AMI(Fig. 4a, b, d, e). Additionally, we evaluated the diagnostic efficacy of CarnitineC20:0 using receiver operating characteristic (ROC) curves and box plots. Results showed that the AUC of the ROC curve for predicting AMI risk with CarnitineC20:0 was 0.803, (95% CI: 0.673–0.909) (Fig. 4c). Box plots demonstrated significant differential expression of CarnitineC20:0 between the disease and normal groups (Fig. 4f). Collectively, our analysis identified CarnitineC20:0 as a causally implicated differential metabolite capable of distinguishing between the AMI group and the HC group.

### GEO Screening of differentially expressed genes

**Fig. 5:**
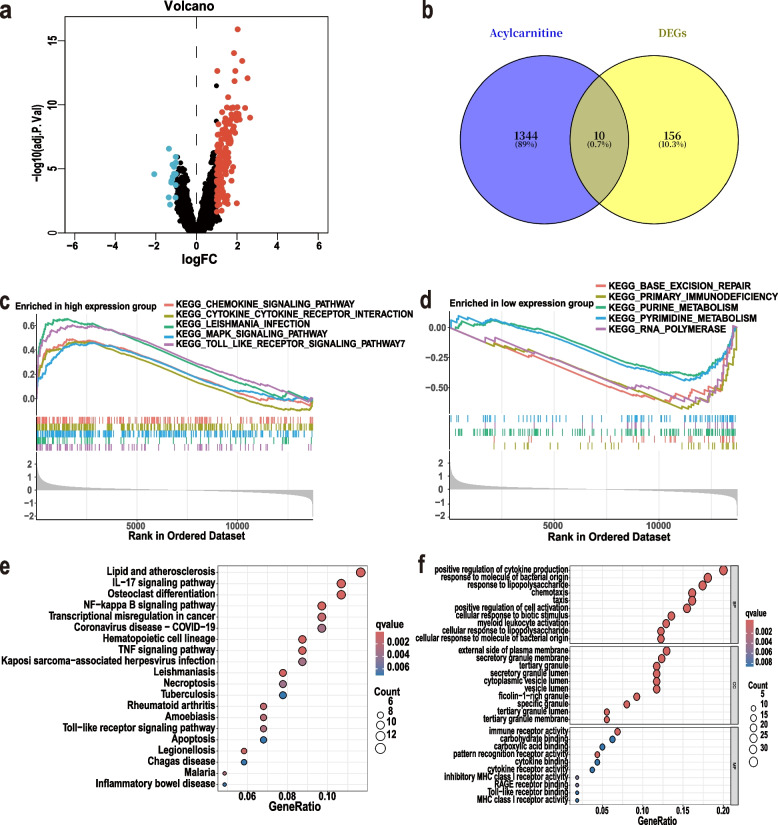
Gene expression patterns associated with AMI. **a** Volcano plot showing 150 highly expressed and 16 lowly expressed genes (**b**) Venn diagram revealing 10 differentially expressed genes from the intersection of 1,372 acylcarnitine-related candidate genes and 166 differentially expressed genes: *ACSL1, PYGL, DYSF, MGAM, SLC7A7, SULF2, KCNJ2, CYP1B1, NCF2,* and *SLC22A4* (**c**) Gene Set Enrichment Analysis (GSEA) analysis reveals significantly activated pathways in the AMI group (**d**) Intergroup GSEA analysis shows significantly activated pathways in the control group (**e**) Gene Ontology (GO) analysis bubble chart displays biological processes, cellular components, and functional analysis of differentially expressed genes (**f**) Top 10 pathways in the KEGG analysis bubble chart

To identify differentially expressed genes, we merged samples from both datasets. To reduce dimensionality and visualize sample distribution in high-dimensional space, we performed principal component analysis (PCA) on the raw data. The PCA plot revealed relatively uniform mixing of sample points across datasets, no longer segregated by origin (Fig. S3). Subsequently, 166 differentially expressed genes were identified using the “limma” method, with the screening criteria set as: adjusted P < 0.05 and |log_2_FC|> 1. Among these, 150 were highly expressed and 16 were lowly expressed (Fig. S4,5a) (Table. S4). After intersecting with genes associated with the key metabolite CarnitineC20:0 (and its acylcarnitine family), 10 potential gene biomarkers were identified (*ACSL1, PYGL, DYSF, MGAM, SLC7A7, SULF2, KCNJ2, CYP1B1, NCF2, SLC22A4*) (Table. S6) (Fig. 5b).

We performed Gene Set Enrichment Analysis (GSEA), Gene Ontology (GO) analysis, and KEGG enrichment analysis on 166 potential biomarkers to preliminarily explore their functions (Table. S5). GSEA revealed significant activation of innate immune response and inflammation-related signaling pathways in the AMI group (Fig. 5c), such as Toll-like receptor pathways and LEISHMANIA-INFECTION, while the HC group showed significant activation in DNA maintenance, replication, transcription, cellular metabolism, and immune function (Fig. 5d). GO analysis revealed that differentially expressed genes were significantly enriched in biological processes such as positive regulation of cytokine production. Significantly enriched cellular components included the outer membrane and secretory granules enzyme, while enriched molecular functions included immune receptor activity (Fig. 5e). KEGG analysis indicated that differentially expressed genes in AMI patients were primarily enriched in the IL-17 signaling pathway and NF-?B signaling pathway (Fig. 5f). In summary, we identified 166 differentially expressed genes and conducted preliminary biological functional exploration.

### Machine learning diagnostic model construction

**Fig. 6:**
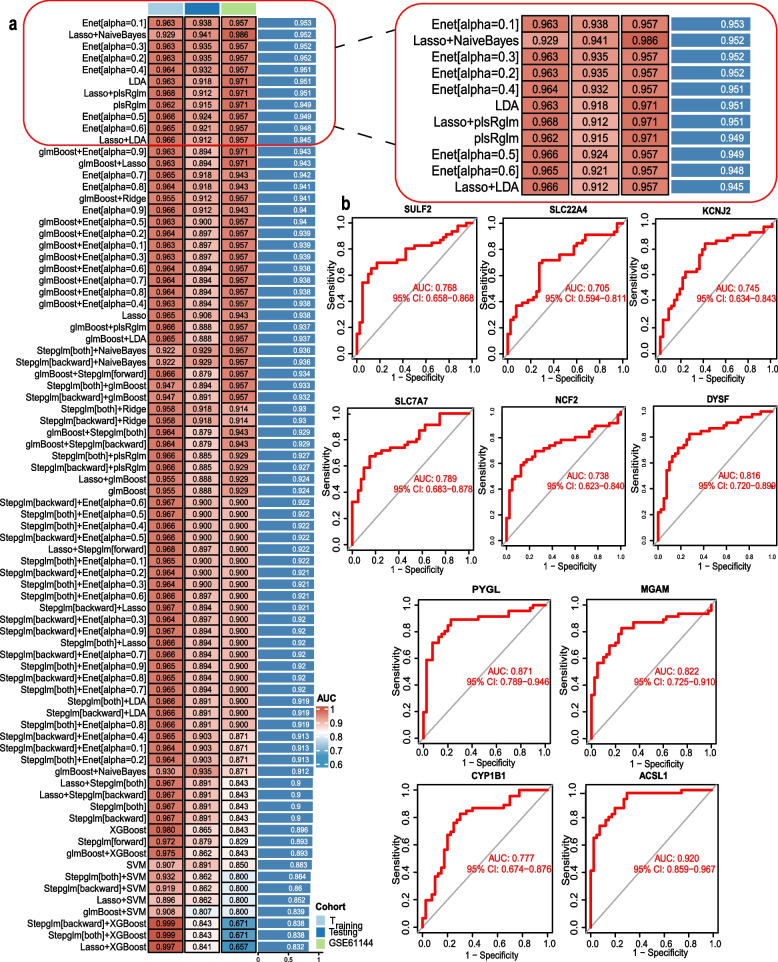
Construction and Validation Results of Machine Learning-Based Diagnostic Models (**a**) AUC values of 106 machine learning algorithm combinations randomly paired from the training and validation sets using 11 machine learning algorithms. Enet[alpha = 0.1] was selected as the final diagnostic model, achieving an AUC of 0.963 on the training set. In the validation set, the AUC value was 0.938; in the independent validation set, the AUC value was 0.957. (**b**) ROC curves were plotted and AUC values calculated for the training set of 10 model genes

To construct an efficient AMI diagnostic model, we employed multiple machine learning algorithms and their random combinations. Using merged data from GSE60993 and GSE66360, we excluded unstable angina(UA) patients (9 cases) and applied random seeding for dataset splitting: 70% as the training set and 30% as the validation set. GSE61144 served as an independent validation set to assess model generalization. The AUC values across all cohorts were averaged and ranked, with the average AUC serving as a composite performance metric to evaluate model stability and generalization capability. Ultimately, the model with the highest average AUC (the Elastic Net model with a = 0.1) was identified as the optimal model. On the training set, this model achieved an AUC of 0.963; on the validation set, the AUC was 0.938. On the independent validation set, the AUC reached 0.957 (Fig. 6a). The ROC curves of 10 genes demonstrate good diagnostic value (Fig. 6b and Fig. S5). Overall, we constructed the Enet[alpha = 0.1] diagnostic machine learning model. The 10 genes demonstrated strong potential for distinguishing between normal and MI conditions(Fig. 6b).

### Validation of the diagnostic efficacy of genes through Logistic machine learning

**Fig. 7:**
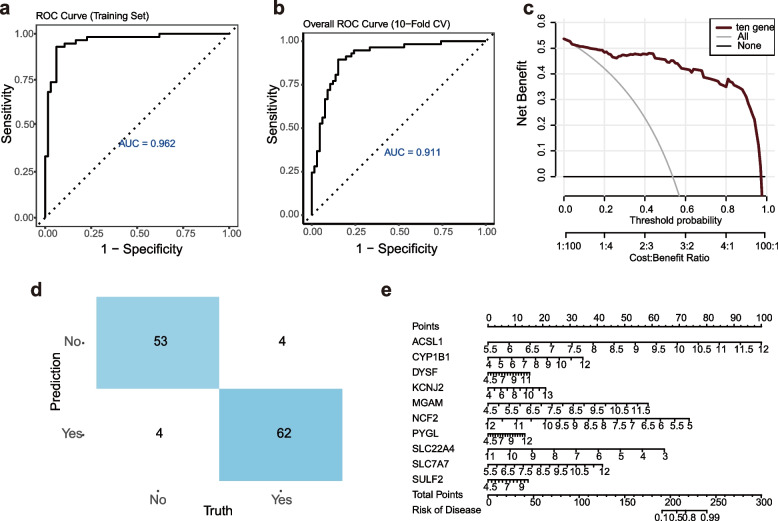
The results of Logistic machine learning (**a**, **b**) ROC curves and ten-fold cross-validation of logistic regression model, the logistic regression model has an AUC value of 0.962, and ten-fold average AUC value of 0.911 (**c**) Decision Curve Analysis (DCA), the red, gray, and black lines represent the net benefit of the 10-gene model, the scenario where all samples receive treatment, and the scenario where no samples receive treatment, respectively. (**d**) Confusion Matrix results, the quantity of samples correctly forecasted as “no” in the upper left blue area is 53, the quantitity of samples incorrectly forecasted as “yes” in the upper right white area is 4, and the total of samples incorrectly predicted to be “no” in the lower left white area is 4, and the count of samples correctly forecasted as “no” in the blue area in the lower right corner is 62, which indicates that the diagnostic model is able to effectively differentiate between “yes” and “no” samples. (**e**) Results of column line graph are used to predict the risk of AMI, on the “Risk of Disease” scale, the risk value corresponding to the 240 risk score is about 0.99, which means that the probability of the sample suffering from AMI is 99%

To validate the diagnostic efficacy of the screened ten genes more comprehensively, we grouped the normal and AMI samples in the dataset and applied logistic regression for validation. Train the logistic regression model using the combined dateset AUC value of 0.962. and the diagnostic model was further evaluated using ten-fold cross-validation, and its ten-times average AUC value was 0.911, which demonstrated that it had a high degree of accuracy and reliability in practical applications (Fig. 7a,b). The DCA plot demonstrates that the 10-gene-based model achieves high net benefit across most threshold ranges under varying Cost–Benefit Ratios (Fig. 7c). Meanwhile, the classification effect of the model is further visualized by comparing the prediction results with the real situation, in which the number of correctly predicted AMI samples and normal samples are both high, while the number of incorrectly predicted samples is relatively low, which proves that the model shows remarkable precision in practical application again(Fig. 7d). We also constructed a predictive column-line graph of AMI based on 10 genes, which shows the distribution of each indicator (10 genes such as ACSL1) in the total number of points, reflecting its contribution to the whole to further quantify the risk of AMI (Fig. 7e).

The results of the study showed that the model not only has high statistical accuracy, but also provides an important reference for early diagnosis and intervention of AMI through the dynamic assessment of gene expression levels.

### Immune filtration analysis explores the function

**Fig. 8:**
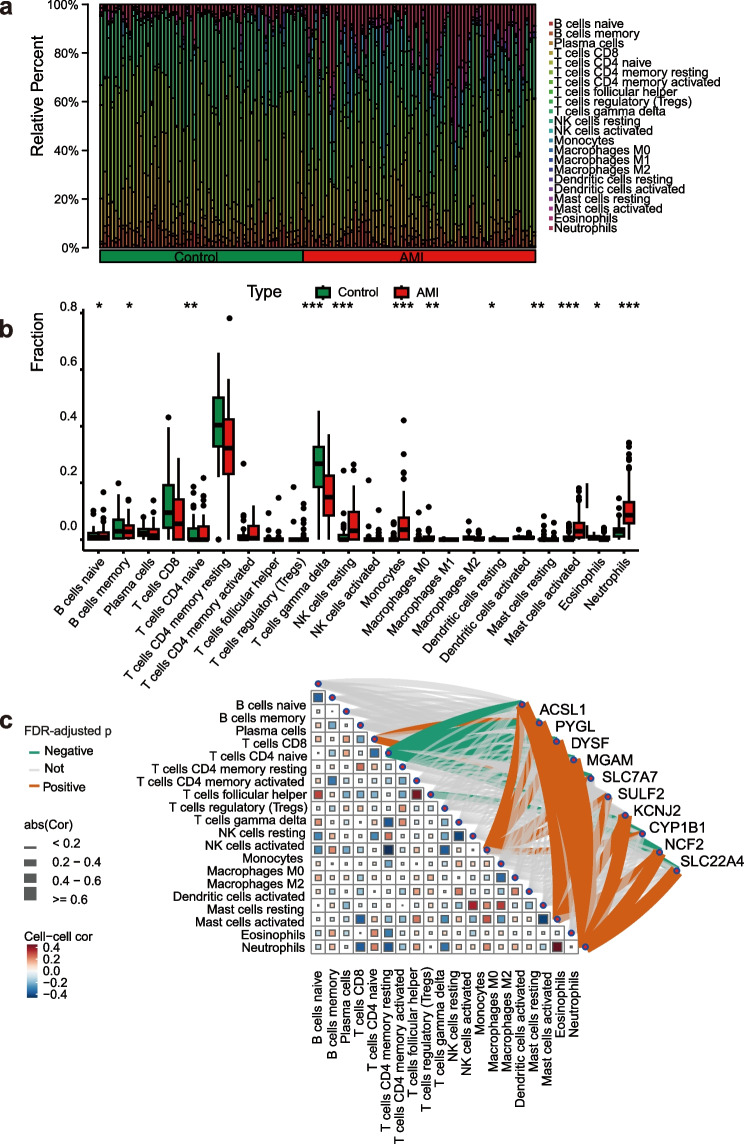
Multidimensional analysis of immune infiltration in AMI. **a**, **b** CIBERSORT analysis reveals immune infiltration between control and diseased groups with significantly increased levels of NK cells resting, monocytes, macrophages M0, mast cells activated and neutrophils, and the standards of plasma cells, CD8 + T cells, memory CD4 + T cells resting, ? d T cells, macrophages M2, and Mast cells resting were greatly decreaed in the group of AMI patients (**c**) Correlation analysis between 10 differentially expressed genes and immune microenvironment cells, red squares show positive association, blue squares show negative correlation, color intensity indicates degree of correlation, green lines indicate negative correlation, orange indicates positive correlation, and line thickness indicates degree of correlation. Both eutrophils and monocytes were positively correlated with multiple genes, and memory CD4 + T cells resting were negatively correlated with most genes *, *p*<0.05; **, *p*<0.01; ***, *p*<0.001.

To depict the potential association between immune changes and core differentially expressed genes in the context of AMI, we conducted a comprehensive analysis of immune cell distribution in both the AMI group and normal control group using CIBERSORT on the samples. The results were visualized as percentage-based bar plots (Fig. 8a) (Table. S7) The results showed a significant increase in NK cells resting, monocytes and mast cells activated, and a significant decrease in plasma cells, CD8 + T cells, memory CD4 + T cells resting and ?d T cells in the group of AMI patients (Fig. 8b). Furthermore, strong positive correlations were observed between activated mast cells and neutrophils, as well as between follicular helper T cells and regulatory T cells in AMI. In contrast, the proportion of resting memory CD4 + T cells showed a negative correlation with the ratios of resting NK cells and monocytes (Fig. S6). The gene-immune cell correlation network map (Fig. 8c), which further demonstrated that both neutrophils and monocytes had significant something to do with multiple genes, indicating that these genes could be involved in neutrophil-mediated inflammatory responses as well as the recruitment, activation, or functional regulation of monocytes. Memory CD4 + T cells resting were greatly negatively correlated with most of the genes, and it is possible that their expression is mutually regulated with the resting state of memory T cells.

These immune cells associated with the identified genes may represent potential immunotherapeutic targets for adjunct treatment of AMI, offering new directions and insights for future immune-based therapeutic strategies.

## Discussion

AMI is one of the cardiovascular diseases characterized by coronary artery obstruction, and current evidence proves that timely reconstruction of obstructed coronary arteries significantly reduces the mortality of AMI [15]. Early on, we used electrocardiogram and biochemical markers of MI for the diagnosis of AMI, but many patients do not exhibit these features at the time of presentation [16]. Genetic testing have the advantage of high specificity and stability as an early diagnosis of AMI. It is important for early diagnosis of MI by finding novel genetic biomarkers [17]. Using a combination of metabolomics, MR and machine learning, we constructed a diagnostic model of AMI and explored its immune profile. Our findings demonstrate that the ten genes, including *ACSL1, PYGL*, and *DYSF*, show potential for the early diagnosis of AMI.

Metabolomics approaches offer great potential and promising tools for the study of the pathogenesis of many diseases, demonstrating significant potential [18]. Plasma metabolites such as 11-cis-retinal, glycerophospholipids, triacylglycerols, acylcarnitines, fatty acids, valine, 3-hydroxybutyrate, anthranilic acid, tryptophan, and aspartate have shown significant alterations in AMI patients, indicating their utility as diagnostic and prognostic biomarkers for cardiac conditions [8, 19–21]. MR, a powerful tool in epidemiology, can be used to estimate the causal effect of exposure on outcome by using genetic variation of the instrumental variable (IV) serves as the exposure [22]. By integrating the strengths of both approaches, we obtained plasma metabolites that not only have differential expression but also exhibit a causal relationship, CarnitineC20:0. CarnitineC20:0 belongs to one of the acylcarnitines. Used alone, it may not fully reflect the metabolic changes in AMI bioprocesses. Instead, the acylcarnitine class as a whole contains a number of different acylcarnitine metabolites that can more fully represent the role of this class of metabolites in the disease. In addition, the processes of synthesis, transport and catabolism of acylcarnitines metabolites involve the regulation of multiple genes. By expanding the scope, more genes related to these metabolic processes can be screened, thus revealing more comprehensively the regulatory mechanisms of genes in diseases. For the above two reasons, we downloaded acylcarnitine-related genes from GeneCards and took the intersection with the differential genes screened in the GEO database, and we got the differential genes based on differential metabolites. They are *ACSL1, PYGL, DYSF, MGAM, SLC7A7, SULF2, KCNJ2, CYP1B1, NCF2, SLC22A4*. Machine learning plays a significant role in disease prediction and prognosis [23]. We employed machine learning diagnostic models to explore those 10 genes, ultimately selecting the Enet[alpha = 0.1] algorithm. ROC curve analysis demonstrated that our 10-gene differential expression model holds significant advantages for early identification of AMI. Unlike conventional metabolomics studies that often report numerous phenotype-associated metabolites lacking causal evidence, our MR-based screening prioritized casual metabolites with genetic support, fundamentally reducing the risks of false positives and casual confounding. By incorporating genes related to these causality-validated metabolites as input features for machine learning models, we not only enhanced predictive performance but also improved model interpretability and more likely to point to intervenable therapeutic targets. Feature prescreening based on genetic causality prioritization can also effectively reduce dimensionality and minimizes overfitting. When combined with rigorous cross-validation and independent cohort testing, it ensures greater model robustness and improved generalizability across diverse populations.

Monocytes and macrophages are pleiotropic cells of the innate immune system, indispensable in both the initial inflammatory response of the heart to injury and the subsequent wound healing process [24]. To clarify the impact of genes like *ACSL1* and *PYGL* on AMI within the context of the immune system and to mechanistically link immune changes with the identified key genes, we conducted a detailed and comprehensive discussion of the 10 genes.

*ACSL1* is a core regulator of lipid metabolism, and its high expression is an independent risk factor for AMI [25]. Elevated *ACSL1* expression reduces fatty acid ß-oxidation and increases triglyceride levels [26]. Additionally, high *ACSL1* expression in monocytes activates the lysophosphatidylcholine (LPC)/lysophosphatidic acid (LPA) metabolic axis, promoting neutrophil extracellular trap (NET) formation and amplifying thrombo-inflammatory responses [25, 27]. As the rate-limiting enzyme in glycogenolysis, the direct association between *PYGL* and AMI remains unclear. However, studies have confirmed that glycogen metabolism is a key regulatory link in macrophage-mediated acute inflammatory responses. Upon activation by IFN-?/LPS stimulation, macrophages undergo *PYGL*-mediated glycogenolysis to produce glucose, which is subsequently metabolized via the pentose phosphate pathway to generate large amounts of NADPH and activate *STAT1* signaling, thereby driving acute inflammation; the specific high expression of *PYGL* in M1 macrophages within atherosclerotic plaques suggests its important role in AMI-related inflammation [28, 29]. In peripheral blood leukocytes of patients with atherosclerosis, hypermethylation of the *DYSF* promoter upregulates its expression, promoting monocyte activation; it also enhances monocyte activation by upregulating selectin L(*SELL)* [30]. Consequently,* DYSF* may facilitate the recruitment of monocytes to ischemic myocardium, exacerbating local inflammation. As an intestinal a-glucosidase, accumulating evidence indicates that gut microbiota dysbiosis is a core driver of cardiometabolic risk factors such as atherosclerosis [31], suggesting that *MGAM* may indirectly influence AMI through the "gut-heart axis". Reduced *MGAM* activity increases intestinal fermentation substrates and elevates short-chain fatty acid (SCFA) levels, playing a critical role in colitis [32, 33], while monocytes and macrophages are also regulated by SCFAs [34]. *SLC7A7* bidirectionally transports arginine and glutamine [35], precisely regulating macrophage polarization and T cell activation, thereby potentially acting as a core player in immunometabolic disorders following AMI [36]. By removing 6-O-sulfate groups from heparan sulfate (HS) in the extracellular space [37], *SULF2* can alter the binding capacity of monocyte chemoattractant protein-1 (CCL2) to HS chains. *SULF2* enhances the activity and stability of CCL2, thereby significantly increasing monocyte recruitment and indirectly participating in inflammatory responses [38, 39]. Cells overexpressing *SULF2* create a microenvironment enriched in specific growth factors (e.g., VEGF, FGF2, Wnt) [40, 41], which strongly promote macrophage polarization, inducing monocyte differentiation into M1/M2-type tumor-associated macrophages (TAMs), and Macrophages co-expressing both M1 and M2 phenotypes, all these components represent potential factors influencing the development of cardiovascular diseases [42]. The protein encoded by *KCNJ2*, inward rectifier potassium channel 2.1 (Kir2.1), is a key "homeostatic" protein. Kir2.1 regulates macrophage metabolic reprogramming and inflammatory responses by maintaining macrophage membrane potential [43]. Impaired endothelial function in hypertension is associated with loss of Kir2.1 activity in human resistance arteries, leading to reduced blood flow-induced vasodilation [44]. We hypothesize that *KCNJ2* contributes to AMI pathogenesis by affecting vascular endothelial injury. Under myocardial hypoxic stress, *CYP1B1* is upregulated in myocardial tissue, triggering oxidative stress and inflammatory responses [45]. Abnormal *CYP1B1* metabolic activity exacerbates neutrophil chemotaxis and NET formation, potentially amplifying the early inflammatory cascade in AMI [46]. Studies have shown that *CYP1B1* enzyme and its associated cardiotoxic intermediate metabolite hydroxyeicosatetraenoic acids (HETEs) exert direct effects on the development of myocardial hypertrophy [47]. *CYP1B1*-mediated metabolites may influence the polarization balance of M1 and M2 macrophages by activating signaling axes such as the aryl hydrocarbon receptor (AHR) [48, 49]. Additionally, oxidative stress promotes the formation of lipid-laden macrophages via *CYP1B1* [50]. Following AMI, *CYP1B1* may not only participate in pathological processes by exacerbating inflammatory damage in ischemic myocardium [51] but also influence myocardial repair and fibrosis by regulating immunometabolic homeostasis. As a component of NADPH oxidase, *NCF2*-mediated reactive oxygen species (ROS) bursts promote low-density lipoprotein (LDL) oxidation and vulnerable plaque formation in the atherosclerotic microenvironment [52, 53]. We hypothesize that when vulnerable plaques rupture, thrombi form on the plaque surface; complete occlusion of the coronary artery by such thrombi results in AMI. Dysfunctional *SLC22A4* (OCTN1) impairs its transport of acetylcholine and L-carnitine [54], potentially disrupting the endogenous "cholinergic anti-inflammatory pathway" and exacerbating chronic inflammation within atherosclerotic plaques [55]. Simultaneously, it promotes macrophage polarization toward a pro-inflammatory phenotype, collectively increasing plaque instability.

Using a sequential multi-omics integration approach, we screened ten metabolite-linked differential genes—*ACSL1, PYGL, DYSF, MGAM, SLC7A7, SULF2, KCNJ2, CYP1B1, NCF2* and *SLC22A4*—on the basis of plasma metabolites, Mendelian randomization and the GEO database. These genes are central to the cardiometabolic and immuno-inflammatory network implicated in the onset and progression of AMI. Emerging evidence identifies ACSL1 as an independent risk factor for AMI: by attenuating fatty-acid ß-oxidation, increasing triglyceride levels and activating the LPC/LPA axis in monocytes, it amplifies thrombo-inflammatory responses and promotes NET formation, supporting its role as a pathological driver and potential therapeutic target [25, 27]. Leveraging these ten genes, we established an Enet (a = 0.1) machine-learning diagnostic model for AMI that offers a novel option for early detection, and we further delineated their immune signature in AMI, illuminating their potential biological functions.

Although our study has achieved some results, it still faces several limitations. First, due to the limited size of the study sample, this may restrict the general applicability of the screened biomarkers. Second, although we have identified 10 diagnostic biomarkers for AMI, their specific mechanisms of action in AMI have not been fully elucidated, and more efforts are needed to achieve clinical translation.

Looking to the future, our study will focus on promoting the clinical translation of diagnostic markers for AMI. Currently, We have identified some potential biomarkers through bioinformatics analysis, and our next step will be to collaborate with clinical teams to conduct validation studies with the aim of developing more effective diagnostic tools. We also plan to expand the sample size of the study and construct a larger-scale AMI cohort through multi-center collaboration to enhance the generalizability of the findings. In terms of functional studies, we will utilize AMI cell and animal models to deeply explore the functions of metabolite-related differentially expressed genes, and reveal their mechanisms of action in the disease process through vivo and in vitro experiments.

## Materials and Methods

### Metabolomics differential analysis

This study was approved by the Ethics Committee of Yuebei People's Hospital affiliated with Shantou University Medical College (Approval No.: KY-2021–050), and all subjects provided written informed consent. Included 60 subjects (30 HC cases and 30 AMI cases). Exclusion criteria included severe hypertension, pulmonary dysfunction, arrhythmia, abnormalities of hepatic and renal function, neoplasms, psychiatric disorders, individuals in gestational or lactation periods, along with those diagnosed with communicable pathologies and congenital disorders.

Plasma samples were stored at - 80 °C; before detection, they were thawed at room temperature, vortexed, centrifuged at 4 °C, and subjected to organic solvent extraction (including internal standards) according to standard procedures, then concentrated by nitrogen blowdown, reconstituted with the mobile phase, and centrifuged again. The resulting supernatant was used for measurement. Mixed QC and internal standards were set up to monitor repeatability and batch-to-batch drift. Lipidomics was analyzed using ExionLC™ AD coupled with QTRAP® 6500 (MRM), with a Thermo Accucore™ C30 column (2.1 × 100 mm); widely targeted metabolomics employed the Agilent 1290–TripleTOF 6600 and Thermo Vanquish–Q Exactive HF-X (HILIC, ACQUITY BEH Amide column) platforms. Major mass spectrometry parameters and gradients were set according to laboratory optimization. Raw data were converted to mzXML using ProteoWizard; peaks were extracted and aligned using XCMS; metabolite identification was based on accurate mass (error < 10 ppm) and MS/MS spectral library matching. Data were normalized, log10-transformed, and Pareto scaled.

After data normalization, the data matrix is imported into R. The differential threshold is set as follows: VIP > 1.0; q-value < 0.05 after BH correction; Fold Change > 1.5 or < 0.67. BH correction is implemented using the R function p.adjust(…, method = "BH"), and volcano plots and heatmaps of differential metabolites are generated. Meanwhile, differential metabolites are mapped to the KEGG database for pathway enrichment analysis, with a significance threshold of Benjamini < 0.05. Correlation analysis uses pairwise complete observations to calculate the Pearson correlation matrix; the original *P*-values are obtained using psych::corr.test and multiple testing is corrected using FDR (Benjamini-Hochberg) method.

### GWAS data for AMI

The GWAS data for AMI were obtained from OpenGWAS (https://gwas.mrcieu.ac.uk/). The MR analysis of plasma metabolites and acute myocardial infarction used the GWAS ID ukb-e-I21_CSA, which includes 8,876 samples (8,502 controls and 374 cases). In the study design, 1,400 plasma metabolites were considered as exposure variables, and acute myocardial infarction was the endpoint event. The analysis strictly followed the three core principles of MR: (i) genetic instruments must be significantly associated with the exposure; (ii) instruments must be independent of potential confounders; (iii) genetic variants affect the outcome only through the exposure.

This study used SNPs as genetic instruments, selecting SNPs significantly associated with metabolites based on a genome-wide significance threshold (P < 5 × 10?8) to ensure the strength of the instrumental variables. Clustering and pruning were performed using genetic distance parameters (window = 10,000 kb) and linkage disequilibrium thresholds (r^2^ < 0.001) to eliminate the effects of population stratification. Further integration of phenotype database information was done to exclude SNPs directly associated with known confounders or endpoint events (P < 5 × 10?8). The exclusivity assumption of the instrumental variables was doubly verified through MR-PRESSO outlier detection and MR-Egger intercept test (significance level a = 0.05).

In the stage of causal effect assessment, the study used the IVW method as the core estimation model, supplemented by the weighted median method, MR-Egger regression, simple mode method, and weighted mode method to construct a comprehensive analysis framework. Cochran's Q test was used to assess study heterogeneity, and the leave-one-out sensitivity analysis was employed to verify the robustness of the results. To identify potential horizontal pleiotropy effects, both MR-Egger intercept test and global pleiotropy test were conducted simultaneously.

### Acquisition of metabolite-related gene data

GeneCards is a comprehensive gene database that integrates information from approximately 150 data sources, providing detailed annotations for genes, including function, disease associations, expression profiles, and pathways. It serves as an essential tool in biomedical research.We obtained the related genes of acylcarnitines using GeneCards database.

### GEO differential gene acquisition

GEO is a bioinformatics database established and maintained by the National Center for Biotechnology Information (NCBI) for storing, retrieving, and analyzing high-throughput gene expression and other forms of genomic counts. We downloaded the microarray datasets GSE66360, GSE60993 and GSE61144 associated with AMI from the NCBI for differential analysis and diagnostic performance assessment. Among them, GSE66360 dataset included 50 normal blood samples and 49 AMI blood samples for primary studies. GSE60993 dataset included 10 non-ST-elevation MI cases, 7 ST-elevation MI cases, 9 UA cases, and 7 HC blood samples.The GSE61144 dataset includes 7 patients with AMI and 10 HC.

To identify differentially expressed genes between normal blood samples and AMI blood samples, we first merged the GSE66360 and GSE60993 datasets. To reduce data dimensionality and observe the distribution characteristics of samples in high-dimensional space, we performed PCA on the raw data. Then, using the merged dataset, we conducted differential expression analysis through the 'limma' package to obtain differentially expressed genes and converted probe IDs to gene symbols. *P*-values were adjusted by the FDR (Benjamini-Hochberg) method. Differentially expressed genes were filtered with thresholds of FDR-adjusted *p*-value < 0.05 and |log_2_FC|= 1. Finally, volcano plots and heatmaps were generated using the 'ggplot2' and 'pheatmap' packages for visualization.

### KEGG analysis, GO analysis, and GSEA enrichment analysis

We performed GO and KEGG analyses using the “clusterProfiler” and “enrichplot” software packages. Both GO and KEGG enrichment analyses employed the BH-FDR method for multiple testing correction of *p*-values, with a significance threshold set at an FDR-corrected (q-value) < 0.05. Results were output as lists and visualized as bar charts/bubble charts to display the top 10–20 most significant GO terms or pathways.

GSEA was performed using the clusterProfiler (v4.x) R package. The differential measure (log_2_FC) between high- and low-risk groups (or high/low gene expression groups) was used to rank genes, generating a ranked list from largest to smallest. Gene set annotation files selected were C2.cp.kegg..gmt (KEGG pathways) and C5.go.bp.cc.mf..gmt (GO functions) from MSigDB v7.x, with species set to Homo sapiens. GSEA significance was determined using BH-FDR correction. gmt (KEGG pathways) and C5.go.bp.cc.mf..gmt (GO functions), both for Homo sapiens. GSEA significance was assessed using BH-FDR correction with a threshold of p.adjust (q value) < 0.05.

### Construction of machine learning model

We apply Elastic Net (Enet), Partial Least Squares Regression for Generalized Linear Models (plsRglm), Ridge, Stepglm, Lasso, NaiveBayes, Generalized Linear Model Boosting (glmBoost), Linear Discriminant Analysis (LDA), XGBoost, Support Vector Machine (SVM) for a total of 10 machine learning algorithms as well as 80 combinations of machine learning algorithms, to build the model.We trained and evaluated all candidate models individually using the custom function RunML. For Elastic Net, separate models were built with alpha preset between 0.1 and 0.9. Within each model, tenfold cross-validation via cv.glmnet() was performed to automatically select the optimal lambda. For other models (XGBoost, SVM, LDA, Naive Bayes, etc.), hyperparameter tuning was performed using caret::train() or the models' built-in cross-validation functions. For each candidate model, CalPredictScore was used to compute predicted risk scores for the training set and each independent validation cohort. RunEval was employed to calculate AUC, and the final model was selected based on the average AUC across all cohorts as the comprehensive evaluation metric. We merged the GSE60993 and GSE66360 datasets into a combined cohort and excluded 9 UA samples. Subsequently, this cohort was split into a training set (70%) and a testing set (30%) using a fixed random seed for model construction. Additionally, the GSE61144 dataset was selected as an external independent validation set to assess the model's generalization capability.

### Logistic model construction

Logistic Regression is a classification algorithm widely used in statistics and machine learning.After merging the entire datasets from GSE60993 and GSE66360 and excluding 9 patients with UA, the final dataset comprised 66 samples from AMI patients and 57 samples from HC. We employed logistic regression to construct a diagnostic model and evaluated its performance through tenfold cross-validation.

### Immune infiltration

The merged GEO expression profiles (GSE66360 merged with GSE60993, excluding 9 patients with UA) were deconvoluted using the CIBERSORT algorithm (LM22 feature matrix, ?-SVR linear kernel, 1,000 permutations) to calculate the relative proportions of 22 immune cell types for downstream analysis. Differences between the control and STEMI groups were compared using the Wilcoxon signed-rank test, with FDR < 0.05 considered significant. Subsequently, Spearman correlation coefficients were calculated for cell types within the STEMI group, and interaction heatmaps were generated using corrplot (hclust clustering). Further extraction of model gene lists was performed. These were collinear with the immune cell infiltration matrix, and linkET was used to construct a “gene-environment” co-occurrence network. Associations with |?|= 0.2 and FDR < 0.05 were defined as significant edges. Positive and negative correlations were distinguished by line width and color, respectively. Force-directed layout visualization displayed key gene-immune cell interaction patterns.

### Data analysis and statistics

All data processing and statistical analyses were performed in R 4.4.1. Metabolomic data were log10-transformed and Pareto-scaled prior to analysis. Differential metabolites were screened using the criteria VIP > 1.0 and |log2FC|> 0.58 (corresponding to FC > 1.5 or < 0.67). Multiple testing was corrected by the BH procedure, with q < 0.05 considered statistically significant. Correlations were assessed with Pearson’s method; raw P values were generated with psych::corr.test and further adjusted by BH-FDR, setting significance at FDR < 0.05. Machine-learning model performance was evaluated with the pROC package: ROC curves were plotted, AUC were computed, and 95% CI were derived from 2,000 bootstrap resamples. AUC comparisons between models were performed using DeLong’s test. The diagnostic logistic regression model was internally validated by tenfold cross-validation repeated 100 times; the mean AUC and its 95% CI were reported. Differences in immune-infiltration fractions were tested with the Wilcoxon rank-sum test, with FDR < 0.05 deemed significant. Gene–immune cell co-occurrence networks were constructed by retaining edges with Spearman |?|= 0.2 and FDR < 0.05. Throughout, *P* < 0.05 was considered statistically significant.

## Supplementary Information

Supplementary Material 1.

## Data Availability

All data supporting the findings of this study are available within the article and its Supplementary Information files. The datasets supporting the results of this article are available in the Gene Expression Omnibus (GEO) repository under the following accession numbers:GSE66360 (https://www.ncbi.nlm.nih.gov/geo/query/acc.cgi?acc=GSE66360), GSE60993 (https://www.ncbi.nlm.nih.gov/geo/query/acc.cgi?acc=GSE60993), GSE61144 (https://www.ncbi.nlm.nih.gov/geo/query/acc.cgi?acc=GSE61144) Supplementary Tables S1–S7 are provided with this submission: Table S1, clinical baseline characteristics; Table S2, metabolomics differential-analysis results; Table S3, Mendelian-randomization estimates after IVW screening; Table S4, differentially expressed genes identified from GEO datasets; Table S5, GO and KEGG enrichment results for the 166 differential genes; Table S6, acyl-carnitine-related gene list; Table S7, immune-infiltration analysis outputs.
