# Supplementary material for: Early diagnostic biomarkers for acute myocardial infarction unveiled by metabolomics, Mendelian randomization, and machine learning
Source: Mol Biomed. 2026 Jan 13;7:5. doi: 10.1186/s43556-025-00387-z (PMC12796025; doi:10.1186/s43556-025-00387-z)
Supplement: Supplementary file 1 — Supplementary Material 1. [file 43556_2025_387_MOESM1_ESM.docx]

****Early Diagnostic Biomarkers for Acute Myocardial Infarction Unveiled by Metabolomics, Mendelian Randomization, and Machine Learning****

Hao Fan^1,†^, Xiaoya Fu^1,†^, Qingqing Guo^1,†^, Feifan Jia^1^, Xiao-Yu Wei^1^, Jun Liu^2^, Ningxuan Zhang^1^, Chenglin Zhu^1^, Jiujin Shi^1^, Lei Zhang^1,*^, Ji-Cheng Li^1,*^
^1^ School of Basic Medical Sciences, Henan University, Kaifeng, 475004, Henan, China;

^2^ Department of Laboratory Medicine, Dongguan Hospital of Guangzhou University of Chinese Medicine, Dongguan, China;

^†^ These authors contributed equally to this work.

^*^Corresponding author:

**Ji-Cheng Li**, School of Basic Medical Sciences, Henan University, Kaifeng, 475004, Henan, China. Email: [zjulijicheng@163.com](mailto:zjulijicheng@163.com)

**Lei Zhang**, School of Basic Medical Sciences, Henan University, Kaifeng, 475004, Henan, China. Emai: [zhlei@henu.edu.cn](mailto:zhlei@henu.edu.cn)


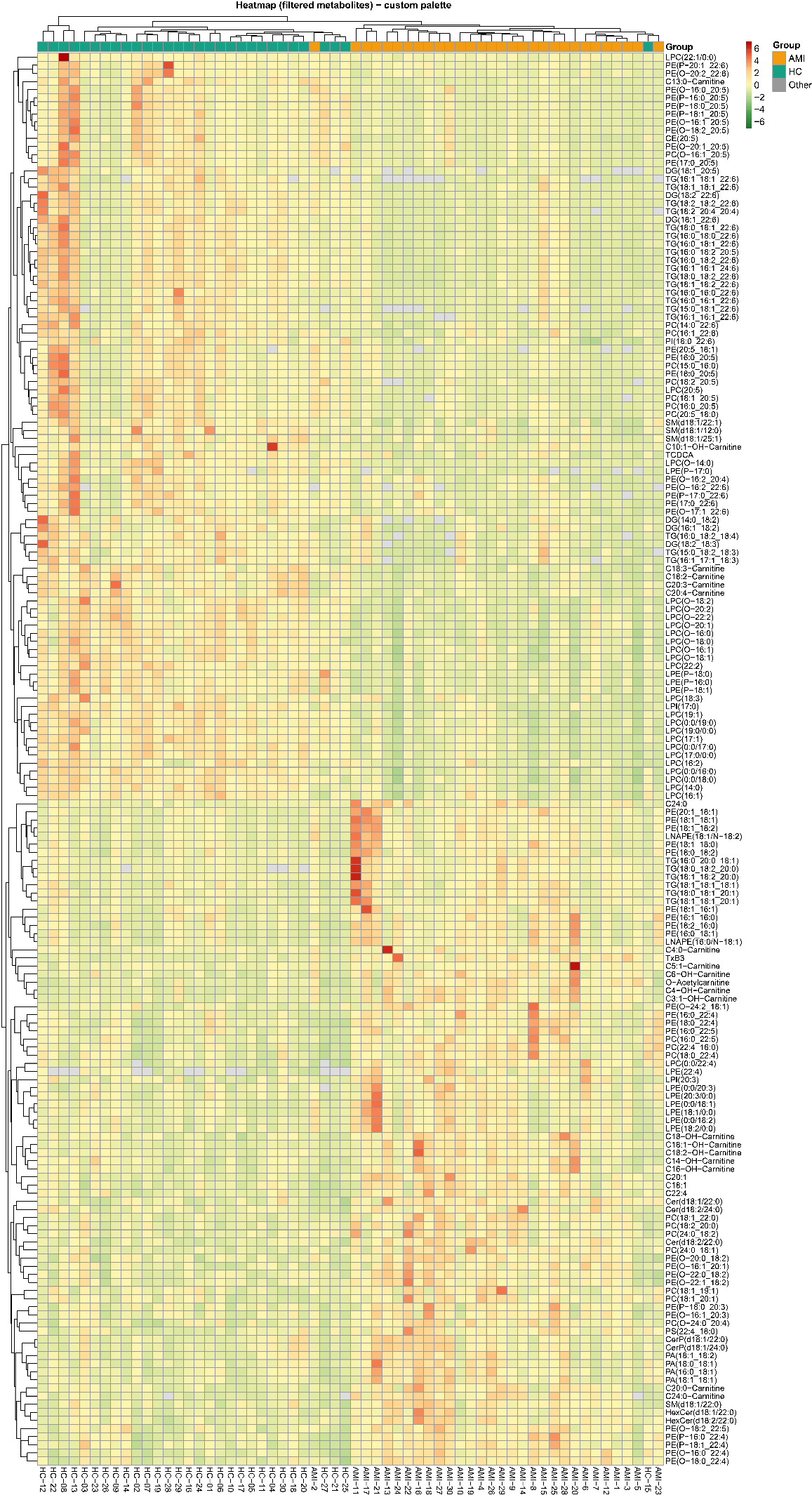


**Fig. S1 Metabolomics Heatmap** Heatmap showing 174 differentially expressed metabolites screened from the AMI and healthy control(HC) groups


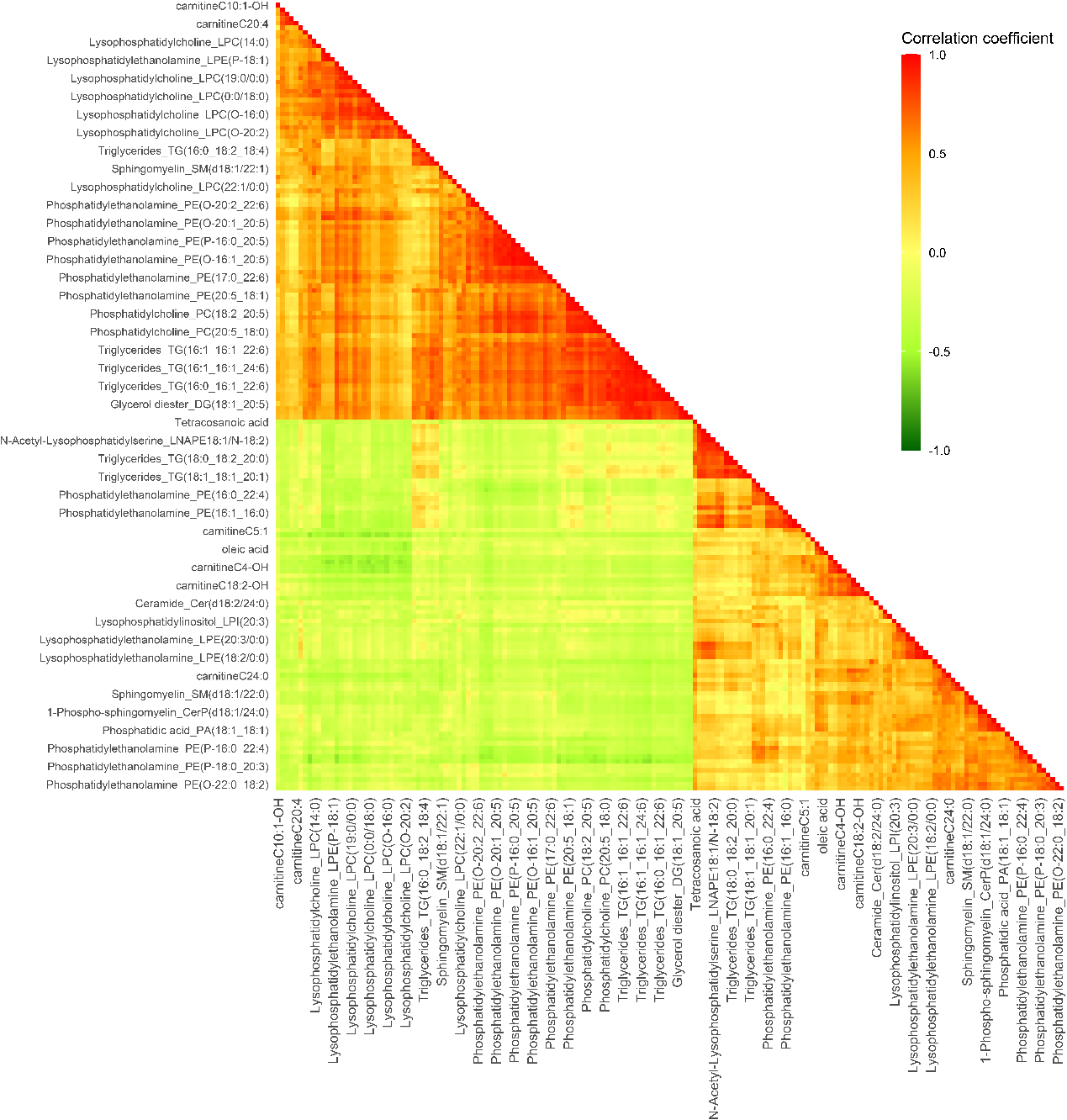


**Fig. S2 Correlation Heatmap** Heatmap illustrating relationships among the 174 differentially expressed lipid metabolites in AMI and HC groups


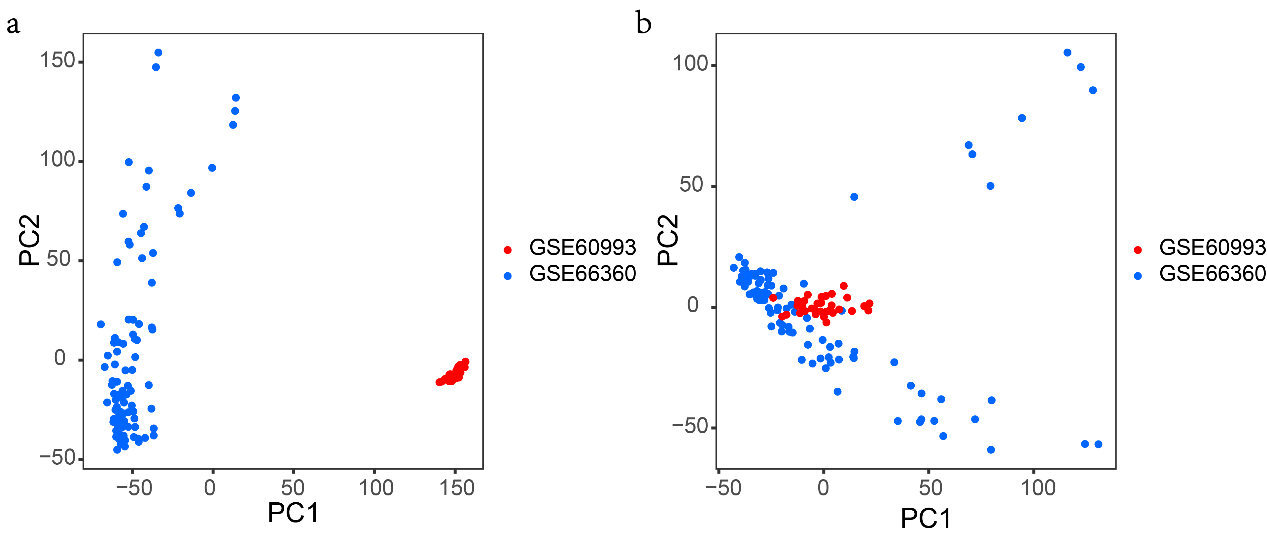


**Fig. S3** **Principal Component Analysis of Two AMI microarray datasets before and after batch effect correction** (a) Before correction using the ComBat algorithm, the samples from GSE66360 (blue) and GSE60993 (red) were completely separated on PC1, indicating the presence of a strong batch effect. (b) After correction, the sample points from both datasets were relatively evenly mixed, indicating that the batch effect had been successfully removed, and the data were suitable for integrated analysis. PCA was performed based on the expression values of common genes in the two datasets.

**Fig. S4** **Differential-gene heatmap** Heatmap showing 166 differentially expressed genes identified


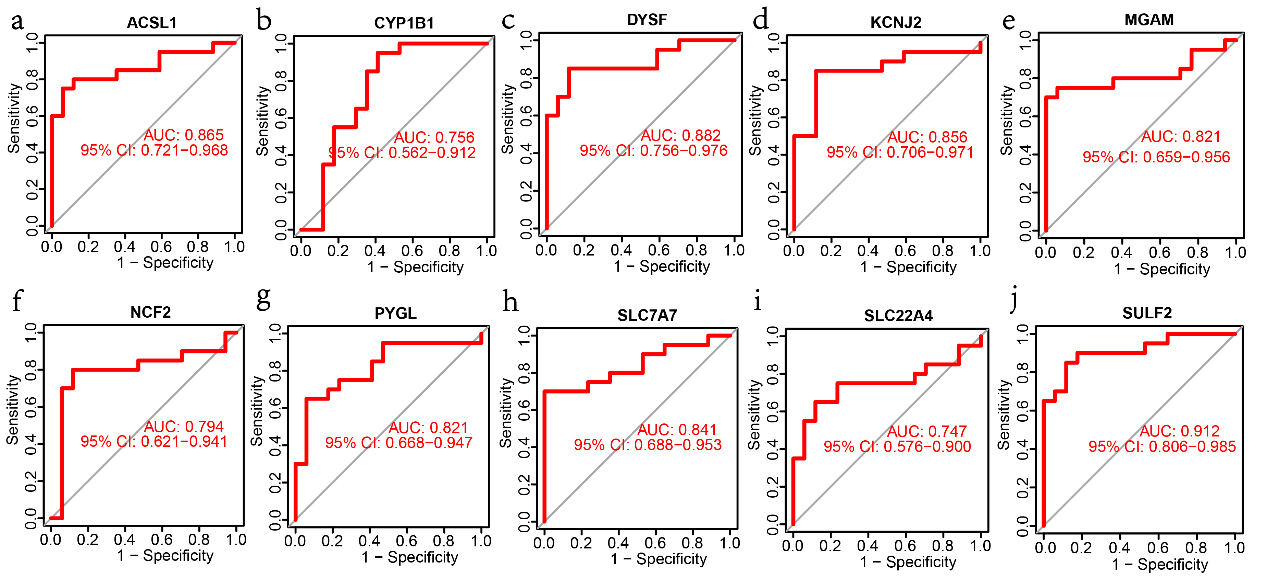


**Fig. S5 The ROC curve of the test set** (a-j) ROC figures represent the AUC values and confidence intervals of ten genes in the machine-learning diagnostic model.


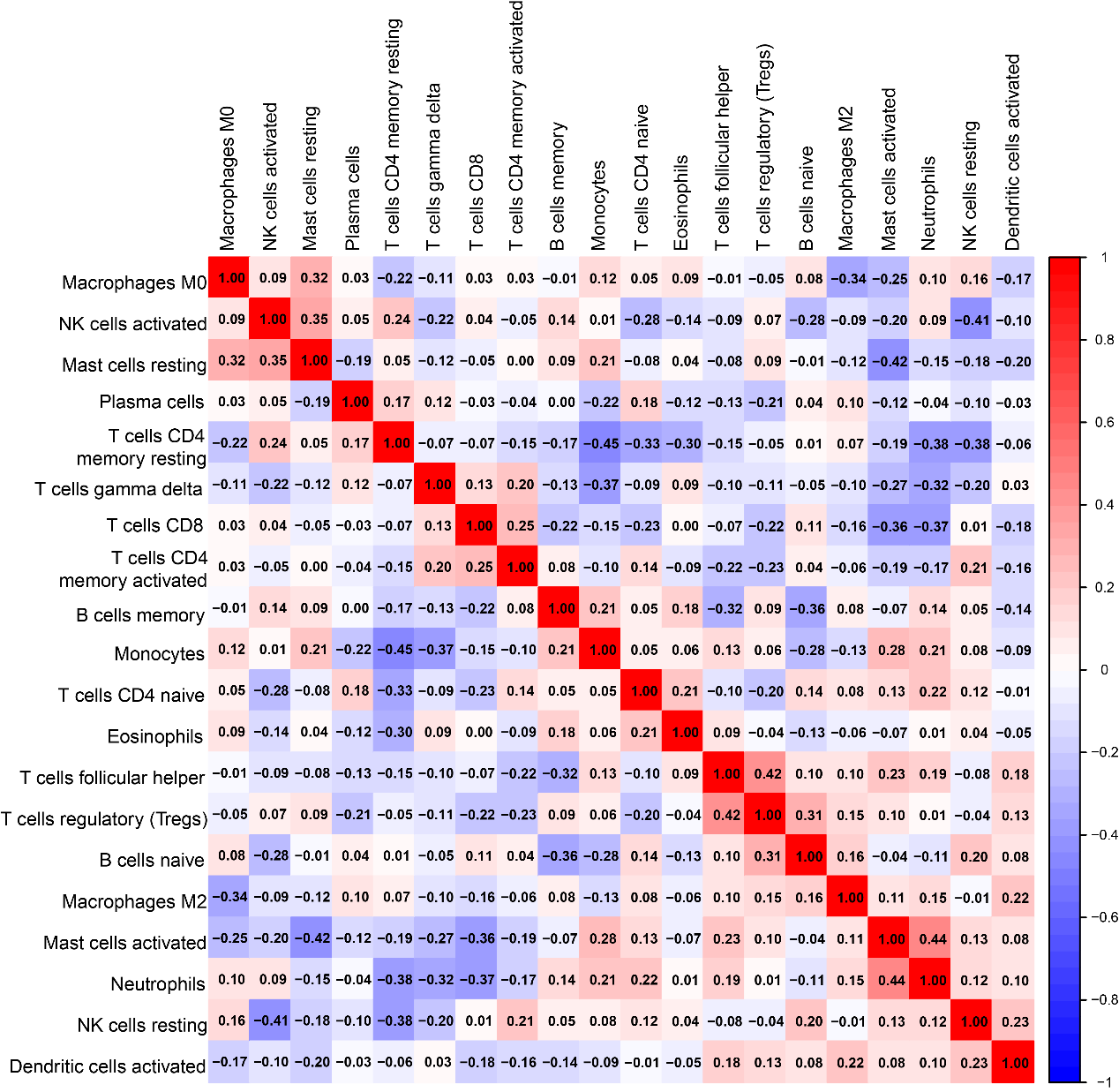


**Fig. S6** **Correlation heatmap of 22 immune cells** Red shows positive association, blue represents negative correlation, and color intensity indicates the extent of association. Levels of Mast cells activated and levels of neutrophils, extent of follicular helper T cells and extent of regulatory T cells, are all strongly positively correlated. The ratio of memory CD4+ T cells resting is negatively linked with the ratio of monocytes

**Table S1** Baseline characteristics of clinical samples in metabolomics studies

| **Characteristic** |  | **Healthy Controls(N = 30)** | **Acute myocardial infarction(N = 30)** |
| --- | --- | --- | --- |
| Sex | Male | 14 | 23 |
|  | Female | 16 | 7 |
| Age | <=60 | 18 | 14 |
|  | >60 | 12 | 16 |
| CKMB |  |  | 54.35(23.77, 194.47) |
| TC |  |  | 4.85 ± 1.05 |
| TG |  |  | 1.34 ± 0.84 |
| HDL |  |  | 1.27 ± 0.73 |
| LDL |  |  | 2.94 ± 0.9 |

**Table S2** 174 differential metabolites between the HC group and the AMI group identified by metabolomics

| **Compounds** | **Class I** | **lipidmaps ID** | **VIP** | **Pvalue** | **Fold_Change** | **Log2FC** | **q_value** |
| --- | --- | --- | --- | --- | --- | --- | --- |
| O-Acetylcarnitine | FA | LMFA07070049 | 1.78646701 | 4.562013432626601e-7 | 1.8815 | 0.9119 | 9.06700169734537e-06 |
| Carnitine C4:0 | FA | LMFA07070054 | 1.34528838 | 0.005939640203542897 | 1.9269 | 0.9463 | 0.0165684700414618 |
| Carnitine C4-OH | FA | LMFA07070071 | 2.07619438 | 1.5209162932047186e-7 | 3.0555 | 1.6114 | 3.45465272313643e-06 |
| Carnitine C6-OH | FA | LMFA07070072 | 1.89422776 | 7.082438992065286e-7 | 1.9602 | 0.971 | 1.27966795424816e-05 |
| Carnitine C13:0 | FA | - | 1.84619315 | 2.1050172387252558e-5 | 0.2652 | -1.9148 | 0.000224862706423219 |
| Carnitine C14-OH | FA | LMFA07070045 | 1.14310938 | 0.0027924392116002905 | 1.5008 | 0.5857 | 0.00887995669288892 |
| Carnitine C16-OH | FA | LMFA07070113 | 1.47463346 | 4.934098519618707e-5 | 1.8123 | 0.8578 | 0.000424296657834603 |
| Carnitine C20:0 | FA | LMFA07070052 | 1.60084355 | 1.167605607274959e-5 | 1.9166 | 0.9385 | 0.000142807147351322 |
| Carnitine C18-OH | FA | LMFA07070043 | 1.48112971 | 3.086443771509213e-5 | 1.5795 | 0.6595 | 0.000306715349793728 |
| Carnitine C3:1-OH | FA | LMFA07070080 | 2.00801602 | 2.734955061244291e-7 | 2.9571 | 1.5642 | 5.87645749645733e-06 |
| Carnitine C5:1 | FA | LMFA07070108 | 1.45184966 | 0.0051424690078821535 | 1.893 | 0.9207 | 0.0146009387902368 |
| Carnitine C10:1-OH | FA | LMFA07070047 | 1.5155534 | 0.0012424654948813925 | 0.5065 | -0.9814 | 0.00488038632809851 |
| Carnitine C18:1-OH | FA | LMFA07070025 | 2.10104956 | 3.6816768303186566e-6 | 3.2059 | 1.6807 | 5.47364487391093e-05 |
| Carnitine C18:2 | FA | LMFA07070009 | 2.03909208 | 9.47482903885017e-9 | 0.4639 | -1.1081 | 4.18471615882549e-07 |
| Carnitine C18:2-OH | FA | LMFA07070042 | 1.27389827 | 0.001787220498339687 | 1.9493 | 0.963 | 0.00612431162146574 |
| CE(20:5) | ST | LMST01020015 | 1.64144458 | 3.0812734112573054e-5 | 0.3798 | -1.3967 | 0.000306715349793728 |
| Cer(d18:1/22:0) | SP | LMSP02010008 | 1.58177297 | 1.4391708408200343e-5 | 1.5627 | 0.644 | 0.000170767286336109 |
| Cer(d18:2/22:0) | SP | LMSP02010029 | 1.82343858 | 3.9127802428229477e-7 | 1.9889 | 0.992 | 7.97605203344678e-06 |
| Cer(d18:2/24:0) | SP | - | 1.18448107 | 0.002379584960458205 | 1.5495 | 0.6318 | 0.0077850619076719 |
| DG(14:0_18:2) | GL | LMGL02010385 | 1.05690767 | 0.011084956126653785 | 0.5301 | -0.9157 | 0.0284275487764186 |
| DG(16:1_18:2) | GL | LMGL02010031 | 1.31230097 | 0.0014179040259665163 | 0.5671 | -0.8183 | 0.00519673612889607 |
| DG(18:2_18:3) | GL | LMGL02010071 | 1.130769 | 0.002082869694679765 | 0.4997 | -1.0009 | 0.00701644664097633 |
| DG(18:1_20:5) | GL | LMGL02010132 | 1.04573335 | 3.133662866463381e-4 | 0.2756 | -1.8594 | 0.00170634382112218 |
| DG(18:1_22:6) | GL | - | 1.89131951 | 2.1213462870115044e-5 | 0.2178 | -2.1989 | 0.000224862706423219 |
| DG(18:2_22:6) | GL | LMGL02010234 | 2.02876677 | 2.5587780915902463e-5 | 0.1794 | -2.4787 | 0.000267661655633453 |
| HexCer(d18:1/22:0) | SP | LMSP0501AA07 | 1.47802863 | 1.7723594257920916e-4 | 1.7322 | 0.7926 | 0.00112722059480377 |
| HexCer(d18:2/22:0) | SP | LMSP0501AA37 | 1.55719871 | 3.806074159254179e-5 | 1.7464 | 0.8044 | 0.00035184057634966 |
| LPC(14:0) | GP | LMGP01050012 | 1.99502067 | 3.3296344500454004e-8 | 0.4229 | -1.2416 | 1.01809976453311e-06 |
| LPC(0:0/16:0) | GP | LMGP01050074 | 2.0201803 | 8.667946006825936e-12 | 0.5732 | -0.8029 | 1.72275426885665e-09 |
| LPC(0:0/17:0) | GP | - | 2.06921626 | 3.857577184872668e-8 | 0.4012 | -1.3176 | 1.1358421711014e-06 |
| LPC(17:0/0:0) | GP | LMGP01050024 | 2.02979245 | 1.2530617929256737e-10 | 0.5155 | -0.956 | 1.24523015671989e-08 |
| LPC(0:0/18:0) | GP | LMGP01050076 | 1.90624966 | 1.4779046800288727e-11 | 0.5636 | -0.8273 | 2.34986844124591e-09 |
| LPC(0:0/19:0) | GP | - | 1.98125799 | 6.453582584348268e-10 | 0.4503 | -1.151 | 4.98295586299143e-08 |
| LPC(19:0/0:0) | GP | LMGP01050041 | 1.89712801 | 5.52261396775162e-9 | 0.5009 | -0.9974 | 2.58263417903679e-07 |
| LPC(16:1) | GP | LMGP01050021 | 1.7869469 | 2.9027270248946814e-8 | 0.5795 | -0.7871 | 9.23067193916509e-07 |
| LPC(17:1) | GP | LMGP01050002 | 2.1782004 | 6.894655911057322e-10 | 0.3575 | -1.484 | 4.98295586299143e-08 |
| LPC(19:1) | GP | LMGP01050130 | 1.94011494 | 9.390726449769872e-11 | 0.4778 | -1.0655 | 1.24427125459451e-08 |
| LPC(22:1/0:0) | GP | LMGP01050134 | 1.61297432 | 0.0167673525901827 | 0.4464 | -1.1636 | 0.0397911800274485 |
| LPC(22:2) | GP | LMGP01050135 | 1.90414762 | 6.596994531293659e-8 | 0.5057 | -0.9836 | 1.69180988786402e-06 |
| LPC(18:3) | GP | LMGP01050038 | 1.35359017 | 7.54022895663074e-4 | 0.5956 | -0.7476 | 0.0033430852280781 |
| LPC(0:0/22:4) | GP | - | 1.13155014 | 2.8544953922659813e-5 | 1.6297 | 0.7046 | 0.000290938953442494 |
| LPC(20:5) | GP | LMGP01050050 | 1.93231873 | 3.4193552672808644e-5 | 0.2236 | -2.161 | 0.000327516558733529 |
| LPC(O-14:0) | GP | LMGP01060007 | 2.38488649 | 3.0388989362479785e-7 | 0.138 | -2.8573 | 6.35769645872932e-06 |
| LPC(O-16:0) | GP | LMGP01060010 | 2.15520392 | 5.110995160784257e-12 | 0.5525 | -0.856 | 1.35441371760783e-09 |
| LPC(O-18:0) | GP | LMGP01060014 | 1.86657752 | 6.581695903743999e-8 | 0.57 | -0.811 | 1.69180988786402e-06 |
| LPC(O-16:1) | GP | LMGP01060028 | 1.80825788 | 1.377985375503817e-8 | 0.5821 | -0.7807 | 4.9795380614797e-07 |
| LPC(O-18:1) | GP | LMGP01060034 | 2.05835908 | 2.691027746875435e-10 | 0.5607 | -0.8347 | 2.37707450973997e-08 |
| LPC(O-20:1) | GP | - | 1.95479366 | 1.3162125509592318e-8 | 0.5303 | -0.9151 | 4.9795380614797e-07 |
| LPC(O-18:2) | GP | LMGP01070012 | 2.24681362 | 1.0397254569094575e-9 | 0.45 | -1.152 | 6.88818115202516e-08 |
| LPC(O-20:2) | GP | - | 2.11598364 | 4.440671795092438e-9 | 0.456 | -1.1329 | 2.20645879818656e-07 |
| LPC(O-22:2) | GP | - | 2.03594736 | 1.007461228957588e-8 | 0.4826 | -1.0511 | 4.21542987905938e-07 |
| LPE(0:0/18:1) | GP | LMGP02050040 | 1.43212705 | 2.3968942095480768e-4 | 1.7585 | 0.8143 | 0.00136467504453156 |
| LPE(18:1/0:0) | GP | LMGP02050004 | 1.39192837 | 1.8977403779709974e-4 | 1.7761 | 0.8287 | 0.00115332143558988 |
| LPE(0:0/18:2) | GP | LMGP02050041 | 1.21423507 | 6.378085905741239e-4 | 1.699 | 0.7647 | 0.0029826931147437 |
| LPE(18:2/0:0) | GP | LMGP02050011 | 1.33691181 | 1.253747024372714e-4 | 1.7875 | 0.8379 | 0.000859249038255438 |
| LPE(0:0/20:3) | GP | LMGP02050049 | 1.0497974 | 0.001075229815147026 | 1.7945 | 0.8436 | 0.0043391253961517 |
| LPE(20:3/0:0) | GP | LMGP02050022 | 1.18684427 | 0.001987664371795984 | 1.6564 | 0.7281 | 0.0067242262790545 |
| LPE(22:4) | GP | LMGP02050057 | 1.63705099 | 8.134950418732389e-4 | 1.7112 | 0.775 | 0.00351524914262669 |
| LPE(P-16:0) | GP | LMGP02070001 | 2.10402518 | 1.781515667829542e-9 | 0.5299 | -0.9162 | 1.08946535071114e-07 |
| LPE(P-17:0) | GP | - | 1.31339618 | 8.843864172155886e-8 | 0.3582 | -1.4812 | 2.19714750526998e-06 |
| LPE(P-18:0) | GP | LMGP02070002 | 2.1128981 | 2.358074224570891e-8 | 0.461 | -1.1172 | 7.81112086889108e-07 |
| LPE(P-18:1) | GP | - | 2.1271926 | 1.9305405079129367e-9 | 0.4796 | -1.0601 | 1.09627121699342e-07 |
| PC(O-24:0_20:4) | GP | LMGP01020254 | 1.36203649 | 1.1653454497695508e-4 | 1.5847 | 0.6642 | 0.000819866931475038 |
| PC(O-16:1_20:5) | GP | - | 1.58312666 | 3.042074107102263e-4 | 0.3626 | -1.4635 | 0.00166789580354917 |
| PE(P-18:0_20:3) | GP | LMGP02030055 | 1.00244253 | 0.0013733042546902662 | 1.5829 | 0.6626 | 0.00512571306328057 |
| PE(P-16:0_20:5) | GP | LMGP02030028 | 1.6206974 | 1.699761350984328e-4 | 0.2786 | -1.8437 | 0.0011017127670194 |
| SM(d18:1/12:0) | SP | LMSP03010002 | 1.60641864 | 1.2284077018157632e-4 | 0.5135 | -0.9616 | 0.000849203585168288 |
| SM(d18:1/22:0) | SP | LMSP03010006 | 1.45906176 | 6.484269635843291e-5 | 1.5296 | 0.6132 | 0.000531442717576847 |
| SM(d18:1/22:1) | SP | LMSP03010072 | 1.73728282 | 1.2395915238007814e-6 | 0.5775 | -0.7921 | 2.05307346129504e-05 |
| SM(d18:1/25:1) | SP | LMSP03010083 | 1.4393783 | 3.5164931822444776e-4 | 0.4583 | -1.1256 | 0.00185139872840024 |
| TG(16:0_20:0_18:1) | GL | LMGL03010205 | 1.43931969 | 0.0036124089974807387 | 2.9425 | 1.557 | 0.0109648663288453 |
| TG(18:0_18:1_20:1) | GL | LMGL03010434 | 1.1873499 | 0.009162567845207784 | 2.0313 | 1.0224 | 0.0240404007819808 |
| TG(18:0_18:2_20:0) | GL | LMGL03010427 | 1.59479376 | 0.0035455560169919036 | 5.5838 | 2.4812 | 0.0108412193596483 |
| TG(18:1_18:1_18:1) | GL | LMGL03010250 | 1.154161 | 0.004121308240572856 | 1.5634 | 0.6447 | 0.0122255225793113 |
| TG(18:1_18:2_20:0) | GL | LMGL03010478 | 1.38783809 | 0.007438284163102537 | 2.4691 | 1.304 | 0.0204617159504032 |
| TG(18:1_18:1_20:1) | GL | LMGL03010484 | 1.47646167 | 0.0015763238366508924 | 2.3223 | 1.2156 | 0.0056291450055745 |
| TG(15:0_18:2_18:3) | GL | LMGL03015166 | 1.19303312 | 0.0037893661358823216 | 0.5568 | -0.8448 | 0.0113986141370032 |
| TG(16:1_17:1_18:3) | GL | LMGL03010108 | 1.15345844 | 0.0076477176613331055 | 0.5436 | -0.8794 | 0.0208267406160844 |
| TG(16:0_18:2_18:4) | GL | LMGL03015732 | 1.25927165 | 0.01697469678400622 | 0.5383 | -0.8935 | 0.0400441660038129 |
| TG(16:0_16:0_22:6) | GL | LMGL03010526 | 1.32150709 | 0.0013976381161276748 | 0.3773 | -1.4062 | 0.00519216029122197 |
| TG(16:0_18:0_22:6) | GL | LMGL03010853 | 1.54918939 | 1.0190101583914275e-4 | 0.3587 | -1.4792 | 0.000729831599928995 |
| TG(16:0_18:2_20:5) | GL | LMGL03010451 | 1.6702148 | 3.5607055293183355e-5 | 0.2989 | -1.7423 | 0.000336995344739057 |
| TG(16:0_16:1_22:6) | GL | LMGL03010586 | 1.74754091 | 4.8332750489184284e-5 | 0.2562 | -1.9647 | 0.000422247655372544 |
| TG(15:0_18:1_22:6) | GL | LMGL03015165 | 1.23196231 | 9.972439019428913e-5 | 0.2915 | -1.7784 | 0.00072073536549509 |
| TG(16:0_18:1_22:6) | GL | LMGL03010925 | 1.5696662 | 6.886852774533575e-5 | 0.3672 | -1.4454 | 0.000553035147045878 |
| TG(18:0_18:1_22:6) | GL | LMGL03011311 | 1.33196177 | 6.014493266012978e-4 | 0.3857 | -1.3744 | 0.00284614413480971 |
| TG(16:1_16:1_22:6) | GL | LMGL03010649 | 1.72215694 | 7.439752315561379e-5 | 0.2217 | -2.1733 | 0.00059146030908713 |
| TG(16:0_18:2_22:6) | GL | LMGL03010999 | 1.8177011 | 6.712322049045326e-6 | 0.2967 | -1.7529 | 8.89382671498506e-05 |
| TG(16:1_18:1_22:6) | GL | LMGL03011000 | 1.30196302 | 4.73320839370076e-4 | 0.394 | -1.3437 | 0.00238158270442538 |
| TG(16:1_16:1_24:6) | GL | - | 1.81293242 | 8.308779263312397e-6 | 0.2941 | -1.7656 | 0.000104848881179895 |
| TG(18:1_18:1_22:6) | GL | LMGL03011389 | 1.3736166 | 6.225194782527149e-4 | 0.4315 | -1.2126 | 0.00292842003083378 |
| TG(18:0_18:2_22:6) | GL | LMGL03011388 | 1.67651349 | 1.2656151284489844e-5 | 0.3463 | -1.5299 | 0.000152449095017719 |
| TG(18:1_18:2_22:6) | GL | LMGL03011467 | 1.7696219 | 1.8249326371134804e-6 | 0.3172 | -1.6565 | 2.91037836619251e-05 |
| TG(18:2_18:2_22:6) | GL | LMGL03011544 | 1.74213976 | 6.113958829645171e-5 | 0.2395 | -2.0619 | 0.000511641817849254 |
| TG(18:2_20:4_20:4) | GL | LMGL03011316 | 1.48633205 | 7.96332092373215e-5 | 0.2806 | -1.8334 | 0.00061464467323952 |
| Carnitine C24:0 | FA | - | 1.25572968 | 1.0515223789406322e-7 | 2.5011 | 1.3226 | 2.53321300381152e-06 |
| Carnitine C18:3 | FA | LMFA07070112 | 2.03774579 | 1.3125239669619177e-8 | 0.3918 | -1.3518 | 4.9795380614797e-07 |
| Carnitine C20:3 | FA | - | 2.00601351 | 2.4126658834186454e-6 | 0.4263 | -1.2301 | 3.68859495638043e-05 |
| Carnitine C20:4 | FA | LMFA07070088 | 1.95381129 | 6.316994148155285e-7 | 0.4072 | -1.2962 | 1.16790938320545e-05 |
| PE(P-16:0_22:4) | GP | LMGP02030033 | 1.46454461 | 2.5998873288191433e-5 | 1.6866 | 0.7541 | 0.00026842992550795 |
| PE(P-18:0_20:5) | GP | LMGP02030056 | 1.56690666 | 3.585298671190665e-4 | 0.2617 | -1.934 | 0.00187230746512656 |
| PE(P-18:1_22:4) | GP | - | 1.38906354 | 8.712511070306902e-5 | 1.5219 | 0.6059 | 0.000659661552466094 |
| PE(P-18:1_20:5) | GP | - | 1.54418342 | 2.2052486515396498e-4 | 0.2952 | -1.7602 | 0.00128909755733384 |
| PE(P-17:0_22:6) | GP | - | 1.20901419 | 0.0013164635342255183 | 0.3792 | -1.399 | 0.00498375480813946 |
| PE(P-20:1_22:6) | GP | - | 1.26166423 | 0.004697783438738092 | 0.5023 | -0.9934 | 0.0135316588181043 |
| LPC(16:2) | GP | - | 1.52827416 | 1.7950455353521822e-5 | 0.4944 | -1.0162 | 0.000200994535296477 |
| Taurochenodeoxycholic acid | ST | LMST05040005 | 1.47338896 | 3.762379229381226e-4 | 0.5085 | -0.9757 | 0.00194226719958317 |
| TxB3 | FA | LMFA03030016 | 2.29752807 | 1.8751617066763585e-7 | 4.9139 | 2.2969 | 4.14098210224362e-06 |
| Tetracosanoic acid | FA | LMFA01010024 | 1.36810138 | 3.4116948465306676e-4 | 1.866 | 0.8999 | 0.00183263338039992 |
| Oleic acid | FA | LMFA01030061 | 1.37423007 | 5.2377260816725364e-5 | 1.6389 | 0.7127 | 0.000442977897332943 |
| Eicosenoic acid | FA | LMFA01030082 | 1.72337322 | 1.5110611401498478e-5 | 2.0835 | 1.059 | 0.000174100522669439 |
| Docosatetraenoic acid | FA | LMFA01030177 | 1.20822863 | 0.0010230060603286146 | 1.6677 | 0.7379 | 0.00417071701518589 |
| LPI(20:3) | GP | LMGP06050021 | 1.18584925 | 0.0015860735613191044 | 1.6008 | 0.6788 | 0.0056291450055745 |
| PC(15:0_16:0) | GP | LMGP01010532 | 1.46361894 | 6.676199265381631e-4 | 0.317 | -1.6574 | 0.0030858014046386 |
| PC(18:1_22:0) | GP | LMGP01010839 | 1.69569984 | 5.274256033764669e-6 | 2.3841 | 1.2534 | 7.6236973578962e-05 |
| PC(24:0_18:1) | GP | LMGP01011026 | 1.83790697 | 8.820343986528993e-7 | 2.2046 | 1.1405 | 1.52438553680229e-05 |
| PC(18:1_19:1) | GP | LMGP01011606 | 1.25413028 | 0.001930102720108816 | 1.5081 | 0.5927 | 0.00656627639312668 |
| PC(18:1_20:1) | GP | LMGP01010843 | 1.38466272 | 4.7829400911382166e-4 | 1.5289 | 0.6125 | 0.00239147004556911 |
| PC(18:2_20:0) | GP | LMGP01011630 | 1.48125951 | 9.917984759114174e-5 | 1.7241 | 0.7858 | 0.00072073536549509 |
| PC(24:0_18:2) | GP | LMGP01012173 | 1.21739852 | 0.0014182413512272651 | 1.7264 | 0.7878 | 0.00519673612889607 |
| PC(22:4_16:0) | GP | LMGP01012072 | 1.46087901 | 1.9562480262959433e-4 | 1.6391 | 0.7129 | 0.00116933622624457 |
| PC(18:0_22:4) | GP | LMGP01010813 | 1.36295653 | 7.605345487414537e-4 | 1.5399 | 0.6228 | 0.0033430852280781 |
| PC(16:0_20:5) | GP | LMGP01010633 | 1.46173651 | 2.017351224779025e-4 | 0.3649 | -1.4544 | 0.00119686136096965 |
| PC(16:0_22:5) | GP | LMGP01010645 | 1.47634507 | 5.814943642805218e-4 | 1.8361 | 0.8766 | 0.00278486758796997 |
| PC(20:5_18:0) | GP | LMGP01011937 | 1.48402979 | 1.8824417573475388e-4 | 0.3413 | -1.5509 | 0.00115332143558988 |
| PC(14:0_22:6) | GP | LMGP01010512 | 1.33647086 | 4.4194329139174535e-4 | 0.4836 | -1.0481 | 0.0022378657111875 |
| PC(16:1_22:6) | GP | LMGP01010696 | 1.44444101 | 9.775427668148416e-5 | 0.5067 | -0.9808 | 0.000719580092238703 |
| PC(18:2_20:5) | GP | LMGP01011634 | 1.31897485 | 1.7045367338790687e-4 | 0.3763 | -1.41 | 0.0011017127670194 |
| PE(16:1_16:0) | GP | LMGP02010520 | 1.00168022 | 0.016456134229543083 | 1.731 | 0.7916 | 0.039287167304765 |
| PE(16:0_18:1) | GP | LMGP02010009 | 1.51424379 | 2.5261376316984666e-4 | 1.8491 | 0.8868 | 0.00141985342788415 |
| PE(18:1_18:0) | GP | LMGP02010050 | 1.39348754 | 8.005260482549032e-4 | 1.7374 | 0.7969 | 0.00349680334265191 |
| PE(18:1_16:1) | GP | LMGP02011198 | 1.14534536 | 0.010690304435013722 | 1.7605 | 0.816 | 0.0275041813133848 |
| PE(18:2_16:0) | GP | LMGP02011194 | 1.53607951 | 6.249958650174662e-5 | 1.8061 | 0.8529 | 0.000517574700717589 |
| PE(18:1_18:1) | GP | LMGP02010039 | 1.7205508 | 4.924022805596724e-4 | 2.4381 | 1.2858 | 0.00243142741021702 |
| PE(18:0_18:2) | GP | LMGP02010044 | 1.07803982 | 0.004437448828147924 | 1.5011 | 0.586 | 0.0129130482377767 |
| PE(20:1_18:1) | GP | LMGP02010853 | 1.5213874 | 3.6033087064699787e-4 | 1.9692 | 0.9776 | 0.00187230746512656 |
| PE(18:1_18:2) | GP | LMGP02010048 | 1.59846748 | 1.9382272636538458e-4 | 2.0652 | 1.0463 | 0.00116734142015516 |
| PE(16:0_22:4) | GP | LMGP02010116 | 1.53190042 | 3.323060004836533e-4 | 2.2725 | 1.1843 | 0.00179716510465649 |
| PE(18:0_22:4) | GP | LMGP02011200 | 1.21608283 | 0.0017415005439792052 | 1.5911 | 0.67 | 0.0060195344889716 |
| PE(16:0_20:5) | GP | LMGP02011221 | 1.287858 | 0.0013521443382623912 | 0.3743 | -1.4177 | 0.0050945722697564 |
| PE(17:0_20:5) | GP | LMGP02010555 | 1.62713277 | 1.746887611594891e-4 | 0.3284 | -1.6065 | 0.00111998036388543 |
| PE(18:0_20:5) | GP | LMGP02010973 | 1.32610055 | 7.457186088739233e-4 | 0.3598 | -1.4747 | 0.00333059715761106 |
| PE(16:0_22:5) | GP | LMGP02011201 | 1.35197222 | 8.957487311075541e-4 | 1.9697 | 0.978 | 0.00382860344747584 |
| PE(20:5_18:1) | GP | LMGP02011172 | 1.07363259 | 0.0019327153157127576 | 0.3686 | -1.4399 | 0.00656627639312668 |
| PE(17:0_22:6) | GP | LMGP02011210 | 1.68665613 | 1.77948114854855e-5 | 0.5678 | -0.8165 | 0.000200994535296477 |
| PI(18:0_22:6) | GP | LMGP06010012 | 1.46862102 | 8.943468784027529e-6 | 0.5958 | -0.7471 | 0.000111094651301592 |
| PS(22:4_18:0) | GP | LMGP03010798 | 1.38297426 | 3.4559866280830837e-4 | 1.6459 | 0.7189 | 0.00184396601968191 |
| CerP(d18:1/22:0) | SP | LMSP02050006 | 1.08610148 | 0.003613578588877309 | 1.7969 | 0.8455 | 0.0109648663288453 |
| CerP(d18:1/24:0) | SP | LMSP02050008 | 1.0860658 | 0.002306646880709923 | 1.765 | 0.8197 | 0.00763574050854251 |
| PA(16:0_18:1) | GP | LMGP10010007 | 1.20693249 | 7.220086277154286e-4 | 1.7224 | 0.7844 | 0.00326134578996458 |
| PA(18:0_18:1) | GP | LMGP10010037 | 1.12573391 | 0.0025620795509308744 | 1.6655 | 0.736 | 0.008246369404818 |
| PA(18:1_18:1) | GP | LMGP10010962 | 1.20739784 | 5.286770504819532e-4 | 1.9588 | 0.97 | 0.00259027216501433 |
| LPI(17:0) | GP | LMGP06050029 | 2.1547539 | 1.2480033689665984e-10 | 0.4657 | -1.1025 | 1.24523015671989e-08 |
| PA(18:1_18:2) | GP | LMGP10010961 | 1.11823001 | 0.0012711173190884523 | 1.7149 | 0.7781 | 0.00490552557609378 |
| PE(O-16:1_20:1) | GP | - | 1.5929166 | 7.842210373368247e-6 | 1.6734 | 0.7428 | 0.000100557374948835 |
| PE(O-20:0_18:2) | GP | LMGP02020073 | 1.35863244 | 4.9634703369330925e-5 | 1.5104 | 0.5949 | 0.000424296657834603 |
| PE(O-22:0_18:2) | GP | - | 1.35633973 | 6.608966148158265e-5 | 1.5379 | 0.621 | 0.000536135519161818 |
| PE(O-22:1_18:2) | GP | - | 1.26392265 | 3.5007778133034706e-4 | 1.535 | 0.6182 | 0.00185139872840024 |
| PE(O-24:2_18:1) | GP | - | 1.33057194 | 7.611300959523726e-4 | 1.5599 | 0.6415 | 0.0033430852280781 |
| PE(O-16:1_20:3) | GP | - | 1.23432734 | 9.589502170633279e-5 | 1.6228 | 0.6985 | 0.000712491049126491 |
| PE(O-16:0_22:4) | GP | LMGP02020037 | 1.76458653 | 1.1818581226013761e-7 | 1.76 | 0.8156 | 2.76346237490616e-06 |
| PE(O-18:0_22:4) | GP | LMGP02020060 | 1.66065708 | 2.06770786384834e-6 | 1.5847 | 0.6642 | 3.22319167011653e-05 |
| PE(O-16:0_20:5) | GP | LMGP02020095 | 1.18188332 | 0.004300656890838423 | 0.3786 | -1.4013 | 0.0125940698663685 |
| PE(O-16:1_20:5) | GP | - | 1.37708221 | 8.167006037453172e-4 | 0.3663 | -1.4489 | 0.00351524914262669 |
| PE(O-16:2_20:4) | GP | - | 1.29341782 | 7.455617908346085e-4 | 0.4483 | -1.1575 | 0.00333059715761106 |
| PE(O-20:1_20:5) | GP | - | 1.49521253 | 5.428312269360987e-4 | 0.3781 | -1.4032 | 0.0026314074720378 |
| PE(O-18:2_20:5) | GP | - | 1.44159791 | 0.0016187053412647582 | 0.3409 | -1.5526 | 0.00571942553913548 |
| PE(O-17:1_22:6) | GP | - | 1.45335406 | 2.5360903994911917e-4 | 0.5326 | -0.9089 | 0.00141985342788415 |
| PE(O-18:2_22:5) | GP | - | 1.26125076 | 4.887498528367534e-4 | 1.6098 | 0.6869 | 0.00242847583128262 |
| PE(O-16:2_22:6) | GP | - | 1.13540741 | 1.4126132625043092e-4 | 0.3725 | -1.4247 | 0.000943720624950358 |
| PE(O-20:2_22:6) | GP | - | 1.36790714 | 0.001159409174639942 | 0.4821 | -1.0526 | 0.00460865146919377 |
| PC(18:1_20:5) | GP | LMGP01010844 | 1.24001069 | 1.9004416108462098e-4 | 0.3684 | -1.4407 | 0.00115332143558988 |
| LNAPE(16:0/N-18:1) | GP | - | 1.56326274 | 9.436301667359602e-5 | 1.9011 | 0.9268 | 0.00070772262505197 |
| LNAPE(18:1/N-18:2) | GP | - | 1.63012708 | 1.70425934937887e-4 | 2.0003 | 1.0002 | 0.0011017127670194 |

**Table S3** 37 metabolites with a causal relationship between AMI and HC identified by Mendelian randomization analysis

| **id.exposure** | **id.outcome** | **outcome** | **exposure** | **method** | **nsnp** | **b** | **se** | **pval** | **lo_ci** | **up_ci** | **or** | **or_lci95** | **or_uci95** |
| --- | --- | --- | --- | --- | --- | --- | --- | --- | --- | --- | --- | --- | --- |
| GCST90199691 | ukb-e-I21_CSA | Acute myocardial infarction | 1-methylhistidine levels | Inverse variance weighted | 26 | 0.667324425 | 0.198319808 | 0.000765741 | 0.278617601 | 1.056031249 | 1.949015601 | 1.321301983 | 2.874938404 |
| GCST90199960 | ukb-e-I21_CSA | Acute myocardial infarction | N-formylanthranilic acid levels | Inverse variance weighted | 19 | 0.474583279 | 0.154122878 | 0.002075206 | 0.172502439 | 0.776664119 | 1.607344244 | 1.188274719 | 2.174207258 |
| GCST90200211 | ukb-e-I21_CSA | Acute myocardial infarction | 3-hydroxypyridine glucuronide levels | Inverse variance weighted | 22 | -0.525405188 | 0.188217996 | 0.005246896 | -0.894312461 | -0.156497916 | 0.591315722 | 0.408888629 | 0.8551333 |
| GCST90200992 | ukb-e-I21_CSA | Acute myocardial infarction | Alanine to asparagine ratio | Inverse variance weighted | 24 | -0.478222771 | 0.17793517 | 0.007196245 | -0.826975705 | -0.129469837 | 0.61988409 | 0.437370024 | 0.878561088 |
| GCST90199628 | ukb-e-I21_CSA | Acute myocardial infarction | 3-methylhistidine levels | Inverse variance weighted | 24 | 0.539134448 | 0.200862121 | 0.007272489 | 0.14544469 | 0.932824205 | 1.714522211 | 1.156553764 | 2.54167727 |
| GCST90200121 | ukb-e-I21_CSA | Acute myocardial infarction | Ceramide (d18:1/17:0, d17:1/18:0) levels | Inverse variance weighted | 18 | 0.451905487 | 0.169106911 | 0.007533182 | 0.120455941 | 0.783355033 | 1.571303434 | 1.128011041 | 2.188803469 |
| GCST90200135 | ukb-e-I21_CSA | Acute myocardial infarction | Arachidoylcarnitine (C20) levels | Inverse variance weighted | 27 | -0.450910922 | 0.182604784 | 0.013536746 | -0.808816299 | -0.093005545 | 0.637047587 | 0.445384957 | 0.911188448 |
| GCST90200029 | ukb-e-I21_CSA | Acute myocardial infarction | Linoleoyl ethanolamide levels | Inverse variance weighted | 19 | -0.458142781 | 0.190167828 | 0.015989734 | -0.830871724 | -0.085413838 | 0.632457167 | 0.435669337 | 0.918132248 |
| GCST90200483 | ukb-e-I21_CSA | Acute myocardial infarction | X-12216 levels | Inverse variance weighted | 21 | 0.535739589 | 0.223491078 | 0.016523572 | 0.097697075 | 0.973782102 | 1.708711519 | 1.102628721 | 2.647940326 |
| GCST90200061 | ukb-e-I21_CSA | Acute myocardial infarction | 1-(1-enyl-palmitoyl)-2-oleoyl-GPE (p-16:0/18:1) levels | Inverse variance weighted | 25 | 0.485180728 | 0.205855572 | 0.018428274 | 0.081703807 | 0.888657649 | 1.624468569 | 1.085134353 | 2.431863045 |
| GCST90200321 | ukb-e-I21_CSA | Acute myocardial infarction | Cholate levels | Inverse variance weighted | 13 | 0.791202314 | 0.339199317 | 0.019671319 | 0.126371653 | 1.456032974 | 2.206047193 | 1.134703806 | 4.288911513 |
| GCST90199771 | ukb-e-I21_CSA | Acute myocardial infarction | 7-methylguanine levels | Inverse variance weighted | 18 | 0.443337867 | 0.193420239 | 0.021900071 | 0.0642342 | 0.822441535 | 1.557898609 | 1.066342107 | 2.276050116 |
| GCST90200409 | ukb-e-I21_CSA | Acute myocardial infarction | Pristanate levels | Inverse variance weighted | 10 | 0.705751929 | 0.309431743 | 0.022560283 | 0.099265713 | 1.312238146 | 2.025369046 | 1.104359703 | 3.714477958 |
| GCST90200050 | ukb-e-I21_CSA | Acute myocardial infarction | 5-hydroxyindole sulfate levels | Inverse variance weighted | 16 | 0.577067303 | 0.254034349 | 0.023109996 | 0.079159979 | 1.074974627 | 1.780808195 | 1.082377466 | 2.929918559 |
| GCST90199713 | ukb-e-I21_CSA | Acute myocardial infarction | Docosapentaenoate n3 DPA; 22:5n3 levels | Inverse variance weighted | 18 | -0.519766094 | 0.230779492 | 0.024308398 | -0.972093898 | -0.067438289 | 0.594659626 | 0.378290107 | 0.934785405 |
| GCST90200049 | ukb-e-I21_CSA | Acute myocardial infarction | Myristoyl dihydrosphingomyelin (d18:0/14:0) levels | Inverse variance weighted | 27 | 0.457923351 | 0.204188321 | 0.024919263 | 0.057714242 | 0.858132461 | 1.580787833 | 1.059412217 | 2.358751517 |
| GCST90200569 | ukb-e-I21_CSA | Acute myocardial infarction | X-18913 levels | Inverse variance weighted | 17 | 0.516833301 | 0.232293486 | 0.026086933 | 0.061538069 | 0.972128534 | 1.6767096 | 1.063470981 | 2.643565395 |
| GCST90200489 | ukb-e-I21_CSA | Acute myocardial infarction | X-12407 levels | Inverse variance weighted | 19 | 0.588427361 | 0.267351139 | 0.027739333 | 0.064419128 | 1.112435594 | 1.801153623 | 1.066539322 | 3.041757868 |
| GCST90200178 | ukb-e-I21_CSA | Acute myocardial infarction | Glucuronide of C10H18O2 (7) levels | Inverse variance weighted | 18 | -0.420532729 | 0.191528022 | 0.028115424 | -0.795927652 | -0.045137805 | 0.656696885 | 0.451162519 | 0.955865749 |
| GCST90200621 | ukb-e-I21_CSA | Acute myocardial infarction | X-23739 levels | Inverse variance weighted | 23 | 0.443047984 | 0.201884929 | 0.028195391 | 0.047353523 | 0.838742445 | 1.557447066 | 1.04849261 | 2.31345585 |
| GCST90200279 | ukb-e-I21_CSA | Acute myocardial infarction | Pantothenate levels | Inverse variance weighted | 21 | -0.357226306 | 0.162852221 | 0.028266973 | -0.676416659 | -0.038035953 | 0.699614153 | 0.50843563 | 0.962678329 |
| GCST90200499 | ukb-e-I21_CSA | Acute myocardial infarction | X-12701 levels | Inverse variance weighted | 11 | 0.608424537 | 0.283902506 | 0.032107027 | 0.051975626 | 1.164873449 | 1.837534151 | 1.053350068 | 3.205517195 |
| GCST90200680 | ukb-e-I21_CSA | Acute myocardial infarction | 5-acetylamino-6-formylamino-3-methyluracil levels | Inverse variance weighted | 18 | -0.248754952 | 0.117054692 | 0.033576826 | -0.478182149 | -0.019327755 | 0.779771031 | 0.619909271 | 0.980857828 |
| GCST90200673 | ukb-e-I21_CSA | Acute myocardial infarction | Carnitine C4 levels | Inverse variance weighted | 34 | -0.205979548 | 0.098143246 | 0.035837676 | -0.39834031 | -0.013618787 | 0.813849721 | 0.671433493 | 0.986473529 |
| GCST90199747 | ukb-e-I21_CSA | Acute myocardial infarction | Isovalerylcarnitine (C5) levels | Inverse variance weighted | 22 | -0.402067582 | 0.193744609 | 0.037963809 | -0.781807015 | -0.022328149 | 0.668935536 | 0.457578413 | 0.977919279 |
| GCST90200120 | ukb-e-I21_CSA | Acute myocardial infarction | Sphingadienine levels | Inverse variance weighted | 17 | 0.378820449 | 0.183717716 | 0.039210577 | 0.018733726 | 0.738907172 | 1.460560767 | 1.018910303 | 2.093646268 |
| GCST90200456 | ukb-e-I21_CSA | Acute myocardial infarction | X-11308 levels | Inverse variance weighted | 25 | 0.394041679 | 0.191618143 | 0.039744916 | 0.018470119 | 0.769613239 | 1.482962356 | 1.018641747 | 2.158931102 |
| GCST90199787 | ukb-e-I21_CSA | Acute myocardial infarction | Homostachydrine levels | Inverse variance weighted | 19 | -0.475066723 | 0.231291982 | 0.039978625 | -0.928399009 | -0.021734438 | 0.621843564 | 0.395185893 | 0.978500053 |
| GCST90200924 | ukb-e-I21_CSA | Acute myocardial infarction | Alpha-tocopherol to glycerol ratio | Inverse variance weighted | 19 | 0.538495548 | 0.263151879 | 0.040723928 | 0.022717865 | 1.054273231 | 1.713427154 | 1.022977881 | 2.869888651 |
| GCST90200013 | ukb-e-I21_CSA | Acute myocardial infarction | Sphingomyelin (d18:1/22:2, d18:2/22:1, d16:1/24:2) levels | Inverse variance weighted | 16 | -0.493996624 | 0.242295759 | 0.041468331 | -0.968896312 | -0.019096937 | 0.610182843 | 0.379501659 | 0.981084254 |
| GCST90200377 | ukb-e-I21_CSA | Acute myocardial infarction | Histidine levels | Inverse variance weighted | 17 | 0.397462762 | 0.195216125 | 0.04174895 | 0.014839158 | 0.780086366 | 1.488044382 | 1.014949805 | 2.18166068 |
| GCST90200722 | ukb-e-I21_CSA | Acute myocardial infarction | S-adenosylhomocysteine (SAH) to leucine ratio | Inverse variance weighted | 23 | -0.385705898 | 0.192696077 | 0.045324736 | -0.76339021 | -0.008021586 | 0.679970477 | 0.466083624 | 0.992010501 |
| GCST90200926 | ukb-e-I21_CSA | Acute myocardial infarction | N-stearoyl-sphingosine (d18:1 to 18:0) to N-palmitoyl-sphinganine (d18:0 to 16:0) ratio | Inverse variance weighted | 25 | -0.384564261 | 0.19259143 | 0.045848215 | -0.762043463 | -0.007085059 | 0.6807472 | 0.466711744 | 0.992939981 |
| GCST90199924 | ukb-e-I21_CSA | Acute myocardial infarction | N-methyltaurine levels | Inverse variance weighted | 20 | 0.377289976 | 0.189020175 | 0.045930636 | 0.006810433 | 0.747769519 | 1.458327128 | 1.006833677 | 2.11228335 |
| GCST90199849 | ukb-e-I21_CSA | Acute myocardial infarction | 21-hydroxypregnenolone disulfate levels | Inverse variance weighted | 33 | 0.317012641 | 0.159770095 | 0.047235768 | 0.003863255 | 0.630162028 | 1.373019929 | 1.003870727 | 1.87791483 |
| GCST90199718 | ukb-e-I21_CSA | Acute myocardial infarction | Sebacate (C10-DC) levels | Inverse variance weighted | 14 | -0.505205519 | 0.255383699 | 0.047903727 | -1.005757569 | -0.004653468 | 0.603381557 | 0.365767436 | 0.995357343 |
| GCST90200452 | ukb-e-I21_CSA | Acute myocardial infarction | Plasma free asparagine levels | Inverse variance weighted | 20 | 0.270595003 | 0.137244386 | 0.048651982 | 0.001596006 | 0.539594 | 1.310744116 | 1.00159728 | 1.715310305 |

**Table S4** 166 differentially expressed genes identified by GEO data analysis

| **id** | **logFC** | **AveExpr** | **t** | **P.Value** | **adj.P.Val** | **B** |
| --- | --- | --- | --- | --- | --- | --- |
| **IRAK3** | 2.03133831888403 | 7.74917217169659 | 11.2532331709847 | 9.43630274814514e-21 | 1.29635927154018e-16 | 36.2513723225541 |
| **ACSL1** | 1.85025968273943 | 8.83482431736709 | 10.3744719335619 | 1.34990601046171e-18 | 9.27250438586147e-15 | 31.4978965359388 |
| **NFIL3** | 2.25575476604204 | 8.48793713301539 | 10.0521854187225 | 8.29596627576027e-18 | 3.79899948987982e-14 | 29.7567618572664 |
| **CD55** | 1.02846453851527 | 10.0962404967593 | 9.67680233599986 | 6.82976343600779e-17 | 2.30533777757956e-13 | 27.7341300883746 |
| **NR4A2** | 1.91450304661784 | 8.03453425984424 | 9.6400679962723 | 8.39036896775207e-17 | 2.30533777757956e-13 | 27.5366228863574 |
| **S100A12** | 2.51964932879935 | 8.48325280243843 | 9.37461249119321 | 3.70032270157326e-16 | 8.47250554570223e-13 | 26.1122056990332 |
| **IL1R2** | 1.87753282787691 | 7.99536607067009 | 9.25294930023078 | 7.28937051443666e-16 | 1.43059103039044e-12 | 25.4612648526214 |
| **PPP1R15A** | 1.56142319899625 | 9.0939353296506 | 8.68588530990481 | 1.6812451253693e-14 | 2.56632728136927e-11 | 22.4474971518274 |
| **CLEC4D** | 2.01761039466747 | 6.88639890190777 | 8.33921780537536 | 1.1204783855574e-13 | 1.53931320607875e-10 | 20.6257179262626 |
| **ZFP36** | 1.50394459647267 | 10.5489844167181 | 8.30347690211499 | 1.36098123202698e-13 | 1.63042682068579e-10 | 20.4389637338138 |
| **TLR2** | 1.79740951051962 | 7.30555383808772 | 8.29513057481108 | 1.42416085661883e-13 | 1.63042682068579e-10 | 20.395383141067 |
| **BST1** | 1.80524824184819 | 7.13353502472023 | 8.25295933687961 | 1.79081414575164e-13 | 1.77982322759405e-10 | 20.1753659658292 |
| **CSTA** | 2.39867056929244 | 8.30890150333018 | 8.25061327744662 | 1.81376657346897e-13 | 1.77982322759405e-10 | 20.1631349904237 |
| **FCN1** | 1.70670671305824 | 8.81954735893384 | 8.23239194795162 | 2.00227621886699e-13 | 1.83381804631965e-10 | 20.0681721949616 |
| **GADD45A** | 1.26595338950125 | 8.95356089333396 | 8.08708483099356 | 4.39569469807145e-13 | 3.7742533601316e-10 | 19.3130055242032 |
| **IL1B** | 1.98221960466952 | 9.59905912718311 | 8.04080262148406 | 5.64204691038602e-13 | 4.55943767381666e-10 | 19.0732942072773 |
| **AQP9** | 2.07396635193408 | 9.01078464795862 | 8.00366665091583 | 6.89113078144547e-13 | 5.20791459945337e-10 | 18.8812513153019 |
| **CCL20** | 1.99937678357072 | 7.86267225740359 | 7.99544898936427 | 7.20267705558407e-13 | 5.20791459945337e-10 | 18.8387912079394 |
| **PYGL** | 1.72366343787376 | 7.91114036763495 | 7.97302936744525 | 8.12559864051145e-13 | 5.58147370616732e-10 | 18.7230180495824 |
| **FOS** | 1.80388364849591 | 8.79883585140775 | 7.89330643433697 | 1.24649211297448e-12 | 7.86812225763777e-10 | 18.3121496597717 |
| **SRGN** | 2.07537832447749 | 11.0870488417219 | 7.89129523142299 | 1.25999919688478e-12 | 7.86812225763777e-10 | 18.3018012263942 |
| **FTH1** | 2.64407282830228 | 9.67482362387328 | 7.83626667621604 | 1.69159412155825e-12 | 1.01039652356379e-09 | 18.0189834198574 |
| **THBD** | 1.42853978698645 | 6.38777371237921 | 7.80395664084869 | 2.01037484477677e-12 | 1.15077206739763e-09 | 17.8532237936286 |
| **IRS2** | 1.71985007982251 | 10.2032522944447 | 7.77930397180172 | 2.29309238919394e-12 | 1.26010012970985e-09 | 17.7268989029462 |
| **TREM1** | 2.13079262196874 | 7.60979293424344 | 7.74746482312832 | 2.71730522613144e-12 | 1.43578227679207e-09 | 17.5639441904512 |
| **C5AR1** | 1.9800796999992 | 9.64510542900547 | 7.71649175514541 | 3.20446949464962e-12 | 1.63048155249987e-09 | 17.405635843886 |
| **S100A9** | 1.64483246451774 | 10.6589129282689 | 7.6759133616047 | 3.97595690060581e-12 | 1.88350675519044e-09 | 17.1985569400988 |
| **CDA** | 1.39090619827357 | 7.51110161227807 | 7.63778497513941 | 4.86767488270629e-12 | 2.22907058462064e-09 | 17.0043209231106 |
| **DYSF** | 1.64435447918772 | 8.24385096590344 | 7.56987543633625 | 6.97392415363927e-12 | 3.09057322653859e-09 | 16.659205228646 |
| **JDP2** | 1.08352044300121 | 6.55972427900683 | 7.54881009488457 | 7.79506252646603e-12 | 3.34651778089345e-09 | 16.5523721885759 |
| **PILRA** | 1.47740477129648 | 8.45071194537702 | 7.53861247499689 | 8.22631316053052e-12 | 3.42463909695056e-09 | 16.5006929723389 |
| **S100P** | 1.84155984454663 | 8.66499834814177 | 7.46205961976542 | 1.23142440753798e-11 | 4.97567897375199e-09 | 16.1135459916554 |
| **MMP9** | 1.41487564767692 | 7.80718109702944 | 7.37871301789681 | 1.90737835612432e-11 | 7.48673253041027e-09 | 15.6936960191087 |
| **MGAM** | 1.80033689784834 | 7.64779715012981 | 7.35089020307651 | 2.20648443745388e-11 | 8.4201897782615e-09 | 15.5539353565778 |
| **NR4A3** | 1.28325051301456 | 6.85917343442427 | 7.32748356128895 | 2.49376568331068e-11 | 9.25928458306003e-09 | 15.4365136732356 |
| **CSF3R** | 1.81719922169814 | 9.0389379691052 | 7.32124150116467 | 2.57644210590445e-11 | 9.31451622392508e-09 | 15.4052239555027 |
| **RBP7** | 1.48029069804543 | 8.12952883502507 | 7.28247332632053 | 3.15423987258915e-11 | 1.11110121460589e-08 | 15.2111199009209 |
| **CXCL16** | 1.64989378926033 | 9.15923256929577 | 7.22886671686754 | 4.16980155868625e-11 | 1.43211834533079e-08 | 14.9433843213772 |
| **FCER1G** | 1.90515220685745 | 10.1869426280549 | 7.20797357538993 | 4.6480358748436e-11 | 1.55743211825857e-08 | 14.8392455269659 |
| **SLC7A7** | 1.52581833043141 | 8.59183255415135 | 7.19707649889126 | 4.91861317979553e-11 | 1.6088549491436e-08 | 14.7849781241388 |
| **TLR4** | 1.28800900296668 | 7.67310362055929 | 7.17013258540236 | 5.65640963606705e-11 | 1.80715710651835e-08 | 14.6509383117495 |
| **THBS1** | 1.31718491146154 | 7.32352145240112 | 7.15733602121909 | 6.04418580771889e-11 | 1.88715965060096e-08 | 14.5873490033297 |
| **ADAM9** | 1.1024308622113 | 6.97413163348914 | 7.13582829282765 | 6.75605667815155e-11 | 2.06254903654324e-08 | 14.4805750922374 |
| **SULF2** | 1.39122557968302 | 7.24046468428115 | 7.1157571359165 | 7.49493646071026e-11 | 2.19565330039023e-08 | 14.3810506526281 |
| **ANPEP** | 1.04424041519566 | 7.56554265983941 | 7.11532489619422 | 7.51169785400648e-11 | 2.19565330039023e-08 | 14.378908613816 |
| **EFEMP1** | 1.45428779595762 | 5.31018149556316 | 7.06807637082511 | 9.58621687360424e-11 | 2.74365515436615e-08 | 14.1450824937546 |
| **IER3** | 1.62812080163077 | 10.2404114369495 | 7.03377221939622 | 1.14384266275972e-10 | 3.20696132673327e-08 | 13.9757203502168 |
| **CMTM2** | 1.65977167854968 | 9.04486119070221 | 6.96593771462273 | 1.62044064392538e-10 | 4.45232271324938e-08 | 13.6418359388611 |
| **GLT1D1** | 1.42013394721837 | 6.34436354045017 | 6.95359109343086 | 1.72624142909809e-10 | 4.65002053979403e-08 | 13.5812130642537 |
| **LRG1** | 1.22385193754115 | 7.05621937249444 | 6.92687974675177 | 1.97906515189572e-10 | 5.18215870965898e-08 | 13.4502159945637 |
| **MCEMP1** | 1.23092773944125 | 7.99371596717912 | 6.92489672928016 | 1.99923141368413e-10 | 5.18215870965898e-08 | 13.4404995788518 |
| **TP53INP2** | 1.68798746418708 | 8.03767947369784 | 6.87755339818246 | 2.54581581841268e-10 | 6.47674402099138e-08 | 13.2088841605884 |
| **ANXA3** | 1.67785467741698 | 5.89924612068564 | 6.81206579908212 | 3.55241024631144e-10 | 8.87327490251393e-08 | 12.8896510778234 |
| **QPCT** | 1.57809807868407 | 6.80439690626689 | 6.79017457936415 | 3.9697401755384e-10 | 9.73862330920475e-08 | 12.7832398499324 |
| **ALDH2** | 1.655584697 | 7.46392235838468 | 6.77103997773099 | 4.37393777656363e-10 | 1.0541957399023e-07 | 12.6903538419237 |
| **LILRA2** | 1.21878831120984 | 7.75813465117055 | 6.74111261410606 | 5.08901175432808e-10 | 1.20539385311999e-07 | 12.5453127956088 |
| **IFNGR1** | 1.05934651784206 | 10.4427179388768 | 6.73349329844212 | 5.28878335019219e-10 | 1.23147975703289e-07 | 12.5084327095905 |
| **PMAIP1** | 1.12487862962776 | 8.68444680404813 | 6.69006262149492 | 6.58442923573407e-10 | 1.50761481400858e-07 | 12.2985767016598 |
| **TMCC3** | 1.02925202820643 | 7.08890521997416 | 6.68107447157413 | 6.88936127299714e-10 | 1.55157451095795e-07 | 12.2552239048909 |
| **WDFY3** | 1.14391025691265 | 6.78246756238619 | 6.59379249949381 | 1.06781545874438e-09 | 2.3660723826178e-07 | 11.8356428798492 |
| **CCL4** | 1.44550570154177 | 8.31628632303221 | 6.58874421554778 | 1.09514092825732e-09 | 2.38810255117446e-07 | 11.8114538554158 |
| **EOMES** | -1.358612538 | 9.64211842457929 | -6.562146987 | 1.25090473304108e-09 | 2.6851451910185e-07 | 11.6841569657988 |
| **HBEGF** | 1.07466109046323 | 6.60672770915946 | 6.52888886332307 | 1.47670752101626e-09 | 3.12107814211098e-07 | 11.5253249499626 |
| **DDIT3** | 1.41700044257752 | 8.06764880597594 | 6.48515338807413 | 1.83576274276525e-09 | 3.82116796365287e-07 | 11.3170454454449 |
| **CD14** | 1.45721267296029 | 8.51425837003717 | 6.47303234535369 | 1.94967200840295e-09 | 3.99770060469249e-07 | 11.2594416508636 |
| **CH25H** | 1.03695552326341 | 5.08783428921081 | 6.46006453109996 | 2.07925729429963e-09 | 4.20071128074828e-07 | 11.1978716357414 |
| **RAB32** | 1.27299275742294 | 7.381092741 | 6.41298728393613 | 2.62510276469633e-09 | 5.1895776504934e-07 | 10.9748606479848 |
| **S100A8** | 1.56842562723291 | 11.9706075685126 | 6.39820185459983 | 2.82404153769692e-09 | 5.46432149927892e-07 | 10.9049857283705 |
| **TULP2** | 1.2061612518942 | 5.97675717784852 | 6.39210734966839 | 2.91030164976762e-09 | 5.55301723118161e-07 | 10.8762067216788 |
| **VNN1** | 1.48969533246893 | 6.40093258070595 | 6.3822796629012 | 3.05489877030105e-09 | 5.67137828464808e-07 | 10.8298277948802 |
| **VNN3** | 1.19726306092277 | 7.350961944 | 6.35477288388727 | 3.49830627024213e-09 | 6.33834148558684e-07 | 10.7002062523465 |
| **TNFAIP3** | 1.41077578375146 | 10.5361977551951 | 6.35430139532429 | 3.5064343638419e-09 | 6.33834148558684e-07 | 10.697986868031 |
| **CD163** | 1.2544138345889 | 6.8596908856979 | 6.34235237792905 | 3.71874997356537e-09 | 6.63482949829104e-07 | 10.6417681265766 |
| **TM6SF1** | 1.43763135052165 | 6.61214307176112 | 6.25150915272859 | 5.80434456584681e-09 | 9.96751070570044e-07 | 10.2161109109756 |
| **PTAFR** | 1.14340493530606 | 7.47068433072588 | 6.23802793129932 | 6.19917090274494e-09 | 1.05140999829518e-06 | 10.1532097319631 |
| **LILRA5** | 1.29769394543778 | 7.35103755015648 | 6.21852955432418 | 6.81738504182581e-09 | 1.14216141103174e-06 | 10.0623570397534 |
| **RORC** | -1.018872724 | 7.49897660154173 | -6.212549131 | 7.01887864640531e-09 | 1.16175126318453e-06 | 10.0345206515892 |
| **CXCL1** | 1.56432097498123 | 7.47972312430576 | 6.18872315361882 | 7.88145184447532e-09 | 1.27382806399296e-06 | 9.92375831395268 |
| **FOSB** | 1.19042472703106 | 7.67280713008429 | 6.18435286640392 | 8.05062063177039e-09 | 1.28200179107109e-06 | 9.90346567605464 |
| **BCL2A1** | 1.37741365679262 | 9.41343618572391 | 6.18262056093403 | 8.11866034526021e-09 | 1.28200179107109e-06 | 9.89542409245356 |
| **LILRB2** | 1.10924820201533 | 7.67545850226101 | 6.13796533178604 | 1.00816082223503e-08 | 1.57387651998463e-06 | 9.68853523002064 |
| **SLC2A3** | 1.0780050771336 | 10.4928205448123 | 6.10921361323209 | 1.15852187975072e-08 | 1.72738268825552e-06 | 9.55574504677306 |
| **P2RY13** | 1.54293676622146 | 6.03887365005718 | 6.03270152177278 | 1.67448247967263e-08 | 2.42147792692028e-06 | 9.20398973769296 |
| **MPEG1** | 1.51212684137281 | 8.11903041574967 | 6.02363507101853 | 1.74892154106503e-08 | 2.50277959699494e-06 | 9.16246535405906 |
| **AREG** | 1.15756866715866 | 6.56030276774795 | 5.98016464252799 | 2.15347701082112e-08 | 3.01882318108781e-06 | 8.96384035150731 |
| **ADM** | 1.60047869053565 | 9.25833130330902 | 5.97297516737553 | 2.22871378968482e-08 | 3.09273434774647e-06 | 8.93106557371971 |
| **NLRP3** | 1.04097019888737 | 8.02796520664755 | 5.9500261991731 | 2.48656093526654e-08 | 3.33704660076312e-06 | 8.82659188471024 |
| **CD33** | 1.1234042291708 | 6.59077881245031 | 5.947307032 | 2.51898961961712e-08 | 3.33704660076312e-06 | 8.81422764668835 |
| **ZNF578** | -1.010489838 | 6.04957289184066 | -5.944696275 | 2.55051603639633e-08 | 3.33704660076312e-06 | 8.80235927261757 |
| **CDKN1A** | 1.28753858371485 | 7.8038769369745 | 5.93728204516449 | 2.64217478808795e-08 | 3.39197210462313e-06 | 8.76867015205233 |
| **GZMA** | -1.034559985 | 11.1459648068116 | -5.927061315 | 2.77385085570562e-08 | 3.48499050726698e-06 | 8.72226663544063 |
| **ICAM1** | 1.09220097516438 | 7.11657082676568 | 5.92580851988746 | 2.79042768815961e-08 | 3.48499050726698e-06 | 8.71658180515764 |
| **FOLR3** | 1.54158610928444 | 7.26139140461889 | 5.89268657570471 | 3.26574597054393e-08 | 4.04187550840833e-06 | 8.56652469781936 |
| **NFKBIZ** | 1.47376677357485 | 9.48095688577247 | 5.87331470033873 | 3.57967600956958e-08 | 4.3908561624524e-06 | 8.47897785468286 |
| **GIMAP7** | -1.179412763 | 9.32546588158494 | -5.855731919 | 3.89013453383194e-08 | 4.72943966599851e-06 | 8.39965559867981 |
| **HAL** | 1.16186315139857 | 7.48759103517443 | 5.85349024214324 | 3.93156736698629e-08 | 4.73788355154891e-06 | 8.38955214333127 |
| **VSIG1** | -1.1066961 | 6.71950769857994 | -5.765685304 | 5.94374059161186e-08 | 6.97906908098836e-06 | 7.99552432266603 |
| **FCGR3B** | 1.46741797794713 | 9.39904453688197 | 5.76065125615263 | 6.08565380774126e-08 | 7.02560605132348e-06 | 7.97303629927079 |
| **ITLN1** | 1.80587564234675 | 6.9998873525125 | 5.75378793424373 | 6.28449943305197e-08 | 7.19470443427233e-06 | 7.94239464174133 |
| **PLAUR** | 1.08553572557352 | 8.17035856660253 | 5.74470329295757 | 6.55752299902101e-08 | 7.44522735211163e-06 | 7.90186791176602 |
| **CD300LF** | 1.32432589949611 | 6.69756067112313 | 5.69223762738827 | 8.37718118032544e-08 | 9.20685720442488e-06 | 7.66853835225695 |
| **GCA** | 1.2884367637809 | 9.06725537290863 | 5.65853348645874 | 9.79819734014503e-08 | 1.05162214889775e-05 | 7.51930044072088 |
| **CEBPB** | 1.30608749862004 | 10.4593540648615 | 5.65006733955481 | 1.01907220106041e-07 | 1.08527239520682e-05 | 7.48189444125158 |
| **MME** | 1.02860145687616 | 7.13569371269987 | 5.64269127499235 | 1.05452288432533e-07 | 1.1069998601633e-05 | 7.44933141730708 |
| **PELI1** | 1.12664148286578 | 9.44139130986829 | 5.64247298834618 | 1.05559020003925e-07 | 1.1069998601633e-05 | 7.44836812866168 |
| **TYROBP** | 1.42370033624695 | 10.7554211367979 | 5.57984995168331 | 1.40978541543501e-07 | 1.45621293513129e-05 | 7.17292067254176 |
| **HIST2H2BE** | 1.14901570139964 | 9.53601490777489 | 5.57359605574178 | 1.45097458843075e-07 | 1.47655473302679e-05 | 7.14551259894465 |
| **PTX3** | 1.72891893519518 | 6.66818101739048 | 5.553540761 | 1.59116502835439e-07 | 1.56138751139519e-05 | 7.05774217272305 |
| **CRTAM** | -1.105447779 | 6.45407376009907 | -5.438488968 | 2.69124211855342e-07 | 2.43246046799564e-05 | 6.55790020373617 |
| **KCNJ2** | 1.41442244063143 | 8.6710242703049 | 5.4297013113033 | 2.80075905532999e-07 | 2.510336746103e-05 | 6.51998275483073 |
| **CCR5** | -1.017272023 | 7.82033081846344 | -5.427232234 | 2.83230598082665e-07 | 2.510336746103e-05 | 6.50933577777064 |
| **RGS1** | -2.080461647 | 7.07255502694955 | -5.416949031 | 2.96746836663521e-07 | 2.61327438595093e-05 | 6.46502508027509 |
| **FAM49A** | 1.01946267251467 | 7.33057351026268 | 5.41360891882854 | 3.01271168382509e-07 | 2.63621866957893e-05 | 6.45064346762517 |
| **PLXDC2** | 1.04445053471789 | 7.78009081704035 | 5.41101894104063 | 3.04825733543835e-07 | 2.6504404603957e-05 | 6.43949546327053 |
| **RNASE2** | 1.45250657774898 | 7.52371478207906 | 5.40584558263436 | 3.1204889299161e-07 | 2.69249488198483e-05 | 6.4172376273691 |
| **CCRL2** | 1.1082468405714 | 6.42446427692906 | 5.40453393545041 | 3.13906653927327e-07 | 2.69249488198483e-05 | 6.41159647418643 |
| **CLEC4A** | 1.0162945613192 | 8.16139123460496 | 5.3979600564683 | 3.2338160978854e-07 | 2.69249488198483e-05 | 6.3833360737916 |
| **SIGLEC5** | 1.08848869164933 | 6.76760411262871 | 5.36954435964464 | 3.67652319722174e-07 | 3.0244356696666e-05 | 6.2614233117469 |
| **ARRDC4** | 1.18271622745699 | 6.13521788652377 | 5.33553005922778 | 4.28467544176207e-07 | 3.46252183640749e-05 | 6.11601338732972 |
| **B3GALT2** | -1.166384272 | 6.40128725387206 | -5.289122935 | 5.27509277442369e-07 | 4.18897251647588e-05 | 5.91855193947397 |
| **TOB2** | 1.2140415910532 | 7.21624613035801 | 5.22610441377985 | 6.98457939589977e-07 | 5.33078620782617e-05 | 5.65214400147533 |
| **GZMK** | -1.224424435 | 10.5301810940948 | -5.10439135 | 1.19446665361453e-06 | 8.4151707114648e-05 | 5.14337541310506 |
| **ST6GAL1** | 1.72867370572437 | 6.39515912375718 | 5.10081956978971 | 1.21328744186186e-06 | 8.50415452872357e-05 | 5.12856174825601 |
| **HCK** | 1.00777974512871 | 8.05978450718029 | 5.09962332338045 | 1.21965511875153e-06 | 8.50539188903985e-05 | 5.12360191683157 |
| **G0S2** | 1.0118073148715 | 9.29432041151808 | 5.07489538177822 | 1.35881073226679e-06 | 9.24125833657482e-05 | 5.0212450777639 |
| **RNF175** | 1.02348160147229 | 7.59479190030906 | 5.01846612242397 | 1.7367011885459e-06 | 0.000113801255661285 | 4.78888338759116 |
| **CPVL** | 1.31233089355451 | 7.36427301790342 | 5.00940096696546 | 1.80625060373375e-06 | 0.00011760317911893 | 4.75171426450983 |
| **GIMAP4** | -1.241222255 | 10.4066406488078 | -5.005113887 | 1.84007780968476e-06 | 0.000118126116586212 | 4.7341517086228 |
| **CYP1B1** | 1.07090362546821 | 7.39012451862002 | 5.00315009744262 | 1.85577802646444e-06 | 0.000118579900128226 | 4.72611010837135 |
| **CD83** | 1.28612598912844 | 8.78729393640716 | 4.99308164962774 | 1.93834169010966e-06 | 0.000122714000639293 | 4.68491312040861 |
| **RGS2** | 1.15823448087394 | 11.1358019780044 | 4.98226850127711 | 2.0309878644322e-06 | 0.000127989501291604 | 4.64073010134876 |
| **GLUL** | 1.08941561272835 | 7.68064047841655 | 4.96550549754423 | 2.18317790973104e-06 | 0.00013632953692675 | 4.57236107146871 |
| **NFKBIA** | 1.25130902557495 | 10.0306075101863 | 4.93645948653829 | 2.47351274122577e-06 | 0.000149696555237707 | 4.4542574800578 |
| **CSF2RB** | 1.11056339808325 | 8.60558658318699 | 4.92661761641058 | 2.58014320791303e-06 | 0.00015411307561004 | 4.41434432001406 |
| **NCALD** | -1.019229192 | 7.88171303278482 | -4.88161108 | 3.12737872797207e-06 | 0.00018360653403795 | 4.2325028241333 |
| **CD1D** | 1.15143652678693 | 6.79160796679699 | 4.74007333770947 | 5.68597092063886e-06 | 0.000311210631504927 | 3.66802914165129 |
| **C15orf48** | 1.24362132576062 | 7.29117492995177 | 4.72147777692138 | 6.14556280724472e-06 | 0.000331089183709521 | 3.59471360173917 |
| **NCF2** | 1.1202462863626 | 8.84872491695078 | 4.71174027940347 | 6.40037328240308e-06 | 0.000342133572582309 | 3.55640162242545 |
| **BTG1** | 1.17767253694113 | 10.7818692431467 | 4.6363550479872 | 8.75028854597794e-06 | 0.000437132596525982 | 3.26166211832363 |
| **PDGFC** | 1.03480217454478 | 6.12924581213283 | 4.62772043464947 | 9.06756321904753e-06 | 0.000451341244577083 | 3.2281147040393 |
| **MANSC1** | 1.0268785756913 | 7.50670192461625 | 4.58746068133162 | 1.06996903176193e-05 | 0.000512168451510291 | 3.07227708945967 |
| **CNOT1** | 1.05505820350381 | 7.25277490783116 | 4.5855256658511 | 1.0784896304984e-05 | 0.000514454532770383 | 3.06481119530216 |
| **FGR** | 1.30733726527456 | 7.80216343996939 | 4.54109958374821 | 1.29315147480395e-05 | 0.000604262413634581 | 2.89401513529216 |
| **SLC22A4** | 1.10280504312504 | 6.28861203564911 | 4.40871115105375 | 2.20616001792511e-05 | 0.000913475667948151 | 2.39211320386936 |
| **CHIC1** | 1.19028623120357 | 6.79468231373752 | 4.39119769887971 | 2.36588163788736e-05 | 0.00096446533950435 | 2.32652092316651 |
| **EGR3** | 1.03773001556351 | 7.66415784910766 | 4.33880101328361 | 2.91300262521451e-05 | 0.00114013760869507 | 2.13141943281263 |
| **USP9Y** | 1.36738383308642 | 6.16390254106682 | 4.32895006749839 | 3.02863991112811e-05 | 0.00117868144756595 | 2.09493042163648 |
| **STEAP4** | 1.07105883041649 | 6.96265204632861 | 4.31511374298563 | 3.19853989363909e-05 | 0.00122399835818423 | 2.04378198430416 |
| **LRRK2** | 1.22306673554856 | 8.64637898928214 | 4.30395694487213 | 3.34217424335978e-05 | 0.00125793944535004 | 2.0026266212643 |
| **RPS16** | 1.53774225603442 | 11.5165605641137 | 4.29351125229216 | 3.48225863401402e-05 | 0.00129165085821436 | 1.96416556197462 |
| **SULF1** | 1.02188048471869 | 6.34216361540496 | 4.27051179865954 | 3.8108596670468e-05 | 0.00137534750301999 | 1.87972487469108 |
| **XIST** | -1.364754292 | 6.04750770444839 | -4.204222922 | 4.9330556938491e-05 | 0.00169255498618998 | 1.63823569377772 |
| **NUP54** | -1.029022937 | 8.03333947837851 | -4.194214071 | 5.1279008625313e-05 | 0.00174398262527709 | 1.60201850539394 |
| **EGR1** | 1.34698380762468 | 5.98690263665093 | 4.17893431839765 | 5.43965638611623e-05 | 0.00183611792217358 | 1.54685314759407 |
| **CARD8** | 1.04162734849915 | 8.49904834807362 | 4.15941835283705 | 5.86433649589873e-05 | 0.00193888592694493 | 1.47661330640515 |
| **MTIF3** | 1.49433620038835 | 6.63358498851378 | 4.05906561322088 | 8.5995354119259e-05 | 0.0026022118389656 | 1.11936201137284 |
| **SERPINB2** | 1.0720229882391 | 5.09197727141739 | 4.05680170315274 | 8.67349517462477e-05 | 0.00261882366393396 | 1.11137906142605 |
| **MNDA** | 1.00722741139598 | 9.1901752041893 | 4.05175498086192 | 8.84056176022541e-05 | 0.00264681084227096 | 1.09359564997617 |
| **LYZ** | 1.40549497035675 | 8.93231196495383 | 3.90598758197337 | 0.000152326092164652 | 0.00407131489135796 | 0.58730640604313 |
| **RPS4Y1** | 1.98960741936185 | 9.92617506387559 | 3.83873443815925 | 0.000194893444264882 | 0.00489478270075127 | 0.358580865754082 |
| **WDR3** | 1.54028790967519 | 5.76590354983467 | 3.79301928529895 | 0.000230046484940743 | 0.00549631062628856 | 0.204886591209251 |
| **ZFY** | 1.1386073801782 | 5.71644641764228 | 3.76193132158343 | 0.000257306343221382 | 0.00596100260231931 | 0.101199750788789 |
| **AIF1** | -1.300958774 | 7.06025809249869 | -3.74817821 | 0.000270319859744526 | 0.00621012413573629 | 0.055545148080717 |
| **GJA1** | 1.04171835465505 | 7.22046577819238 | 3.69376788067886 | 0.000328182877037029 | 0.00724851505584358 | -0.123769078 |
| **EIF1AY** | 1.23763110484341 | 6.99371839242118 | 3.32574436224992 | 0.00115616124828175 | 0.0184261522376968 | -1.280493202 |
| **PPP2R5C** | 1.00828825774358 | 8.0964145214615 | 3.24157930835824 | 0.00152179797867517 | 0.0224800651946661 | -1.530893271 |

**Table S5** KEGG and GO analysis results files

| KEGG |  |  |  |  |  |  |  |  |  |  |  |  |  |
| --- | --- | --- | --- | --- | --- | --- | --- | --- | --- | --- | --- | --- | --- |
| category | subcategory | ID | Description | GeneRatio | BgRatio | RichFactor | FoldEnrichment | zScore | pvalue | p.adjust | qvalue | geneID | Count |
| Organismal Systems | Immune system | hsa04657 | IL-17 signaling pathway | 11/103 | 95/9446 | 0.115789 | 10.61891 | 9.893157 | 5.50E-09 | 1.23E-06 | 9.32E-07 | IL1B/CCL20/FOS/S100A9/MMP9/S100A8/TNFAIP3/CXCL1/FOSB/CEBPB/NFKBIA | 11 |
| Environmental Information Processing | Signal transduction | hsa04064 | NF-kappa B signaling pathway | 10/103 | 105/9446 | 0.095238 | 8.734166 | 8.367348 | 1.86E-07 | 2.09E-05 | 1.58E-05 | GADD45A/IL1B/TLR4/CCL4/CD14/TNFAIP3/CXCL1/BCL2A1/ICAM1/NFKBIA | 10 |
| Organismal Systems | Development and regeneration | hsa04380 | Osteoclast differentiation | 11/103 | 143/9446 | 0.076923 | 7.054518 | 7.659713 | 3.95E-07 | 2.95E-05 | 2.23E-05 | IL1B/FOS/LILRA2/IFNGR1/LILRA5/FOSB/LILRB2/FCGR3B/TYROBP/NFKBIA/NCF2 | 11 |
| Organismal Systems | Immune system | hsa04640 | Hematopoietic cell lineage | 9/103 | 100/9446 | 0.09 | 8.253786 | 7.656465 | 1.28E-06 | 7.17E-05 | 5.42E-05 | CD55/IL1R2/IL1B/CSF3R/ANPEP/CD14/CD33/MME/CD1D | 9 |
| Human Diseases | Infectious disease: parasitic | hsa05140 | Leishmaniasis | 8/103 | 79/9446 | 0.101266 | 9.286961 | 7.765778 | 2.09E-06 | 9.37E-05 | 7.09E-05 | TLR2/IL1B/FOS/TLR4/IFNGR1/FCGR3B/NFKBIA/NCF2 | 8 |
| Human Diseases | Cardiovascular disease | hsa05417 | Lipid and atherosclerosis | 12/103 | 216/9446 | 0.055556 | 5.09493 | 6.392179 | 3.69E-06 | 0.000138 | 0.000104 | TLR2/IL1B/FOS/MMP9/TLR4/DDIT3/CD14/CXCL1/NLRP3/ICAM1/NFKBIA/NCF2 | 12 |
| Environmental Information Processing | Signal transduction | hsa04668 | TNF signaling pathway | 9/103 | 119/9446 | 0.07563 | 6.935955 | 6.841786 | 5.48E-06 | 0.000176 | 0.000133 | IL1B/CCL20/FOS/MMP9/TNFAIP3/CXCL1/ICAM1/CEBPB/NFKBIA | 9 |
| Human Diseases | Infectious disease: bacterial | hsa05134 | Legionellosis | 6/103 | 56/9446 | 0.107143 | 9.825936 | 6.955021 | 3.03E-05 | 0.000849 | 0.000642 | TLR2/IL1B/TLR4/CD14/CXCL1/NFKBIA | 6 |
| Human Diseases | Cancer: overview | hsa05202 | Transcriptional misregulation in cancer | 10/103 | 201/9446 | 0.049751 | 4.562624 | 5.360323 | 6.43E-05 | 0.001599 | 0.00121 | IL1R2/GADD45A/MMP9/NR4A3/DDIT3/CD14/BCL2A1/CDKN1A/NFKBIZ/CEBPB | 10 |
| Human Diseases | Immune disease | hsa05323 | Rheumatoid arthritis | 7/103 | 95/9446 | 0.073684 | 6.757486 | 5.921641 | 7.52E-05 | 0.001684 | 0.001274 | TLR2/IL1B/CCL20/FOS/TLR4/CXCL1/ICAM1 | 7 |
| Human Diseases | Infectious disease: parasitic | hsa05146 | Amoebiasis | 7/103 | 103/9446 | 0.067961 | 6.232633 | 5.606245 | 0.000126 | 0.002557 | 0.001935 | IL1R2/TLR2/IL1B/TLR4/CD14/CXCL1/CD1D | 7 |
| Organismal Systems | Immune system | hsa04620 | Toll-like receptor signaling pathway | 7/103 | 109/9446 | 0.06422 | 5.889552 | 5.390823 | 0.000179 | 0.003342 | 0.002529 | TLR2/IL1B/FOS/TLR4/CCL4/CD14/NFKBIA | 7 |
| Human Diseases | Infectious disease: parasitic | hsa05144 | Malaria | 5/103 | 50/9446 | 0.1 | 9.170874 | 6.082168 | 0.000201 | 0.003458 | 0.002616 | TLR2/IL1B/TLR4/THBS1/ICAM1 | 5 |
| Human Diseases | Infectious disease: viral | hsa05171 | Coronavirus disease - COVID-19 | 10/103 | 238/9446 | 0.042017 | 3.853308 | 4.680917 | 0.000259 | 0.004136 | 0.003129 | TLR2/IL1B/FOS/C5AR1/TLR4/HBEGF/NLRP3/NFKBIA/RPS16/RPS4Y1 | 10 |
| Human Diseases | Infectious disease: viral | hsa05167 | Kaposi sarcoma-associated herpesvirus infection | 9/103 | 196/9446 | 0.045918 | 4.211116 | 4.769683 | 0.000277 | 0.004136 | 0.003129 | ZFP36/FOS/IFNGR1/CXCL1/CDKN1A/ICAM1/CCR5/HCK/NFKBIA | 9 |
| Cellular Processes | Cell growth and death | hsa04217 | Necroptosis | 8/103 | 159/9446 | 0.050314 | 4.614276 | 4.825681 | 0.000332 | 0.004644 | 0.003514 | IL1B/PYGL/FTH1/TLR4/IFNGR1/TNFAIP3/NLRP3/GLUL | 8 |
| Cellular Processes | Cell growth and death | hsa04210 | Apoptosis | 7/103 | 137/9446 | 0.051095 | 4.685848 | 4.562707 | 0.000721 | 0.009179 | 0.006945 | GADD45A/FOS/PMAIP1/DDIT3/BCL2A1/NFKBIA/CSF2RB | 7 |
| Human Diseases | Immune disease | hsa05321 | Inflammatory bowel disease | 5/103 | 66/9446 | 0.075758 | 6.947632 | 5.090859 | 0.000738 | 0.009179 | 0.006945 | TLR2/IL1B/TLR4/IFNGR1/RORC | 5 |
| Human Diseases | Infectious disease: bacterial | hsa05152 | Tuberculosis | 8/103 | 182/9446 | 0.043956 | 4.031153 | 4.335316 | 0.000815 | 0.009202 | 0.006962 | TLR2/IL1B/FCER1G/TLR4/IFNGR1/CD14/FCGR3B/CEBPB | 8 |
| Human Diseases | Infectious disease: parasitic | hsa05142 | Chagas disease | 6/103 | 103/9446 | 0.058252 | 5.342257 | 4.652296 | 0.000884 | 0.009202 | 0.006962 | TLR2/IL1B/FOS/TLR4/IFNGR1/NFKBIA | 6 |
| Human Diseases | Cardiovascular disease | hsa05418 | Fluid shear stress and atherosclerosis | 7/103 | 142/9446 | 0.049296 | 4.520853 | 4.438474 | 0.000891 | 0.009202 | 0.006962 | IL1R2/IL1B/FOS/THBD/MMP9/ICAM1/NCF2 | 7 |
| Human Diseases | Endocrine and metabolic disease | hsa04936 | Alcoholic liver disease | 7/103 | 144/9446 | 0.048611 | 4.458064 | 4.390384 | 0.000968 | 0.009202 | 0.006962 | IL1B/C5AR1/TLR4/ALDH2/CD14/CXCL1/NFKBIA | 7 |
| Organismal Systems | Immune system | hsa04625 | C-type lectin receptor signaling pathway | 6/103 | 105/9446 | 0.057143 | 5.240499 | 4.587662 | 0.000977 | 0.009202 | 0.006962 | CLEC4D/IL1B/FCER1G/NLRP3/NFKBIA/EGR3 | 6 |
| Human Diseases | Cancer: specific types | hsa05219 | Bladder cancer | 4/103 | 41/9446 | 0.097561 | 8.947194 | 5.354301 | 0.00099 | 0.009202 | 0.006962 | MMP9/THBS1/HBEGF/CDKN1A | 4 |
| Human Diseases | Cancer: specific types | hsa05215 | Prostate cancer | 6/103 | 106/9446 | 0.056604 | 5.191061 | 4.55596 | 0.001027 | 0.009202 | 0.006962 | IL1R2/MMP9/CDKN1A/FOLR3/NFKBIA/PDGFC | 6 |
| Organismal Systems | Immune system | hsa04062 | Chemokine signaling pathway | 8/103 | 193/9446 | 0.041451 | 3.801398 | 4.128466 | 0.001191 | 0.010264 | 0.007766 | CCL20/CXCL16/CCL4/CXCL1/CCR5/HCK/NFKBIA/FGR | 8 |
| Environmental Information Processing | Signaling molecules and interaction | hsa04060 | Cytokine-cytokine receptor interaction | 10/103 | 298/9446 | 0.033557 | 3.077474 | 3.826107 | 0.001483 | 0.012301 | 0.009307 | IL1R2/IL1B/CCL20/CSF3R/CXCL16/IFNGR1/CCL4/CXCL1/CCR5/CSF2RB | 10 |
| Human Diseases | Infectious disease: bacterial | hsa05133 | Pertussis | 5/103 | 78/9446 | 0.064103 | 5.878765 | 4.54266 | 0.001572 | 0.012574 | 0.009514 | IL1B/FOS/TLR4/CD14/NLRP3 | 5 |
| Human Diseases | Infectious disease: viral | hsa05161 | Hepatitis B | 7/103 | 163/9446 | 0.042945 | 3.938412 | 3.973185 | 0.001982 | 0.015312 | 0.011585 | TLR2/FOS/MMP9/TLR4/CDKN1A/NFKBIA/EGR3 | 7 |
| Human Diseases | Cancer: specific types | hsa05210 | Colorectal cancer | 5/103 | 87/9446 | 0.057471 | 5.270617 | 4.201573 | 0.002547 | 0.019017 | 0.014388 | GADD45A/FOS/PMAIP1/AREG/CDKN1A | 5 |
| Organismal Systems | Immune system | hsa04610 | Complement and coagulation cascades | 5/103 | 88/9446 | 0.056818 | 5.210724 | 4.166611 | 0.002677 | 0.019346 | 0.014637 | CD55/THBD/C5AR1/PLAUR/SERPINB2 | 5 |
| Human Diseases | Cancer: overview | hsa05235 | PD-L1 expression and PD-1 checkpoint pathway in cancer | 5/103 | 90/9446 | 0.055556 | 5.09493 | 4.098256 | 0.002953 | 0.020668 | 0.015637 | TLR2/FOS/TLR4/IFNGR1/NFKBIA | 5 |
| Organismal Systems | Immune system | hsa04662 | B cell receptor signaling pathway | 5/103 | 91/9446 | 0.054945 | 5.038942 | 4.064834 | 0.003098 | 0.021027 | 0.015908 | FOS/LILRA2/LILRA5/LILRB2/NFKBIA | 5 |
| Human Diseases | Infectious disease: viral | hsa05162 | Measles | 6/103 | 139/9446 | 0.043165 | 3.958651 | 3.689544 | 0.004054 | 0.026705 | 0.020205 | TLR2/IL1B/FOS/TLR4/TNFAIP3/NFKBIA | 6 |
| Organismal Systems | Immune system | hsa04621 | NOD-like receptor signaling pathway | 7/103 | 189/9446 | 0.037037 | 3.39662 | 3.49439 | 0.004522 | 0.02894 | 0.021896 | IL1B/TLR4/TNFAIP3/CXCL1/NLRP3/NFKBIA/CARD8 | 7 |
| Organismal Systems | Immune system | hsa04659 | Th17 cell differentiation | 5/103 | 109/9446 | 0.045872 | 4.206823 | 3.535582 | 0.006674 | 0.041286 | 0.031236 | IL1B/FOS/IFNGR1/RORC/NFKBIA | 5 |
| Human Diseases | Cancer: overview | hsa05205 | Proteoglycans in cancer | 7/103 | 204/9446 | 0.034314 | 3.146868 | 3.254722 | 0.00682 | 0.041286 | 0.031236 | TLR2/MMP9/TLR4/THBS1/HBEGF/CDKN1A/PLAUR | 7 |
| Human Diseases | Infectious disease: parasitic | hsa05145 | Toxoplasmosis | 5/103 | 112/9446 | 0.044643 | 4.09414 | 3.45853 | 0.00747 | 0.044035 | 0.033316 | TLR2/TLR4/IFNGR1/CCR5/NFKBIA | 5 |
| Cellular Processes | Transport and catabolism | hsa04145 | Phagosome | 6/103 | 159/9446 | 0.037736 | 3.460707 | 3.285468 | 0.007739 | 0.044448 | 0.033629 | TLR2/TLR4/THBS1/CD14/FCGR3B/NCF2 | 6 |
| Organismal Systems | Endocrine system | hsa04928 | Parathyroid hormone synthesis, secretion and action | 5/103 | 115/9446 | 0.043478 | 3.987336 | 3.384117 | 0.008331 | 0.046652 | 0.035296 | NR4A2/FOS/HBEGF/CDKN1A/EGR1 | 5 |
| Cellular Processes | Cell growth and death | hsa04115 | p53 signaling pathway | 4/103 | 75/9446 | 0.053333 | 4.891133 | 3.55214 | 0.008967 | 0.048988 | 0.037063 | GADD45A/THBS1/PMAIP1/CDKN1A | 4 |
| Human Diseases | Infectious disease: viral | hsa05166 | Human T-cell leukemia virus 1 infection | 7/103 | 224/9446 | 0.03125 | 2.865898 | 2.967398 | 0.011113 | 0.059268 | 0.044841 | IL1R2/ZFP36/FOS/CDKN1A/ICAM1/NFKBIA/EGR1 | 7 |
| Human Diseases | Infectious disease: viral | hsa05164 | Influenza A | 6/103 | 173/9446 | 0.034682 | 3.18065 | 3.039312 | 0.011462 | 0.059708 | 0.045174 | IL1B/TLR4/IFNGR1/NLRP3/ICAM1/NFKBIA | 6 |
| Organismal Systems | Immune system | hsa04623 | Cytosolic DNA-sensing pathway | 4/103 | 83/9446 | 0.048193 | 4.419698 | 3.285457 | 0.012692 | 0.064613 | 0.048885 | IL1B/CCL4/NLRP3/NFKBIA | 4 |
|  |  |  |  |  |  |  |  |  |  |  |  |  |  |
| GO |  |  |  |  |  |  |  |  |  |  |  |  |  |
| ONTOLOGY | ID | Description | GeneRatio | BgRatio | RichFactor | FoldEnrichment | zScore | pvalue | p.adjust | qvalue | geneID | Count |  |
| BP | GO:0002237 | response to molecule of bacterial origin | 28/155 | 369/18870 | 0.075881 | 9.23787 | 14.54373 | 2.82E-19 | 8.17E-16 | 6.01E-16 | CD55/ZFP36/TLR2/IL1B/FOS/THBD/C5AR1/S100A9/MMP9/TLR4/ADAM9/LILRA2/CD14/S100A8/TNFAIP3/PTAFR/CXCL1/LILRB2/ADM/NLRP3/NFKBIZ/CEBPB/PELI1/CCR5/HCK/NFKBIA/CSF2RB/CARD8 | 28 |  |
| BP | GO:0032496 | response to lipopolysaccharide | 27/155 | 348/18870 | 0.077586 | 9.445495 | 14.47159 | 7.41E-19 | 8.87E-16 | 6.52E-16 | CD55/ZFP36/TLR2/IL1B/FOS/THBD/S100A9/MMP9/TLR4/ADAM9/LILRA2/CD14/S100A8/TNFAIP3/PTAFR/CXCL1/LILRB2/ADM/NLRP3/NFKBIZ/CEBPB/PELI1/CCR5/HCK/NFKBIA/CSF2RB/CARD8 | 27 |  |
| BP | GO:0001819 | positive regulation of cytokine production | 31/155 | 499/18870 | 0.062124 | 7.563126 | 13.52197 | 9.19E-19 | 8.87E-16 | 6.52E-16 | CD55/TLR2/FCN1/IL1B/C5AR1/NR4A3/FCER1G/TLR4/THBS1/SULF2/LILRA2/IFNGR1/DDIT3/CD14/PTAFR/LILRA5/LILRB2/NLRP3/CEBPB/PELI1/TYROBP/CRTAM/ARRDC4/CYP1B1/CD83/FGR/LRRK2/SULF1/EGR1/CARD8/AIF1 | 31 |  |
| BP | GO:0071216 | cellular response to biotic stimulus | 21/155 | 265/18870 | 0.079245 | 9.647474 | 12.90158 | 4.97E-15 | 3.60E-12 | 2.64E-12 | CD55/ZFP36/TLR2/IL1B/CDA/MMP9/TLR4/ADAM9/LILRA2/DDIT3/CD14/TNFAIP3/PTAFR/CXCL1/LILRB2/NLRP3/NFKBIZ/CCR5/HCK/NFKBIA/CARD8 | 21 |  |
| BP | GO:0002274 | myeloid leukocyte activation | 20/155 | 241/18870 | 0.082988 | 10.10307 | 12.94335 | 9.60E-15 | 5.56E-12 | 4.09E-12 | S100A12/CLEC4D/TLR2/C5AR1/DYSF/NR4A3/FCER1G/TLR4/THBS1/ADAM9/ANXA3/LILRA2/IFNGR1/PTAFR/CD33/CD300LF/TYROBP/FGR/LRRK2/AIF1 | 20 |  |
| BP | GO:0050867 | positive regulation of cell activation | 24/155 | 396/18870 | 0.060606 | 7.378299 | 11.67394 | 1.97E-14 | 9.49E-12 | 6.98E-12 | CD55/CLEC4D/BST1/IL1B/IRS2/NR4A3/TLR4/THBS1/LILRA2/VNN1/PTAFR/LILRA5/LILRB2/NLRP3/CDKN1A/NFKBIZ/PELI1/TYROBP/CD83/CD1D/FGR/EGR3/LRRK2/AIF1 | 24 |  |
| BP | GO:0071222 | cellular response to lipopolysaccharide | 19/155 | 226/18870 | 0.084071 | 10.23494 | 12.71055 | 3.68E-14 | 1.52E-11 | 1.12E-11 | CD55/ZFP36/TLR2/IL1B/MMP9/TLR4/ADAM9/LILRA2/CD14/TNFAIP3/PTAFR/CXCL1/LILRB2/NLRP3/NFKBIZ/CCR5/HCK/NFKBIA/CARD8 | 19 |  |
| BP | GO:0006935 | chemotaxis | 25/155 | 468/18870 | 0.053419 | 6.503309 | 10.97134 | 9.38E-14 | 2.99E-11 | 2.20E-11 | S100A12/BST1/IL1B/CCL20/TREM1/C5AR1/S100A9/CSF3R/CXCL16/FCER1G/THBS1/CMTM2/CCL4/HBEGF/CH25H/S100A8/PTAFR/CXCL1/PLAUR/CCR5/RNASE2/CCRL2/ST6GAL1/EGR3/AIF1 | 25 |  |
| BP | GO:0071219 | cellular response to molecule of bacterial origin | 19/155 | 239/18870 | 0.079498 | 9.678229 | 12.28733 | 1.02E-13 | 2.99E-11 | 2.20E-11 | CD55/ZFP36/TLR2/IL1B/MMP9/TLR4/ADAM9/LILRA2/CD14/TNFAIP3/PTAFR/CXCL1/LILRB2/NLRP3/NFKBIZ/CCR5/HCK/NFKBIA/CARD8 | 19 |  |
| BP | GO:0042330 | taxis | 25/155 | 470/18870 | 0.053191 | 6.475635 | 10.94006 | 1.03E-13 | 2.99E-11 | 2.20E-11 | S100A12/BST1/IL1B/CCL20/TREM1/C5AR1/S100A9/CSF3R/CXCL16/FCER1G/THBS1/CMTM2/CCL4/HBEGF/CH25H/S100A8/PTAFR/CXCL1/PLAUR/CCR5/RNASE2/CCRL2/ST6GAL1/EGR3/AIF1 | 25 |  |
| BP | GO:0002696 | positive regulation of leukocyte activation | 22/155 | 380/18870 | 0.057895 | 7.048217 | 10.83917 | 6.63E-13 | 1.75E-10 | 1.28E-10 | CD55/CLEC4D/BST1/IL1B/IRS2/NR4A3/TLR4/THBS1/VNN1/PTAFR/LILRB2/NLRP3/CDKN1A/NFKBIZ/PELI1/TYROBP/CD83/CD1D/FGR/EGR3/LRRK2/AIF1 | 22 |  |
| BP | GO:0031349 | positive regulation of defense response | 24/155 | 480/18870 | 0.05 | 6.087097 | 10.27413 | 1.27E-12 | 3.08E-10 | 2.26E-10 | S100A12/TLR2/FCN1/IL1B/S100A9/TLR4/LILRA2/CD14/S100A8/TNFAIP3/LILRA5/NLRP3/NFKBIZ/CD300LF/CEBPB/PELI1/TYROBP/CRTAM/HCK/NFKBIA/CD1D/LRRK2/CARD8/MNDA | 24 |  |
| BP | GO:0060326 | cell chemotaxis | 20/155 | 325/18870 | 0.061538 | 7.491811 | 10.74335 | 2.63E-12 | 5.87E-10 | 4.31E-10 | S100A12/BST1/IL1B/CCL20/TREM1/C5AR1/S100A9/CSF3R/CXCL16/FCER1G/THBS1/CCL4/HBEGF/CH25H/S100A8/CXCL1/CCR5/CCRL2/EGR3/AIF1 | 20 |  |
| BP | GO:0050900 | leukocyte migration | 21/155 | 396/18870 | 0.05303 | 6.456012 | 9.985913 | 1.22E-11 | 2.53E-09 | 1.86E-09 | S100A12/BST1/IL1B/CCL20/TREM1/C5AR1/S100A9/CSF3R/CXCL16/FCER1G/THBS1/CCL4/CH25H/S100A8/PTAFR/CXCL1/ICAM1/CRTAM/CCR5/HCK/AIF1 | 21 |  |
| BP | GO:0030595 | leukocyte chemotaxis | 17/155 | 240/18870 | 0.070833 | 8.623387 | 10.81664 | 1.39E-11 | 2.68E-09 | 1.97E-09 | S100A12/BST1/IL1B/CCL20/TREM1/C5AR1/S100A9/CSF3R/CXCL16/FCER1G/THBS1/CCL4/CH25H/S100A8/CXCL1/CCR5/AIF1 | 17 |  |
| BP | GO:0031663 | lipopolysaccharide-mediated signaling pathway | 10/155 | 58/18870 | 0.172414 | 20.98999 | 13.87569 | 3.88E-11 | 7.03E-09 | 5.17E-09 | CD55/TLR2/TLR4/LILRA2/CD14/TNFAIP3/PTAFR/HCK/NFKBIA/CARD8 | 10 |  |
| BP | GO:0071621 | granulocyte chemotaxis | 13/155 | 130/18870 | 0.1 | 12.17419 | 11.63452 | 5.34E-11 | 9.09E-09 | 6.68E-09 | S100A12/BST1/IL1B/CCL20/TREM1/C5AR1/S100A9/CSF3R/FCER1G/THBS1/CCL4/S100A8/CXCL1 | 13 |  |
| BP | GO:0070663 | regulation of leukocyte proliferation | 17/155 | 267/18870 | 0.06367 | 7.751359 | 10.11115 | 7.47E-11 | 1.19E-08 | 8.72E-09 | CD55/BST1/IL1B/IRS2/TLR4/TNFAIP3/LILRB2/CDKN1A/CEBPB/PELI1/TYROBP/CRTAM/ST6GAL1/CSF2RB/CD1D/MNDA/AIF1 | 17 |  |
| BP | GO:0030593 | neutrophil chemotaxis | 12/155 | 107/18870 | 0.11215 | 13.6533 | 11.9451 | 7.78E-11 | 1.19E-08 | 8.72E-09 | S100A12/BST1/IL1B/CCL20/TREM1/C5AR1/S100A9/CSF3R/FCER1G/CCL4/S100A8/CXCL1 | 12 |  |
| BP | GO:0006909 | phagocytosis | 16/155 | 237/18870 | 0.067511 | 8.218865 | 10.17764 | 1.17E-10 | 1.69E-08 | 1.25E-08 | TLR2/FCN1/IL1B/DYSF/FCER1G/TLR4/THBS1/ANXA3/CD14/CD300LF/TYROBP/PTX3/HCK/NCF2/FGR/AIF1 | 16 |  |
| BP | GO:0002764 | immune response-regulating signaling pathway | 22/155 | 500/18870 | 0.044 | 5.356645 | 8.985205 | 1.42E-10 | 1.95E-08 | 1.44E-08 | CLEC4D/TLR2/FCN1/C5AR1/NR4A3/FCER1G/TLR4/LILRA2/CD14/TNFAIP3/LILRB2/NLRP3/CD33/NFKBIZ/CD300LF/PELI1/TYROBP/HCK/NFKBIA/FGR/CARD8/MNDA | 22 |  |
| BP | GO:0032640 | tumor necrosis factor production | 14/155 | 186/18870 | 0.075269 | 9.163371 | 10.18208 | 4.31E-10 | 5.43E-08 | 3.99E-08 | ZFP36/TLR2/TLR4/THBS1/LILRA2/IFNGR1/CD14/TNFAIP3/PTAFR/LILRA5/CD33/TYROBP/CLEC4A/LRRK2 | 14 |  |
| BP | GO:0032680 | regulation of tumor necrosis factor production | 14/155 | 186/18870 | 0.075269 | 9.163371 | 10.18208 | 4.31E-10 | 5.43E-08 | 3.99E-08 | ZFP36/TLR2/TLR4/THBS1/LILRA2/IFNGR1/CD14/TNFAIP3/PTAFR/LILRA5/CD33/TYROBP/CLEC4A/LRRK2 | 14 |  |
| BP | GO:0097530 | granulocyte migration | 13/155 | 156/18870 | 0.083333 | 10.14516 | 10.43796 | 5.29E-10 | 6.26E-08 | 4.60E-08 | S100A12/BST1/IL1B/CCL20/TREM1/C5AR1/S100A9/CSF3R/FCER1G/THBS1/CCL4/S100A8/CXCL1 | 13 |  |
| BP | GO:0002768 | immune response-regulating cell surface receptor signaling pathway | 18/155 | 346/18870 | 0.052023 | 6.333395 | 9.112128 | 5.40E-10 | 6.26E-08 | 4.60E-08 | CLEC4D/TLR2/FCN1/C5AR1/NR4A3/FCER1G/TLR4/LILRA2/CD14/TNFAIP3/LILRB2/NFKBIZ/PELI1/TYROBP/HCK/NFKBIA/FGR/MNDA | 18 |  |
| BP | GO:0071706 | tumor necrosis factor superfamily cytokine production | 14/155 | 191/18870 | 0.073298 | 8.923493 | 10.01618 | 6.11E-10 | 6.56E-08 | 4.82E-08 | ZFP36/TLR2/TLR4/THBS1/LILRA2/IFNGR1/CD14/TNFAIP3/PTAFR/LILRA5/CD33/TYROBP/CLEC4A/LRRK2 | 14 |  |
| BP | GO:1903555 | regulation of tumor necrosis factor superfamily cytokine production | 14/155 | 191/18870 | 0.073298 | 8.923493 | 10.01618 | 6.11E-10 | 6.56E-08 | 4.82E-08 | ZFP36/TLR2/TLR4/THBS1/LILRA2/IFNGR1/CD14/TNFAIP3/PTAFR/LILRA5/CD33/TYROBP/CLEC4A/LRRK2 | 14 |  |
| BP | GO:0070661 | leukocyte proliferation | 18/155 | 350/18870 | 0.051429 | 6.261014 | 9.041247 | 6.50E-10 | 6.72E-08 | 4.94E-08 | CD55/BST1/IL1B/IRS2/TLR4/TNFAIP3/LILRB2/CDKN1A/NFKBIZ/CEBPB/PELI1/TYROBP/CRTAM/ST6GAL1/CSF2RB/CD1D/MNDA/AIF1 | 18 |  |
| BP | GO:1990266 | neutrophil migration | 12/155 | 129/18870 | 0.093023 | 11.32483 | 10.70845 | 7.03E-10 | 7.03E-08 | 5.16E-08 | S100A12/BST1/IL1B/CCL20/TREM1/C5AR1/S100A9/CSF3R/FCER1G/CCL4/S100A8/CXCL1 | 12 |  |
| BP | GO:0032944 | regulation of mononuclear cell proliferation | 15/155 | 242/18870 | 0.061983 | 7.545988 | 9.327068 | 1.50E-09 | 1.45E-07 | 1.06E-07 | CD55/BST1/IL1B/IRS2/TLR4/LILRB2/CDKN1A/CEBPB/PELI1/TYROBP/CRTAM/ST6GAL1/CD1D/MNDA/AIF1 | 15 |  |
| BP | GO:0007159 | leukocyte cell-cell adhesion | 19/155 | 419/18870 | 0.045346 | 5.520517 | 8.5159 | 1.72E-09 | 1.61E-07 | 1.18E-07 | CD55/IL1B/S100A9/NR4A3/LRG1/S100A8/VNN1/PTAFR/LILRB2/NLRP3/ICAM1/NFKBIZ/CEBPB/PELI1/CRTAM/CD83/CD1D/EGR3/AIF1 | 19 |  |
| BP | GO:0042742 | defense response to bacterium | 17/155 | 330/18870 | 0.051515 | 6.271554 | 8.791969 | 1.95E-09 | 1.77E-07 | 1.30E-07 | S100A12/CLEC4D/TLR2/IL1B/CCL20/TREM1/C5AR1/S100A9/FCER1G/TLR4/ANXA3/S100A8/MPEG1/NFKBIZ/CEBPB/FGR/LYZ | 17 |  |
| BP | GO:0050727 | regulation of inflammatory response | 19/155 | 425/18870 | 0.044706 | 5.442581 | 8.43016 | 2.18E-09 | 1.91E-07 | 1.40E-07 | S100A12/ZFP36/TLR2/BST1/IL1B/S100A9/MMP9/TLR4/IER3/S100A8/TNFAIP3/LILRA5/NLRP3/NFKBIZ/CEBPB/HCK/NFKBIA/FGR/LRRK2 | 19 |  |
| BP | GO:0045089 | positive regulation of innate immune response | 17/155 | 343/18870 | 0.049563 | 6.033857 | 8.562306 | 3.49E-09 | 2.97E-07 | 2.19E-07 | TLR2/FCN1/TLR4/LILRA2/CD14/TNFAIP3/NLRP3/NFKBIZ/CD300LF/PELI1/TYROBP/CRTAM/HCK/NFKBIA/CD1D/CARD8/MNDA | 17 |  |
| BP | GO:0050729 | positive regulation of inflammatory response | 12/155 | 152/18870 | 0.078947 | 9.611205 | 9.700663 | 4.65E-09 | 3.85E-07 | 2.83E-07 | S100A12/TLR2/IL1B/S100A9/TLR4/S100A8/LILRA5/NLRP3/NFKBIZ/CEBPB/NFKBIA/LRRK2 | 12 |  |
| BP | GO:0002544 | chronic inflammatory response | 6/155 | 19/18870 | 0.315789 | 38.44482 | 14.86096 | 6.92E-09 | 5.48E-07 | 4.03E-07 | S100A9/THBS1/S100A8/VNN1/TNFAIP3/NFKBIZ | 6 |  |
| BP | GO:0032943 | mononuclear cell proliferation | 16/155 | 314/18870 | 0.050955 | 6.203411 | 8.461671 | 7.00E-09 | 5.48E-07 | 4.03E-07 | CD55/BST1/IL1B/IRS2/TLR4/LILRB2/CDKN1A/NFKBIZ/CEBPB/PELI1/TYROBP/CRTAM/ST6GAL1/CD1D/MNDA/AIF1 | 16 |  |
| BP | GO:0002429 | immune response-activating cell surface receptor signaling pathway | 16/155 | 318/18870 | 0.050314 | 6.12538 | 8.388604 | 8.37E-09 | 6.38E-07 | 4.69E-07 | TLR2/FCN1/C5AR1/NR4A3/FCER1G/TLR4/LILRA2/CD14/TNFAIP3/NFKBIZ/PELI1/TYROBP/HCK/NFKBIA/FGR/MNDA | 16 |  |
| BP | GO:0002218 | activation of innate immune response | 15/155 | 276/18870 | 0.054348 | 6.61641 | 8.55406 | 8.95E-09 | 6.65E-07 | 4.89E-07 | TLR2/FCN1/TLR4/LILRA2/CD14/TNFAIP3/NLRP3/NFKBIZ/CD300LF/PELI1/TYROBP/HCK/NFKBIA/CARD8/MNDA | 15 |  |
| BP | GO:0002833 | positive regulation of response to biotic stimulus | 17/155 | 369/18870 | 0.04607 | 5.608707 | 8.136546 | 1.04E-08 | 7.45E-07 | 5.48E-07 | TLR2/FCN1/TLR4/LILRA2/CD14/TNFAIP3/NLRP3/NFKBIZ/CD300LF/PELI1/TYROBP/CRTAM/HCK/NFKBIA/CD1D/CARD8/MNDA | 17 |  |
| BP | GO:0050670 | regulation of lymphocyte proliferation | 14/155 | 238/18870 | 0.058824 | 7.16129 | 8.705141 | 1.05E-08 | 7.45E-07 | 5.48E-07 | CD55/BST1/IL1B/IRS2/TLR4/LILRB2/CDKN1A/CEBPB/PELI1/TYROBP/CRTAM/CD1D/MNDA/AIF1 | 14 |  |
| BP | GO:0002757 | immune response-activating signaling pathway | 19/155 | 473/18870 | 0.040169 | 4.890268 | 7.797977 | 1.24E-08 | 8.50E-07 | 6.25E-07 | TLR2/FCN1/C5AR1/NR4A3/FCER1G/TLR4/LILRA2/CD14/TNFAIP3/NLRP3/NFKBIZ/CD300LF/PELI1/TYROBP/HCK/NFKBIA/FGR/CARD8/MNDA | 19 |  |
| BP | GO:0070665 | positive regulation of leukocyte proliferation | 12/155 | 166/18870 | 0.072289 | 8.800622 | 9.186739 | 1.26E-08 | 8.50E-07 | 6.25E-07 | CD55/BST1/IL1B/IRS2/TLR4/LILRB2/CDKN1A/PELI1/ST6GAL1/CSF2RB/CD1D/AIF1 | 12 |  |
| BP | GO:0097529 | myeloid leukocyte migration | 14/155 | 242/18870 | 0.057851 | 7.042922 | 8.610273 | 1.30E-08 | 8.58E-07 | 6.31E-07 | S100A12/BST1/IL1B/CCL20/TREM1/C5AR1/S100A9/CSF3R/FCER1G/THBS1/CCL4/S100A8/CXCL1/AIF1 | 14 |  |
| BP | GO:0045088 | regulation of innate immune response | 18/155 | 425/18870 | 0.042353 | 5.156129 | 7.886595 | 1.37E-08 | 8.84E-07 | 6.50E-07 | TLR2/FCN1/TLR4/LILRA2/CD14/TNFAIP3/NLRP3/NFKBIZ/CD300LF/PELI1/TYROBP/CRTAM/HCK/NFKBIA/CD1D/FGR/CARD8/MNDA | 18 |  |
| BP | GO:0051251 | positive regulation of lymphocyte activation | 16/155 | 330/18870 | 0.048485 | 5.902639 | 8.176688 | 1.41E-08 | 8.89E-07 | 6.54E-07 | CD55/BST1/IL1B/IRS2/TLR4/VNN1/LILRB2/NLRP3/CDKN1A/NFKBIZ/PELI1/TYROBP/CD83/CD1D/EGR3/AIF1 | 16 |  |
| BP | GO:0032760 | positive regulation of tumor necrosis factor production | 10/155 | 106/18870 | 0.09434 | 11.48509 | 9.851621 | 1.69E-08 | 1.04E-06 | 7.67E-07 | TLR2/TLR4/THBS1/LILRA2/IFNGR1/CD14/PTAFR/LILRA5/TYROBP/LRRK2 | 10 |  |
| BP | GO:1903557 | positive regulation of tumor necrosis factor superfamily cytokine production | 10/155 | 110/18870 | 0.090909 | 11.06745 | 9.637064 | 2.42E-08 | 1.46E-06 | 1.08E-06 | TLR2/TLR4/THBS1/LILRA2/IFNGR1/CD14/PTAFR/LILRA5/TYROBP/LRRK2 | 10 |  |
| BP | GO:0002758 | innate immune response-activating signaling pathway | 14/155 | 255/18870 | 0.054902 | 6.683871 | 8.316262 | 2.52E-08 | 1.49E-06 | 1.10E-06 | TLR2/FCN1/TLR4/LILRA2/CD14/TNFAIP3/NLRP3/NFKBIZ/CD300LF/PELI1/TYROBP/HCK/NFKBIA/CARD8 | 14 |  |
| BP | GO:0002366 | leukocyte activation involved in immune response | 15/155 | 301/18870 | 0.049834 | 6.066874 | 8.064445 | 2.85E-08 | 1.65E-06 | 1.21E-06 | CLEC4D/DYSF/NR4A3/FCER1G/TLR4/ANXA3/LILRA2/EOMES/PTAFR/RORC/NLRP3/ICAM1/NFKBIZ/TYROBP/FGR | 15 |  |
| BP | GO:0002263 | cell activation involved in immune response | 15/155 | 305/18870 | 0.04918 | 5.987308 | 7.991237 | 3.39E-08 | 1.93E-06 | 1.42E-06 | CLEC4D/DYSF/NR4A3/FCER1G/TLR4/ANXA3/LILRA2/EOMES/PTAFR/RORC/NLRP3/ICAM1/NFKBIZ/TYROBP/FGR | 15 |  |
| BP | GO:0046651 | lymphocyte proliferation | 15/155 | 307/18870 | 0.04886 | 5.948303 | 7.955121 | 3.70E-08 | 2.06E-06 | 1.52E-06 | CD55/BST1/IL1B/IRS2/TLR4/LILRB2/CDKN1A/NFKBIZ/CEBPB/PELI1/TYROBP/CRTAM/CD1D/MNDA/AIF1 | 15 |  |
| BP | GO:0032946 | positive regulation of mononuclear cell proliferation | 11/155 | 148/18870 | 0.074324 | 9.048387 | 8.94559 | 3.91E-08 | 2.14E-06 | 1.57E-06 | CD55/BST1/IL1B/IRS2/TLR4/LILRB2/CDKN1A/PELI1/ST6GAL1/CD1D/AIF1 | 11 |  |
| BP | GO:0002220 | innate immune response activating cell surface receptor signaling pathway | 9/155 | 91/18870 | 0.098901 | 12.04041 | 9.607604 | 5.95E-08 | 3.19E-06 | 2.35E-06 | TLR2/FCN1/TLR4/LILRA2/CD14/TNFAIP3/PELI1/TYROBP/NFKBIA | 9 |  |
| BP | GO:0051384 | response to glucocorticoid | 10/155 | 127/18870 | 0.07874 | 9.585979 | 8.835223 | 9.60E-08 | 5.06E-06 | 3.72E-06 | ZFP36/FOS/ADAM9/ANXA3/PTAFR/FOSB/AREG/ADM/CDKN1A/CYP1B1 | 10 |  |
| BP | GO:0002752 | cell surface pattern recognition receptor signaling pathway | 8/155 | 70/18870 | 0.114286 | 13.91336 | 9.850423 | 1.06E-07 | 5.49E-06 | 4.03E-06 | TLR2/FCN1/TLR4/LILRA2/CD14/TNFAIP3/PELI1/NFKBIA | 8 |  |
| BP | GO:0032612 | interleukin-1 production | 10/155 | 130/18870 | 0.076923 | 9.364764 | 8.709354 | 1.20E-07 | 5.98E-06 | 4.40E-06 | IL1R2/TLR4/LILRA2/TNFAIP3/LILRA5/NLRP3/CD33/TYROBP/EGR1/CARD8 | 10 |  |
| BP | GO:0032652 | regulation of interleukin-1 production | 10/155 | 130/18870 | 0.076923 | 9.364764 | 8.709354 | 1.20E-07 | 5.98E-06 | 4.40E-06 | IL1R2/TLR4/LILRA2/TNFAIP3/LILRA5/NLRP3/CD33/TYROBP/EGR1/CARD8 | 10 |  |
| BP | GO:0050764 | regulation of phagocytosis | 9/155 | 100/18870 | 0.09 | 10.95677 | 9.085146 | 1.36E-07 | 6.66E-06 | 4.90E-06 | TLR2/FCN1/IL1B/DYSF/FCER1G/CD300LF/PTX3/HCK/FGR | 9 |  |
| BP | GO:0032755 | positive regulation of interleukin-6 production | 9/155 | 101/18870 | 0.089109 | 10.84829 | 9.031219 | 1.48E-07 | 7.14E-06 | 5.25E-06 | TLR2/IL1B/TLR4/LILRA2/PTAFR/LILRA5/LILRB2/TYROBP/AIF1 | 9 |  |
| BP | GO:2001233 | regulation of apoptotic signaling pathway | 16/155 | 398/18870 | 0.040201 | 4.894148 | 7.145661 | 1.87E-07 | 8.89E-06 | 6.53E-06 | NR4A2/IL1B/S100A9/MMP9/TLR4/THBS1/IER3/PMAIP1/DDIT3/S100A8/VNN1/TNFAIP3/ICAM1/PLAUR/G0S2/LRRK2 | 16 |  |
| BP | GO:0048659 | smooth muscle cell proliferation | 11/155 | 175/18870 | 0.062857 | 7.65235 | 8.04594 | 2.17E-07 | 1.01E-05 | 7.45E-06 | MMP9/NR4A3/TLR4/THBS1/HBEGF/DDIT3/TNFAIP3/PTAFR/CDKN1A/AIF1/GJA1 | 11 |  |
| BP | GO:0034612 | response to tumor necrosis factor | 13/155 | 259/18870 | 0.050193 | 6.110599 | 7.536717 | 2.30E-07 | 1.06E-05 | 7.77E-06 | ZFP36/CCL20/FOS/CXCL16/THBS1/ADAM9/CCL4/CD14/TNFAIP3/CYP1B1/NFKBIA/LRRK2/CARD8 | 13 |  |
| BP | GO:0032635 | interleukin-6 production | 11/155 | 177/18870 | 0.062147 | 7.565883 | 7.987036 | 2.43E-07 | 1.07E-05 | 7.90E-06 | TLR2/IL1B/TLR4/LILRA2/TNFAIP3/PTAFR/LILRA5/LILRB2/CEBPB/TYROBP/AIF1 | 11 |  |
| BP | GO:0032675 | regulation of interleukin-6 production | 11/155 | 177/18870 | 0.062147 | 7.565883 | 7.987036 | 2.43E-07 | 1.07E-05 | 7.90E-06 | TLR2/IL1B/TLR4/LILRA2/TNFAIP3/PTAFR/LILRA5/LILRB2/CEBPB/TYROBP/AIF1 | 11 |  |
| BP | GO:0051090 | regulation of DNA-binding transcription factor activity | 16/155 | 406/18870 | 0.039409 | 4.797712 | 7.039916 | 2.45E-07 | 1.07E-05 | 7.90E-06 | S100A12/TLR2/IL1B/S100A9/TLR4/ANXA3/EOMES/DDIT3/S100A8/TNFAIP3/NLRP3/HCK/CYP1B1/NFKBIA/LRRK2/CARD8 | 16 |  |
| BP | GO:0030217 | T cell differentiation | 14/155 | 307/18870 | 0.045603 | 5.55175 | 7.317603 | 2.51E-07 | 1.08E-05 | 7.96E-06 | CLEC4D/IL1B/FCER1G/EOMES/VNN1/RORC/LILRB2/NLRP3/NFKBIZ/CRTAM/CD83/CD1D/EGR3/EGR1 | 14 |  |
| BP | GO:0042116 | macrophage activation | 9/155 | 110/18870 | 0.081818 | 9.960704 | 8.577632 | 3.09E-07 | 1.32E-05 | 9.67E-06 | TLR2/C5AR1/DYSF/TLR4/THBS1/IFNGR1/TYROBP/LRRK2/AIF1 | 9 |  |
| BP | GO:0050671 | positive regulation of lymphocyte proliferation | 10/155 | 145/18870 | 0.068966 | 8.395996 | 8.136078 | 3.34E-07 | 1.36E-05 | 1.00E-05 | CD55/BST1/IL1B/IRS2/TLR4/LILRB2/CDKN1A/PELI1/CD1D/AIF1 | 10 |  |
| BP | GO:0032611 | interleukin-1 beta production | 9/155 | 111/18870 | 0.081081 | 9.870968 | 8.530471 | 3.34E-07 | 1.36E-05 | 1.00E-05 | TLR4/LILRA2/TNFAIP3/LILRA5/NLRP3/CD33/TYROBP/EGR1/CARD8 | 9 |  |
| BP | GO:0032651 | regulation of interleukin-1 beta production | 9/155 | 111/18870 | 0.081081 | 9.870968 | 8.530471 | 3.34E-07 | 1.36E-05 | 1.00E-05 | TLR4/LILRA2/TNFAIP3/LILRA5/NLRP3/CD33/TYROBP/EGR1/CARD8 | 9 |  |
| BP | GO:0150076 | neuroinflammatory response | 8/155 | 82/18870 | 0.097561 | 11.87726 | 8.983211 | 3.68E-07 | 1.47E-05 | 1.08E-05 | TLR2/IL1B/C5AR1/MMP9/IFNGR1/TYROBP/LRRK2/AIF1 | 8 |  |
| BP | GO:0002283 | neutrophil activation involved in immune response | 5/155 | 19/18870 | 0.263158 | 32.03735 | 12.31798 | 3.71E-07 | 1.47E-05 | 1.08E-05 | FCER1G/ANXA3/LILRA2/PTAFR/TYROBP | 5 |  |
| BP | GO:0097193 | intrinsic apoptotic signaling pathway | 14/155 | 319/18870 | 0.043887 | 5.342906 | 7.119304 | 3.98E-07 | 1.56E-05 | 1.15E-05 | PPP1R15A/S100A9/MMP9/IER3/PMAIP1/DDIT3/S100A8/VNN1/BCL2A1/CDKN1A/PLAUR/CEBPB/CYP1B1/LRRK2 | 14 |  |
| BP | GO:0061900 | glial cell activation | 7/155 | 57/18870 | 0.122807 | 14.95076 | 9.59957 | 4.13E-07 | 1.59E-05 | 1.17E-05 | TLR2/IL1B/C5AR1/IFNGR1/TYROBP/LRRK2/AIF1 | 7 |  |
| BP | GO:0031960 | response to corticosteroid | 10/155 | 149/18870 | 0.067114 | 8.1706 | 7.997044 | 4.29E-07 | 1.64E-05 | 1.20E-05 | ZFP36/FOS/ADAM9/ANXA3/PTAFR/FOSB/AREG/ADM/CDKN1A/CYP1B1 | 10 |  |
| BP | GO:1903131 | mononuclear cell differentiation | 17/155 | 481/18870 | 0.035343 | 4.30273 | 6.677469 | 4.68E-07 | 1.76E-05 | 1.29E-05 | NFIL3/CLEC4D/IL1B/FOS/FCER1G/EOMES/VNN1/RORC/LILRB2/NLRP3/NFKBIZ/CEBPB/CRTAM/CD83/CD1D/EGR3/EGR1 | 17 |  |
| BP | GO:0002221 | pattern recognition receptor signaling pathway | 12/155 | 236/18870 | 0.050847 | 6.190268 | 7.301937 | 5.94E-07 | 2.20E-05 | 1.61E-05 | TLR2/FCN1/TLR4/LILRA2/CD14/TNFAIP3/NLRP3/NFKBIZ/CD300LF/PELI1/NFKBIA/CARD8 | 12 |  |
| BP | GO:0048545 | response to steroid hormone | 14/155 | 330/18870 | 0.042424 | 5.164809 | 6.946125 | 5.99E-07 | 2.20E-05 | 1.61E-05 | ZFP36/TLR2/FOS/NR4A3/THBS1/ADAM9/ANXA3/PTAFR/FOSB/AREG/ADM/CDKN1A/CYP1B1/CNOT1 | 14 |  |
| BP | GO:1903037 | regulation of leukocyte cell-cell adhesion | 15/155 | 382/18870 | 0.039267 | 4.780442 | 6.793205 | 6.21E-07 | 2.25E-05 | 1.65E-05 | CD55/IL1B/NR4A3/VNN1/PTAFR/LILRB2/NLRP3/NFKBIZ/CEBPB/PELI1/CRTAM/CD83/CD1D/EGR3/AIF1 | 15 |  |
| BP | GO:0032731 | positive regulation of interleukin-1 beta production | 7/155 | 62/18870 | 0.112903 | 13.74506 | 9.147696 | 7.42E-07 | 2.65E-05 | 1.95E-05 | TLR4/LILRA2/LILRA5/NLRP3/TYROBP/EGR1/CARD8 | 7 |  |
| BP | GO:0032757 | positive regulation of interleukin-8 production | 7/155 | 63/18870 | 0.111111 | 13.52688 | 9.063561 | 8.29E-07 | 2.89E-05 | 2.13E-05 | TLR2/FCN1/IL1B/TLR4/LILRA2/DDIT3/CD14 | 7 |  |
| BP | GO:0140895 | cell surface toll-like receptor signaling pathway | 7/155 | 63/18870 | 0.111111 | 13.52688 | 9.063561 | 8.29E-07 | 2.89E-05 | 2.13E-05 | TLR2/TLR4/LILRA2/CD14/TNFAIP3/PELI1/NFKBIA | 7 |  |
| BP | GO:0033002 | muscle cell proliferation | 12/155 | 247/18870 | 0.048583 | 5.914588 | 7.075484 | 9.63E-07 | 3.32E-05 | 2.44E-05 | FOS/MMP9/NR4A3/TLR4/THBS1/HBEGF/DDIT3/TNFAIP3/PTAFR/CDKN1A/AIF1/GJA1 | 12 |  |
| BP | GO:0030888 | regulation of B cell proliferation | 7/155 | 65/18870 | 0.107692 | 13.11067 | 8.900892 | 1.03E-06 | 3.51E-05 | 2.58E-05 | BST1/IRS2/TLR4/CDKN1A/PELI1/TYROBP/MNDA | 7 |  |
| BP | GO:0071674 | mononuclear cell migration | 11/155 | 207/18870 | 0.05314 | 6.469378 | 7.200751 | 1.16E-06 | 3.90E-05 | 2.86E-05 | S100A12/CCL20/C5AR1/CXCL16/THBS1/CCL4/CH25H/ICAM1/CRTAM/CCR5/AIF1 | 11 |  |
| BP | GO:0009620 | response to fungus | 7/155 | 67/18870 | 0.104478 | 12.71931 | 8.745228 | 1.27E-06 | 4.22E-05 | 3.10E-05 | S100A12/CLEC4D/S100A9/TLR4/S100A8/PTX3/CLEC4A | 7 |  |
| BP | GO:0014002 | astrocyte development | 6/155 | 43/18870 | 0.139535 | 16.98725 | 9.551305 | 1.32E-06 | 4.35E-05 | 3.20E-05 | IL1B/C5AR1/S100A9/TLR4/IFNGR1/S100A8 | 6 |  |
| BP | GO:0002275 | myeloid cell activation involved in immune response | 8/155 | 98/18870 | 0.081633 | 9.938117 | 8.073265 | 1.46E-06 | 4.69E-05 | 3.44E-05 | DYSF/NR4A3/FCER1G/ANXA3/LILRA2/PTAFR/TYROBP/FGR | 8 |  |
| BP | GO:0048661 | positive regulation of smooth muscle cell proliferation | 8/155 | 98/18870 | 0.081633 | 9.938117 | 8.073265 | 1.46E-06 | 4.69E-05 | 3.44E-05 | MMP9/NR4A3/TLR4/THBS1/HBEGF/PTAFR/AIF1/GJA1 | 8 |  |
| BP | GO:0002443 | leukocyte mediated immunity | 16/155 | 466/18870 | 0.034335 | 4.179981 | 6.325672 | 1.52E-06 | 4.79E-05 | 3.52E-05 | CD55/IL1B/TREM1/NR4A3/FCER1G/TLR4/ANXA3/PTAFR/NLRP3/ICAM1/NFKBIZ/TYROBP/CRTAM/CSF2RB/CD1D/FGR | 16 |  |
| BP | GO:0048660 | regulation of smooth muscle cell proliferation | 10/155 | 171/18870 | 0.05848 | 7.119411 | 7.315498 | 1.52E-06 | 4.79E-05 | 3.52E-05 | MMP9/NR4A3/TLR4/THBS1/HBEGF/TNFAIP3/PTAFR/CDKN1A/AIF1/GJA1 | 10 |  |
| BP | GO:1903706 | regulation of hemopoiesis | 15/155 | 417/18870 | 0.035971 | 4.379206 | 6.350306 | 1.85E-06 | 5.77E-05 | 4.24E-05 | ZFP36/FOS/CSF3R/TLR4/VNN1/LILRB2/NLRP3/NFKBIZ/CEBPB/TYROBP/CRTAM/TOB2/CD83/NFKBIA/EGR3 | 15 |  |
| BP | GO:0051346 | negative regulation of hydrolase activity | 11/155 | 218/18870 | 0.050459 | 6.142942 | 6.950604 | 1.92E-06 | 5.91E-05 | 4.35E-05 | PPP1R15A/CSTA/MMP9/THBS1/GZMA/PLAUR/PTX3/RGS2/LRRK2/CARD8/SERPINB2 | 11 |  |
| BP | GO:0032677 | regulation of interleukin-8 production | 8/155 | 103/18870 | 0.07767 | 9.455684 | 7.830966 | 2.12E-06 | 6.48E-05 | 4.76E-05 | TLR2/FCN1/IL1B/TLR4/LILRA2/DDIT3/CD14/CD33 | 8 |  |
| BP | GO:0043086 | negative regulation of catalytic activity | 16/155 | 480/18870 | 0.033333 | 4.058065 | 6.176206 | 2.22E-06 | 6.69E-05 | 4.92E-05 | PPP1R15A/ZFP36/CSTA/GADD45A/IL1B/IRS2/MMP9/THBS1/CDKN1A/GZMA/PLAUR/PTX3/RGS2/LRRK2/CARD8/SERPINB2 | 16 |  |
| BP | GO:0032732 | positive regulation of interleukin-1 production | 7/155 | 73/18870 | 0.09589 | 11.67388 | 8.315436 | 2.28E-06 | 6.69E-05 | 4.92E-05 | TLR4/LILRA2/LILRA5/NLRP3/TYROBP/EGR1/CARD8 | 7 |  |
| BP | GO:0032637 | interleukin-8 production | 8/155 | 104/18870 | 0.076923 | 9.364764 | 7.784485 | 2.29E-06 | 6.69E-05 | 4.92E-05 | TLR2/FCN1/IL1B/TLR4/LILRA2/DDIT3/CD14/CD33 | 8 |  |
| BP | GO:0042100 | B cell proliferation | 8/155 | 104/18870 | 0.076923 | 9.364764 | 7.784485 | 2.29E-06 | 6.69E-05 | 4.92E-05 | BST1/IRS2/TLR4/CDKN1A/NFKBIZ/PELI1/TYROBP/MNDA | 8 |  |
| BP | GO:0050866 | negative regulation of cell activation | 11/155 | 223/18870 | 0.049327 | 6.005208 | 6.84251 | 2.39E-06 | 6.93E-05 | 5.09E-05 | THBD/TNFAIP3/LILRB2/CD33/CD300LF/CEBPB/PELI1/TYROBP/CRTAM/FGR/MNDA | 11 |  |
| BP | GO:0002699 | positive regulation of immune effector process | 12/155 | 270/18870 | 0.044444 | 5.410753 | 6.643294 | 2.44E-06 | 6.99E-05 | 5.14E-05 | CD55/FCN1/IL1B/NR4A3/TLR4/PTAFR/NLRP3/NFKBIZ/TYROBP/CRTAM/CD1D/FGR | 12 |  |
| BP | GO:0010631 | epithelial cell migration | 14/155 | 372/18870 | 0.037634 | 4.581686 | 6.349535 | 2.46E-06 | 6.99E-05 | 5.14E-05 | S100A12/GADD45A/IRS2/S100A9/S100P/MMP9/THBS1/ADAM9/LRG1/ANXA3/HBEGF/CYP1B1/GLUL/EGR3 | 14 |  |
| BP | GO:0030098 | lymphocyte differentiation | 15/155 | 429/18870 | 0.034965 | 4.256711 | 6.209563 | 2.62E-06 | 7.38E-05 | 5.43E-05 | NFIL3/CLEC4D/IL1B/FCER1G/EOMES/VNN1/RORC/LILRB2/NLRP3/NFKBIZ/CRTAM/CD83/CD1D/EGR3/EGR1 | 15 |  |
| BP | GO:0022409 | positive regulation of cell-cell adhesion | 13/155 | 322/18870 | 0.040373 | 4.915047 | 6.448545 | 2.66E-06 | 7.41E-05 | 5.44E-05 | CD55/IL1B/NR4A3/VNN1/PTAFR/LILRB2/NLRP3/NFKBIZ/PLAUR/CD83/CD1D/EGR3/AIF1 | 13 |  |
| BP | GO:0090132 | epithelium migration | 14/155 | 375/18870 | 0.037333 | 4.545032 | 6.310359 | 2.70E-06 | 7.45E-05 | 5.48E-05 | S100A12/GADD45A/IRS2/S100A9/S100P/MMP9/THBS1/ADAM9/LRG1/ANXA3/HBEGF/CYP1B1/GLUL/EGR3 | 14 |  |
| BP | GO:0001774 | microglial cell activation | 6/155 | 49/18870 | 0.122449 | 14.90718 | 8.870764 | 2.91E-06 | 7.86E-05 | 5.78E-05 | TLR2/C5AR1/IFNGR1/TYROBP/LRRK2/AIF1 | 6 |  |
| BP | GO:1903039 | positive regulation of leukocyte cell-cell adhesion | 12/155 | 275/18870 | 0.043636 | 5.312375 | 6.555868 | 2.95E-06 | 7.86E-05 | 5.78E-05 | CD55/IL1B/NR4A3/VNN1/PTAFR/LILRB2/NLRP3/NFKBIZ/CD83/CD1D/EGR3/AIF1 | 12 |  |
| BP | GO:0070486 | leukocyte aggregation | 4/155 | 13/18870 | 0.307692 | 37.45906 | 11.96702 | 2.96E-06 | 7.86E-05 | 5.78E-05 | IL1B/S100A9/NR4A3/S100A8 | 4 |  |
| BP | GO:0031664 | regulation of lipopolysaccharide-mediated signaling pathway | 5/155 | 28/18870 | 0.178571 | 21.73963 | 9.99452 | 2.96E-06 | 7.86E-05 | 5.78E-05 | CD55/LILRA2/CD14/TNFAIP3/CARD8 | 5 |  |
| BP | GO:0022407 | regulation of cell-cell adhesion | 16/155 | 493/18870 | 0.032454 | 3.951057 | 6.042395 | 3.13E-06 | 8.22E-05 | 6.04E-05 | CD55/IL1B/NR4A3/VNN1/PTAFR/LILRB2/NLRP3/NFKBIZ/PLAUR/CEBPB/PELI1/CRTAM/CD83/CD1D/EGR3/AIF1 | 16 |  |
| BP | GO:0090130 | tissue migration | 14/155 | 380/18870 | 0.036842 | 4.485229 | 6.245972 | 3.15E-06 | 8.22E-05 | 6.04E-05 | S100A12/GADD45A/IRS2/S100A9/S100P/MMP9/THBS1/ADAM9/LRG1/ANXA3/HBEGF/CYP1B1/GLUL/EGR3 | 14 |  |
| BP | GO:0001818 | negative regulation of cytokine production | 14/155 | 381/18870 | 0.036745 | 4.473457 | 6.233229 | 3.25E-06 | 8.40E-05 | 6.17E-05 | IL1R2/ZFP36/SRGN/TLR4/THBS1/DDIT3/TNFAIP3/LILRA5/NLRP3/CD33/TYROBP/CLEC4A/CD83/CARD8 | 14 |  |
| BP | GO:2000377 | regulation of reactive oxygen species metabolic process | 9/155 | 147/18870 | 0.061224 | 7.453588 | 7.148543 | 3.54E-06 | 9.07E-05 | 6.66E-05 | BST1/GADD45A/TLR4/THBS1/IER3/CDKN1A/TYROBP/CYP1B1/LRRK2 | 9 |  |
| BP | GO:0072593 | reactive oxygen species metabolic process | 11/155 | 234/18870 | 0.047009 | 5.722911 | 6.615867 | 3.81E-06 | 9.67E-05 | 7.11E-05 | BST1/GADD45A/TLR4/THBS1/IER3/PMAIP1/CDKN1A/TYROBP/CYP1B1/NCF2/LRRK2 | 11 |  |
| BP | GO:0002269 | leukocyte activation involved in inflammatory response | 6/155 | 52/18870 | 0.115385 | 14.04715 | 8.573849 | 4.15E-06 | 0.000104 | 7.63E-05 | TLR2/C5AR1/IFNGR1/TYROBP/LRRK2/AIF1 | 6 |  |
| BP | GO:0006968 | cellular defense response | 6/155 | 52/18870 | 0.115385 | 14.04715 | 8.573849 | 4.15E-06 | 0.000104 | 7.63E-05 | C5AR1/LILRB2/TYROBP/CCR5/NCF2/MNDA | 6 |  |
| BP | GO:0010575 | positive regulation of vascular endothelial growth factor production | 5/155 | 30/18870 | 0.166667 | 20.29032 | 9.622879 | 4.23E-06 | 0.000105 | 7.70E-05 | IL1B/C5AR1/SULF2/CYP1B1/SULF1 | 5 |  |
| BP | GO:0034142 | toll-like receptor 4 signaling pathway | 6/155 | 55/18870 | 0.109091 | 13.28094 | 8.300536 | 5.80E-06 | 0.000142 | 0.000105 | TLR4/LILRA2/CD14/TNFAIP3/PELI1/NFKBIA | 6 |  |
| BP | GO:0051591 | response to cAMP | 7/155 | 85/18870 | 0.082353 | 10.02581 | 7.589886 | 6.36E-06 | 0.000155 | 0.000114 | AQP9/FOS/THBD/PTAFR/FOSB/AREG/CYP1B1 | 7 |  |
| BP | GO:0002695 | negative regulation of leukocyte activation | 10/155 | 202/18870 | 0.049505 | 6.026828 | 6.536816 | 6.75E-06 | 0.000163 | 0.00012 | TNFAIP3/LILRB2/CD33/CD300LF/CEBPB/PELI1/TYROBP/CRTAM/FGR/MNDA | 10 |  |
| BP | GO:0032088 | negative regulation of NF-kappaB transcription factor activity | 7/155 | 87/18870 | 0.08046 | 9.795328 | 7.48298 | 7.42E-06 | 0.000176 | 0.00013 | DDIT3/TNFAIP3/NLRP3/CYP1B1/NFKBIA/LRRK2/CARD8 | 7 |  |
| BP | GO:0048708 | astrocyte differentiation | 7/155 | 87/18870 | 0.08046 | 9.795328 | 7.48298 | 7.42E-06 | 0.000176 | 0.00013 | IL1B/C5AR1/S100A9/TLR4/IFNGR1/EOMES/S100A8 | 7 |  |
| BP | GO:0043406 | positive regulation of MAP kinase activity | 7/155 | 88/18870 | 0.079545 | 9.684018 | 7.430816 | 8.01E-06 | 0.000189 | 0.000139 | S100A12/IL1B/TLR4/THBS1/ADAM9/PDGFC/LRRK2 | 7 |  |
| BP | GO:0032633 | interleukin-4 production | 5/155 | 35/18870 | 0.142857 | 17.39171 | 8.833254 | 9.33E-06 | 0.000216 | 0.000159 | FCER1G/DDIT3/NLRP3/CEBPB/CD83 | 5 |  |
| BP | GO:0032673 | regulation of interleukin-4 production | 5/155 | 35/18870 | 0.142857 | 17.39171 | 8.833254 | 9.33E-06 | 0.000216 | 0.000159 | FCER1G/DDIT3/NLRP3/CEBPB/CD83 | 5 |  |
| BP | GO:0006959 | humoral immune response | 11/155 | 258/18870 | 0.042636 | 5.190548 | 6.167789 | 9.63E-06 | 0.000221 | 0.000163 | CD55/S100A12/BST1/FCN1/IL1B/TREM1/S100A9/CXCL1/ST6GAL1/CD83/LYZ | 11 |  |
| BP | GO:0046683 | response to organophosphorus | 8/155 | 127/18870 | 0.062992 | 7.668783 | 6.862373 | 1.01E-05 | 0.000231 | 0.00017 | IL1B/AQP9/FOS/THBD/PTAFR/FOSB/AREG/CYP1B1 | 8 |  |
| BP | GO:0050864 | regulation of B cell activation | 8/155 | 130/18870 | 0.061538 | 7.491811 | 6.759244 | 1.20E-05 | 0.000273 | 0.0002 | BST1/IRS2/TLR4/TNFAIP3/CDKN1A/PELI1/TYROBP/MNDA | 8 |  |
| BP | GO:0031100 | animal organ regeneration | 6/155 | 64/18870 | 0.09375 | 11.41331 | 7.594091 | 1.41E-05 | 0.000315 | 0.000231 | NR4A3/SULF2/ANXA3/ADM/CDKN1A/CEBPB | 6 |  |
| BP | GO:1902105 | regulation of leukocyte differentiation | 12/155 | 321/18870 | 0.037383 | 4.5511 | 5.839835 | 1.42E-05 | 0.000315 | 0.000231 | FOS/TLR4/VNN1/LILRB2/NLRP3/NFKBIZ/CEBPB/TYROBP/CRTAM/TOB2/CD83/EGR3 | 12 |  |
| BP | GO:0070664 | negative regulation of leukocyte proliferation | 7/155 | 96/18870 | 0.072917 | 8.877016 | 7.041486 | 1.42E-05 | 0.000315 | 0.000231 | TNFAIP3/LILRB2/CEBPB/PELI1/TYROBP/CRTAM/MNDA | 7 |  |
| BP | GO:0001667 | ameboidal-type cell migration | 15/155 | 497/18870 | 0.030181 | 3.674304 | 5.498501 | 1.53E-05 | 0.000336 | 0.000247 | S100A12/GADD45A/IRS2/S100A9/S100P/MMP9/THBS1/ADAM9/LRG1/ANXA3/HBEGF/CYP1B1/GLUL/EGR3/GJA1 | 15 |  |
| BP | GO:0050863 | regulation of T cell activation | 13/155 | 381/18870 | 0.034121 | 4.153924 | 5.659817 | 1.63E-05 | 0.000354 | 0.00026 | CD55/IL1B/VNN1/LILRB2/NLRP3/NFKBIZ/CEBPB/PELI1/CRTAM/CD83/CD1D/EGR3/AIF1 | 13 |  |
| BP | GO:0009595 | detection of biotic stimulus | 5/155 | 40/18870 | 0.125 | 15.21774 | 8.191828 | 1.83E-05 | 0.000392 | 0.000288 | TLR2/TLR4/NLRP3/CRTAM/CD1D | 5 |  |
| BP | GO:0032570 | response to progesterone | 5/155 | 40/18870 | 0.125 | 15.21774 | 8.191828 | 1.83E-05 | 0.000392 | 0.000288 | TLR2/FOS/THBS1/FOSB/CYP1B1 | 5 |  |
| BP | GO:0051546 | keratinocyte migration | 4/155 | 20/18870 | 0.2 | 24.34839 | 9.507396 | 1.92E-05 | 0.000408 | 0.0003 | MMP9/ADAM9/LRG1/HBEGF | 4 |  |
| BP | GO:2000379 | positive regulation of reactive oxygen species metabolic process | 6/155 | 68/18870 | 0.088235 | 10.74194 | 7.323911 | 2.00E-05 | 0.000424 | 0.000312 | GADD45A/TLR4/THBS1/CDKN1A/TYROBP/CYP1B1 | 6 |  |
| BP | GO:0002697 | regulation of immune effector process | 13/155 | 389/18870 | 0.033419 | 4.068497 | 5.56523 | 2.02E-05 | 0.000424 | 0.000312 | CD55/FCN1/IL1B/NR4A3/TLR4/PTAFR/NLRP3/ICAM1/NFKBIZ/TYROBP/CRTAM/CD1D/FGR | 13 |  |
| BP | GO:0001659 | temperature homeostasis | 9/155 | 183/18870 | 0.04918 | 5.987308 | 6.169747 | 2.08E-05 | 0.000433 | 0.000318 | ACSL1/IL1B/TLR4/DDIT3/CEBPB/G0S2/PDGFC/EGR1/GJA1 | 9 |  |
| BP | GO:0043405 | regulation of MAP kinase activity | 8/155 | 142/18870 | 0.056338 | 6.858701 | 6.377422 | 2.29E-05 | 0.000471 | 0.000346 | S100A12/IL1B/TLR4/THBS1/ADAM9/RGS2/PDGFC/LRRK2 | 8 |  |
| BP | GO:0062012 | regulation of small molecule metabolic process | 12/155 | 337/18870 | 0.035608 | 4.335024 | 5.621944 | 2.29E-05 | 0.000471 | 0.000346 | IL1B/IRS2/CDA/NR4A3/SLC7A7/IER3/PMAIP1/CH25H/PTAFR/RORC/ADM/EGR1 | 12 |  |
| BP | GO:0042119 | neutrophil activation | 5/155 | 42/18870 | 0.119048 | 14.49309 | 7.966715 | 2.33E-05 | 0.000476 | 0.00035 | FCER1G/ANXA3/LILRA2/PTAFR/TYROBP | 5 |  |
| BP | GO:0014074 | response to purine-containing compound | 8/155 | 143/18870 | 0.055944 | 6.810738 | 6.347615 | 2.41E-05 | 0.000484 | 0.000356 | IL1B/AQP9/FOS/THBD/PTAFR/FOSB/AREG/CYP1B1 | 8 |  |
| BP | GO:0034341 | response to type II interferon | 8/155 | 143/18870 | 0.055944 | 6.810738 | 6.347615 | 2.41E-05 | 0.000484 | 0.000356 | TLR2/CCL20/CXCL16/TLR4/IFNGR1/CCL4/HCK/AIF1 | 8 |  |
| BP | GO:2001235 | positive regulation of apoptotic signaling pathway | 8/155 | 145/18870 | 0.055172 | 6.716796 | 6.288849 | 2.66E-05 | 0.000532 | 0.000391 | S100A9/TLR4/THBS1/PMAIP1/DDIT3/S100A8/VNN1/G0S2 | 8 |  |
| BP | GO:0071356 | cellular response to tumor necrosis factor | 10/155 | 238/18870 | 0.042017 | 5.115207 | 5.814279 | 2.80E-05 | 0.000556 | 0.000409 | ZFP36/CCL20/FOS/THBS1/CCL4/TNFAIP3/CYP1B1/NFKBIA/LRRK2/CARD8 | 10 |  |
| BP | GO:0030100 | regulation of endocytosis | 11/155 | 291/18870 | 0.037801 | 4.601929 | 5.635284 | 2.94E-05 | 0.00058 | 0.000426 | TLR2/FCN1/IL1B/DYSF/FCER1G/CD14/CD300LF/PTX3/HCK/FGR/LRRK2 | 11 |  |
| BP | GO:0045765 | regulation of angiogenesis | 12/155 | 349/18870 | 0.034384 | 4.185969 | 5.467231 | 3.23E-05 | 0.000632 | 0.000464 | GADD45A/IL1B/C5AR1/THBS1/LRG1/ANXA3/TNFAIP3/ADM/CYP1B1/GLUL/BTG1/SULF1 | 12 |  |
| BP | GO:0030890 | positive regulation of B cell proliferation | 5/155 | 45/18870 | 0.111111 | 13.52688 | 7.656444 | 3.29E-05 | 0.000639 | 0.00047 | BST1/IRS2/TLR4/CDKN1A/PELI1 | 5 |  |
| BP | GO:1901342 | regulation of vasculature development | 12/155 | 354/18870 | 0.033898 | 4.126845 | 5.404802 | 3.70E-05 | 0.000716 | 0.000526 | GADD45A/IL1B/C5AR1/THBS1/LRG1/ANXA3/TNFAIP3/ADM/CYP1B1/GLUL/BTG1/SULF1 | 12 |  |
| BP | GO:0045444 | fat cell differentiation | 10/155 | 248/18870 | 0.040323 | 4.908949 | 5.639207 | 3.97E-05 | 0.000762 | 0.00056 | NR4A2/ZFP36/JDP2/NR4A3/LRG1/DDIT3/RORC/CEBPB/RGS2/STEAP4 | 10 |  |
| BP | GO:0002703 | regulation of leukocyte mediated immunity | 10/155 | 249/18870 | 0.040161 | 4.889234 | 5.622218 | 4.11E-05 | 0.000784 | 0.000576 | CD55/IL1B/TLR4/PTAFR/NLRP3/ICAM1/TYROBP/CRTAM/CD1D/FGR | 10 |  |
| BP | GO:0050673 | epithelial cell proliferation | 14/155 | 480/18870 | 0.029167 | 3.550806 | 5.151725 | 4.32E-05 | 0.000818 | 0.000601 | ZFP36/IRS2/C5AR1/NR4A3/THBS1/SULF2/LRG1/TNFAIP3/AREG/CDKN1A/NFKBIZ/CEBPB/EGR3/SULF1 | 14 |  |
| BP | GO:0050870 | positive regulation of T cell activation | 10/155 | 251/18870 | 0.039841 | 4.850276 | 5.588509 | 4.40E-05 | 0.000825 | 0.000607 | CD55/IL1B/VNN1/LILRB2/NLRP3/NFKBIZ/CD83/CD1D/EGR3/AIF1 | 10 |  |
| BP | GO:0032102 | negative regulation of response to external stimulus | 14/155 | 481/18870 | 0.029106 | 3.543424 | 5.142303 | 4.42E-05 | 0.000825 | 0.000607 | ZFP36/THBD/THBS1/IER3/LILRA2/TNFAIP3/NLRP3/PLAUR/ST6GAL1/HCK/FGR/CARD8/SERPINB2/AIF1 | 14 |  |
| BP | GO:0045785 | positive regulation of cell adhesion | 14/155 | 482/18870 | 0.029046 | 3.536073 | 5.132907 | 4.52E-05 | 0.000839 | 0.000617 | CD55/IL1B/NR4A3/ADAM9/VNN1/PTAFR/LILRB2/NLRP3/NFKBIZ/PLAUR/CD83/CD1D/EGR3/AIF1 | 14 |  |
| BP | GO:0019882 | antigen processing and presentation | 7/155 | 116/18870 | 0.060345 | 7.346496 | 6.239667 | 4.86E-05 | 0.000891 | 0.000655 | FCER1G/THBS1/RAB32/LILRB2/ICAM1/CLEC4A/CD1D | 7 |  |
| BP | GO:0097305 | response to alcohol | 10/155 | 254/18870 | 0.03937 | 4.79299 | 5.538609 | 4.86E-05 | 0.000891 | 0.000655 | FOS/CDA/CD14/S100A8/FOSB/CDKN1A/CCR5/ST6GAL1/CYP1B1/RGS2 | 10 |  |
| BP | GO:0036230 | granulocyte activation | 5/155 | 49/18870 | 0.102041 | 12.42265 | 7.285994 | 4.99E-05 | 0.00091 | 0.000669 | FCER1G/ANXA3/LILRA2/PTAFR/TYROBP | 5 |  |
| BP | GO:0044703 | multi-organism reproductive process | 9/155 | 206/18870 | 0.043689 | 5.318822 | 5.672075 | 5.26E-05 | 0.000952 | 0.0007 | IL1B/FOS/THBD/MMP9/PTAFR/FOSB/ADM/RGS2/GJA1 | 9 |  |
| BP | GO:0071346 | cellular response to type II interferon | 7/155 | 120/18870 | 0.058333 | 7.101613 | 6.10211 | 6.03E-05 | 0.001085 | 0.000798 | TLR2/CCL20/TLR4/IFNGR1/CCL4/HCK/AIF1 | 7 |  |
| BP | GO:0043281 | regulation of cysteine-type endopeptidase activity involved in apoptotic process | 8/155 | 163/18870 | 0.04908 | 5.975064 | 5.805449 | 6.13E-05 | 0.001096 | 0.000805 | S100A9/MMP9/THBS1/PMAIP1/S100A8/NLRP3/PLAUR/CARD8 | 8 |  |
| BP | GO:0015911 | long-chain fatty acid import across plasma membrane | 3/155 | 10/18870 | 0.3 | 36.52258 | 10.22537 | 6.25E-05 | 0.001098 | 0.000807 | ACSL1/IRS2/THBS1 | 3 |  |
| BP | GO:0070391 | response to lipoteichoic acid | 3/155 | 10/18870 | 0.3 | 36.52258 | 10.22537 | 6.25E-05 | 0.001098 | 0.000807 | TLR2/TLR4/CD14 | 3 |  |
| BP | GO:0071223 | cellular response to lipoteichoic acid | 3/155 | 10/18870 | 0.3 | 36.52258 | 10.22537 | 6.25E-05 | 0.001098 | 0.000807 | TLR2/TLR4/CD14 | 3 |  |
| BP | GO:0042730 | fibrinolysis | 4/155 | 27/18870 | 0.148148 | 18.03584 | 8.0615 | 6.63E-05 | 0.001148 | 0.000844 | THBD/THBS1/PLAUR/SERPINB2 | 4 |  |
| BP | GO:0032692 | negative regulation of interleukin-1 production | 5/155 | 52/18870 | 0.096154 | 11.70596 | 7.035351 | 6.67E-05 | 0.001148 | 0.000844 | IL1R2/TNFAIP3/NLRP3/CD33/CARD8 | 5 |  |
| BP | GO:0002292 | T cell differentiation involved in immune response | 6/155 | 84/18870 | 0.071429 | 8.695853 | 6.433164 | 6.68E-05 | 0.001148 | 0.000844 | CLEC4D/FCER1G/EOMES/RORC/NLRP3/NFKBIZ | 6 |  |
| BP | GO:0021782 | glial cell development | 7/155 | 122/18870 | 0.057377 | 6.985193 | 6.035677 | 6.70E-05 | 0.001148 | 0.000844 | TLR2/IL1B/C5AR1/S100A9/TLR4/IFNGR1/S100A8 | 7 |  |
| BP | GO:0002286 | T cell activation involved in immune response | 7/155 | 123/18870 | 0.056911 | 6.928403 | 6.003019 | 7.05E-05 | 0.001202 | 0.000884 | CLEC4D/FCER1G/EOMES/RORC/NLRP3/ICAM1/NFKBIZ | 7 |  |
| BP | GO:0043433 | negative regulation of DNA-binding transcription factor activity | 8/155 | 167/18870 | 0.047904 | 5.831949 | 5.707821 | 7.27E-05 | 0.001231 | 0.000905 | EOMES/DDIT3/TNFAIP3/NLRP3/CYP1B1/NFKBIA/LRRK2/CARD8 | 8 |  |
| BP | GO:0072676 | lymphocyte migration | 7/155 | 124/18870 | 0.056452 | 6.872529 | 5.970725 | 7.43E-05 | 0.001251 | 0.000919 | CCL20/CXCL16/CCL4/CH25H/ICAM1/CRTAM/AIF1 | 7 |  |
| BP | GO:0044706 | multi-multicellular organism process | 9/155 | 216/18870 | 0.041667 | 5.072581 | 5.478428 | 7.58E-05 | 0.001269 | 0.000933 | IL1B/FOS/THBD/MMP9/PTAFR/FOSB/ADM/RGS2/GJA1 | 9 |  |
| BP | GO:0051250 | negative regulation of lymphocyte activation | 8/155 | 169/18870 | 0.047337 | 5.762932 | 5.660186 | 7.90E-05 | 0.001315 | 0.000967 | TNFAIP3/LILRB2/CEBPB/PELI1/TYROBP/CRTAM/FGR/MNDA | 8 |  |
| BP | GO:0050672 | negative regulation of lymphocyte proliferation | 6/155 | 88/18870 | 0.068182 | 8.300587 | 6.247029 | 8.67E-05 | 0.001436 | 0.001055 | LILRB2/CEBPB/PELI1/TYROBP/CRTAM/MNDA | 6 |  |
| BP | GO:0032945 | negative regulation of mononuclear cell proliferation | 6/155 | 89/18870 | 0.067416 | 8.207321 | 6.202331 | 9.24E-05 | 0.001521 | 0.001118 | LILRB2/CEBPB/PELI1/TYROBP/CRTAM/MNDA | 6 |  |
| BP | GO:0038093 | Fc receptor signaling pathway | 5/155 | 56/18870 | 0.089286 | 10.86982 | 6.731438 | 9.55E-05 | 0.001555 | 0.001143 | CLEC4D/NR4A3/FCER1G/HCK/FGR | 5 |  |
| BP | GO:0050832 | defense response to fungus | 5/155 | 56/18870 | 0.089286 | 10.86982 | 6.731438 | 9.55E-05 | 0.001555 | 0.001143 | S100A12/CLEC4D/S100A9/S100A8/CLEC4A | 5 |  |
| BP | GO:0001906 | cell killing | 9/155 | 223/18870 | 0.040359 | 4.913352 | 5.349857 | 9.67E-05 | 0.001565 | 0.00115 | CD55/S100A12/TREM1/GZMA/ICAM1/TYROBP/CRTAM/CD1D/LYZ | 9 |  |
| BP | GO:0010955 | negative regulation of protein processing | 4/155 | 30/18870 | 0.133333 | 16.23226 | 7.598535 | 0.000102 | 0.001626 | 0.001195 | IL1R2/THBS1/LRRK2/CARD8 | 4 |  |
| BP | GO:1903318 | negative regulation of protein maturation | 4/155 | 30/18870 | 0.133333 | 16.23226 | 7.598535 | 0.000102 | 0.001626 | 0.001195 | IL1R2/THBS1/LRRK2/CARD8 | 4 |  |
| BP | GO:0033674 | positive regulation of kinase activity | 11/155 | 337/18870 | 0.032641 | 3.973772 | 5.012971 | 0.00011 | 0.001752 | 0.001287 | S100A12/IL1B/TLR4/THBS1/ADAM9/LILRA5/CDKN1A/PDGFC/FGR/LRRK2/EGR1 | 11 |  |
| BP | GO:0014831 | gastro-intestinal system smooth muscle contraction | 3/155 | 12/18870 | 0.25 | 30.43548 | 9.28238 | 0.000113 | 0.001764 | 0.001297 | SULF2/PTAFR/SULF1 | 3 |  |
| BP | GO:0042117 | monocyte activation | 3/155 | 12/18870 | 0.25 | 30.43548 | 9.28238 | 0.000113 | 0.001764 | 0.001297 | DYSF/ADAM9/CD33 | 3 |  |
| BP | GO:0051238 | sequestering of metal ion | 3/155 | 12/18870 | 0.25 | 30.43548 | 9.28238 | 0.000113 | 0.001764 | 0.001297 | FTH1/S100A9/S100A8 | 3 |  |
| BP | GO:0051549 | positive regulation of keratinocyte migration | 3/155 | 12/18870 | 0.25 | 30.43548 | 9.28238 | 0.000113 | 0.001764 | 0.001297 | MMP9/ADAM9/HBEGF | 3 |  |
| BP | GO:0045860 | positive regulation of protein kinase activity | 10/155 | 282/18870 | 0.035461 | 4.31709 | 5.107529 | 0.000116 | 0.001777 | 0.001306 | S100A12/IL1B/TLR4/THBS1/ADAM9/LILRA5/CDKN1A/PDGFC/LRRK2/EGR1 | 10 |  |
| BP | GO:0034143 | regulation of toll-like receptor 4 signaling pathway | 4/155 | 31/18870 | 0.129032 | 15.70864 | 7.458813 | 0.000116 | 0.001777 | 0.001306 | LILRA2/CD14/TNFAIP3/PELI1 | 4 |  |
| BP | GO:2000191 | regulation of fatty acid transport | 4/155 | 31/18870 | 0.129032 | 15.70864 | 7.458813 | 0.000116 | 0.001777 | 0.001306 | ACSL1/IL1B/IRS2/THBS1 | 4 |  |
| BP | GO:0097191 | extrinsic apoptotic signaling pathway | 9/155 | 229/18870 | 0.039301 | 4.784618 | 5.243853 | 0.000118 | 0.001804 | 0.001326 | IL1B/TLR4/THBS1/PMAIP1/TNFAIP3/BCL2A1/ICAM1/NFKBIZ/G0S2 | 9 |  |
| BP | GO:0010574 | regulation of vascular endothelial growth factor production | 5/155 | 59/18870 | 0.084746 | 10.31711 | 6.522991 | 0.000123 | 0.001852 | 0.001361 | IL1B/C5AR1/SULF2/CYP1B1/SULF1 | 5 |  |
| BP | GO:0043616 | keratinocyte proliferation | 5/155 | 59/18870 | 0.084746 | 10.31711 | 6.522991 | 0.000123 | 0.001852 | 0.001361 | ZFP36/LRG1/AREG/CDKN1A/NFKBIZ | 5 |  |
| BP | GO:0071900 | regulation of protein serine/threonine kinase activity | 10/155 | 285/18870 | 0.035088 | 4.271647 | 5.064691 | 0.000126 | 0.001896 | 0.001394 | S100A12/GADD45A/IL1B/TLR4/THBS1/ADAM9/CDKN1A/RGS2/PDGFC/LRRK2 | 10 |  |
| BP | GO:0046631 | alpha-beta T cell activation | 8/155 | 181/18870 | 0.044199 | 5.380859 | 5.38953 | 0.000127 | 0.001902 | 0.001398 | CD55/EOMES/RORC/NLRP3/NFKBIZ/CRTAM/CLEC4A/CD83 | 8 |  |
| BP | GO:0002431 | Fc receptor mediated stimulatory signaling pathway | 4/155 | 32/18870 | 0.125 | 15.21774 | 7.325438 | 0.000132 | 0.001945 | 0.00143 | NR4A3/FCER1G/HCK/FGR | 4 |  |
| BP | GO:0070498 | interleukin-1-mediated signaling pathway | 4/155 | 32/18870 | 0.125 | 15.21774 | 7.325438 | 0.000132 | 0.001945 | 0.00143 | IL1R2/IL1B/NFKBIA/EGR1 | 4 |  |
| BP | GO:0042129 | regulation of T cell proliferation | 8/155 | 182/18870 | 0.043956 | 5.351294 | 5.368069 | 0.000132 | 0.001945 | 0.00143 | CD55/IL1B/LILRB2/CEBPB/PELI1/CRTAM/CD1D/AIF1 | 8 |  |
| BP | GO:0071496 | cellular response to external stimulus | 11/155 | 346/18870 | 0.031792 | 3.870408 | 4.904105 | 0.000139 | 0.002029 | 0.001491 | NR4A2/GADD45A/IL1B/FOS/CDA/TLR4/PMAIP1/CDKN1A/KCNJ2/GLUL/LRRK2 | 11 |  |
| BP | GO:0045766 | positive regulation of angiogenesis | 8/155 | 185/18870 | 0.043243 | 5.264516 | 5.304622 | 0.000148 | 0.002123 | 0.001561 | IL1B/C5AR1/THBS1/LRG1/ANXA3/ADM/CYP1B1/BTG1 | 8 |  |
| BP | GO:1902107 | positive regulation of leukocyte differentiation | 8/155 | 185/18870 | 0.043243 | 5.264516 | 5.304622 | 0.000148 | 0.002123 | 0.001561 | FOS/VNN1/LILRB2/NLRP3/NFKBIZ/TYROBP/CD83/EGR3 | 8 |  |
| BP | GO:1903708 | positive regulation of hemopoiesis | 8/155 | 185/18870 | 0.043243 | 5.264516 | 5.304622 | 0.000148 | 0.002123 | 0.001561 | FOS/VNN1/LILRB2/NLRP3/NFKBIZ/TYROBP/CD83/EGR3 | 8 |  |
| BP | GO:1904018 | positive regulation of vasculature development | 8/155 | 185/18870 | 0.043243 | 5.264516 | 5.304622 | 0.000148 | 0.002123 | 0.001561 | IL1B/C5AR1/THBS1/LRG1/ANXA3/ADM/CYP1B1/BTG1 | 8 |  |
| BP | GO:0038094 | Fc-gamma receptor signaling pathway | 4/155 | 33/18870 | 0.121212 | 14.7566 | 7.197929 | 0.000149 | 0.002124 | 0.001561 | CLEC4D/FCER1G/HCK/FGR | 4 |  |
| BP | GO:0043410 | positive regulation of MAPK cascade | 13/155 | 474/18870 | 0.027426 | 3.338914 | 4.693395 | 0.000151 | 0.002139 | 0.001572 | S100A12/GADD45A/IL1B/CCL20/C5AR1/TLR4/THBS1/ADAM9/CCL4/LILRA5/ICAM1/PDGFC/LRRK2 | 13 |  |
| BP | GO:0007565 | female pregnancy | 8/155 | 186/18870 | 0.043011 | 5.236212 | 5.283779 | 0.000154 | 0.002171 | 0.001596 | IL1B/FOS/THBD/MMP9/FOSB/ADM/RGS2/GJA1 | 8 |  |
| BP | GO:0046942 | carboxylic acid transport | 11/155 | 351/18870 | 0.031339 | 3.815274 | 4.845191 | 0.000157 | 0.00221 | 0.001625 | ACSL1/IL1B/AQP9/IRS2/RBP7/SLC7A7/THBS1/FOLR3/RGS2/SLC22A4/GJA1 | 11 |  |
| BP | GO:0071902 | positive regulation of protein serine/threonine kinase activity | 7/155 | 140/18870 | 0.05 | 6.087097 | 5.498073 | 0.000159 | 0.002223 | 0.001634 | S100A12/IL1B/TLR4/THBS1/ADAM9/PDGFC/LRRK2 | 7 |  |
| BP | GO:0015849 | organic acid transport | 11/155 | 352/18870 | 0.03125 | 3.804435 | 4.833538 | 0.000161 | 0.002244 | 0.001649 | ACSL1/IL1B/AQP9/IRS2/RBP7/SLC7A7/THBS1/FOLR3/RGS2/SLC22A4/GJA1 | 11 |  |
| BP | GO:0051347 | positive regulation of transferase activity | 12/155 | 414/18870 | 0.028986 | 3.528752 | 4.734596 | 0.000164 | 0.002268 | 0.001667 | S100A12/IL1B/TLR4/THBS1/ADAM9/LILRA5/CDKN1A/ARRDC4/PDGFC/FGR/LRRK2/EGR1 | 12 |  |
| BP | GO:0010573 | vascular endothelial growth factor production | 5/155 | 63/18870 | 0.079365 | 9.662058 | 6.26725 | 0.000168 | 0.002315 | 0.001702 | IL1B/C5AR1/SULF2/CYP1B1/SULF1 | 5 |  |
| BP | GO:2001242 | regulation of intrinsic apoptotic signaling pathway | 8/155 | 190/18870 | 0.042105 | 5.125976 | 5.201882 | 0.000178 | 0.002441 | 0.001794 | S100A9/MMP9/PMAIP1/DDIT3/S100A8/VNN1/PLAUR/LRRK2 | 8 |  |
| BP | GO:2001244 | positive regulation of intrinsic apoptotic signaling pathway | 5/155 | 64/18870 | 0.078125 | 9.511089 | 6.206864 | 0.000181 | 0.002472 | 0.001817 | S100A9/PMAIP1/DDIT3/S100A8/VNN1 | 5 |  |
| BP | GO:2001234 | negative regulation of apoptotic signaling pathway | 9/155 | 243/18870 | 0.037037 | 4.508961 | 5.010206 | 0.000185 | 0.002483 | 0.001825 | NR4A2/IL1B/MMP9/THBS1/IER3/TNFAIP3/ICAM1/PLAUR/LRRK2 | 9 |  |
| BP | GO:0031665 | negative regulation of lipopolysaccharide-mediated signaling pathway | 3/155 | 14/18870 | 0.214286 | 26.08756 | 8.545608 | 0.000185 | 0.002483 | 0.001825 | LILRA2/TNFAIP3/CARD8 | 3 |  |
| BP | GO:0051412 | response to corticosterone | 3/155 | 14/18870 | 0.214286 | 26.08756 | 8.545608 | 0.000185 | 0.002483 | 0.001825 | FOS/FOSB/CDKN1A | 3 |  |
| BP | GO:0051547 | regulation of keratinocyte migration | 3/155 | 14/18870 | 0.214286 | 26.08756 | 8.545608 | 0.000185 | 0.002483 | 0.001825 | MMP9/ADAM9/HBEGF | 3 |  |
| BP | GO:0051091 | positive regulation of DNA-binding transcription factor activity | 9/155 | 244/18870 | 0.036885 | 4.490481 | 4.994198 | 0.000191 | 0.002544 | 0.00187 | S100A12/TLR2/IL1B/S100A9/TLR4/ANXA3/DDIT3/S100A8/NLRP3 | 9 |  |
| BP | GO:0007249 | canonical NF-kappaB signal transduction | 10/155 | 300/18870 | 0.033333 | 4.058065 | 4.858998 | 0.000192 | 0.002546 | 0.001871 | S100A12/CLEC4D/TLR2/IL1B/TLR4/TNFAIP3/PELI1/NFKBIA/CARD8/GJA1 | 10 |  |
| BP | GO:0032613 | interleukin-10 production | 5/155 | 65/18870 | 0.076923 | 9.364764 | 6.147791 | 0.000195 | 0.002552 | 0.001876 | TLR2/TLR4/LILRA5/TYROBP/CD83 | 5 |  |
| BP | GO:0032653 | regulation of interleukin-10 production | 5/155 | 65/18870 | 0.076923 | 9.364764 | 6.147791 | 0.000195 | 0.002552 | 0.001876 | TLR2/TLR4/LILRA5/TYROBP/CD83 | 5 |  |
| BP | GO:0070059 | intrinsic apoptotic signaling pathway in response to endoplasmic reticulum stress | 5/155 | 65/18870 | 0.076923 | 9.364764 | 6.147791 | 0.000195 | 0.002552 | 0.001876 | PPP1R15A/PMAIP1/DDIT3/CEBPB/LRRK2 | 5 |  |
| BP | GO:0062207 | regulation of pattern recognition receptor signaling pathway | 7/155 | 145/18870 | 0.048276 | 5.877197 | 5.365235 | 0.000197 | 0.002575 | 0.001893 | TLR4/LILRA2/CD14/TNFAIP3/CD300LF/PELI1/CARD8 | 7 |  |
| BP | GO:2000116 | regulation of cysteine-type endopeptidase activity | 8/155 | 195/18870 | 0.041026 | 4.994541 | 5.102691 | 0.000212 | 0.002758 | 0.002027 | S100A9/MMP9/THBS1/PMAIP1/S100A8/NLRP3/PLAUR/CARD8 | 8 |  |
| BP | GO:0070613 | regulation of protein processing | 5/155 | 67/18870 | 0.074627 | 9.085219 | 6.033384 | 0.000225 | 0.002905 | 0.002135 | IL1R2/THBS1/PLAUR/LRRK2/CARD8 | 5 |  |
| BP | GO:0032602 | chemokine production | 6/155 | 105/18870 | 0.057143 | 6.956682 | 5.570192 | 0.00023 | 0.002958 | 0.002174 | TLR2/IL1B/TREM1/TLR4/EGR1/AIF1 | 6 |  |
| BP | GO:0002687 | positive regulation of leukocyte migration | 7/155 | 149/18870 | 0.04698 | 5.71942 | 5.263354 | 0.000233 | 0.002989 | 0.002197 | CCL20/C5AR1/THBS1/CCL4/PTAFR/ICAM1/AIF1 | 7 |  |
| BP | GO:0051092 | positive regulation of NF-kappaB transcription factor activity | 7/155 | 150/18870 | 0.046667 | 5.68129 | 5.23846 | 0.000243 | 0.003087 | 0.002269 | S100A12/TLR2/IL1B/S100A9/TLR4/S100A8/NLRP3 | 7 |  |
| BP | GO:0120161 | regulation of cold-induced thermogenesis | 7/155 | 150/18870 | 0.046667 | 5.68129 | 5.23846 | 0.000243 | 0.003087 | 0.002269 | ACSL1/TLR4/DDIT3/CEBPB/G0S2/PDGFC/GJA1 | 7 |  |
| BP | GO:0002705 | positive regulation of leukocyte mediated immunity | 7/155 | 151/18870 | 0.046358 | 5.643666 | 5.213789 | 0.000253 | 0.003187 | 0.002343 | CD55/IL1B/PTAFR/NLRP3/TYROBP/CRTAM/CD1D | 7 |  |
| BP | GO:0106106 | cold-induced thermogenesis | 7/155 | 151/18870 | 0.046358 | 5.643666 | 5.213789 | 0.000253 | 0.003187 | 0.002343 | ACSL1/TLR4/DDIT3/CEBPB/G0S2/PDGFC/GJA1 | 7 |  |
| BP | GO:0045670 | regulation of osteoclast differentiation | 5/155 | 69/18870 | 0.072464 | 8.821879 | 5.923666 | 0.000258 | 0.003235 | 0.002378 | FOS/TLR4/CEBPB/TYROBP/TOB2 | 5 |  |
| BP | GO:0052547 | regulation of peptidase activity | 10/155 | 312/18870 | 0.032051 | 3.901985 | 4.703837 | 0.000262 | 0.003276 | 0.002408 | CSTA/S100A9/MMP9/THBS1/PMAIP1/S100A8/NLRP3/PLAUR/CARD8/SERPINB2 | 10 |  |
| BP | GO:0051770 | positive regulation of nitric-oxide synthase biosynthetic process | 3/155 | 16/18870 | 0.1875 | 22.82661 | 7.948588 | 0.000281 | 0.003499 | 0.002572 | TLR2/TLR4/LRRK2 | 3 |  |
| BP | GO:1903317 | regulation of protein maturation | 5/155 | 71/18870 | 0.070423 | 8.573376 | 5.818308 | 0.000295 | 0.003643 | 0.002678 | IL1R2/THBS1/PLAUR/LRRK2/CARD8 | 5 |  |
| BP | GO:0002444 | myeloid leukocyte mediated immunity | 6/155 | 110/18870 | 0.054545 | 6.640469 | 5.399338 | 0.000296 | 0.003643 | 0.002678 | TREM1/NR4A3/ANXA3/PTAFR/TYROBP/FGR | 6 |  |
| BP | GO:0045807 | positive regulation of endocytosis | 7/155 | 155/18870 | 0.045161 | 5.498023 | 5.117266 | 0.000297 | 0.003643 | 0.002678 | FCN1/IL1B/FCER1G/CD14/CD300LF/PTX3/LRRK2 | 7 |  |
| BP | GO:0015711 | organic anion transport | 12/155 | 443/18870 | 0.027088 | 3.29775 | 4.453718 | 0.000304 | 0.003717 | 0.002732 | ACSL1/IL1B/AQP9/IRS2/RBP7/SLC7A7/THBS1/SLC2A3/FOLR3/RGS2/SLC22A4/GJA1 | 12 |  |
| BP | GO:0046718 | symbiont entry into host cell | 7/155 | 156/18870 | 0.044872 | 5.462779 | 5.093658 | 0.000309 | 0.003754 | 0.002759 | CD55/FCN1/ANPEP/CH25H/ICAM1/PTX3/CCR5 | 7 |  |
| BP | GO:0071347 | cellular response to interleukin-1 | 6/155 | 111/18870 | 0.054054 | 6.580645 | 5.366442 | 0.00031 | 0.003754 | 0.002759 | IL1R2/IL1B/CCL20/CCL4/NFKBIA/EGR1 | 6 |  |
| BP | GO:0002460 | adaptive immune response based on somatic recombination of immune receptors built from immunoglobulin superfamily domains | 11/155 | 380/18870 | 0.028947 | 3.524109 | 4.523523 | 0.000311 | 0.003754 | 0.002759 | CD55/IL1B/FCER1G/TLR4/TNFAIP3/RORC/NLRP3/ICAM1/NFKBIZ/CSF2RB/CD1D | 11 |  |
| BP | GO:0032722 | positive regulation of chemokine production | 5/155 | 72/18870 | 0.069444 | 8.454301 | 5.76717 | 0.000315 | 0.003769 | 0.002771 | TLR2/IL1B/TLR4/EGR1/AIF1 | 5 |  |
| BP | GO:0050766 | positive regulation of phagocytosis | 5/155 | 72/18870 | 0.069444 | 8.454301 | 5.76717 | 0.000315 | 0.003769 | 0.002771 | FCN1/IL1B/FCER1G/CD300LF/PTX3 | 5 |  |
| BP | GO:1901654 | response to ketone | 8/155 | 208/18870 | 0.038462 | 4.682382 | 4.859894 | 0.000328 | 0.003914 | 0.002877 | TLR2/FOS/CDA/THBS1/PTAFR/FOSB/CDKN1A/CYP1B1 | 8 |  |
| BP | GO:2001236 | regulation of extrinsic apoptotic signaling pathway | 7/155 | 158/18870 | 0.044304 | 5.39363 | 5.047046 | 0.000333 | 0.003959 | 0.00291 | IL1B/TLR4/THBS1/PMAIP1/TNFAIP3/ICAM1/G0S2 | 7 |  |
| BP | GO:0044539 | long-chain fatty acid import into cell | 3/155 | 17/18870 | 0.176471 | 21.48387 | 7.689385 | 0.00034 | 0.004016 | 0.002952 | ACSL1/IRS2/THBS1 | 3 |  |
| BP | GO:0032733 | positive regulation of interleukin-10 production | 4/155 | 42/18870 | 0.095238 | 11.59447 | 6.255286 | 0.000384 | 0.004441 | 0.003265 | TLR2/TLR4/LILRA5/CD83 | 4 |  |
| BP | GO:0140467 | integrated stress response signaling | 4/155 | 42/18870 | 0.095238 | 11.59447 | 6.255286 | 0.000384 | 0.004441 | 0.003265 | PPP1R15A/FOS/DDIT3/CEBPB | 4 |  |
| BP | GO:0002285 | lymphocyte activation involved in immune response | 8/155 | 213/18870 | 0.037559 | 4.572467 | 4.771803 | 0.000385 | 0.004441 | 0.003265 | CLEC4D/FCER1G/TLR4/EOMES/RORC/NLRP3/ICAM1/NFKBIZ | 8 |  |
| BP | GO:0009612 | response to mechanical stimulus | 8/155 | 213/18870 | 0.037559 | 4.572467 | 4.771803 | 0.000385 | 0.004441 | 0.003265 | GADD45A/IL1B/FOS/TLR4/THBS1/FOSB/KCNJ2/NFKBIA | 8 |  |
| BP | GO:0042098 | T cell proliferation | 8/155 | 213/18870 | 0.037559 | 4.572467 | 4.771803 | 0.000385 | 0.004441 | 0.003265 | CD55/IL1B/LILRB2/CEBPB/PELI1/CRTAM/CD1D/AIF1 | 8 |  |
| BP | GO:0045637 | regulation of myeloid cell differentiation | 8/155 | 213/18870 | 0.037559 | 4.572467 | 4.771803 | 0.000385 | 0.004441 | 0.003265 | ZFP36/FOS/CSF3R/TLR4/CEBPB/TYROBP/TOB2/NFKBIA | 8 |  |
| BP | GO:0045582 | positive regulation of T cell differentiation | 6/155 | 116/18870 | 0.051724 | 6.296997 | 5.207834 | 0.000393 | 0.004522 | 0.003324 | VNN1/LILRB2/NLRP3/NFKBIZ/CD83/EGR3 | 6 |  |
| BP | GO:0044409 | symbiont entry into host | 7/155 | 163/18870 | 0.042945 | 5.228181 | 4.933904 | 0.000402 | 0.004609 | 0.003388 | CD55/FCN1/ANPEP/CH25H/ICAM1/PTX3/CCR5 | 7 |  |
| BP | GO:0051917 | regulation of fibrinolysis | 3/155 | 18/18870 | 0.166667 | 20.29032 | 7.451478 | 0.000405 | 0.004621 | 0.003397 | THBD/THBS1/PLAUR | 3 |  |
| BP | GO:0032890 | regulation of organic acid transport | 5/155 | 77/18870 | 0.064935 | 7.90532 | 5.525563 | 0.00043 | 0.004878 | 0.003586 | ACSL1/IL1B/IRS2/THBS1/RGS2 | 5 |  |
| BP | GO:0050830 | defense response to Gram-positive bacterium | 6/155 | 118/18870 | 0.050847 | 6.190268 | 5.146979 | 0.000431 | 0.004878 | 0.003586 | TLR2/IL1B/C5AR1/MPEG1/FGR/LYZ | 6 |  |
| BP | GO:0042594 | response to starvation | 8/155 | 218/18870 | 0.036697 | 4.467594 | 4.686398 | 0.000449 | 0.005025 | 0.003694 | ZFP36/FOS/PMAIP1/DDIT3/ADM/CDKN1A/GLUL/LRRK2 | 8 |  |
| BP | GO:0071214 | cellular response to abiotic stimulus | 10/155 | 334/18870 | 0.02994 | 3.644968 | 4.438447 | 0.000449 | 0.005025 | 0.003694 | GADD45A/IL1B/MMP9/TLR4/PTAFR/NLRP3/CDKN1A/MME/KCNJ2/EGR1 | 10 |  |
| BP | GO:0104004 | cellular response to environmental stimulus | 10/155 | 334/18870 | 0.02994 | 3.644968 | 4.438447 | 0.000449 | 0.005025 | 0.003694 | GADD45A/IL1B/MMP9/TLR4/PTAFR/NLRP3/CDKN1A/MME/KCNJ2/EGR1 | 10 |  |
| BP | GO:0046209 | nitric oxide metabolic process | 5/155 | 78/18870 | 0.064103 | 7.80397 | 5.479849 | 0.000457 | 0.005089 | 0.003741 | TLR2/IL1B/TLR4/PTX3/CYP1B1 | 5 |  |
| BP | GO:0032691 | negative regulation of interleukin-1 beta production | 4/155 | 44/18870 | 0.090909 | 11.06745 | 6.084321 | 0.00046 | 0.00509 | 0.003742 | TNFAIP3/NLRP3/CD33/CARD8 | 4 |  |
| BP | GO:2000516 | positive regulation of CD4-positive, alpha-beta T cell activation | 4/155 | 44/18870 | 0.090909 | 11.06745 | 6.084321 | 0.00046 | 0.00509 | 0.003742 | CD55/NLRP3/NFKBIZ/CD83 | 4 |  |
| BP | GO:0070431 | nucleotide-binding oligomerization domain containing 2 signaling pathway | 3/155 | 19/18870 | 0.157895 | 19.22241 | 7.23204 | 0.000478 | 0.005248 | 0.003857 | TLR4/TNFAIP3/NFKBIA | 3 |  |
| BP | GO:0098543 | detection of other organism | 3/155 | 19/18870 | 0.157895 | 19.22241 | 7.23204 | 0.000478 | 0.005248 | 0.003857 | TLR2/TLR4/CD1D | 3 |  |
| BP | GO:1990845 | adaptive thermogenesis | 7/155 | 168/18870 | 0.041667 | 5.072581 | 4.825315 | 0.000483 | 0.005275 | 0.003878 | ACSL1/TLR4/DDIT3/CEBPB/G0S2/PDGFC/GJA1 | 7 |  |
| BP | GO:2001057 | reactive nitrogen species metabolic process | 5/155 | 79/18870 | 0.063291 | 7.705186 | 5.43494 | 0.000484 | 0.005275 | 0.003878 | TLR2/IL1B/TLR4/PTX3/CYP1B1 | 5 |  |
| BP | GO:0042113 | B cell activation | 9/155 | 278/18870 | 0.032374 | 3.941286 | 4.496162 | 0.000496 | 0.005383 | 0.003957 | BST1/IRS2/TLR4/TNFAIP3/CDKN1A/NFKBIZ/PELI1/TYROBP/MNDA | 9 |  |
| BP | GO:0051051 | negative regulation of transport | 12/155 | 469/18870 | 0.025586 | 3.114932 | 4.220924 | 0.000507 | 0.005476 | 0.004026 | TLR2/IL1B/IRS2/DYSF/MMP9/THBS1/LILRB2/CD33/CD300LF/RGS2/LRRK2/GJA1 | 12 |  |
| BP | GO:0002367 | cytokine production involved in immune response | 6/155 | 122/18870 | 0.04918 | 5.987308 | 5.029375 | 0.000515 | 0.005544 | 0.004075 | CD55/IL1B/TREM1/NR4A3/TLR4/NLRP3 | 6 |  |
| BP | GO:0002720 | positive regulation of cytokine production involved in immune response | 5/155 | 81/18870 | 0.061728 | 7.514934 | 5.347442 | 0.000543 | 0.00581 | 0.00427 | CD55/IL1B/NR4A3/TLR4/NLRP3 | 5 |  |
| BP | GO:0043299 | leukocyte degranulation | 5/155 | 81/18870 | 0.061728 | 7.514934 | 5.347442 | 0.000543 | 0.00581 | 0.00427 | NR4A3/ANXA3/PTAFR/HCK/FGR | 5 |  |
| BP | GO:0050678 | regulation of epithelial cell proliferation | 11/155 | 407/18870 | 0.027027 | 3.290323 | 4.250971 | 0.000553 | 0.00587 | 0.004315 | ZFP36/IRS2/C5AR1/NR4A3/THBS1/SULF2/LRG1/TNFAIP3/AREG/EGR3/SULF1 | 11 |  |
| BP | GO:0051767 | nitric-oxide synthase biosynthetic process | 3/155 | 20/18870 | 0.15 | 18.26129 | 7.028748 | 0.000559 | 0.00587 | 0.004315 | TLR2/TLR4/LRRK2 | 3 |  |
| BP | GO:0051769 | regulation of nitric-oxide synthase biosynthetic process | 3/155 | 20/18870 | 0.15 | 18.26129 | 7.028748 | 0.000559 | 0.00587 | 0.004315 | TLR2/TLR4/LRRK2 | 3 |  |
| BP | GO:0140354 | lipid import into cell | 3/155 | 20/18870 | 0.15 | 18.26129 | 7.028748 | 0.000559 | 0.00587 | 0.004315 | ACSL1/IRS2/THBS1 | 3 |  |
| BP | GO:1902001 | fatty acid transmembrane transport | 3/155 | 20/18870 | 0.15 | 18.26129 | 7.028748 | 0.000559 | 0.00587 | 0.004315 | ACSL1/IRS2/THBS1 | 3 |  |
| BP | GO:0043542 | endothelial cell migration | 9/155 | 284/18870 | 0.03169 | 3.858019 | 4.416484 | 0.000578 | 0.00605 | 0.004447 | S100A12/GADD45A/S100A9/S100P/THBS1/ANXA3/CYP1B1/GLUL/EGR3 | 9 |  |
| BP | GO:0002685 | regulation of leukocyte migration | 8/155 | 230/18870 | 0.034783 | 4.234502 | 4.491525 | 0.000639 | 0.006608 | 0.004858 | BST1/CCL20/C5AR1/THBS1/CCL4/PTAFR/ICAM1/AIF1 | 8 |  |
| BP | GO:0052548 | regulation of endopeptidase activity | 9/155 | 288/18870 | 0.03125 | 3.804435 | 4.364564 | 0.000639 | 0.006608 | 0.004858 | S100A9/MMP9/THBS1/PMAIP1/S100A8/NLRP3/PLAUR/CARD8/SERPINB2 | 9 |  |
| BP | GO:0050871 | positive regulation of B cell activation | 5/155 | 84/18870 | 0.059524 | 7.246544 | 5.221649 | 0.000642 | 0.006608 | 0.004858 | BST1/IRS2/TLR4/CDKN1A/PELI1 | 5 |  |
| BP | GO:0030195 | negative regulation of blood coagulation | 4/155 | 48/18870 | 0.083333 | 10.14516 | 5.773305 | 0.000643 | 0.006608 | 0.004858 | THBD/THBS1/PLAUR/SERPINB2 | 4 |  |
| BP | GO:0043277 | apoptotic cell clearance | 4/155 | 48/18870 | 0.083333 | 10.14516 | 5.773305 | 0.000643 | 0.006608 | 0.004858 | FCN1/THBS1/CD300LF/TYROBP | 4 |  |
| BP | GO:0050765 | negative regulation of phagocytosis | 3/155 | 21/18870 | 0.142857 | 17.39171 | 6.839668 | 0.000649 | 0.006639 | 0.00488 | TLR2/DYSF/CD300LF | 3 |  |
| BP | GO:0051101 | regulation of DNA binding | 5/155 | 85/18870 | 0.058824 | 7.16129 | 5.181087 | 0.000678 | 0.006913 | 0.005082 | MMP9/DDIT3/GZMA/PLAUR/NFKBIA | 5 |  |
| BP | GO:0030522 | intracellular receptor signaling pathway | 10/155 | 353/18870 | 0.028329 | 3.44878 | 4.226659 | 0.00069 | 0.007001 | 0.005146 | NR4A2/NR4A3/TLR4/TNFAIP3/RORC/NLRP3/PELI1/NFKBIA/CNOT1/CARD8 | 10 |  |
| BP | GO:0002456 | T cell mediated immunity | 6/155 | 129/18870 | 0.046512 | 5.662416 | 4.83565 | 0.000691 | 0.007001 | 0.005146 | CD55/IL1B/NLRP3/ICAM1/NFKBIZ/CD1D | 6 |  |
| BP | GO:0014911 | positive regulation of smooth muscle cell migration | 4/155 | 49/18870 | 0.081633 | 9.938117 | 5.701224 | 0.000696 | 0.007001 | 0.005146 | NR4A3/TLR4/CYP1B1/AIF1 | 4 |  |
| BP | GO:1900047 | negative regulation of hemostasis | 4/155 | 49/18870 | 0.081633 | 9.938117 | 5.701224 | 0.000696 | 0.007001 | 0.005146 | THBD/THBS1/PLAUR/SERPINB2 | 4 |  |
| BP | GO:0045621 | positive regulation of lymphocyte differentiation | 6/155 | 130/18870 | 0.046154 | 5.618859 | 4.809134 | 0.00072 | 0.007215 | 0.005304 | VNN1/LILRB2/NLRP3/NFKBIZ/CD83/EGR3 | 6 |  |
| BP | GO:0045861 | negative regulation of proteolysis | 8/155 | 235/18870 | 0.034043 | 4.144406 | 4.414213 | 0.000735 | 0.007347 | 0.005401 | IL1R2/CSTA/MMP9/THBS1/PLAUR/LRRK2/CARD8/SERPINB2 | 8 |  |
| BP | GO:0045580 | regulation of T cell differentiation | 7/155 | 181/18870 | 0.038674 | 4.708252 | 4.562058 | 0.000751 | 0.007458 | 0.005482 | VNN1/LILRB2/NLRP3/NFKBIZ/CRTAM/CD83/EGR3 | 7 |  |
| BP | GO:1902041 | regulation of extrinsic apoptotic signaling pathway via death domain receptors | 4/155 | 50/18870 | 0.08 | 9.739355 | 5.631187 | 0.000752 | 0.007458 | 0.005482 | THBS1/PMAIP1/TNFAIP3/ICAM1 | 4 |  |
| BP | GO:0061041 | regulation of wound healing | 6/155 | 133/18870 | 0.045113 | 5.492117 | 4.73121 | 0.000811 | 0.00802 | 0.005895 | THBD/THBS1/HBEGF/TNFAIP3/PLAUR/SERPINB2 | 6 |  |
| BP | GO:0050829 | defense response to Gram-negative bacterium | 5/155 | 89/18870 | 0.05618 | 6.839435 | 5.025182 | 0.000835 | 0.008232 | 0.006051 | TREM1/TLR4/MPEG1/NFKBIZ/LYZ | 5 |  |
| BP | GO:0046597 | negative regulation of viral entry into host cell | 3/155 | 23/18870 | 0.130435 | 15.87938 | 6.497901 | 0.000853 | 0.008322 | 0.006117 | FCN1/CH25H/PTX3 | 3 |  |
| BP | GO:0072574 | hepatocyte proliferation | 3/155 | 23/18870 | 0.130435 | 15.87938 | 6.497901 | 0.000853 | 0.008322 | 0.006117 | SULF2/TNFAIP3/CEBPB | 3 |  |
| BP | GO:0072575 | epithelial cell proliferation involved in liver morphogenesis | 3/155 | 23/18870 | 0.130435 | 15.87938 | 6.497901 | 0.000853 | 0.008322 | 0.006117 | SULF2/TNFAIP3/CEBPB | 3 |  |
| BP | GO:0050819 | negative regulation of coagulation | 4/155 | 52/18870 | 0.076923 | 9.364764 | 5.496852 | 0.000873 | 0.008482 | 0.006235 | THBD/THBS1/PLAUR/SERPINB2 | 4 |  |
| BP | GO:0034219 | carbohydrate transmembrane transport | 6/155 | 136/18870 | 0.044118 | 5.370968 | 4.655616 | 0.000911 | 0.008828 | 0.006489 | IL1B/AQP9/IRS2/NR4A3/SLC2A3/ITLN1 | 6 |  |
| BP | GO:0070098 | chemokine-mediated signaling pathway | 5/155 | 91/18870 | 0.054945 | 6.689117 | 4.950793 | 0.000924 | 0.00892 | 0.006557 | CCL20/CCL4/CXCL1/CCR5/CCRL2 | 5 |  |
| BP | GO:1904645 | response to amyloid-beta | 4/155 | 53/18870 | 0.075472 | 9.188071 | 5.432374 | 0.000938 | 0.008996 | 0.006613 | MMP9/TLR4/ICAM1/GJA1 | 4 |  |
| BP | GO:2001238 | positive regulation of extrinsic apoptotic signaling pathway | 4/155 | 53/18870 | 0.075472 | 9.188071 | 5.432374 | 0.000938 | 0.008996 | 0.006613 | TLR4/THBS1/PMAIP1/G0S2 | 4 |  |
| BP | GO:0010466 | negative regulation of peptidase activity | 6/155 | 137/18870 | 0.043796 | 5.331764 | 4.630914 | 0.000947 | 0.00905 | 0.006652 | CSTA/MMP9/THBS1/PLAUR/CARD8/SERPINB2 | 6 |  |
| BP | GO:0016485 | protein processing | 8/155 | 245/18870 | 0.032653 | 3.975247 | 4.265827 | 0.000963 | 0.009176 | 0.006745 | IL1R2/SRGN/THBD/THBS1/PLAUR/MME/LRRK2/CARD8 | 8 |  |
| BP | GO:0032891 | negative regulation of organic acid transport | 3/155 | 24/18870 | 0.125 | 15.21774 | 6.342669 | 0.000969 | 0.009176 | 0.006745 | IRS2/THBS1/RGS2 | 3 |  |
| BP | GO:0040037 | negative regulation of fibroblast growth factor receptor signaling pathway | 3/155 | 24/18870 | 0.125 | 15.21774 | 6.342669 | 0.000969 | 0.009176 | 0.006745 | THBS1/SULF2/SULF1 | 3 |  |
| BP | GO:0070555 | response to interleukin-1 | 6/155 | 140/18870 | 0.042857 | 5.217512 | 4.558236 | 0.001059 | 0.009993 | 0.007346 | IL1R2/IL1B/CCL20/CCL4/NFKBIA/EGR1 | 6 |  |
| BP | GO:0051047 | positive regulation of secretion | 9/155 | 310/18870 | 0.029032 | 3.534443 | 4.094679 | 0.001075 | 0.010108 | 0.00743 | TLR2/IL1B/IRS2/TLR4/ADAM9/S100A8/PTAFR/CD33/FGR | 9 |  |
| BP | GO:0048143 | astrocyte activation | 3/155 | 25/18870 | 0.12 | 14.60903 | 6.196473 | 0.001095 | 0.010198 | 0.007496 | IL1B/C5AR1/IFNGR1 | 3 |  |
| BP | GO:0072576 | liver morphogenesis | 3/155 | 25/18870 | 0.12 | 14.60903 | 6.196473 | 0.001095 | 0.010198 | 0.007496 | SULF2/TNFAIP3/CEBPB | 3 |  |
| BP | GO:0090200 | positive regulation of release of cytochrome c from mitochondria | 3/155 | 25/18870 | 0.12 | 14.60903 | 6.196473 | 0.001095 | 0.010198 | 0.007496 | MMP9/PMAIP1/PLAUR | 3 |  |
| BP | GO:1990874 | vascular associated smooth muscle cell proliferation | 5/155 | 95/18870 | 0.052632 | 6.40747 | 4.808519 | 0.001121 | 0.01041 | 0.007652 | MMP9/NR4A3/DDIT3/CDKN1A/GJA1 | 5 |  |
| BP | GO:0010720 | positive regulation of cell development | 11/155 | 444/18870 | 0.024775 | 3.016129 | 3.912368 | 0.001126 | 0.010419 | 0.007658 | TLR2/IL1B/FOS/VNN1/LILRB2/NLRP3/NFKBIZ/MME/TYROBP/CD83/EGR3 | 11 |  |
| BP | GO:0010001 | glial cell differentiation | 8/155 | 252/18870 | 0.031746 | 3.864823 | 4.166554 | 0.001154 | 0.010614 | 0.007802 | TLR2/IL1B/C5AR1/S100A9/TLR4/IFNGR1/EOMES/S100A8 | 8 |  |
| BP | GO:0001836 | release of cytochrome c from mitochondria | 4/155 | 56/18870 | 0.071429 | 8.695853 | 5.248746 | 0.001154 | 0.010614 | 0.007802 | MMP9/PMAIP1/BCL2A1/PLAUR | 4 |  |
| BP | GO:0014909 | smooth muscle cell migration | 5/155 | 96/18870 | 0.052083 | 6.340726 | 4.774225 | 0.001175 | 0.010772 | 0.007918 | NR4A3/TLR4/DDIT3/CYP1B1/AIF1 | 5 |  |
| BP | GO:0010565 | regulation of cellular ketone metabolic process | 6/155 | 144/18870 | 0.041667 | 5.072581 | 4.46451 | 0.001225 | 0.011172 | 0.008212 | IL1B/IRS2/NR4A3/SLC7A7/ADM/EGR1 | 6 |  |
| BP | GO:0002726 | positive regulation of T cell cytokine production | 3/155 | 26/18870 | 0.115385 | 14.04715 | 6.058443 | 0.00123 | 0.011172 | 0.008212 | CD55/IL1B/NLRP3 | 3 |  |
| BP | GO:0098581 | detection of external biotic stimulus | 3/155 | 26/18870 | 0.115385 | 14.04715 | 6.058443 | 0.00123 | 0.011172 | 0.008212 | TLR2/TLR4/CD1D | 3 |  |
| BP | GO:0062197 | cellular response to chemical stress | 9/155 | 317/18870 | 0.028391 | 3.456396 | 4.013897 | 0.001255 | 0.011362 | 0.008352 | NR4A2/FOS/MMP9/DDIT3/TNFAIP3/NLRP3/CYP1B1/LRRK2/AIF1 | 9 |  |
| BP | GO:0002753 | cytoplasmic pattern recognition receptor signaling pathway | 6/155 | 146/18870 | 0.041096 | 5.003093 | 4.418941 | 0.001315 | 0.011864 | 0.00872 | TLR4/TNFAIP3/NLRP3/PELI1/NFKBIA/CARD8 | 6 |  |
| BP | GO:1990868 | response to chemokine | 5/155 | 99/18870 | 0.050505 | 6.148583 | 4.674196 | 0.001348 | 0.012092 | 0.008888 | CCL20/CCL4/CXCL1/CCR5/CCRL2 | 5 |  |
| BP | GO:1990869 | cellular response to chemokine | 5/155 | 99/18870 | 0.050505 | 6.148583 | 4.674196 | 0.001348 | 0.012092 | 0.008888 | CCL20/CCL4/CXCL1/CCR5/CCRL2 | 5 |  |
| BP | GO:0062013 | positive regulation of small molecule metabolic process | 6/155 | 147/18870 | 0.040816 | 4.969059 | 4.396467 | 0.001361 | 0.012172 | 0.008947 | IL1B/IRS2/NR4A3/PMAIP1/PTAFR/ADM | 6 |  |
| BP | GO:0032753 | positive regulation of interleukin-4 production | 3/155 | 27/18870 | 0.111111 | 13.52688 | 5.927823 | 0.001376 | 0.012263 | 0.009014 | FCER1G/NLRP3/CEBPB | 3 |  |
| BP | GO:0044403 | biological process involved in symbiotic interaction | 9/155 | 322/18870 | 0.02795 | 3.402725 | 3.957572 | 0.001398 | 0.012427 | 0.009134 | CD55/FCN1/TREM1/ANPEP/CCL4/CH25H/ICAM1/PTX3/CCR5 | 9 |  |
| BP | GO:0120162 | positive regulation of cold-induced thermogenesis | 5/155 | 101/18870 | 0.049505 | 6.026828 | 4.609773 | 0.001474 | 0.013055 | 0.009596 | ACSL1/CEBPB/G0S2/PDGFC/GJA1 | 5 |  |
| BP | GO:1903901 | negative regulation of viral life cycle | 3/155 | 28/18870 | 0.107143 | 13.04378 | 5.80395 | 0.001532 | 0.013527 | 0.009943 | FCN1/CH25H/PTX3 | 3 |  |
| BP | GO:0000302 | response to reactive oxygen species | 7/155 | 205/18870 | 0.034146 | 4.157042 | 4.136077 | 0.001547 | 0.013623 | 0.010014 | FOS/MMP9/ADAM9/TNFAIP3/AREG/CYP1B1/LRRK2 | 7 |  |
| BP | GO:0043122 | regulation of canonical NF-kappaB signal transduction | 8/155 | 265/18870 | 0.030189 | 3.675228 | 3.991299 | 0.001587 | 0.013933 | 0.010242 | S100A12/CLEC4D/IL1B/TLR4/TNFAIP3/PELI1/CARD8/GJA1 | 8 |  |
| BP | GO:0032642 | regulation of chemokine production | 5/155 | 104/18870 | 0.048077 | 5.852978 | 4.516318 | 0.001678 | 0.014683 | 0.010793 | TLR2/IL1B/TLR4/EGR1/AIF1 | 5 |  |
| BP | GO:0035872 | nucleotide-binding domain, leucine rich repeat containing receptor signaling pathway | 3/155 | 29/18870 | 0.103448 | 12.59399 | 5.686243 | 0.001698 | 0.014772 | 0.010859 | TLR4/TNFAIP3/NFKBIA | 3 |  |
| BP | GO:0036037 | CD8-positive, alpha-beta T cell activation | 3/155 | 29/18870 | 0.103448 | 12.59399 | 5.686243 | 0.001698 | 0.014772 | 0.010859 | EOMES/CRTAM/CLEC4A | 3 |  |
| BP | GO:0042102 | positive regulation of T cell proliferation | 5/155 | 105/18870 | 0.047619 | 5.797235 | 4.485974 | 0.00175 | 0.015179 | 0.011157 | CD55/IL1B/LILRB2/CD1D/AIF1 | 5 |  |
| BP | GO:0006979 | response to oxidative stress | 10/155 | 400/18870 | 0.025 | 3.043548 | 3.759472 | 0.001763 | 0.015245 | 0.011206 | NR4A2/FOS/MMP9/ADAM9/VNN1/TNFAIP3/AREG/CYP1B1/LRRK2/AIF1 | 10 |  |
| BP | GO:0019722 | calcium-mediated signaling | 7/155 | 210/18870 | 0.033333 | 4.058065 | 4.055513 | 0.001775 | 0.015302 | 0.011248 | BST1/CCL20/CCL4/CCR5/CCRL2/NCALD/LRRK2 | 7 |  |
| BP | GO:0032623 | interleukin-2 production | 4/155 | 63/18870 | 0.063492 | 7.729647 | 4.869094 | 0.00179 | 0.015346 | 0.01128 | ZFP36/IL1B/TNFAIP3/CD83 | 4 |  |
| BP | GO:0032663 | regulation of interleukin-2 production | 4/155 | 63/18870 | 0.063492 | 7.729647 | 4.869094 | 0.00179 | 0.015346 | 0.01128 | ZFP36/IL1B/TNFAIP3/CD83 | 4 |  |
| BP | GO:0051701 | biological process involved in interaction with host | 7/155 | 211/18870 | 0.033175 | 4.038832 | 4.0397 | 0.001823 | 0.015547 | 0.011428 | CD55/FCN1/ANPEP/CH25H/ICAM1/PTX3/CCR5 | 7 |  |
| BP | GO:0030316 | osteoclast differentiation | 5/155 | 106/18870 | 0.04717 | 5.742544 | 4.456018 | 0.001825 | 0.015547 | 0.011428 | FOS/TLR4/CEBPB/TYROBP/TOB2 | 5 |  |
| BP | GO:1905039 | carboxylic acid transmembrane transport | 6/155 | 156/18870 | 0.038462 | 4.682382 | 4.20294 | 0.001842 | 0.01565 | 0.011503 | ACSL1/IRS2/SLC7A7/THBS1/RGS2/SLC22A4 | 6 |  |
| BP | GO:0045619 | regulation of lymphocyte differentiation | 7/155 | 212/18870 | 0.033019 | 4.019781 | 4.023984 | 0.001873 | 0.015748 | 0.011575 | VNN1/LILRB2/NLRP3/NFKBIZ/CRTAM/CD83/EGR3 | 7 |  |
| BP | GO:0098739 | import across plasma membrane | 7/155 | 212/18870 | 0.033019 | 4.019781 | 4.023984 | 0.001873 | 0.015748 | 0.011575 | ACSL1/IRS2/THBS1/SLC2A3/KCNJ2/RGS2/SLC22A4 | 7 |  |
| BP | GO:0043032 | positive regulation of macrophage activation | 3/155 | 30/18870 | 0.1 | 12.17419 | 5.57419 | 0.001875 | 0.015748 | 0.011575 | TLR4/THBS1/LRRK2 | 3 |  |
| BP | GO:0032615 | interleukin-12 production | 4/155 | 64/18870 | 0.0625 | 7.608871 | 4.819638 | 0.001898 | 0.015748 | 0.011575 | TLR2/TLR4/THBS1/LILRA5 | 4 |  |
| BP | GO:0032655 | regulation of interleukin-12 production | 4/155 | 64/18870 | 0.0625 | 7.608871 | 4.819638 | 0.001898 | 0.015748 | 0.011575 | TLR2/TLR4/THBS1/LILRA5 | 4 |  |
| BP | GO:0048247 | lymphocyte chemotaxis | 4/155 | 64/18870 | 0.0625 | 7.608871 | 4.819638 | 0.001898 | 0.015748 | 0.011575 | CCL20/CXCL16/CCL4/CH25H | 4 |  |
| BP | GO:0002526 | acute inflammatory response | 5/155 | 107/18870 | 0.046729 | 5.688875 | 4.426442 | 0.001902 | 0.015748 | 0.011575 | IL1B/VNN1/CD163/NLRP3/CEBPB | 5 |  |
| BP | GO:0007173 | epidermal growth factor receptor signaling pathway | 5/155 | 107/18870 | 0.046729 | 5.688875 | 4.426442 | 0.001902 | 0.015748 | 0.011575 | MMP9/EFEMP1/HBEGF/AREG/PLAUR | 5 |  |
| BP | GO:1903825 | organic acid transmembrane transport | 6/155 | 157/18870 | 0.038217 | 4.652558 | 4.182352 | 0.001903 | 0.015748 | 0.011575 | ACSL1/IRS2/SLC7A7/THBS1/RGS2/SLC22A4 | 6 |  |
| BP | GO:0042063 | gliogenesis | 9/155 | 338/18870 | 0.026627 | 3.241649 | 3.784515 | 0.001948 | 0.016076 | 0.011817 | TLR2/IL1B/C5AR1/S100A9/TLR4/IFNGR1/EOMES/S100A8/AREG | 9 |  |
| BP | GO:2000117 | negative regulation of cysteine-type endopeptidase activity | 4/155 | 65/18870 | 0.061538 | 7.491811 | 4.77124 | 0.002009 | 0.016538 | 0.012156 | MMP9/THBS1/PLAUR/CARD8 | 4 |  |
| BP | GO:0008643 | carbohydrate transport | 6/155 | 159/18870 | 0.037736 | 4.594035 | 4.141692 | 0.002028 | 0.016643 | 0.012233 | IL1B/AQP9/IRS2/NR4A3/SLC2A3/ITLN1 | 6 |  |
| BP | GO:0018108 | peptidyl-tyrosine phosphorylation | 8/155 | 276/18870 | 0.028986 | 3.528752 | 3.85141 | 0.002047 | 0.01675 | 0.012312 | BST1/EFEMP1/HBEGF/LILRA5/AREG/HCK/PDGFC/FGR | 8 |  |
| BP | GO:0018212 | peptidyl-tyrosine modification | 8/155 | 278/18870 | 0.028777 | 3.503365 | 3.82674 | 0.002141 | 0.017421 | 0.012805 | BST1/EFEMP1/HBEGF/LILRA5/AREG/HCK/PDGFC/FGR | 8 |  |
| BP | GO:0008637 | apoptotic mitochondrial changes | 5/155 | 110/18870 | 0.045455 | 5.533724 | 4.339907 | 0.002147 | 0.017421 | 0.012805 | MMP9/IER3/PMAIP1/BCL2A1/PLAUR | 5 |  |
| BP | GO:0014812 | muscle cell migration | 5/155 | 110/18870 | 0.045455 | 5.533724 | 4.339907 | 0.002147 | 0.017421 | 0.012805 | NR4A3/TLR4/DDIT3/CYP1B1/AIF1 | 5 |  |
| BP | GO:0050679 | positive regulation of epithelial cell proliferation | 7/155 | 218/18870 | 0.03211 | 3.909145 | 3.931663 | 0.002192 | 0.01774 | 0.01304 | IRS2/C5AR1/NR4A3/LRG1/TNFAIP3/AREG/EGR3 | 7 |  |
| BP | GO:0031341 | regulation of cell killing | 5/155 | 111/18870 | 0.045045 | 5.483871 | 4.311765 | 0.002233 | 0.017973 | 0.013211 | CD55/ICAM1/TYROBP/CRTAM/CD1D | 5 |  |
| BP | GO:0043280 | positive regulation of cysteine-type endopeptidase activity involved in apoptotic process | 5/155 | 111/18870 | 0.045045 | 5.483871 | 4.311765 | 0.002233 | 0.017973 | 0.013211 | S100A9/PMAIP1/S100A8/NLRP3/CARD8 | 5 |  |
| BP | GO:1901889 | negative regulation of cell junction assembly | 3/155 | 32/18870 | 0.09375 | 11.41331 | 5.36527 | 0.002264 | 0.018167 | 0.013354 | TLR2/IL1B/THBS1 | 3 |  |
| BP | GO:0030193 | regulation of blood coagulation | 4/155 | 68/18870 | 0.058824 | 7.16129 | 4.63201 | 0.002372 | 0.018979 | 0.013951 | THBD/THBS1/PLAUR/SERPINB2 | 4 |  |
| BP | GO:1901222 | regulation of non-canonical NF-kappaB signal transduction | 5/155 | 113/18870 | 0.044248 | 5.386811 | 4.256492 | 0.002414 | 0.019267 | 0.014163 | TLR2/IL1B/TLR4/NLRP3/NFKBIA | 5 |  |
| BP | GO:0033238 | regulation of amine metabolic process | 3/155 | 33/18870 | 0.090909 | 11.06745 | 5.267638 | 0.002475 | 0.019593 | 0.014402 | NR4A2/SLC7A7/ALDH2 | 3 |  |
| BP | GO:0050869 | negative regulation of B cell activation | 3/155 | 33/18870 | 0.090909 | 11.06745 | 5.267638 | 0.002475 | 0.019593 | 0.014402 | TNFAIP3/TYROBP/MNDA | 3 |  |
| BP | GO:1902235 | regulation of endoplasmic reticulum stress-induced intrinsic apoptotic signaling pathway | 3/155 | 33/18870 | 0.090909 | 11.06745 | 5.267638 | 0.002475 | 0.019593 | 0.014402 | PMAIP1/DDIT3/LRRK2 | 3 |  |
| BP | GO:0002711 | positive regulation of T cell mediated immunity | 4/155 | 69/18870 | 0.057971 | 7.057504 | 4.587469 | 0.002502 | 0.01964 | 0.014437 | CD55/IL1B/NLRP3/CD1D | 4 |  |
| BP | GO:0045576 | mast cell activation | 4/155 | 69/18870 | 0.057971 | 7.057504 | 4.587469 | 0.002502 | 0.01964 | 0.014437 | S100A12/NR4A3/CD300LF/FGR | 4 |  |
| BP | GO:1901224 | positive regulation of non-canonical NF-kappaB signal transduction | 4/155 | 69/18870 | 0.057971 | 7.057504 | 4.587469 | 0.002502 | 0.01964 | 0.014437 | TLR2/IL1B/TLR4/NLRP3 | 4 |  |
| BP | GO:0044344 | cellular response to fibroblast growth factor stimulus | 5/155 | 115/18870 | 0.043478 | 5.293128 | 4.202518 | 0.002606 | 0.020401 | 0.014996 | ZFP36/THBS1/SULF2/EGR3/SULF1 | 5 |  |
| BP | GO:1900046 | regulation of hemostasis | 4/155 | 70/18870 | 0.057143 | 6.956682 | 4.543807 | 0.002637 | 0.020542 | 0.0151 | THBD/THBS1/PLAUR/SERPINB2 | 4 |  |
| BP | GO:0031667 | response to nutrient levels | 11/155 | 495/18870 | 0.022222 | 2.705376 | 3.499085 | 0.002643 | 0.020542 | 0.0151 | ACSL1/ZFP36/FOS/PMAIP1/DDIT3/ADM/CDKN1A/NFKBIZ/CYP1B1/GLUL/LRRK2 | 11 |  |
| BP | GO:0042060 | wound healing | 10/155 | 423/18870 | 0.023641 | 2.87806 | 3.555184 | 0.002645 | 0.020542 | 0.0151 | THBD/FCER1G/TLR4/THBS1/LRG1/HBEGF/TNFAIP3/CDKN1A/PLAUR/SERPINB2 | 10 |  |
| BP | GO:1903532 | positive regulation of secretion by cell | 8/155 | 288/18870 | 0.027778 | 3.38172 | 3.706689 | 0.002662 | 0.020617 | 0.015154 | TLR2/IL1B/IRS2/TLR4/ADAM9/PTAFR/CD33/FGR | 8 |  |
| BP | GO:0043372 | positive regulation of CD4-positive, alpha-beta T cell differentiation | 3/155 | 34/18870 | 0.088235 | 10.74194 | 5.174111 | 0.002699 | 0.020787 | 0.01528 | NLRP3/NFKBIZ/CD83 | 3 |  |
| BP | GO:0015908 | fatty acid transport | 5/155 | 116/18870 | 0.043103 | 5.247497 | 4.176 | 0.002705 | 0.020787 | 0.01528 | ACSL1/IL1B/IRS2/RBP7/THBS1 | 5 |  |
| BP | GO:0046634 | regulation of alpha-beta T cell activation | 5/155 | 116/18870 | 0.043103 | 5.247497 | 4.176 | 0.002705 | 0.020787 | 0.01528 | CD55/NLRP3/NFKBIZ/CRTAM/CD83 | 5 |  |
| BP | GO:0002548 | monocyte chemotaxis | 4/155 | 71/18870 | 0.056338 | 6.858701 | 4.500994 | 0.002776 | 0.021278 | 0.015641 | S100A12/CCL20/CCL4/AIF1 | 4 |  |
| BP | GO:1904659 | D-glucose transmembrane transport | 5/155 | 117/18870 | 0.042735 | 5.202647 | 4.149787 | 0.002808 | 0.02146 | 0.015775 | IL1B/IRS2/NR4A3/SLC2A3/ITLN1 | 5 |  |
| BP | GO:0070339 | response to bacterial lipopeptide | 2/155 | 10/18870 | 0.2 | 24.34839 | 6.720962 | 0.002889 | 0.021795 | 0.01602 | TLR2/CD14 | 2 |  |
| BP | GO:0070432 | regulation of nucleotide-binding oligomerization domain containing 2 signaling pathway | 2/155 | 10/18870 | 0.2 | 24.34839 | 6.720962 | 0.002889 | 0.021795 | 0.01602 | TLR4/TNFAIP3 | 2 |  |
| BP | GO:0071220 | cellular response to bacterial lipoprotein | 2/155 | 10/18870 | 0.2 | 24.34839 | 6.720962 | 0.002889 | 0.021795 | 0.01602 | TLR2/CD14 | 2 |  |
| BP | GO:0071221 | cellular response to bacterial lipopeptide | 2/155 | 10/18870 | 0.2 | 24.34839 | 6.720962 | 0.002889 | 0.021795 | 0.01602 | TLR2/CD14 | 2 |  |
| BP | GO:1902237 | positive regulation of endoplasmic reticulum stress-induced intrinsic apoptotic signaling pathway | 2/155 | 10/18870 | 0.2 | 24.34839 | 6.720962 | 0.002889 | 0.021795 | 0.01602 | PMAIP1/DDIT3 | 2 |  |
| BP | GO:0032481 | positive regulation of type I interferon production | 4/155 | 72/18870 | 0.055556 | 6.763441 | 4.459002 | 0.002921 | 0.021795 | 0.01602 | TLR2/TLR4/CD14/ARRDC4 | 4 |  |
| BP | GO:0042130 | negative regulation of T cell proliferation | 4/155 | 72/18870 | 0.055556 | 6.763441 | 4.459002 | 0.002921 | 0.021795 | 0.01602 | LILRB2/CEBPB/PELI1/CRTAM | 4 |  |
| BP | GO:0046635 | positive regulation of alpha-beta T cell activation | 4/155 | 72/18870 | 0.055556 | 6.763441 | 4.459002 | 0.002921 | 0.021795 | 0.01602 | CD55/NLRP3/NFKBIZ/CD83 | 4 |  |
| BP | GO:0071260 | cellular response to mechanical stimulus | 4/155 | 72/18870 | 0.055556 | 6.763441 | 4.459002 | 0.002921 | 0.021795 | 0.01602 | GADD45A/IL1B/TLR4/KCNJ2 | 4 |  |
| BP | GO:0002446 | neutrophil mediated immunity | 3/155 | 35/18870 | 0.085714 | 10.43502 | 5.084398 | 0.002934 | 0.021795 | 0.01602 | TREM1/ANXA3/PTAFR | 3 |  |
| BP | GO:0043392 | negative regulation of DNA binding | 3/155 | 35/18870 | 0.085714 | 10.43502 | 5.084398 | 0.002934 | 0.021795 | 0.01602 | DDIT3/GZMA/NFKBIA | 3 |  |
| BP | GO:0030099 | myeloid cell differentiation | 10/155 | 430/18870 | 0.023256 | 2.831208 | 3.495721 | 0.002974 | 0.021981 | 0.016157 | ZFP36/TLR2/FOS/MMP9/CSF3R/TLR4/CEBPB/TYROBP/TOB2/NFKBIA | 10 |  |
| BP | GO:0043434 | response to peptide hormone | 10/155 | 430/18870 | 0.023256 | 2.831208 | 3.495721 | 0.002974 | 0.021981 | 0.016157 | NR4A2/TLR2/IL1B/FOS/IRS2/NR4A3/AREG/ADM/CYP1B1/EGR1 | 10 |  |
| BP | GO:0032609 | type II interferon production | 5/155 | 119/18870 | 0.042017 | 5.115207 | 4.098249 | 0.003021 | 0.022154 | 0.016285 | IL1B/TLR4/DDIT3/CD14/CRTAM | 5 |  |
| BP | GO:0032649 | regulation of type II interferon production | 5/155 | 119/18870 | 0.042017 | 5.115207 | 4.098249 | 0.003021 | 0.022154 | 0.016285 | IL1B/TLR4/DDIT3/CD14/CRTAM | 5 |  |
| BP | GO:0035710 | CD4-positive, alpha-beta T cell activation | 5/155 | 119/18870 | 0.042017 | 5.115207 | 4.098249 | 0.003021 | 0.022154 | 0.016285 | CD55/RORC/NLRP3/NFKBIZ/CD83 | 5 |  |
| BP | GO:0006809 | nitric oxide biosynthetic process | 4/155 | 73/18870 | 0.054795 | 6.670791 | 4.417801 | 0.003071 | 0.022314 | 0.016403 | IL1B/TLR4/PTX3/CYP1B1 | 4 |  |
| BP | GO:0050818 | regulation of coagulation | 4/155 | 73/18870 | 0.054795 | 6.670791 | 4.417801 | 0.003071 | 0.022314 | 0.016403 | THBD/THBS1/PLAUR/SERPINB2 | 4 |  |
| BP | GO:0072678 | T cell migration | 4/155 | 73/18870 | 0.054795 | 6.670791 | 4.417801 | 0.003071 | 0.022314 | 0.016403 | CCL20/CXCL16/ICAM1/AIF1 | 4 |  |
| BP | GO:0016032 | viral process | 10/155 | 432/18870 | 0.023148 | 2.8181 | 3.47895 | 0.003074 | 0.022314 | 0.016403 | CD55/ZFP36/FCN1/ANPEP/CCL4/CH25H/ICAM1/PTX3/CCR5/ST6GAL1 | 10 |  |
| BP | GO:0010632 | regulation of epithelial cell migration | 8/155 | 295/18870 | 0.027119 | 3.301476 | 3.625755 | 0.003082 | 0.022314 | 0.016403 | GADD45A/IRS2/MMP9/THBS1/ADAM9/ANXA3/HBEGF/GLUL | 8 |  |
| BP | GO:1903034 | regulation of response to wounding | 6/155 | 173/18870 | 0.034682 | 4.222264 | 3.874749 | 0.003089 | 0.022314 | 0.016403 | THBD/THBS1/HBEGF/TNFAIP3/PLAUR/SERPINB2 | 6 |  |
| BP | GO:0002718 | regulation of cytokine production involved in immune response | 5/155 | 120/18870 | 0.041667 | 5.072581 | 4.072912 | 0.003132 | 0.022511 | 0.016547 | CD55/IL1B/NR4A3/TLR4/NLRP3 | 5 |  |
| BP | GO:0008645 | hexose transmembrane transport | 5/155 | 120/18870 | 0.041667 | 5.072581 | 4.072912 | 0.003132 | 0.022511 | 0.016547 | IL1B/IRS2/NR4A3/SLC2A3/ITLN1 | 5 |  |
| BP | GO:0009410 | response to xenobiotic stimulus | 10/155 | 434/18870 | 0.023041 | 2.805114 | 3.462274 | 0.003177 | 0.022779 | 0.016744 | ACSL1/S100A12/IL1B/FOS/THBS1/RORC/FOSB/CDKN1A/NFKBIZ/CYP1B1 | 10 |  |
| BP | GO:0002224 | toll-like receptor signaling pathway | 4/155 | 74/18870 | 0.054054 | 6.580645 | 4.377367 | 0.003226 | 0.023076 | 0.016963 | TLR2/TLR4/NFKBIZ/CD300LF | 4 |  |
| BP | GO:0045471 | response to ethanol | 5/155 | 121/18870 | 0.041322 | 5.030658 | 4.047856 | 0.003245 | 0.023099 | 0.01698 | FOS/CD14/S100A8/ST6GAL1/RGS2 | 5 |  |
| BP | GO:0051100 | negative regulation of binding | 5/155 | 121/18870 | 0.041322 | 5.030658 | 4.047856 | 0.003245 | 0.023099 | 0.01698 | DDIT3/CDKN1A/GZMA/NFKBIA/LRRK2 | 5 |  |
| BP | GO:0009615 | response to virus | 10/155 | 436/18870 | 0.022936 | 2.792246 | 3.445692 | 0.003282 | 0.023303 | 0.017129 | TLR2/IL1B/IFNGR1/PMAIP1/CCL4/TNFAIP3/NLRP3/RNASE2/FGR/CARD8 | 10 |  |
| BP | GO:0002573 | myeloid leukocyte differentiation | 7/155 | 235/18870 | 0.029787 | 3.626356 | 3.686957 | 0.003329 | 0.023579 | 0.017332 | TLR2/FOS/MMP9/TLR4/CEBPB/TYROBP/TOB2 | 7 |  |
| BP | GO:0010634 | positive regulation of epithelial cell migration | 6/155 | 176/18870 | 0.034091 | 4.150293 | 3.821216 | 0.003362 | 0.023697 | 0.017419 | IRS2/MMP9/THBS1/ADAM9/ANXA3/HBEGF | 6 |  |
| BP | GO:0022612 | gland morphogenesis | 5/155 | 122/18870 | 0.040984 | 4.989424 | 4.023074 | 0.003362 | 0.023697 | 0.017419 | SULF2/TNFAIP3/AREG/CEBPB/SULF1 | 5 |  |
| BP | GO:0061045 | negative regulation of wound healing | 4/155 | 75/18870 | 0.053333 | 6.492903 | 4.337673 | 0.003386 | 0.023717 | 0.017433 | THBD/THBS1/PLAUR/SERPINB2 | 4 |  |
| BP | GO:0002449 | lymphocyte mediated immunity | 9/155 | 368/18870 | 0.024457 | 2.977384 | 3.48619 | 0.003435 | 0.023717 | 0.017433 | CD55/IL1B/FCER1G/NLRP3/ICAM1/NFKBIZ/CRTAM/CSF2RB/CD1D | 9 |  |
| BP | GO:0044546 | NLRP3 inflammasome complex assembly | 3/155 | 37/18870 | 0.081081 | 9.870968 | 4.915384 | 0.003442 | 0.023717 | 0.017433 | TLR4/NLRP3/CARD8 | 3 |  |
| BP | GO:0051385 | response to mineralocorticoid | 3/155 | 37/18870 | 0.081081 | 9.870968 | 4.915384 | 0.003442 | 0.023717 | 0.017433 | FOS/FOSB/CDKN1A | 3 |  |
| BP | GO:0015749 | monosaccharide transmembrane transport | 5/155 | 123/18870 | 0.04065 | 4.948859 | 3.998561 | 0.003482 | 0.023717 | 0.017433 | IL1B/IRS2/NR4A3/SLC2A3/ITLN1 | 5 |  |
| BP | GO:0032479 | regulation of type I interferon production | 5/155 | 123/18870 | 0.04065 | 4.948859 | 3.998561 | 0.003482 | 0.023717 | 0.017433 | TLR2/TLR4/CD14/TYROBP/ARRDC4 | 5 |  |
| BP | GO:0032606 | type I interferon production | 5/155 | 123/18870 | 0.04065 | 4.948859 | 3.998561 | 0.003482 | 0.023717 | 0.017433 | TLR2/TLR4/CD14/TYROBP/ARRDC4 | 5 |  |
| BP | GO:0071774 | response to fibroblast growth factor | 5/155 | 123/18870 | 0.04065 | 4.948859 | 3.998561 | 0.003482 | 0.023717 | 0.017433 | ZFP36/THBS1/SULF2/EGR3/SULF1 | 5 |  |
| BP | GO:0002765 | immune response-inhibiting signal transduction | 2/155 | 11/18870 | 0.181818 | 22.1349 | 6.380909 | 0.003512 | 0.023717 | 0.017433 | LILRB2/CD33 | 2 |  |
| BP | GO:0032493 | response to bacterial lipoprotein | 2/155 | 11/18870 | 0.181818 | 22.1349 | 6.380909 | 0.003512 | 0.023717 | 0.017433 | TLR2/CD14 | 2 |  |
| BP | GO:0032836 | glomerular basement membrane development | 2/155 | 11/18870 | 0.181818 | 22.1349 | 6.380909 | 0.003512 | 0.023717 | 0.017433 | SULF2/SULF1 | 2 |  |
| BP | GO:0033089 | positive regulation of T cell differentiation in thymus | 2/155 | 11/18870 | 0.181818 | 22.1349 | 6.380909 | 0.003512 | 0.023717 | 0.017433 | VNN1/EGR3 | 2 |  |
| BP | GO:0034139 | regulation of toll-like receptor 3 signaling pathway | 2/155 | 11/18870 | 0.181818 | 22.1349 | 6.380909 | 0.003512 | 0.023717 | 0.017433 | TNFAIP3/PELI1 | 2 |  |
| BP | GO:0035666 | TRIF-dependent toll-like receptor signaling pathway | 2/155 | 11/18870 | 0.181818 | 22.1349 | 6.380909 | 0.003512 | 0.023717 | 0.017433 | TLR4/CD300LF | 2 |  |
| BP | GO:0071492 | cellular response to UV-A | 2/155 | 11/18870 | 0.181818 | 22.1349 | 6.380909 | 0.003512 | 0.023717 | 0.017433 | MMP9/MME | 2 |  |
| BP | GO:1904464 | regulation of matrix metallopeptidase secretion | 2/155 | 11/18870 | 0.181818 | 22.1349 | 6.380909 | 0.003512 | 0.023717 | 0.017433 | TLR2/TLR4 | 2 |  |
| BP | GO:1990773 | matrix metallopeptidase secretion | 2/155 | 11/18870 | 0.181818 | 22.1349 | 6.380909 | 0.003512 | 0.023717 | 0.017433 | TLR2/TLR4 | 2 |  |
| BP | GO:2000109 | regulation of macrophage apoptotic process | 2/155 | 11/18870 | 0.181818 | 22.1349 | 6.380909 | 0.003512 | 0.023717 | 0.017433 | CCR5/ST6GAL1 | 2 |  |
| BP | GO:0002287 | alpha-beta T cell activation involved in immune response | 4/155 | 76/18870 | 0.052632 | 6.40747 | 4.298696 | 0.003552 | 0.023875 | 0.01755 | EOMES/RORC/NLRP3/NFKBIZ | 4 |  |
| BP | GO:0002293 | alpha-beta T cell differentiation involved in immune response | 4/155 | 76/18870 | 0.052632 | 6.40747 | 4.298696 | 0.003552 | 0.023875 | 0.01755 | EOMES/RORC/NLRP3/NFKBIZ | 4 |  |
| BP | GO:0002761 | regulation of myeloid leukocyte differentiation | 5/155 | 124/18870 | 0.040323 | 4.908949 | 3.974312 | 0.003604 | 0.024004 | 0.017644 | FOS/TLR4/CEBPB/TYROBP/TOB2 | 5 |  |
| BP | GO:0002824 | positive regulation of adaptive immune response based on somatic recombination of immune receptors built from immunoglobulin superfamily domains | 5/155 | 124/18870 | 0.040323 | 4.908949 | 3.974312 | 0.003604 | 0.024004 | 0.017644 | CD55/IL1B/NLRP3/NFKBIZ/CD1D | 5 |  |
| BP | GO:0038127 | ERBB signaling pathway | 5/155 | 124/18870 | 0.040323 | 4.908949 | 3.974312 | 0.003604 | 0.024004 | 0.017644 | MMP9/EFEMP1/HBEGF/AREG/PLAUR | 5 |  |
| BP | GO:0046632 | alpha-beta T cell differentiation | 5/155 | 124/18870 | 0.040323 | 4.908949 | 3.974312 | 0.003604 | 0.024004 | 0.017644 | EOMES/RORC/NLRP3/NFKBIZ/CD83 | 5 |  |
| BP | GO:0015718 | monocarboxylic acid transport | 6/155 | 179/18870 | 0.03352 | 4.080735 | 3.76886 | 0.003652 | 0.024267 | 0.017838 | ACSL1/IL1B/AQP9/IRS2/RBP7/THBS1 | 6 |  |
| BP | GO:0002369 | T cell cytokine production | 3/155 | 38/18870 | 0.078947 | 9.611205 | 4.835628 | 0.003714 | 0.024448 | 0.017971 | CD55/IL1B/NLRP3 | 3 |  |
| BP | GO:0002724 | regulation of T cell cytokine production | 3/155 | 38/18870 | 0.078947 | 9.611205 | 4.835628 | 0.003714 | 0.024448 | 0.017971 | CD55/IL1B/NLRP3 | 3 |  |
| BP | GO:0040036 | regulation of fibroblast growth factor receptor signaling pathway | 3/155 | 38/18870 | 0.078947 | 9.611205 | 4.835628 | 0.003714 | 0.024448 | 0.017971 | THBS1/SULF2/SULF1 | 3 |  |
| BP | GO:0045429 | positive regulation of nitric oxide biosynthetic process | 3/155 | 38/18870 | 0.078947 | 9.611205 | 4.835628 | 0.003714 | 0.024448 | 0.017971 | IL1B/TLR4/PTX3 | 3 |  |
| BP | GO:0032729 | positive regulation of type II interferon production | 4/155 | 77/18870 | 0.051948 | 6.324256 | 4.260412 | 0.003723 | 0.024448 | 0.017971 | IL1B/TLR4/CD14/CRTAM | 4 |  |
| BP | GO:0071675 | regulation of mononuclear cell migration | 5/155 | 125/18870 | 0.04 | 4.869677 | 3.950321 | 0.00373 | 0.024448 | 0.017971 | CCL20/C5AR1/THBS1/CCL4/AIF1 | 5 |  |
| BP | GO:1901653 | cellular response to peptide | 9/155 | 374/18870 | 0.024064 | 2.929619 | 3.430155 | 0.003819 | 0.024972 | 0.018356 | NR4A2/IL1B/FOS/IRS2/NR4A3/TLR4/ICAM1/CYP1B1/GJA1 | 9 |  |
| BP | GO:0002688 | regulation of leukocyte chemotaxis | 5/155 | 126/18870 | 0.039683 | 4.831029 | 3.926585 | 0.003859 | 0.025179 | 0.018508 | BST1/C5AR1/THBS1/CCL4/AIF1 | 5 |  |
| BP | GO:0010827 | regulation of D-glucose transmembrane transport | 4/155 | 78/18870 | 0.051282 | 6.243176 | 4.222801 | 0.0039 | 0.025273 | 0.018577 | IL1B/IRS2/NR4A3/ITLN1 | 4 |  |
| BP | GO:0032720 | negative regulation of tumor necrosis factor production | 4/155 | 78/18870 | 0.051282 | 6.243176 | 4.222801 | 0.0039 | 0.025273 | 0.018577 | TLR4/TNFAIP3/CD33/CLEC4A | 4 |  |
| BP | GO:2000514 | regulation of CD4-positive, alpha-beta T cell activation | 4/155 | 78/18870 | 0.051282 | 6.243176 | 4.222801 | 0.0039 | 0.025273 | 0.018577 | CD55/NLRP3/NFKBIZ/CD83 | 4 |  |
| BP | GO:0097242 | amyloid-beta clearance | 3/155 | 39/18870 | 0.076923 | 9.364764 | 4.75877 | 0.004 | 0.025751 | 0.018928 | C5AR1/IFNGR1/MME | 3 |  |
| BP | GO:0140632 | canonical inflammasome complex assembly | 3/155 | 39/18870 | 0.076923 | 9.364764 | 4.75877 | 0.004 | 0.025751 | 0.018928 | TLR4/NLRP3/CARD8 | 3 |  |
| BP | GO:1904407 | positive regulation of nitric oxide metabolic process | 3/155 | 39/18870 | 0.076923 | 9.364764 | 4.75877 | 0.004 | 0.025751 | 0.018928 | IL1B/TLR4/PTX3 | 3 |  |
| BP | GO:0060759 | regulation of response to cytokine stimulus | 6/155 | 183/18870 | 0.032787 | 3.991539 | 3.700801 | 0.004068 | 0.026132 | 0.019208 | IL1R2/TLR2/TLR4/TNFAIP3/CD300LF/CARD8 | 6 |  |
| BP | GO:0010951 | negative regulation of endopeptidase activity | 5/155 | 128/18870 | 0.039063 | 4.755544 | 3.879853 | 0.004126 | 0.026447 | 0.01944 | MMP9/THBS1/PLAUR/CARD8/SERPINB2 | 5 |  |
| BP | GO:0014004 | microglia differentiation | 2/155 | 12/18870 | 0.166667 | 20.29032 | 6.083138 | 0.004192 | 0.026573 | 0.019533 | TLR2/TLR4 | 2 |  |
| BP | GO:0042416 | dopamine biosynthetic process | 2/155 | 12/18870 | 0.166667 | 20.29032 | 6.083138 | 0.004192 | 0.026573 | 0.019533 | NR4A2/ALDH2 | 2 |  |
| BP | GO:0043312 | neutrophil degranulation | 2/155 | 12/18870 | 0.166667 | 20.29032 | 6.083138 | 0.004192 | 0.026573 | 0.019533 | ANXA3/PTAFR | 2 |  |
| BP | GO:0071493 | cellular response to UV-B | 2/155 | 12/18870 | 0.166667 | 20.29032 | 6.083138 | 0.004192 | 0.026573 | 0.019533 | CDKN1A/MME | 2 |  |
| BP | GO:2001267 | regulation of cysteine-type endopeptidase activity involved in apoptotic signaling pathway | 2/155 | 12/18870 | 0.166667 | 20.29032 | 6.083138 | 0.004192 | 0.026573 | 0.019533 | MMP9/PLAUR | 2 |  |
| BP | GO:0002708 | positive regulation of lymphocyte mediated immunity | 5/155 | 129/18870 | 0.03876 | 4.71868 | 3.856849 | 0.004265 | 0.026889 | 0.019765 | CD55/IL1B/NLRP3/CRTAM/CD1D | 5 |  |
| BP | GO:2001056 | positive regulation of cysteine-type endopeptidase activity | 5/155 | 129/18870 | 0.03876 | 4.71868 | 3.856849 | 0.004265 | 0.026889 | 0.019765 | S100A9/PMAIP1/S100A8/NLRP3/CARD8 | 5 |  |
| BP | GO:1903556 | negative regulation of tumor necrosis factor superfamily cytokine production | 4/155 | 80/18870 | 0.05 | 6.087097 | 4.149512 | 0.00427 | 0.026889 | 0.019765 | TLR4/TNFAIP3/CD33/CLEC4A | 4 |  |
| BP | GO:0032728 | positive regulation of interferon-beta production | 3/155 | 40/18870 | 0.075 | 9.130645 | 4.684629 | 0.004299 | 0.026954 | 0.019813 | TLR2/TLR4/ARRDC4 | 3 |  |
| BP | GO:0045730 | respiratory burst | 3/155 | 40/18870 | 0.075 | 9.130645 | 4.684629 | 0.004299 | 0.026954 | 0.019813 | CD55/HCK/NCF2 | 3 |  |
| BP | GO:0002821 | positive regulation of adaptive immune response | 5/155 | 130/18870 | 0.038462 | 4.682382 | 3.834079 | 0.004407 | 0.027573 | 0.020268 | CD55/IL1B/NLRP3/NFKBIZ/CD1D | 5 |  |
| BP | GO:0002691 | regulation of cellular extravasation | 3/155 | 41/18870 | 0.073171 | 8.907946 | 4.613042 | 0.00461 | 0.028661 | 0.021067 | BST1/PTAFR/ICAM1 | 3 |  |
| BP | GO:0045923 | positive regulation of fatty acid metabolic process | 3/155 | 41/18870 | 0.073171 | 8.907946 | 4.613042 | 0.00461 | 0.028661 | 0.021067 | IL1B/IRS2/NR4A3 | 3 |  |
| BP | GO:0150077 | regulation of neuroinflammatory response | 3/155 | 41/18870 | 0.073171 | 8.907946 | 4.613042 | 0.00461 | 0.028661 | 0.021067 | IL1B/MMP9/LRRK2 | 3 |  |
| BP | GO:1905897 | regulation of response to endoplasmic reticulum stress | 4/155 | 82/18870 | 0.04878 | 5.938631 | 4.07867 | 0.004663 | 0.028924 | 0.021261 | PPP1R15A/PMAIP1/DDIT3/LRRK2 | 4 |  |
| BP | GO:0032490 | detection of molecule of bacterial origin | 2/155 | 13/18870 | 0.153846 | 18.72953 | 5.819396 | 0.004927 | 0.029974 | 0.022033 | TLR2/TLR4 | 2 |  |
| BP | GO:0034145 | positive regulation of toll-like receptor 4 signaling pathway | 2/155 | 13/18870 | 0.153846 | 18.72953 | 5.819396 | 0.004927 | 0.029974 | 0.022033 | CD14/PELI1 | 2 |  |
| BP | GO:0035589 | G protein-coupled purinergic nucleotide receptor signaling pathway | 2/155 | 13/18870 | 0.153846 | 18.72953 | 5.819396 | 0.004927 | 0.029974 | 0.022033 | PTAFR/P2RY13 | 2 |  |
| BP | GO:0036006 | cellular response to macrophage colony-stimulating factor stimulus | 2/155 | 13/18870 | 0.153846 | 18.72953 | 5.819396 | 0.004927 | 0.029974 | 0.022033 | TLR2/TLR4 | 2 |  |
| BP | GO:0051918 | negative regulation of fibrinolysis | 2/155 | 13/18870 | 0.153846 | 18.72953 | 5.819396 | 0.004927 | 0.029974 | 0.022033 | THBD/THBS1 | 2 |  |
| BP | GO:0060213 | positive regulation of nuclear-transcribed mRNA poly(A) tail shortening | 2/155 | 13/18870 | 0.153846 | 18.72953 | 5.819396 | 0.004927 | 0.029974 | 0.022033 | ZFP36/CNOT1 | 2 |  |
| BP | GO:1902947 | regulation of tau-protein kinase activity | 2/155 | 13/18870 | 0.153846 | 18.72953 | 5.819396 | 0.004927 | 0.029974 | 0.022033 | C5AR1/EGR1 | 2 |  |
| BP | GO:2001198 | regulation of dendritic cell differentiation | 2/155 | 13/18870 | 0.153846 | 18.72953 | 5.819396 | 0.004927 | 0.029974 | 0.022033 | LILRB2/CEBPB | 2 |  |
| BP | GO:0141084 | inflammasome-mediated signaling pathway | 3/155 | 42/18870 | 0.071429 | 8.695853 | 4.543857 | 0.004935 | 0.029974 | 0.022033 | TLR4/NLRP3/CARD8 | 3 |  |
| BP | GO:2000403 | positive regulation of lymphocyte migration | 3/155 | 42/18870 | 0.071429 | 8.695853 | 4.543857 | 0.004935 | 0.029974 | 0.022033 | CCL20/CCL4/AIF1 | 3 |  |
| BP | GO:0031099 | regeneration | 6/155 | 191/18870 | 0.031414 | 3.824354 | 3.570297 | 0.005004 | 0.030327 | 0.022292 | NR4A3/SULF2/ANXA3/ADM/CDKN1A/CEBPB | 6 |  |
| BP | GO:0034599 | cellular response to oxidative stress | 7/155 | 255/18870 | 0.027451 | 3.341935 | 3.426564 | 0.005189 | 0.031381 | 0.023067 | NR4A2/FOS/MMP9/TNFAIP3/CYP1B1/LRRK2/AIF1 | 7 |  |
| BP | GO:0002822 | regulation of adaptive immune response based on somatic recombination of immune receptors built from immunoglobulin superfamily domains | 6/155 | 193/18870 | 0.031088 | 3.784723 | 3.538772 | 0.005261 | 0.031698 | 0.0233 | CD55/IL1B/TNFAIP3/NLRP3/NFKBIZ/CD1D | 6 |  |
| BP | GO:0033003 | regulation of mast cell activation | 3/155 | 43/18870 | 0.069767 | 8.493623 | 4.476936 | 0.005274 | 0.031698 | 0.0233 | NR4A3/CD300LF/FGR | 3 |  |
| BP | GO:1904646 | cellular response to amyloid-beta | 3/155 | 43/18870 | 0.069767 | 8.493623 | 4.476936 | 0.005274 | 0.031698 | 0.0233 | TLR4/ICAM1/GJA1 | 3 |  |
| BP | GO:0002702 | positive regulation of production of molecular mediator of immune response | 5/155 | 136/18870 | 0.036765 | 4.475806 | 3.70216 | 0.005329 | 0.031962 | 0.023494 | CD55/IL1B/NR4A3/TLR4/NLRP3 | 5 |  |
| BP | GO:0008625 | extrinsic apoptotic signaling pathway via death domain receptors | 4/155 | 86/18870 | 0.046512 | 5.662416 | 3.94377 | 0.00552 | 0.03304 | 0.024286 | THBS1/PMAIP1/TNFAIP3/ICAM1 | 4 |  |
| BP | GO:0010828 | positive regulation of D-glucose transmembrane transport | 3/155 | 44/18870 | 0.068182 | 8.300587 | 4.412152 | 0.005626 | 0.033606 | 0.024702 | IRS2/NR4A3/ITLN1 | 3 |  |
| BP | GO:0043123 | positive regulation of canonical NF-kappaB signal transduction | 6/155 | 196/18870 | 0.030612 | 3.726794 | 3.492264 | 0.005664 | 0.033736 | 0.024798 | S100A12/CLEC4D/IL1B/TLR4/PELI1/GJA1 | 6 |  |
| BP | GO:0032908 | regulation of transforming growth factor beta1 production | 2/155 | 14/18870 | 0.142857 | 17.39171 | 5.583529 | 0.005718 | 0.033736 | 0.024798 | THBS1/TYROBP | 2 |  |
| BP | GO:0061469 | regulation of type B pancreatic cell proliferation | 2/155 | 14/18870 | 0.142857 | 17.39171 | 5.583529 | 0.005718 | 0.033736 | 0.024798 | IRS2/NR4A3 | 2 |  |
| BP | GO:0070141 | response to UV-A | 2/155 | 14/18870 | 0.142857 | 17.39171 | 5.583529 | 0.005718 | 0.033736 | 0.024798 | MMP9/MME | 2 |  |
| BP | GO:0071888 | macrophage apoptotic process | 2/155 | 14/18870 | 0.142857 | 17.39171 | 5.583529 | 0.005718 | 0.033736 | 0.024798 | CCR5/ST6GAL1 | 2 |  |
| BP | GO:1902043 | positive regulation of extrinsic apoptotic signaling pathway via death domain receptors | 2/155 | 14/18870 | 0.142857 | 17.39171 | 5.583529 | 0.005718 | 0.033736 | 0.024798 | THBS1/PMAIP1 | 2 |  |
| BP | GO:0090199 | regulation of release of cytochrome c from mitochondria | 3/155 | 45/18870 | 0.066667 | 8.116129 | 4.349386 | 0.005992 | 0.035233 | 0.025899 | MMP9/PMAIP1/PLAUR | 3 |  |
| BP | GO:0051098 | regulation of binding | 7/155 | 262/18870 | 0.026718 | 3.252647 | 3.341484 | 0.005996 | 0.035233 | 0.025899 | MMP9/DDIT3/CDKN1A/GZMA/PLAUR/NFKBIA/LRRK2 | 7 |  |
| BP | GO:0001909 | leukocyte mediated cytotoxicity | 5/155 | 140/18870 | 0.035714 | 4.347926 | 3.618398 | 0.006015 | 0.035273 | 0.025928 | TREM1/ICAM1/TYROBP/CRTAM/CD1D | 5 |  |
| BP | GO:0014910 | regulation of smooth muscle cell migration | 4/155 | 89/18870 | 0.044944 | 5.471548 | 3.848034 | 0.006228 | 0.03645 | 0.026793 | NR4A3/TLR4/CYP1B1/AIF1 | 4 |  |
| BP | GO:1900271 | regulation of long-term synaptic potentiation | 3/155 | 46/18870 | 0.065217 | 7.939691 | 4.28853 | 0.006372 | 0.037217 | 0.027357 | LILRB2/MME/TYROBP | 3 |  |
| BP | GO:0034976 | response to endoplasmic reticulum stress | 7/155 | 266/18870 | 0.026316 | 3.203735 | 3.294143 | 0.006497 | 0.037348 | 0.027453 | PPP1R15A/THBS1/PMAIP1/DDIT3/CEBPB/RNF175/LRRK2 | 7 |  |
| BP | GO:0050728 | negative regulation of inflammatory response | 6/155 | 202/18870 | 0.029703 | 3.616097 | 3.401935 | 0.006537 | 0.037348 | 0.027453 | ZFP36/IER3/TNFAIP3/NLRP3/HCK/FGR | 6 |  |
| BP | GO:0002468 | dendritic cell antigen processing and presentation | 2/155 | 15/18870 | 0.133333 | 16.23226 | 5.370838 | 0.006562 | 0.037348 | 0.027453 | THBS1/CLEC4A | 2 |  |
| BP | GO:0010838 | positive regulation of keratinocyte proliferation | 2/155 | 15/18870 | 0.133333 | 16.23226 | 5.370838 | 0.006562 | 0.037348 | 0.027453 | LRG1/AREG | 2 |  |
| BP | GO:0032352 | positive regulation of hormone metabolic process | 2/155 | 15/18870 | 0.133333 | 16.23226 | 5.370838 | 0.006562 | 0.037348 | 0.027453 | ADM/EGR1 | 2 |  |
| BP | GO:0032725 | positive regulation of granulocyte macrophage colony-stimulating factor production | 2/155 | 15/18870 | 0.133333 | 16.23226 | 5.370838 | 0.006562 | 0.037348 | 0.027453 | IL1B/LILRA2 | 2 |  |
| BP | GO:0034144 | negative regulation of toll-like receptor 4 signaling pathway | 2/155 | 15/18870 | 0.133333 | 16.23226 | 5.370838 | 0.006562 | 0.037348 | 0.027453 | LILRA2/TNFAIP3 | 2 |  |
| BP | GO:0044849 | estrous cycle | 2/155 | 15/18870 | 0.133333 | 16.23226 | 5.370838 | 0.006562 | 0.037348 | 0.027453 | CYP1B1/EGR1 | 2 |  |
| BP | GO:0046321 | positive regulation of fatty acid oxidation | 2/155 | 15/18870 | 0.133333 | 16.23226 | 5.370838 | 0.006562 | 0.037348 | 0.027453 | IRS2/NR4A3 | 2 |  |
| BP | GO:0060211 | regulation of nuclear-transcribed mRNA poly(A) tail shortening | 2/155 | 15/18870 | 0.133333 | 16.23226 | 5.370838 | 0.006562 | 0.037348 | 0.027453 | ZFP36/CNOT1 | 2 |  |
| BP | GO:0070207 | protein homotrimerization | 2/155 | 15/18870 | 0.133333 | 16.23226 | 5.370838 | 0.006562 | 0.037348 | 0.027453 | ITLN1/STEAP4 | 2 |  |
| BP | GO:0070424 | regulation of nucleotide-binding domain, leucine rich repeat containing receptor signaling pathway | 2/155 | 15/18870 | 0.133333 | 16.23226 | 5.370838 | 0.006562 | 0.037348 | 0.027453 | TLR4/TNFAIP3 | 2 |  |
| BP | GO:1900153 | positive regulation of nuclear-transcribed mRNA catabolic process, deadenylation-dependent decay | 2/155 | 15/18870 | 0.133333 | 16.23226 | 5.370838 | 0.006562 | 0.037348 | 0.027453 | ZFP36/CNOT1 | 2 |  |
| BP | GO:0034763 | negative regulation of transmembrane transport | 5/155 | 144/18870 | 0.034722 | 4.227151 | 3.537719 | 0.006761 | 0.038209 | 0.028086 | IL1B/IRS2/MMP9/THBS1/RGS2 | 5 |  |
| BP | GO:0050714 | positive regulation of protein secretion | 5/155 | 144/18870 | 0.034722 | 4.227151 | 3.537719 | 0.006761 | 0.038209 | 0.028086 | TLR2/IRS2/TLR4/ADAM9/CD33 | 5 |  |
| BP | GO:1904019 | epithelial cell apoptotic process | 5/155 | 144/18870 | 0.034722 | 4.227151 | 3.537719 | 0.006761 | 0.038209 | 0.028086 | ZFP36/THBS1/TNFAIP3/ICAM1/NFKBIZ | 5 |  |
| BP | GO:0010837 | regulation of keratinocyte proliferation | 3/155 | 47/18870 | 0.06383 | 7.770762 | 4.229484 | 0.006766 | 0.038209 | 0.028086 | ZFP36/LRG1/AREG | 3 |  |
| BP | GO:0045834 | positive regulation of lipid metabolic process | 5/155 | 145/18870 | 0.034483 | 4.197998 | 3.518006 | 0.006957 | 0.03921 | 0.028822 | IL1B/IRS2/NR4A3/ADM/FGR | 5 |  |
| BP | GO:0031640 | killing of cells of another organism | 4/155 | 92/18870 | 0.043478 | 5.293128 | 3.756543 | 0.006994 | 0.039267 | 0.028864 | S100A12/TREM1/GZMA/LYZ | 4 |  |
| BP | GO:0141061 | disruption of cell in another organism | 4/155 | 92/18870 | 0.043478 | 5.293128 | 3.756543 | 0.006994 | 0.039267 | 0.028864 | S100A12/TREM1/GZMA/LYZ | 4 |  |
| BP | GO:0030177 | positive regulation of Wnt signaling pathway | 5/155 | 146/18870 | 0.034247 | 4.169244 | 3.49847 | 0.007157 | 0.039968 | 0.029379 | TLR2/SULF2/TNFAIP3/LRRK2/SULF1 | 5 |  |
| BP | GO:0045598 | regulation of fat cell differentiation | 5/155 | 146/18870 | 0.034247 | 4.169244 | 3.49847 | 0.007157 | 0.039968 | 0.029379 | ZFP36/JDP2/DDIT3/RORC/CEBPB | 5 |  |
| BP | GO:0006953 | acute-phase response | 3/155 | 48/18870 | 0.0625 | 7.608871 | 4.172154 | 0.007174 | 0.039968 | 0.029379 | IL1B/CD163/CEBPB | 3 |  |
| BP | GO:0042088 | T-helper 1 type immune response | 3/155 | 48/18870 | 0.0625 | 7.608871 | 4.172154 | 0.007174 | 0.039968 | 0.029379 | IL1B/TLR4/NFKBIZ | 3 |  |
| BP | GO:0039531 | regulation of cytoplasmic pattern recognition receptor signaling pathway | 4/155 | 93/18870 | 0.043011 | 5.236212 | 3.726932 | 0.007263 | 0.040152 | 0.029514 | TLR4/TNFAIP3/PELI1/CARD8 | 4 |  |
| BP | GO:0043367 | CD4-positive, alpha-beta T cell differentiation | 4/155 | 93/18870 | 0.043011 | 5.236212 | 3.726932 | 0.007263 | 0.040152 | 0.029514 | RORC/NLRP3/NFKBIZ/CD83 | 4 |  |
| BP | GO:0048525 | negative regulation of viral process | 4/155 | 93/18870 | 0.043011 | 5.236212 | 3.726932 | 0.007263 | 0.040152 | 0.029514 | ZFP36/FCN1/CH25H/PTX3 | 4 |  |
| BP | GO:1904705 | regulation of vascular associated smooth muscle cell proliferation | 4/155 | 93/18870 | 0.043011 | 5.236212 | 3.726932 | 0.007263 | 0.040152 | 0.029514 | MMP9/NR4A3/CDKN1A/GJA1 | 4 |  |
| BP | GO:0019216 | regulation of lipid metabolic process | 8/155 | 342/18870 | 0.023392 | 2.847765 | 3.138274 | 0.007395 | 0.040543 | 0.029801 | IL1B/IRS2/NR4A3/CH25H/RORC/ADM/FGR/EGR1 | 8 |  |
| BP | GO:0002756 | MyD88-independent toll-like receptor signaling pathway | 2/155 | 16/18870 | 0.125 | 15.21774 | 5.177669 | 0.007459 | 0.040543 | 0.029801 | TLR4/CD300LF | 2 |  |
| BP | GO:0016045 | detection of bacterium | 2/155 | 16/18870 | 0.125 | 15.21774 | 5.177669 | 0.007459 | 0.040543 | 0.029801 | TLR2/CD1D | 2 |  |
| BP | GO:0017014 | protein nitrosylation | 2/155 | 16/18870 | 0.125 | 15.21774 | 5.177669 | 0.007459 | 0.040543 | 0.029801 | S100A9/S100A8 | 2 |  |
| BP | GO:0018119 | peptidyl-cysteine S-nitrosylation | 2/155 | 16/18870 | 0.125 | 15.21774 | 5.177669 | 0.007459 | 0.040543 | 0.029801 | S100A9/S100A8 | 2 |  |
| BP | GO:0030201 | heparan sulfate proteoglycan metabolic process | 2/155 | 16/18870 | 0.125 | 15.21774 | 5.177669 | 0.007459 | 0.040543 | 0.029801 | SULF2/SULF1 | 2 |  |
| BP | GO:0042448 | progesterone metabolic process | 2/155 | 16/18870 | 0.125 | 15.21774 | 5.177669 | 0.007459 | 0.040543 | 0.029801 | ADM/EGR1 | 2 |  |
| BP | GO:0051044 | positive regulation of membrane protein ectodomain proteolysis | 2/155 | 16/18870 | 0.125 | 15.21774 | 5.177669 | 0.007459 | 0.040543 | 0.029801 | IL1B/ADAM9 | 2 |  |
| BP | GO:2000345 | regulation of hepatocyte proliferation | 2/155 | 16/18870 | 0.125 | 15.21774 | 5.177669 | 0.007459 | 0.040543 | 0.029801 | SULF2/TNFAIP3 | 2 |  |
| BP | GO:0002819 | regulation of adaptive immune response | 6/155 | 208/18870 | 0.028846 | 3.511787 | 3.314978 | 0.007504 | 0.040712 | 0.029926 | CD55/IL1B/TNFAIP3/NLRP3/NFKBIZ/CD1D | 6 |  |
| BP | GO:0070482 | response to oxygen levels | 8/155 | 343/18870 | 0.023324 | 2.839462 | 3.128821 | 0.007521 | 0.040724 | 0.029935 | NR4A2/TLR2/FOS/THBS1/PMAIP1/ADM/CDKN1A/EGR1 | 8 |  |
| BP | GO:0038061 | non-canonical NF-kappaB signal transduction | 5/155 | 148/18870 | 0.033784 | 4.112903 | 3.459917 | 0.007569 | 0.040755 | 0.029957 | TLR2/IL1B/TLR4/NLRP3/NFKBIA | 5 |  |
| BP | GO:0010543 | regulation of platelet activation | 3/155 | 49/18870 | 0.061224 | 7.453588 | 4.116454 | 0.007597 | 0.040755 | 0.029957 | THBD/FCER1G/TLR4 | 3 |  |
| BP | GO:0019884 | antigen processing and presentation of exogenous antigen | 3/155 | 49/18870 | 0.061224 | 7.453588 | 4.116454 | 0.007597 | 0.040755 | 0.029957 | FCER1G/CLEC4A/CD1D | 3 |  |
| BP | GO:0046596 | regulation of viral entry into host cell | 3/155 | 49/18870 | 0.061224 | 7.453588 | 4.116454 | 0.007597 | 0.040755 | 0.029957 | FCN1/CH25H/PTX3 | 3 |  |
| BP | GO:0072337 | modified amino acid transport | 3/155 | 49/18870 | 0.061224 | 7.453588 | 4.116454 | 0.007597 | 0.040755 | 0.029957 | SLC7A7/FOLR3/SLC22A4 | 3 |  |
| BP | GO:0001558 | regulation of cell growth | 9/155 | 417/18870 | 0.021583 | 2.627524 | 3.058491 | 0.007643 | 0.04093 | 0.030086 | S100A9/CDA/CXCL16/HBEGF/S100A8/CDKN1A/RGS2/BTG1/GJA1 | 9 |  |
| BP | GO:0045638 | negative regulation of myeloid cell differentiation | 4/155 | 95/18870 | 0.042105 | 5.125976 | 3.668968 | 0.00782 | 0.041798 | 0.030724 | ZFP36/TLR4/TOB2/NFKBIA | 4 |  |
| BP | GO:0071320 | cellular response to cAMP | 3/155 | 50/18870 | 0.06 | 7.304516 | 4.062303 | 0.008034 | 0.04286 | 0.031505 | AQP9/PTAFR/CYP1B1 | 3 |  |
| BP | GO:0001910 | regulation of leukocyte mediated cytotoxicity | 4/155 | 96/18870 | 0.041667 | 5.072581 | 3.640594 | 0.008109 | 0.043103 | 0.031684 | ICAM1/TYROBP/CRTAM/CD1D | 4 |  |
| BP | GO:0002690 | positive regulation of leukocyte chemotaxis | 4/155 | 96/18870 | 0.041667 | 5.072581 | 3.640594 | 0.008109 | 0.043103 | 0.031684 | C5AR1/THBS1/CCL4/AIF1 | 4 |  |
| BP | GO:0001894 | tissue homeostasis | 7/155 | 279/18870 | 0.02509 | 3.054457 | 3.14625 | 0.008345 | 0.043521 | 0.03199 | TLR4/TP53INP2/TNFAIP3/NFKBIZ/VSIG1/LYZ/GJA1 | 7 |  |
| BP | GO:0060249 | anatomical structure homeostasis | 7/155 | 279/18870 | 0.02509 | 3.054457 | 3.14625 | 0.008345 | 0.043521 | 0.03199 | TLR4/TP53INP2/TNFAIP3/NFKBIZ/VSIG1/LYZ/GJA1 | 7 |  |
| BP | GO:1903035 | negative regulation of response to wounding | 4/155 | 97/18870 | 0.041237 | 5.020286 | 3.612612 | 0.008405 | 0.043521 | 0.03199 | THBD/THBS1/PLAUR/SERPINB2 | 4 |  |
| BP | GO:0007252 | I-kappaB phosphorylation | 2/155 | 17/18870 | 0.117647 | 14.32258 | 5.001128 | 0.008408 | 0.043521 | 0.03199 | TLR2/TLR4 | 2 |  |
| BP | GO:0008228 | opsonization | 2/155 | 17/18870 | 0.117647 | 14.32258 | 5.001128 | 0.008408 | 0.043521 | 0.03199 | FCN1/PTX3 | 2 |  |
| BP | GO:0030889 | negative regulation of B cell proliferation | 2/155 | 17/18870 | 0.117647 | 14.32258 | 5.001128 | 0.008408 | 0.043521 | 0.03199 | TYROBP/MNDA | 2 |  |
| BP | GO:0032905 | transforming growth factor beta1 production | 2/155 | 17/18870 | 0.117647 | 14.32258 | 5.001128 | 0.008408 | 0.043521 | 0.03199 | THBS1/TYROBP | 2 |  |
| BP | GO:0034134 | toll-like receptor 2 signaling pathway | 2/155 | 17/18870 | 0.117647 | 14.32258 | 5.001128 | 0.008408 | 0.043521 | 0.03199 | TLR2/TNFAIP3 | 2 |  |
| BP | GO:0036005 | response to macrophage colony-stimulating factor | 2/155 | 17/18870 | 0.117647 | 14.32258 | 5.001128 | 0.008408 | 0.043521 | 0.03199 | TLR2/TLR4 | 2 |  |
| BP | GO:0055119 | relaxation of cardiac muscle | 2/155 | 17/18870 | 0.117647 | 14.32258 | 5.001128 | 0.008408 | 0.043521 | 0.03199 | KCNJ2/RGS2 | 2 |  |
| BP | GO:0070206 | protein trimerization | 2/155 | 17/18870 | 0.117647 | 14.32258 | 5.001128 | 0.008408 | 0.043521 | 0.03199 | ITLN1/STEAP4 | 2 |  |
| BP | GO:0090399 | replicative senescence | 2/155 | 17/18870 | 0.117647 | 14.32258 | 5.001128 | 0.008408 | 0.043521 | 0.03199 | CDKN1A/MME | 2 |  |
| BP | GO:0097396 | response to interleukin-17 | 2/155 | 17/18870 | 0.117647 | 14.32258 | 5.001128 | 0.008408 | 0.043521 | 0.03199 | IL1B/NFKBIZ | 2 |  |
| BP | GO:0097398 | cellular response to interleukin-17 | 2/155 | 17/18870 | 0.117647 | 14.32258 | 5.001128 | 0.008408 | 0.043521 | 0.03199 | IL1B/NFKBIZ | 2 |  |
| BP | GO:0010821 | regulation of mitochondrion organization | 5/155 | 152/18870 | 0.032895 | 4.004669 | 3.384808 | 0.008442 | 0.043521 | 0.03199 | MMP9/IER3/PMAIP1/PLAUR/LRRK2 | 5 |  |
| BP | GO:0045862 | positive regulation of proteolysis | 8/155 | 350/18870 | 0.022857 | 2.782673 | 3.063589 | 0.008443 | 0.043521 | 0.03199 | IL1B/S100A9/ADAM9/PMAIP1/S100A8/NLRP3/LRRK2/CARD8 | 8 |  |
| BP | GO:0051235 | maintenance of location | 8/155 | 350/18870 | 0.022857 | 2.782673 | 3.063589 | 0.008443 | 0.043521 | 0.03199 | IL1B/SRGN/FTH1/S100A9/DDIT3/S100A8/CCR5/NFKBIA | 8 |  |
| BP | GO:0032368 | regulation of lipid transport | 5/155 | 153/18870 | 0.03268 | 3.978495 | 3.366432 | 0.008671 | 0.044619 | 0.032798 | ACSL1/IL1B/IRS2/THBS1/NFKBIA | 5 |  |
| BP | GO:0034614 | cellular response to reactive oxygen species | 5/155 | 154/18870 | 0.032468 | 3.95266 | 3.34821 | 0.008904 | 0.045654 | 0.033558 | FOS/MMP9/TNFAIP3/CYP1B1/LRRK2 | 5 |  |
| BP | GO:0035306 | positive regulation of dephosphorylation | 3/155 | 52/18870 | 0.057692 | 7.023573 | 3.958353 | 0.008951 | 0.045654 | 0.033558 | PPP1R15A/LILRB2/CD33 | 3 |  |
| BP | GO:0043154 | negative regulation of cysteine-type endopeptidase activity involved in apoptotic process | 3/155 | 52/18870 | 0.057692 | 7.023573 | 3.958353 | 0.008951 | 0.045654 | 0.033558 | MMP9/THBS1/PLAUR | 3 |  |
| BP | GO:0046638 | positive regulation of alpha-beta T cell differentiation | 3/155 | 52/18870 | 0.057692 | 7.023573 | 3.958353 | 0.008951 | 0.045654 | 0.033558 | NLRP3/NFKBIZ/CD83 | 3 |  |
| BP | GO:1904707 | positive regulation of vascular associated smooth muscle cell proliferation | 3/155 | 52/18870 | 0.057692 | 7.023573 | 3.958353 | 0.008951 | 0.045654 | 0.033558 | MMP9/NR4A3/GJA1 | 3 |  |
| BP | GO:0002709 | regulation of T cell mediated immunity | 4/155 | 99/18870 | 0.040404 | 4.918866 | 3.557785 | 0.009017 | 0.04575 | 0.033629 | CD55/IL1B/NLRP3/CD1D | 4 |  |
| BP | GO:0141060 | disruption of anatomical structure in another organism | 4/155 | 99/18870 | 0.040404 | 4.918866 | 3.557785 | 0.009017 | 0.04575 | 0.033629 | S100A12/TREM1/GZMA/LYZ | 4 |  |
| BP | GO:2001237 | negative regulation of extrinsic apoptotic signaling pathway | 4/155 | 99/18870 | 0.040404 | 4.918866 | 3.557785 | 0.009017 | 0.04575 | 0.033629 | IL1B/THBS1/TNFAIP3/ICAM1 | 4 |  |
| BP | GO:0071901 | negative regulation of protein serine/threonine kinase activity | 4/155 | 100/18870 | 0.04 | 4.869677 | 3.530921 | 0.009334 | 0.046754 | 0.034367 | GADD45A/IL1B/CDKN1A/RGS2 | 4 |  |
| BP | GO:0070374 | positive regulation of ERK1 and ERK2 cascade | 6/155 | 218/18870 | 0.027523 | 3.350695 | 3.176927 | 0.00934 | 0.046754 | 0.034367 | CCL20/C5AR1/TLR4/CCL4/ICAM1/PDGFC | 6 |  |
| BP | GO:0010950 | positive regulation of endopeptidase activity | 5/155 | 156/18870 | 0.032051 | 3.901985 | 3.312223 | 0.009383 | 0.046754 | 0.034367 | S100A9/PMAIP1/S100A8/NLRP3/CARD8 | 5 |  |
| BP | GO:0002693 | positive regulation of cellular extravasation | 2/155 | 18/18870 | 0.111111 | 13.52688 | 4.838892 | 0.009409 | 0.046754 | 0.034367 | PTAFR/ICAM1 | 2 |  |
| BP | GO:0010042 | response to manganese ion | 2/155 | 18/18870 | 0.111111 | 13.52688 | 4.838892 | 0.009409 | 0.046754 | 0.034367 | ADAM9/LRRK2 | 2 |  |
| BP | GO:0010755 | regulation of plasminogen activation | 2/155 | 18/18870 | 0.111111 | 13.52688 | 4.838892 | 0.009409 | 0.046754 | 0.034367 | THBS1/PLAUR | 2 |  |
| BP | GO:0016264 | gap junction assembly | 2/155 | 18/18870 | 0.111111 | 13.52688 | 4.838892 | 0.009409 | 0.046754 | 0.034367 | IL1B/GJA1 | 2 |  |
| BP | GO:0032604 | granulocyte macrophage colony-stimulating factor production | 2/155 | 18/18870 | 0.111111 | 13.52688 | 4.838892 | 0.009409 | 0.046754 | 0.034367 | IL1B/LILRA2 | 2 |  |
| BP | GO:0032645 | regulation of granulocyte macrophage colony-stimulating factor production | 2/155 | 18/18870 | 0.111111 | 13.52688 | 4.838892 | 0.009409 | 0.046754 | 0.034367 | IL1B/LILRA2 | 2 |  |
| BP | GO:0036499 | PERK-mediated unfolded protein response | 2/155 | 18/18870 | 0.111111 | 13.52688 | 4.838892 | 0.009409 | 0.046754 | 0.034367 | PPP1R15A/DDIT3 | 2 |  |
| BP | GO:0038128 | ERBB2 signaling pathway | 2/155 | 18/18870 | 0.111111 | 13.52688 | 4.838892 | 0.009409 | 0.046754 | 0.034367 | HBEGF/AREG | 2 |  |
| BP | GO:0150078 | positive regulation of neuroinflammatory response | 2/155 | 18/18870 | 0.111111 | 13.52688 | 4.838892 | 0.009409 | 0.046754 | 0.034367 | IL1B/LRRK2 | 2 |  |
| BP | GO:0071622 | regulation of granulocyte chemotaxis | 3/155 | 53/18870 | 0.056604 | 6.891053 | 3.908418 | 0.009432 | 0.046788 | 0.034392 | BST1/C5AR1/THBS1 | 3 |  |
| BP | GO:0042542 | response to hydrogen peroxide | 4/155 | 101/18870 | 0.039604 | 4.821463 | 3.504412 | 0.009658 | 0.04783 | 0.035158 | ADAM9/TNFAIP3/AREG/CYP1B1 | 4 |  |
| BP | GO:0010038 | response to metal ion | 8/155 | 359/18870 | 0.022284 | 2.712912 | 2.982035 | 0.00975 | 0.048201 | 0.035431 | FOS/MMP9/THBS1/ADAM9/CD14/S100A8/FOSB/LRRK2 | 8 |  |
| BP | GO:0050873 | brown fat cell differentiation | 3/155 | 54/18870 | 0.055556 | 6.763441 | 3.859761 | 0.009927 | 0.048995 | 0.036014 | LRG1/CEBPB/RGS2 | 3 |  |
| BP | GO:0001829 | trophectodermal cell differentiation | 2/155 | 19/18870 | 0.105263 | 12.81494 | 4.689068 | 0.01046 | 0.050841 | 0.037371 | EOMES/CNOT1 | 2 |  |
| BP | GO:0002281 | macrophage activation involved in immune response | 2/155 | 19/18870 | 0.105263 | 12.81494 | 4.689068 | 0.01046 | 0.050841 | 0.037371 | DYSF/TYROBP | 2 |  |
| BP | GO:0002523 | leukocyte migration involved in inflammatory response | 2/155 | 19/18870 | 0.105263 | 12.81494 | 4.689068 | 0.01046 | 0.050841 | 0.037371 | S100A9/S100A8 | 2 |  |
| BP | GO:0009251 | glucan catabolic process | 2/155 | 19/18870 | 0.105263 | 12.81494 | 4.689068 | 0.01046 | 0.050841 | 0.037371 | PYGL/MGAM | 2 |  |
| BP | GO:0010759 | positive regulation of macrophage chemotaxis | 2/155 | 19/18870 | 0.105263 | 12.81494 | 4.689068 | 0.01046 | 0.050841 | 0.037371 | C5AR1/THBS1 | 2 |  |
| BP | GO:0032026 | response to magnesium ion | 2/155 | 19/18870 | 0.105263 | 12.81494 | 4.689068 | 0.01046 | 0.050841 | 0.037371 | THBS1/CD14 | 2 |  |
| BP | GO:0032695 | negative regulation of interleukin-12 production | 2/155 | 19/18870 | 0.105263 | 12.81494 | 4.689068 | 0.01046 | 0.050841 | 0.037371 | THBS1/LILRA5 | 2 |  |
| BP | GO:0035743 | CD4-positive, alpha-beta T cell cytokine production | 2/155 | 19/18870 | 0.105263 | 12.81494 | 4.689068 | 0.01046 | 0.050841 | 0.037371 | IL1B/NLRP3 | 2 |  |
| BP | GO:0042053 | regulation of dopamine metabolic process | 2/155 | 19/18870 | 0.105263 | 12.81494 | 4.689068 | 0.01046 | 0.050841 | 0.037371 | NR4A2/ALDH2 | 2 |  |
| BP | GO:0007596 | blood coagulation | 6/155 | 224/18870 | 0.026786 | 3.260945 | 3.097894 | 0.010585 | 0.051364 | 0.037755 | THBD/FCER1G/TLR4/THBS1/PLAUR/SERPINB2 | 6 |  |
| BP | GO:0019218 | regulation of steroid metabolic process | 4/155 | 104/18870 | 0.038462 | 4.682382 | 3.426929 | 0.010675 | 0.051713 | 0.038012 | CH25H/RORC/ADM/EGR1 | 4 |  |
| BP | GO:0051348 | negative regulation of transferase activity | 6/155 | 225/18870 | 0.026667 | 3.246452 | 3.084981 | 0.010803 | 0.052248 | 0.038405 | ZFP36/GADD45A/IL1B/IRS2/CDKN1A/RGS2 | 6 |  |
| BP | GO:0043370 | regulation of CD4-positive, alpha-beta T cell differentiation | 3/155 | 56/18870 | 0.053571 | 6.521889 | 3.766054 | 0.010963 | 0.05276 | 0.038781 | NLRP3/NFKBIZ/CD83 | 3 |  |
| BP | GO:0052372 | modulation by symbiont of entry into host | 3/155 | 56/18870 | 0.053571 | 6.521889 | 3.766054 | 0.010963 | 0.05276 | 0.038781 | FCN1/CH25H/PTX3 | 3 |  |
| BP | GO:0070542 | response to fatty acid | 3/155 | 56/18870 | 0.053571 | 6.521889 | 3.766054 | 0.010963 | 0.05276 | 0.038781 | ACSL1/TLR2/PTAFR | 3 |  |
| BP | GO:0042180 | cellular ketone metabolic process | 6/155 | 226/18870 | 0.026549 | 3.232087 | 3.072141 | 0.011025 | 0.052965 | 0.038933 | IL1B/IRS2/NR4A3/SLC7A7/ADM/EGR1 | 6 |  |
| BP | GO:0045055 | regulated exocytosis | 6/155 | 227/18870 | 0.026432 | 3.217849 | 3.059372 | 0.011249 | 0.053955 | 0.039661 | NR4A3/ANXA3/PTAFR/HCK/FGR/LRRK2 | 6 |  |
| BP | GO:0038066 | p38MAPK cascade | 3/155 | 57/18870 | 0.052632 | 6.40747 | 3.720899 | 0.011504 | 0.054809 | 0.040288 | ZFP36/GADD45A/IL1B | 3 |  |
| BP | GO:0043331 | response to dsRNA | 3/155 | 57/18870 | 0.052632 | 6.40747 | 3.720899 | 0.011504 | 0.054809 | 0.040288 | PMAIP1/PELI1/NFKBIA | 3 |  |
| BP | GO:0002577 | regulation of antigen processing and presentation | 2/155 | 20/18870 | 0.1 | 12.17419 | 4.550099 | 0.01156 | 0.054809 | 0.040288 | THBS1/LILRB2 | 2 |  |
| BP | GO:0002755 | MyD88-dependent toll-like receptor signaling pathway | 2/155 | 20/18870 | 0.1 | 12.17419 | 4.550099 | 0.01156 | 0.054809 | 0.040288 | TLR4/CD300LF | 2 |  |
| BP | GO:0006700 | C21-steroid hormone biosynthetic process | 2/155 | 20/18870 | 0.1 | 12.17419 | 4.550099 | 0.01156 | 0.054809 | 0.040288 | ADM/EGR1 | 2 |  |
| BP | GO:0033033 | negative regulation of myeloid cell apoptotic process | 2/155 | 20/18870 | 0.1 | 12.17419 | 4.550099 | 0.01156 | 0.054809 | 0.040288 | CCR5/ST6GAL1 | 2 |  |
| BP | GO:0042069 | regulation of catecholamine metabolic process | 2/155 | 20/18870 | 0.1 | 12.17419 | 4.550099 | 0.01156 | 0.054809 | 0.040288 | NR4A2/ALDH2 | 2 |  |
| BP | GO:0001666 | response to hypoxia | 7/155 | 298/18870 | 0.02349 | 2.85971 | 2.944892 | 0.011705 | 0.055333 | 0.040673 | NR4A2/TLR2/FOS/THBS1/PMAIP1/ADM/EGR1 | 7 |  |
| BP | GO:0050817 | coagulation | 6/155 | 229/18870 | 0.026201 | 3.189745 | 3.034045 | 0.011708 | 0.055333 | 0.040673 | THBD/FCER1G/TLR4/THBS1/PLAUR/SERPINB2 | 6 |  |
| BP | GO:0009743 | response to carbohydrate | 6/155 | 230/18870 | 0.026087 | 3.175877 | 3.021486 | 0.011943 | 0.056349 | 0.04142 | IL1B/IRS2/THBS1/ICAM1/GLUL/EGR1 | 6 |  |
| BP | GO:0045428 | regulation of nitric oxide biosynthetic process | 3/155 | 58/18870 | 0.051724 | 6.296997 | 3.676813 | 0.01206 | 0.056808 | 0.041758 | IL1B/TLR4/PTX3 | 3 |  |
| BP | GO:0007599 | hemostasis | 6/155 | 231/18870 | 0.025974 | 3.162128 | 3.008995 | 0.01218 | 0.057284 | 0.042107 | THBD/FCER1G/TLR4/THBS1/PLAUR/SERPINB2 | 6 |  |
| BP | GO:0050920 | regulation of chemotaxis | 6/155 | 232/18870 | 0.025862 | 3.148498 | 2.996572 | 0.012422 | 0.058323 | 0.042871 | BST1/C5AR1/THBS1/CCL4/ST6GAL1/AIF1 | 6 |  |
| BP | GO:0006939 | smooth muscle contraction | 4/155 | 109/18870 | 0.036697 | 4.467594 | 3.304143 | 0.012517 | 0.058437 | 0.042955 | SULF2/PTAFR/RGS2/SULF1 | 4 |  |
| BP | GO:0045936 | negative regulation of phosphate metabolic process | 8/155 | 376/18870 | 0.021277 | 2.590254 | 2.834591 | 0.012623 | 0.058437 | 0.042955 | PPP1R15A/GADD45A/IL1B/IRS2/CDA/CDKN1A/RGS2/LRRK2 | 8 |  |
| BP | GO:0002886 | regulation of myeloid leukocyte mediated immunity | 3/155 | 59/18870 | 0.050847 | 6.190268 | 3.633751 | 0.012631 | 0.058437 | 0.042955 | PTAFR/TYROBP/FGR | 3 |  |
| BP | GO:0031638 | zymogen activation | 3/155 | 59/18870 | 0.050847 | 6.190268 | 3.633751 | 0.012631 | 0.058437 | 0.042955 | THBD/THBS1/PLAUR | 3 |  |
| BP | GO:0032608 | interferon-beta production | 3/155 | 59/18870 | 0.050847 | 6.190268 | 3.633751 | 0.012631 | 0.058437 | 0.042955 | TLR2/TLR4/ARRDC4 | 3 |  |
| BP | GO:0032648 | regulation of interferon-beta production | 3/155 | 59/18870 | 0.050847 | 6.190268 | 3.633751 | 0.012631 | 0.058437 | 0.042955 | TLR2/TLR4/ARRDC4 | 3 |  |
| BP | GO:2000107 | negative regulation of leukocyte apoptotic process | 3/155 | 59/18870 | 0.050847 | 6.190268 | 3.633751 | 0.012631 | 0.058437 | 0.042955 | IRS2/CCR5/ST6GAL1 | 3 |  |
| BP | GO:0000272 | polysaccharide catabolic process | 2/155 | 21/18870 | 0.095238 | 11.59447 | 4.42069 | 0.012708 | 0.058437 | 0.042955 | PYGL/MGAM | 2 |  |
| BP | GO:0002888 | positive regulation of myeloid leukocyte mediated immunity | 2/155 | 21/18870 | 0.095238 | 11.59447 | 4.42069 | 0.012708 | 0.058437 | 0.042955 | PTAFR/TYROBP | 2 |  |
| BP | GO:0010224 | response to UV-B | 2/155 | 21/18870 | 0.095238 | 11.59447 | 4.42069 | 0.012708 | 0.058437 | 0.042955 | CDKN1A/MME | 2 |  |
| BP | GO:0010566 | regulation of ketone biosynthetic process | 2/155 | 21/18870 | 0.095238 | 11.59447 | 4.42069 | 0.012708 | 0.058437 | 0.042955 | ADM/EGR1 | 2 |  |
| BP | GO:0032495 | response to muramyl dipeptide | 2/155 | 21/18870 | 0.095238 | 11.59447 | 4.42069 | 0.012708 | 0.058437 | 0.042955 | TNFAIP3/NFKBIA | 2 |  |
| BP | GO:0033005 | positive regulation of mast cell activation | 2/155 | 21/18870 | 0.095238 | 11.59447 | 4.42069 | 0.012708 | 0.058437 | 0.042955 | NR4A3/FGR | 2 |  |
| BP | GO:0010563 | negative regulation of phosphorus metabolic process | 8/155 | 377/18870 | 0.02122 | 2.583383 | 2.826171 | 0.01281 | 0.05881 | 0.043229 | PPP1R15A/GADD45A/IL1B/IRS2/CDA/CDKN1A/RGS2/LRRK2 | 8 |  |
| BP | GO:0034250 | positive regulation of amide metabolic process | 5/155 | 169/18870 | 0.029586 | 3.601832 | 3.091973 | 0.012937 | 0.0593 | 0.043589 | PPP1R15A/THBS1/IFNGR1/PTAFR/CYP1B1 | 5 |  |
| BP | GO:0007566 | embryo implantation | 3/155 | 60/18870 | 0.05 | 6.087097 | 3.591671 | 0.013217 | 0.060393 | 0.044392 | IL1B/MMP9/GJA1 | 3 |  |
| BP | GO:0080164 | regulation of nitric oxide metabolic process | 3/155 | 60/18870 | 0.05 | 6.087097 | 3.591671 | 0.013217 | 0.060393 | 0.044392 | IL1B/TLR4/PTX3 | 3 |  |
| BP | GO:0010952 | positive regulation of peptidase activity | 5/155 | 170/18870 | 0.029412 | 3.580645 | 3.075936 | 0.013243 | 0.060417 | 0.04441 | S100A9/PMAIP1/S100A8/NLRP3/CARD8 | 5 |  |
| BP | GO:0007229 | integrin-mediated signaling pathway | 4/155 | 111/18870 | 0.036036 | 4.387097 | 3.257089 | 0.013307 | 0.060616 | 0.044556 | BST1/ADAM9/HCK/FGR | 4 |  |
| BP | GO:0030856 | regulation of epithelial cell differentiation | 5/155 | 171/18870 | 0.02924 | 3.559706 | 3.06002 | 0.013554 | 0.061642 | 0.045311 | ZFP36/IL1B/MMP9/CEBPB/BTG1 | 5 |  |
| BP | GO:0051651 | maintenance of location in cell | 6/155 | 237/18870 | 0.025316 | 3.082074 | 2.935448 | 0.013677 | 0.062105 | 0.045651 | SRGN/FTH1/S100A9/DDIT3/S100A8/CCR5 | 6 |  |
| BP | GO:0034113 | heterotypic cell-cell adhesion | 3/155 | 61/18870 | 0.04918 | 5.987308 | 3.550534 | 0.013818 | 0.062354 | 0.045834 | IL1B/LILRB2/CD1D | 3 |  |
| BP | GO:2000351 | regulation of endothelial cell apoptotic process | 3/155 | 61/18870 | 0.04918 | 5.987308 | 3.550534 | 0.013818 | 0.062354 | 0.045834 | THBS1/TNFAIP3/ICAM1 | 3 |  |
| BP | GO:0009713 | catechol-containing compound biosynthetic process | 2/155 | 22/18870 | 0.090909 | 11.06745 | 4.299753 | 0.013904 | 0.062354 | 0.045834 | NR4A2/ALDH2 | 2 |  |
| BP | GO:0030449 | regulation of complement activation | 2/155 | 22/18870 | 0.090909 | 11.06745 | 4.299753 | 0.013904 | 0.062354 | 0.045834 | CD55/IL1B | 2 |  |
| BP | GO:0032700 | negative regulation of interleukin-17 production | 2/155 | 22/18870 | 0.090909 | 11.06745 | 4.299753 | 0.013904 | 0.062354 | 0.045834 | TLR4/DDIT3 | 2 |  |
| BP | GO:0035313 | wound healing, spreading of epidermal cells | 2/155 | 22/18870 | 0.090909 | 11.06745 | 4.299753 | 0.013904 | 0.062354 | 0.045834 | LRG1/HBEGF | 2 |  |
| BP | GO:0042423 | catecholamine biosynthetic process | 2/155 | 22/18870 | 0.090909 | 11.06745 | 4.299753 | 0.013904 | 0.062354 | 0.045834 | NR4A2/ALDH2 | 2 |  |
| BP | GO:0045624 | positive regulation of T-helper cell differentiation | 2/155 | 22/18870 | 0.090909 | 11.06745 | 4.299753 | 0.013904 | 0.062354 | 0.045834 | NLRP3/NFKBIZ | 2 |  |
| BP | GO:1904035 | regulation of epithelial cell apoptotic process | 4/155 | 113/18870 | 0.035398 | 4.309449 | 3.211135 | 0.014129 | 0.063264 | 0.046503 | ZFP36/THBS1/TNFAIP3/ICAM1 | 4 |  |
| BP | GO:0007204 | positive regulation of cytosolic calcium ion concentration | 5/155 | 173/18870 | 0.028902 | 3.518553 | 3.028542 | 0.014191 | 0.063443 | 0.046634 | CD55/C5AR1/ADM/CCR5/CCRL2 | 5 |  |
| BP | GO:0043030 | regulation of macrophage activation | 3/155 | 62/18870 | 0.048387 | 5.890739 | 3.510301 | 0.014435 | 0.064336 | 0.047291 | TLR4/THBS1/LRRK2 | 3 |  |
| BP | GO:0090303 | positive regulation of wound healing | 3/155 | 62/18870 | 0.048387 | 5.890739 | 3.510301 | 0.014435 | 0.064336 | 0.047291 | THBD/THBS1/HBEGF | 3 |  |
| BP | GO:0070372 | regulation of ERK1 and ERK2 cascade | 7/155 | 313/18870 | 0.022364 | 2.722663 | 2.796817 | 0.014979 | 0.066656 | 0.048996 | IL1B/CCL20/C5AR1/TLR4/CCL4/ICAM1/PDGFC | 7 |  |
| BP | GO:0043171 | peptide catabolic process | 2/155 | 23/18870 | 0.086957 | 10.58626 | 4.186366 | 0.015147 | 0.067097 | 0.049321 | ANPEP/MME | 2 |  |
| BP | GO:0061760 | antifungal innate immune response | 2/155 | 23/18870 | 0.086957 | 10.58626 | 4.186366 | 0.015147 | 0.067097 | 0.049321 | CLEC4D/CLEC4A | 2 |  |
| BP | GO:2000193 | positive regulation of fatty acid transport | 2/155 | 23/18870 | 0.086957 | 10.58626 | 4.186366 | 0.015147 | 0.067097 | 0.049321 | ACSL1/IL1B | 2 |  |
| BP | GO:0002440 | production of molecular mediator of immune response | 7/155 | 314/18870 | 0.022293 | 2.713992 | 2.787256 | 0.015218 | 0.067307 | 0.049475 | CD55/IL1B/TREM1/NR4A3/TLR4/NLRP3/NFKBIZ | 7 |  |
| CC | GO:0070820 | tertiary granule | 19/162 | 164/19886 | 0.115854 | 14.22139 | 15.40801 | 8.48E-17 | 1.91E-14 | 1.61E-14 | CD55/CLEC4D/FTH1/CDA/MMP9/MGAM/FCER1G/LRG1/MCEMP1/QPCT/PTAFR/CXCL1/LILRB2/SLC2A3/CD33/FOLR3/PTX3/SIGLEC5/LYZ | 19 |  |
| CC | GO:0030667 | secretory granule membrane | 20/162 | 314/19886 | 0.063694 | 7.818668 | 11.03747 | 1.26E-12 | 1.42E-10 | 1.19E-10 | CD55/CLEC4D/TLR2/BST1/C5AR1/MGAM/FCER1G/ANPEP/MCEMP1/CD14/VNN1/PTAFR/LILRB2/SLC2A3/CD33/FCGR3B/PLAUR/MME/TYROBP/SIGLEC5 | 20 |  |
| CC | GO:0009897 | external side of plasma membrane | 21/162 | 387/19886 | 0.054264 | 6.66102 | 10.19217 | 7.13E-12 | 5.35E-10 | 4.50E-10 | CLEC4D/FCN1/THBD/CSF3R/FCER1G/TLR4/THBS1/ADAM9/ANPEP/CD14/CD163/CD33/ICAM1/FOLR3/PLAUR/CCR5/CCRL2/CLEC4A/CD83/CSF2RB/CD1D | 21 |  |
| CC | GO:0034774 | secretory granule lumen | 19/162 | 322/19886 | 0.059006 | 7.243195 | 10.23596 | 1.81E-11 | 8.40E-10 | 7.07E-10 | S100A12/FCN1/PYGL/SRGN/S100A9/CDA/S100P/THBS1/LRG1/QPCT/S100A8/CXCL1/FOLR3/GCA/PTX3/RNASE2/FGR/MNDA/LYZ | 19 |  |
| CC | GO:0060205 | cytoplasmic vesicle lumen | 19/162 | 325/19886 | 0.058462 | 7.176334 | 10.17419 | 2.12E-11 | 8.40E-10 | 7.07E-10 | S100A12/FCN1/PYGL/SRGN/S100A9/CDA/S100P/THBS1/LRG1/QPCT/S100A8/CXCL1/FOLR3/GCA/PTX3/RNASE2/FGR/MNDA/LYZ | 19 |  |
| CC | GO:0031983 | vesicle lumen | 19/162 | 326/19886 | 0.058282 | 7.154321 | 10.15377 | 2.24E-11 | 8.40E-10 | 7.07E-10 | S100A12/FCN1/PYGL/SRGN/S100A9/CDA/S100P/THBS1/LRG1/QPCT/S100A8/CXCL1/FOLR3/GCA/PTX3/RNASE2/FGR/MNDA/LYZ | 19 |  |
| CC | GO:0101002 | ficolin-1-rich granule | 15/162 | 185/19886 | 0.081081 | 9.952953 | 11.08743 | 3.16E-11 | 1.02E-09 | 8.56E-10 | CD55/CLEC4D/FCN1/PYGL/FTH1/CDA/MMP9/MGAM/FCER1G/LRG1/QPCT/LILRB2/SLC2A3/SIGLEC5/MNDA | 15 |  |
| CC | GO:1904724 | tertiary granule lumen | 9/162 | 55/19886 | 0.163636 | 20.08687 | 12.84596 | 5.83E-10 | 1.64E-08 | 1.38E-08 | FTH1/CDA/MMP9/LRG1/QPCT/CXCL1/FOLR3/PTX3/LYZ | 9 |  |
| CC | GO:0042581 | specific granule | 13/162 | 160/19886 | 0.08125 | 9.973688 | 10.32843 | 6.68E-10 | 1.67E-08 | 1.41E-08 | CLEC4D/BST1/LRG1/MCEMP1/ANXA3/QPCT/CXCL1/SLC2A3/CD33/FOLR3/PLAUR/PTX3/LYZ | 13 |  |
| CC | GO:0070821 | tertiary granule membrane | 9/162 | 73/19886 | 0.123288 | 15.13394 | 10.96409 | 7.86E-09 | 1.77E-07 | 1.49E-07 | CLEC4D/MGAM/FCER1G/MCEMP1/PTAFR/LILRB2/SLC2A3/CD33/SIGLEC5 | 9 |  |
| CC | GO:0101003 | ficolin-1-rich granule membrane | 7/162 | 61/19886 | 0.114754 | 14.08642 | 9.276881 | 6.30E-07 | 1.29E-05 | 1.09E-05 | CD55/CLEC4D/MGAM/FCER1G/LILRB2/SLC2A3/SIGLEC5 | 7 |  |
| CC | GO:1904813 | ficolin-1-rich granule lumen | 8/162 | 124/19886 | 0.064516 | 7.919554 | 7.004809 | 8.07E-06 | 0.000151 | 0.000127 | FCN1/PYGL/FTH1/CDA/MMP9/LRG1/QPCT/MNDA | 8 |  |
| CC | GO:0035580 | specific granule lumen | 6/162 | 62/19886 | 0.096774 | 11.87933 | 7.775444 | 1.12E-05 | 0.000194 | 0.000164 | LRG1/QPCT/CXCL1/FOLR3/PTX3/LYZ | 6 |  |
| CC | GO:1905370 | serine-type endopeptidase complex | 3/162 | 11/19886 | 0.272727 | 33.47811 | 9.764643 | 8.35E-05 | 0.001341 | 0.00113 | FCN1/THBD/PLAUR | 3 |  |
| CC | GO:0035579 | specific granule membrane | 6/162 | 91/19886 | 0.065934 | 8.09361 | 6.146573 | 0.0001 | 0.001504 | 0.001267 | CLEC4D/BST1/MCEMP1/SLC2A3/CD33/PLAUR | 6 |  |
| CC | GO:0045121 | membrane raft | 10/162 | 286/19886 | 0.034965 | 4.292066 | 5.082136 | 0.000123 | 0.001669 | 0.001406 | CD55/TLR2/CD14/ICAM1/ITLN1/MME/HCK/LRRK2/SULF1/GJA1 | 10 |  |
| CC | GO:0098857 | membrane microdomain | 10/162 | 287/19886 | 0.034843 | 4.277111 | 5.068016 | 0.000126 | 0.001669 | 0.001406 | CD55/TLR2/CD14/ICAM1/ITLN1/MME/HCK/LRRK2/SULF1/GJA1 | 10 |  |
| CC | GO:1905286 | serine-type peptidase complex | 3/162 | 13/19886 | 0.230769 | 28.32764 | 8.932331 | 0.000143 | 0.001787 | 0.001505 | FCN1/THBD/PLAUR | 3 |  |
| CC | GO:0005767 | secondary lysosome | 3/162 | 18/19886 | 0.166667 | 20.45885 | 7.485126 | 0.000396 | 0.004687 | 0.003947 | FTH1/NCF2/LRRK2 | 3 |  |
| CC | GO:0030139 | endocytic vesicle | 10/162 | 348/19886 | 0.028736 | 3.527388 | 4.310664 | 0.000584 | 0.006571 | 0.005533 | TLR2/DYSF/CSF3R/ANXA3/HBEGF/RAB32/CD163/MPEG1/AREG/NCF2 | 10 |  |
| CC | GO:0030666 | endocytic vesicle membrane | 7/162 | 202/19886 | 0.034653 | 4.25382 | 4.212455 | 0.001361 | 0.014587 | 0.012284 | TLR2/CSF3R/ANXA3/HBEGF/RAB32/CD163/AREG | 7 |  |
| CC | GO:0009925 | basal plasma membrane | 8/162 | 282/19886 | 0.028369 | 3.482357 | 3.804865 | 0.002232 | 0.022828 | 0.019224 | AQP9/C5AR1/SLC7A7/ADAM9/VSIG1/CD1D/SLC22A4/LRRK2 | 8 |  |
| CC | GO:0045178 | basal part of cell | 8/162 | 301/19886 | 0.026578 | 3.262541 | 3.584599 | 0.003326 | 0.03254 | 0.027402 | AQP9/C5AR1/SLC7A7/ADAM9/VSIG1/CD1D/SLC22A4/LRRK2 | 8 |  |
| CC | GO:0044754 | autolysosome | 2/162 | 12/19886 | 0.166667 | 20.45885 | 6.110657 | 0.004126 | 0.03868 | 0.032573 | FTH1/LRRK2 | 2 |  |
| CC | GO:0016323 | basolateral plasma membrane | 7/162 | 249/19886 | 0.028112 | 3.45089 | 3.527027 | 0.00438 | 0.039416 | 0.033192 | AQP9/C5AR1/SLC7A7/ADAM9/VSIG1/CD1D/LRRK2 | 7 |  |
| CC | GO:0001772 | immunological synapse | 3/162 | 45/19886 | 0.066667 | 8.183539 | 4.372056 | 0.005861 | 0.050721 | 0.042712 | GZMA/ICAM1/CRTAM | 3 |  |
| CC | GO:0045335 | phagocytic vesicle | 5/162 | 142/19886 | 0.035211 | 4.322292 | 3.600702 | 0.006177 | 0.051476 | 0.043348 | TLR2/ANXA3/RAB32/MPEG1/NCF2 | 5 |  |
| CC | GO:0035578 | azurophil granule lumen | 4/162 | 91/19886 | 0.043956 | 5.39574 | 3.808884 | 0.00655 | 0.052633 | 0.044322 | GCA/RNASE2/MNDA/LYZ | 4 |  |
| MF | GO:0038187 | pattern recognition receptor activity | 7/160 | 34/18496 | 0.205882 | 23.8 | 12.42996 | 1.40E-08 | 6.10E-06 | 4.97E-06 | CLEC4D/TLR2/FCN1/TLR4/CD14/PTAFR/CARD8 | 7 |  |
| MF | GO:0140375 | immune receptor activity | 11/160 | 147/18496 | 0.07483 | 8.65034 | 8.698975 | 6.18E-08 | 1.34E-05 | 1.09E-05 | IL1R2/C5AR1/CSF3R/FCER1G/LILRA2/IFNGR1/LILRA5/LILRB2/CCR5/CCRL2/CSF2RB | 11 |  |
| MF | GO:0032396 | inhibitory MHC class I receptor activity | 3/160 | 10/18496 | 0.3 | 34.68 | 9.951429 | 7.29E-05 | 0.007911 | 0.006447 | LILRA2/LILRA5/LILRB2 | 3 |  |
| MF | GO:0050786 | RAGE receptor binding | 3/160 | 10/18496 | 0.3 | 34.68 | 9.951429 | 7.29E-05 | 0.007911 | 0.006447 | S100A12/S100A9/S100A8 | 3 |  |
| MF | GO:0030246 | carbohydrate binding | 10/160 | 273/18496 | 0.03663 | 4.234432 | 5.029262 | 0.000136 | 0.010862 | 0.008852 | CLEC4D/FCN1/PYGL/MGAM/SLC2A3/CD33/ITLN1/PTX3/CLEC4A/SIGLEC5 | 10 |  |
| MF | GO:0035325 | Toll-like receptor binding | 3/160 | 13/18496 | 0.230769 | 26.67692 | 8.65094 | 0.00017 | 0.010862 | 0.008852 | TLR2/S100A9/S100A8 | 3 |  |
| MF | GO:0004896 | cytokine receptor activity | 6/160 | 96/18496 | 0.0625 | 7.225 | 5.712167 | 0.000186 | 0.010862 | 0.008852 | IL1R2/CSF3R/IFNGR1/CCR5/CCRL2/CSF2RB | 6 |  |
| MF | GO:0032393 | MHC class I receptor activity | 3/160 | 15/18496 | 0.2 | 23.12 | 8.005766 | 0.000268 | 0.010862 | 0.008852 | LILRA2/LILRA5/LILRB2 | 3 |  |
| MF | GO:0019955 | cytokine binding | 7/160 | 145/18496 | 0.048276 | 5.58069 | 5.172731 | 0.00027 | 0.010862 | 0.008852 | IL1R2/ZFP36/CSF3R/THBS1/IFNGR1/CCR5/CCRL2 | 7 |  |
| MF | GO:0031406 | carboxylic acid binding | 8/160 | 195/18496 | 0.041026 | 4.742564 | 4.907768 | 0.000301 | 0.010862 | 0.008852 | FCN1/PYGL/S100A9/RBP7/S100A8/CD33/FOLR3/SIGLEC5 | 8 |  |
| MF | GO:0003953 | NAD+ nucleosidase activity | 3/160 | 16/18496 | 0.1875 | 21.675 | 7.728396 | 0.000328 | 0.010862 | 0.008852 | TLR2/BST1/TLR4 | 3 |  |
| MF | GO:0050135 | NADP+ nucleosidase activity | 3/160 | 16/18496 | 0.1875 | 21.675 | 7.728396 | 0.000328 | 0.010862 | 0.008852 | TLR2/BST1/TLR4 | 3 |  |
| MF | GO:0061809 | NAD+ nucleotidase, cyclic ADP-ribose generating | 3/160 | 16/18496 | 0.1875 | 21.675 | 7.728396 | 0.000328 | 0.010862 | 0.008852 | TLR2/BST1/TLR4 | 3 |  |
| MF | GO:0001530 | lipopolysaccharide binding | 4/160 | 39/18496 | 0.102564 | 11.85641 | 6.339761 | 0.00035 | 0.010862 | 0.008852 | TLR2/TLR4/CD14/PTAFR | 4 |  |
| MF | GO:0043177 | organic acid binding | 8/160 | 207/18496 | 0.038647 | 4.467633 | 4.686603 | 0.000449 | 0.012291 | 0.010016 | FCN1/PYGL/S100A9/RBP7/S100A8/CD33/FOLR3/SIGLEC5 | 8 |  |
| MF | GO:0042379 | chemokine receptor binding | 5/160 | 74/18496 | 0.067568 | 7.810811 | 5.483794 | 0.000453 | 0.012291 | 0.010016 | CCL20/CXCL16/CCL4/CXCL1/CCRL2 | 5 |  |
| MF | GO:0048306 | calcium-dependent protein binding | 5/160 | 79/18496 | 0.063291 | 7.316456 | 5.25548 | 0.000612 | 0.015633 | 0.01274 | S100A12/S100A9/S100P/ANXA3/S100A8 | 5 |  |
| MF | GO:0008009 | chemokine activity | 4/160 | 49/18496 | 0.081633 | 9.436735 | 5.523882 | 0.000844 | 0.019804 | 0.016139 | CCL20/CXCL16/CCL4/CXCL1 | 4 |  |
| MF | GO:0033691 | sialic acid binding | 3/160 | 22/18496 | 0.136364 | 15.76364 | 6.472306 | 0.000867 | 0.019804 | 0.016139 | FCN1/CD33/SIGLEC5 | 3 |  |
| MF | GO:0019865 | immunoglobulin binding | 3/160 | 24/18496 | 0.125 | 14.45 | 6.158938 | 0.001125 | 0.023252 | 0.018949 | FCER1G/LILRA2/FCGR3B | 3 |  |
| MF | GO:0019957 | C-C chemokine binding | 3/160 | 24/18496 | 0.125 | 14.45 | 6.158938 | 0.001125 | 0.023252 | 0.018949 | ZFP36/CCR5/CCRL2 | 3 |  |
| MF | GO:0070851 | growth factor receptor binding | 6/160 | 138/18496 | 0.043478 | 5.026087 | 4.434502 | 0.001282 | 0.025288 | 0.020608 | IL1B/EFEMP1/HBEGF/AREG/CD300LF/PDGFC | 6 |  |
| MF | GO:0071889 | 14-3-3 protein binding | 3/160 | 32/18496 | 0.09375 | 10.8375 | 5.202735 | 0.002621 | 0.047639 | 0.038823 | ZFP36/IRS2/TMCC3 | 3 |  |
| MF | GO:0005126 | cytokine receptor binding | 8/160 | 273/18496 | 0.029304 | 3.387546 | 3.712427 | 0.002634 | 0.047639 | 0.038823 | IL1B/CCL20/CXCL16/LRG1/CCL4/CXCL1/CD300LF/CCRL2 | 8 |  |
| MF | GO:0019956 | chemokine binding | 3/160 | 33/18496 | 0.090909 | 10.50909 | 5.107163 | 0.002865 | 0.049738 | 0.040533 | ZFP36/CCR5/CCRL2 | 3 |  |
| MF | GO:0071723 | lipopeptide binding | 2/160 | 10/18496 | 0.2 | 23.12 | 6.535796 | 0.003197 | 0.053372 | 0.043495 | TLR2/CD1D | 2 |  |
| MF | GO:0005154 | epidermal growth factor receptor binding | 3/160 | 35/18496 | 0.085714 | 9.908571 | 4.927758 | 0.003394 | 0.054553 | 0.044457 | EFEMP1/HBEGF/AREG | 3 |  |
| MF | GO:0005536 | D-glucose binding | 2/160 | 11/18496 | 0.181818 | 21.01818 | 6.203633 | 0.003886 | 0.056215 | 0.045812 | PYGL/SLC2A3 | 2 |  |
| MF | GO:0019864 | IgG binding | 2/160 | 11/18496 | 0.181818 | 21.01818 | 6.203633 | 0.003886 | 0.056215 | 0.045812 | FCER1G/FCGR3B | 2 |  |
| MF | GO:0043522 | leucine zipper domain binding | 2/160 | 11/18496 | 0.181818 | 21.01818 | 6.203633 | 0.003886 | 0.056215 | 0.045812 | JDP2/DDIT3 | 2 |  |
| MF | GO:0048029 | monosaccharide binding | 4/160 | 75/18496 | 0.053333 | 6.165333 | 4.187041 | 0.004071 | 0.056697 | 0.046205 | CLEC4D/PYGL/SLC2A3/CLEC4A | 4 |  |
| MF | GO:0016799 | hydrolase activity, hydrolyzing N-glycosyl compounds | 3/160 | 38/18496 | 0.078947 | 9.126316 | 4.68412 | 0.004292 | 0.056697 | 0.046205 | TLR2/BST1/TLR4 | 3 |  |
| MF | GO:0005125 | cytokine activity | 7/160 | 237/18496 | 0.029536 | 3.414346 | 3.494379 | 0.004623 | 0.056697 | 0.046205 | IL1B/CCL20/CXCL16/CMTM2/CCL4/CXCL1/AREG | 7 |  |
| MF | GO:0035259 | nuclear glucocorticoid receptor binding | 2/160 | 12/18496 | 0.166667 | 19.26667 | 5.912713 | 0.004637 | 0.056697 | 0.046205 | NR4A2/NR4A3 | 2 |  |
| MF | GO:0035497 | cAMP response element binding | 2/160 | 12/18496 | 0.166667 | 19.26667 | 5.912713 | 0.004637 | 0.056697 | 0.046205 | JDP2/NR4A3 | 2 |  |
| MF | GO:0001618 | virus receptor activity | 4/160 | 79/18496 | 0.050633 | 5.853165 | 4.037978 | 0.0049 | 0.056697 | 0.046205 | CD55/ANPEP/ICAM1/CCR5 | 4 |  |
| MF | GO:0033293 | monocarboxylic acid binding | 4/160 | 79/18496 | 0.050633 | 5.853165 | 4.037978 | 0.0049 | 0.056697 | 0.046205 | PYGL/S100A9/RBP7/S100A8 | 4 |  |
| MF | GO:0140313 | molecular sequestering activity | 3/160 | 40/18496 | 0.075 | 8.67 | 4.536192 | 0.004964 | 0.056697 | 0.046205 | FTH1/CDKN1A/NFKBIA | 3 |  |
| MF | GO:0140272 | exogenous protein binding | 4/160 | 80/18496 | 0.05 | 5.78 | 4.002304 | 0.005124 | 0.057018 | 0.046466 | CD55/ANPEP/ICAM1/CCR5 | 4 |  |
| MF | GO:0045028 | G protein-coupled purinergic nucleotide receptor activity | 2/160 | 13/18496 | 0.153846 | 17.78462 | 5.654988 | 0.005449 | 0.059117 | 0.048177 | PTAFR/P2RY13 | 2 |  |
| MF | GO:0016798 | hydrolase activity, acting on glycosyl bonds | 5/160 | 131/18496 | 0.038168 | 4.412214 | 3.661097 | 0.005651 | 0.059822 | 0.048751 | TLR2/BST1/MGAM/TLR4/LYZ | 5 |  |

**Table S6** Acylcarnitine-related genes

| **Gene Symbol** | **Description** | **Category** | **Uniprot ID** | **Gifts** | **GC Id** | **Relevance score** | **GeneCards Link** |
| --- | --- | --- | --- | --- | --- | --- | --- |
| SLC25A20 | Solute Carrier Family 25 Member 20 | Protein Coding | O43772 | 59 | GC03M048969 | 109.7962 | https://www.genecards.org/cgi-bin/carddisp.pl?gene=SLC25A20 |
| CPT2 | Carnitine Palmitoyltransferase 2 | Protein Coding | P23786 | 63 | GC01P053196 | 22.18254 | https://www.genecards.org/cgi-bin/carddisp.pl?gene=CPT2 |
| HADHA | Hydroxyacyl-CoA Dehydrogenase Trifunctional Multienzyme Complex Subunit Alpha | Protein Coding | P40939 | 60 | GC02M026190 | 16.55965 | https://www.genecards.org/cgi-bin/carddisp.pl?gene=HADHA |
| ACADVL | Acyl-CoA Dehydrogenase Very Long Chain | Protein Coding | P49748 | 59 | GC17P143337 | 15.82084 | https://www.genecards.org/cgi-bin/carddisp.pl?gene=ACADVL |
| SLC25A29 | Solute Carrier Family 25 Member 29 | Protein Coding | Q8N8R3 | 51 | GC14M123213 | 14.61401 | https://www.genecards.org/cgi-bin/carddisp.pl?gene=SLC25A29 |
| CPT1A | Carnitine Palmitoyltransferase 1A | Protein Coding | P50416 | 61 | GC11M068754 | 14.14382 | https://www.genecards.org/cgi-bin/carddisp.pl?gene=CPT1A |
| ETFDH | Electron Transfer Flavoprotein Dehydrogenase | Protein Coding | Q16134 | 59 | GC04P158672 | 12.44958 | https://www.genecards.org/cgi-bin/carddisp.pl?gene=ETFDH |
| ACADS | Acyl-CoA Dehydrogenase Short Chain | Protein Coding | P16219 | 59 | GC12P137479 | 11.96389 | https://www.genecards.org/cgi-bin/carddisp.pl?gene=ACADS |
| ACAD8 | Acyl-CoA Dehydrogenase Family Member 8 | Protein Coding | Q9UKU7 | 56 | GC11P134253 | 11.7344 | https://www.genecards.org/cgi-bin/carddisp.pl?gene=ACAD8 |
| HADH | Hydroxyacyl-CoA Dehydrogenase | Protein Coding | Q16836 | 60 | GC04P107989 | 11.07321 | https://www.genecards.org/cgi-bin/carddisp.pl?gene=HADH |
| HADHB | Hydroxyacyl-CoA Dehydrogenase Trifunctional Multienzyme Complex Subunit Beta | Protein Coding | P55084 | 59 | GC02P026243 | 10.93453 | https://www.genecards.org/cgi-bin/carddisp.pl?gene=HADHB |
| SLC22A5 | Solute Carrier Family 22 Member 5 | Protein Coding | O76082 | 60 | GC05P132369 | 10.89967 | https://www.genecards.org/cgi-bin/carddisp.pl?gene=SLC22A5 |
| SLC52A1 | Solute Carrier Family 52 Member 1 | Protein Coding | Q9NWF4 | 51 | GC17M005032 | 10.23983 | https://www.genecards.org/cgi-bin/carddisp.pl?gene=SLC52A1 |
| ACADM | Acyl-CoA Dehydrogenase Medium Chain | Protein Coding | P11310 | 59 | GC01P075724 | 10.13369 | https://www.genecards.org/cgi-bin/carddisp.pl?gene=ACADM |
| ETFB | Electron Transfer Flavoprotein Subunit Beta | Protein Coding | P38117 | 59 | GC19M051345 | 8.801371 | https://www.genecards.org/cgi-bin/carddisp.pl?gene=ETFB |
| GCDH | Glutaryl-CoA Dehydrogenase | Protein Coding | Q92947 | 58 | GC19P012891 | 8.748331 | https://www.genecards.org/cgi-bin/carddisp.pl?gene=GCDH |
| TANGO2 | Transport And Golgi Organization 2 Homolog | Protein Coding | Q6ICL3 | 47 | GC22P020017 | 8.74606 | https://www.genecards.org/cgi-bin/carddisp.pl?gene=TANGO2 |
| CBS | Cystathionine Beta-Synthase | Protein Coding | P35520 | 64 | GC21M043053 | 8.714819 | https://www.genecards.org/cgi-bin/carddisp.pl?gene=CBS |
| ACADSB | Acyl-CoA Dehydrogenase Short/Branched Chain | Protein Coding | P45954 | 58 | GC10P123008 | 7.775606 | https://www.genecards.org/cgi-bin/carddisp.pl?gene=ACADSB |
| CD320 | CD320 Molecule | Protein Coding | Q9NPF0 | 53 | GC19M008302 | 7.171522 | https://www.genecards.org/cgi-bin/carddisp.pl?gene=CD320 |
| ETHE1 | ETHE1 Persulfide Dioxygenase | Protein Coding | O95571 | 56 | GC19M043506 | 7.126925 | https://www.genecards.org/cgi-bin/carddisp.pl?gene=ETHE1 |
| MMAB | Metabolism Of Cobalamin Associated B | Protein Coding | Q96EY8 | 56 | GC12M109553 | 6.962017 | https://www.genecards.org/cgi-bin/carddisp.pl?gene=MMAB |
| IVD | Isovaleryl-CoA Dehydrogenase | Protein Coding | P26440 | 57 | GC15P040405 | 6.876271 | https://www.genecards.org/cgi-bin/carddisp.pl?gene=IVD |
| PPIA | Peptidylprolyl Isomerase A | Protein Coding | P62937 | 58 | GC07P044869 | 6.870746 | https://www.genecards.org/cgi-bin/carddisp.pl?gene=PPIA |
| NUP153 | Nucleoporin 153 | Protein Coding | P49790 | 54 | GC06M017615 | 6.870746 | https://www.genecards.org/cgi-bin/carddisp.pl?gene=NUP153 |
| TRIM5 | Tripartite Motif Containing 5 | Protein Coding | Q9C035 | 54 | GC11M014174 | 6.870746 | https://www.genecards.org/cgi-bin/carddisp.pl?gene=TRIM5 |
| CPSF6 | Cleavage And Polyadenylation Specific Factor 6 | Protein Coding | Q16630 | 50 | GC12P069239 | 6.870746 | https://www.genecards.org/cgi-bin/carddisp.pl?gene=CPSF6 |
| IPPK | Inositol-Pentakisphosphate 2-Kinase | Protein Coding | Q9H8X2 | 47 | GC09M092613 | 6.870746 | https://www.genecards.org/cgi-bin/carddisp.pl?gene=IPPK |
| RGPD2 | RANBP2 Like And GRIP Domain Containing 2 | Protein Coding | P0DJD1 | 40 | GC02M087755 | 6.870746 | https://www.genecards.org/cgi-bin/carddisp.pl?gene=RGPD2 |
| LMBRD1 | LMBR1 Domain Containing 1 | Protein Coding | Q9NUN5 | 52 | GC06M103806 | 6.843258 | https://www.genecards.org/cgi-bin/carddisp.pl?gene=LMBRD1 |
| ACAD9 | Acyl-CoA Dehydrogenase Family Member 9 | Protein Coding | Q9H845 | 57 | GC03P141955 | 6.827713 | https://www.genecards.org/cgi-bin/carddisp.pl?gene=ACAD9 |
| MMACHC | Metabolism Of Cobalamin Associated C | Protein Coding | Q9Y4U1 | 56 | GC01P045500 | 6.440285 | https://www.genecards.org/cgi-bin/carddisp.pl?gene=MMACHC |
| MCEE | Methylmalonyl-CoA Epimerase | Protein Coding | Q96PE7 | 52 | GC02M071110 | 5.986885 | https://www.genecards.org/cgi-bin/carddisp.pl?gene=MCEE |
| CRAT | Carnitine O-Acetyltransferase | Protein Coding | P43155 | 59 | GC09M129094 | 5.957776 | https://www.genecards.org/cgi-bin/carddisp.pl?gene=CRAT |
| ABCD4 | ATP Binding Cassette Subfamily D Member 4 | Protein Coding | O14678 | 54 | GC14M074285 | 5.778746 | https://www.genecards.org/cgi-bin/carddisp.pl?gene=ABCD4 |
| ACAT1 | Acetyl-CoA Acetyltransferase 1 | Protein Coding | P24752 | 63 | GC11P108121 | 5.422899 | https://www.genecards.org/cgi-bin/carddisp.pl?gene=ACAT1 |
| LYRM7 | LYR Motif Containing 7 | Protein Coding | Q5U5X0 | 51 | GC05P131170 | 5.229558 | https://www.genecards.org/cgi-bin/carddisp.pl?gene=LYRM7 |
| NADK2 | NAD Kinase 2, Mitochondrial | Protein Coding | Q4G0N4 | 50 | GC05M036629 | 5.218705 | https://www.genecards.org/cgi-bin/carddisp.pl?gene=NADK2 |
| TAFAZZIN | Tafazzin, Phospholipid-Lysophospholipid Transacylase | Protein Coding | Q16635 | 54 | GC0XP155056 | 5.172917 | https://www.genecards.org/cgi-bin/carddisp.pl?gene=TAFAZZIN |
| ETFA | Electron Transfer Flavoprotein Subunit Alpha | Protein Coding | P13804 | 59 | GC15M159336 | 5.087959 | https://www.genecards.org/cgi-bin/carddisp.pl?gene=ETFA |
| COX16 | Cytochrome C Oxidase Assembly Factor COX16 | Protein Coding | Q9P0S2 | 46 | GC14M070326 | 5.042312 | https://www.genecards.org/cgi-bin/carddisp.pl?gene=COX16 |
| HSD17B10 | Hydroxysteroid 17-Beta Dehydrogenase 10 | Protein Coding | Q99714 | 60 | GC0XM053431 | 4.746778 | https://www.genecards.org/cgi-bin/carddisp.pl?gene=HSD17B10 |
| FLAD1 | Flavin Adenine Dinucleotide Synthetase 1 | Protein Coding | Q8NFF5 | 53 | GC01P154983 | 4.571025 | https://www.genecards.org/cgi-bin/carddisp.pl?gene=FLAD1 |
| MCCC2 | Methylcrotonyl-CoA Carboxylase Subunit 2 | Protein Coding | Q9HCC0 | 56 | GC05P081216 | 4.445385 | https://www.genecards.org/cgi-bin/carddisp.pl?gene=MCCC2 |
| GAREM2 | GRB2 Associated Regulator Of MAPK1 Subtype 2 | Protein Coding | Q75VX8 | 40 | GC02P027023 | 4.311293 | https://www.genecards.org/cgi-bin/carddisp.pl?gene=GAREM2 |
| SLC25A13 | Solute Carrier Family 25 Member 13 | Protein Coding | Q9UJS0 | 59 | GC07M096120 | 4.22928 | https://www.genecards.org/cgi-bin/carddisp.pl?gene=SLC25A13 |
| HLCS | Holocarboxylase Synthetase | Protein Coding | P50747 | 56 | GC21M036750 | 4.221951 | https://www.genecards.org/cgi-bin/carddisp.pl?gene=HLCS |
| SLC52A3 | Solute Carrier Family 52 Member 3 | Protein Coding | Q9NQ40 | 55 | GC20M000741 | 3.971438 | https://www.genecards.org/cgi-bin/carddisp.pl?gene=SLC52A3 |
| PC | Pyruvate Carboxylase | Protein Coding | P11498 | 60 | GC11M066848 | 3.561182 | https://www.genecards.org/cgi-bin/carddisp.pl?gene=PC |
| SLC52A2 | Solute Carrier Family 52 Member 2 | Protein Coding | Q9HAB3 | 51 | GC08P144333 | 3.54289 | https://www.genecards.org/cgi-bin/carddisp.pl?gene=SLC52A2 |
| GAMT | Guanidinoacetate N-Methyltransferase | Protein Coding | Q14353 | 60 | GC19M001397 | 3.509829 | https://www.genecards.org/cgi-bin/carddisp.pl?gene=GAMT |
| MMUT | Methylmalonyl-CoA Mutase | Protein Coding | P22033 | 59 | GC06M049430 | 3.400647 | https://www.genecards.org/cgi-bin/carddisp.pl?gene=MMUT |
| MTHFR | Methylenetetrahydrofolate Reductase | Protein Coding | P42898 | 61 | GC01M011785 | 3.305044 | https://www.genecards.org/cgi-bin/carddisp.pl?gene=MTHFR |
| MCCC1 | Methylcrotonyl-CoA Carboxylase Subunit 1 | Protein Coding | Q96RQ3 | 57 | GC03M183015 | 3.264039 | https://www.genecards.org/cgi-bin/carddisp.pl?gene=MCCC1 |
| NAGS | N-Acetylglutamate Synthase | Protein Coding | Q8N159 | 54 | GC17P044004 | 3.177626 | https://www.genecards.org/cgi-bin/carddisp.pl?gene=NAGS |
| PCCB | Propionyl-CoA Carboxylase Subunit Beta | Protein Coding | P05166 | 60 | GC03P136250 | 3.140154 | https://www.genecards.org/cgi-bin/carddisp.pl?gene=PCCB |
| HBB | Hemoglobin Subunit Beta | Protein Coding | P68871 | 59 | GC11M014141 | 3.11903 | https://www.genecards.org/cgi-bin/carddisp.pl?gene=HBB |
| CHAT | Choline O-Acetyltransferase | Protein Coding | P28329 | 61 | GC10P049609 | 3.111593 | https://www.genecards.org/cgi-bin/carddisp.pl?gene=CHAT |
| APOB | Apolipoprotein B | Protein Coding | P04114 | 59 | GC02M020956 | 3.111593 | https://www.genecards.org/cgi-bin/carddisp.pl?gene=APOB |
| CYP3A4 | Cytochrome P450 Family 3 Subfamily A Member 4 | Protein Coding | P08684 | 61 | GC07M106386 | 3.102929 | https://www.genecards.org/cgi-bin/carddisp.pl?gene=CYP3A4 |
| HSD17B4 | Hydroxysteroid 17-Beta Dehydrogenase 4 | Protein Coding | P51659 | 60 | GC05P119452 | 3.086148 | https://www.genecards.org/cgi-bin/carddisp.pl?gene=HSD17B4 |
| SLC22A1 | Solute Carrier Family 22 Member 1 | Protein Coding | O15245 | 55 | GC06P160121 | 3.047498 | https://www.genecards.org/cgi-bin/carddisp.pl?gene=SLC22A1 |
| ECHS1 | Enoyl-CoA Hydratase, Short Chain 1 | Protein Coding | P30084 | 59 | GC10M133362 | 2.953765 | https://www.genecards.org/cgi-bin/carddisp.pl?gene=ECHS1 |
| OTC | Ornithine Transcarbamylase | Protein Coding | P00480 | 60 | GC0XP038345 | 2.941741 | https://www.genecards.org/cgi-bin/carddisp.pl?gene=OTC |
| PAH | Phenylalanine Hydroxylase | Protein Coding | P00439 | 60 | GC12M102836 | 2.941741 | https://www.genecards.org/cgi-bin/carddisp.pl?gene=PAH |
| ACADL | Acyl-CoA Dehydrogenase Long Chain | Protein Coding | P28330 | 53 | GC02M210187 | 2.930552 | https://www.genecards.org/cgi-bin/carddisp.pl?gene=ACADL |
| SLC25A48 | Solute Carrier Family 25 Member 48 | Protein Coding | Q6ZT89 | 37 | GC05P135870 | 2.91228 | https://www.genecards.org/cgi-bin/carddisp.pl?gene=SLC25A48 |
| UCP3 | Uncoupling Protein 3 | Protein Coding | P55916 | 54 | GC11M074000 | 2.91129 | https://www.genecards.org/cgi-bin/carddisp.pl?gene=UCP3 |
| QDPR | Quinoid Dihydropteridine Reductase | Protein Coding | P09417 | 59 | GC04M017460 | 2.906589 | https://www.genecards.org/cgi-bin/carddisp.pl?gene=QDPR |
| BCKDHB | Branched Chain Keto Acid Dehydrogenase E1 Subunit Beta | Protein Coding | P21953 | 56 | GC06P080106 | 2.862356 | https://www.genecards.org/cgi-bin/carddisp.pl?gene=BCKDHB |
| MLYCD | Malonyl-CoA Decarboxylase | Protein Coding | O95822 | 55 | GC16P083899 | 2.8579 | https://www.genecards.org/cgi-bin/carddisp.pl?gene=MLYCD |
| MT-CO2 | Mitochondrially Encoded Cytochrome C Oxidase II | Protein Coding | P00403 | 49 | GCMTP007587 | 2.856747 | https://www.genecards.org/cgi-bin/carddisp.pl?gene=MT-CO2 |
| MAT1A | Methionine Adenosyltransferase 1A | Protein Coding | Q00266 | 60 | GC10M080271 | 2.82778 | https://www.genecards.org/cgi-bin/carddisp.pl?gene=MAT1A |
| SLC25A32 | Solute Carrier Family 25 Member 32 | Protein Coding | Q9H2D1 | 53 | GC08M103398 | 2.812227 | https://www.genecards.org/cgi-bin/carddisp.pl?gene=SLC25A32 |
| CPT1B | Carnitine Palmitoyltransferase 1B | Protein Coding | Q92523 | 58 | GC22M084041 | 2.809093 | https://www.genecards.org/cgi-bin/carddisp.pl?gene=CPT1B |
| RFK | Riboflavin Kinase | Protein Coding | Q969G6 | 51 | GC09M076385 | 2.79787 | https://www.genecards.org/cgi-bin/carddisp.pl?gene=RFK |
| INS | Insulin | Protein Coding | P01308 | 61 | GC11M002159 | 2.780034 | https://www.genecards.org/cgi-bin/carddisp.pl?gene=INS |
| FTCD | Formimidoyltransferase Cyclodeaminase | Protein Coding | O95954 | 53 | GC21M054604 | 2.762502 | https://www.genecards.org/cgi-bin/carddisp.pl?gene=FTCD |
| PNPLA2 | Patatin Like Phospholipase Domain Containing 2 | Protein Coding | Q96AD5 | 57 | GC11P015747 | 2.74806 | https://www.genecards.org/cgi-bin/carddisp.pl?gene=PNPLA2 |
| BTD | Biotinidase | Protein Coding | P43251 | 58 | GC03P025710 | 2.746597 | https://www.genecards.org/cgi-bin/carddisp.pl?gene=BTD |
| PPARA | Peroxisome Proliferator Activated Receptor Alpha | Protein Coding | Q07869 | 57 | GC22P046150 | 2.713141 | https://www.genecards.org/cgi-bin/carddisp.pl?gene=PPARA |
| ABHD5 | Abhydrolase Domain Containing 5, Lysophosphatidic Acid Acyltransferase | Protein Coding | Q8WTS1 | 58 | GC03P044154 | 2.687145 | https://www.genecards.org/cgi-bin/carddisp.pl?gene=ABHD5 |
| DECR1 | 2,4-Dienoyl-CoA Reductase 1 | Protein Coding | Q16698 | 53 | GC08P090001 | 2.675967 | https://www.genecards.org/cgi-bin/carddisp.pl?gene=DECR1 |
| HMGCL | 3-Hydroxy-3-Methylglutaryl-CoA Lyase | Protein Coding | P35914 | 59 | GC01M023801 | 2.670703 | https://www.genecards.org/cgi-bin/carddisp.pl?gene=HMGCL |
| BCS1L | BCS1 Homolog, Ubiquinol-Cytochrome C Reductase Complex Chaperone | Protein Coding | Q9Y276 | 57 | GC02P218658 | 2.670703 | https://www.genecards.org/cgi-bin/carddisp.pl?gene=BCS1L |
| BCKDHA | Branched Chain Keto Acid Dehydrogenase E1 Subunit Alpha | Protein Coding | P12694 | 56 | GC19P143009 | 2.670703 | https://www.genecards.org/cgi-bin/carddisp.pl?gene=BCKDHA |
| MOCS1 | Molybdenum Cofactor Synthesis 1 | Protein Coding | Q9NZB8 | 55 | GC06M039899 | 2.653093 | https://www.genecards.org/cgi-bin/carddisp.pl?gene=MOCS1 |
| TRMU | TRNA Mitochondrial 2-Thiouridylase | Protein Coding | O75648 | 52 | GC22P046330 | 2.653093 | https://www.genecards.org/cgi-bin/carddisp.pl?gene=TRMU |
| ETF1 | Eukaryotic Translation Termination Factor 1 | Protein Coding | P62495 | 52 | GC05M138506 | 2.621764 | https://www.genecards.org/cgi-bin/carddisp.pl?gene=ETF1 |
| ATAD1 | ATPase Family AAA Domain Containing 1 | Protein Coding | Q8NBU5 | 53 | GC10M087751 | 2.61396 | https://www.genecards.org/cgi-bin/carddisp.pl?gene=ATAD1 |
| OBSCN | Obscurin, Cytoskeletal Calmodulin And Titin-Interacting RhoGEF | Protein Coding | Q5VST9 | 53 | GC01P228208 | 2.61396 | https://www.genecards.org/cgi-bin/carddisp.pl?gene=OBSCN |
| PGM2L1 | Phosphoglucomutase 2 Like 1 | Protein Coding | Q6PCE3 | 50 | GC11M074330 | 2.61396 | https://www.genecards.org/cgi-bin/carddisp.pl?gene=PGM2L1 |
| SLCO1B1 | Solute Carrier Organic Anion Transporter Family Member 1B1 | Protein Coding | Q9Y6L6 | 59 | GC12P068706 | 2.608501 | https://www.genecards.org/cgi-bin/carddisp.pl?gene=SLCO1B1 |
| SUCLA2 | Succinate-CoA Ligase ADP-Forming Subunit Beta | Protein Coding | Q9P2R7 | 59 | GC13M047745 | 2.574353 | https://www.genecards.org/cgi-bin/carddisp.pl?gene=SUCLA2 |
| CHPT1 | Choline Phosphotransferase 1 | Protein Coding | Q8WUD6 | 49 | GC12P101696 | 2.558577 | https://www.genecards.org/cgi-bin/carddisp.pl?gene=CHPT1 |
| SLC19A3 | Solute Carrier Family 19 Member 3 | Protein Coding | Q9BZV2 | 59 | GC02M227685 | 2.530088 | https://www.genecards.org/cgi-bin/carddisp.pl?gene=SLC19A3 |
| PEX1 | Peroxisomal Biogenesis Factor 1 | Protein Coding | O43933 | 57 | GC07M092487 | 2.530088 | https://www.genecards.org/cgi-bin/carddisp.pl?gene=PEX1 |
| SURF1 | SURF1 Cytochrome C Oxidase Assembly Factor | Protein Coding | Q15526 | 56 | GC09M133351 | 2.530088 | https://www.genecards.org/cgi-bin/carddisp.pl?gene=SURF1 |
| MPV17 | Mitochondrial Inner Membrane Protein MPV17 | Protein Coding | P39210 | 53 | GC02M027309 | 2.530088 | https://www.genecards.org/cgi-bin/carddisp.pl?gene=MPV17 |
| MMAA | Metabolism Of Cobalamin Associated A | Protein Coding | Q8IVH4 | 52 | GC04P145652 | 2.529224 | https://www.genecards.org/cgi-bin/carddisp.pl?gene=MMAA |
| F10 | Coagulation Factor X | Protein Coding | P00742 | 63 | GC13P113122 | 2.497895 | https://www.genecards.org/cgi-bin/carddisp.pl?gene=F10 |
| GSR | Glutathione-Disulfide Reductase | Protein Coding | P00390 | 63 | GC08M030678 | 2.490458 | https://www.genecards.org/cgi-bin/carddisp.pl?gene=GSR |
| CRP | C-Reactive Protein | Protein Coding | P02741 | 59 | GC01M166465 | 2.490458 | https://www.genecards.org/cgi-bin/carddisp.pl?gene=CRP |
| TKT | Transketolase | Protein Coding | P29401 | 59 | GC03M053224 | 2.490458 | https://www.genecards.org/cgi-bin/carddisp.pl?gene=TKT |
| PLAGL1 | PLAG1 Like Zinc Finger 1 | Protein Coding | Q9UM63 | 56 | GC06M143940 | 2.490458 | https://www.genecards.org/cgi-bin/carddisp.pl?gene=PLAGL1 |
| TKTL2 | Transketolase Like 2 | Protein Coding | Q9H0I9 | 48 | GC04M163471 | 2.490458 | https://www.genecards.org/cgi-bin/carddisp.pl?gene=TKTL2 |
| RTBDN | Retbindin | Protein Coding | Q9BSG5 | 46 | GC19M104453 | 2.490458 | https://www.genecards.org/cgi-bin/carddisp.pl?gene=RTBDN |
| ACSF3 | Acyl-CoA Synthetase Family Member 3 | Protein Coding | Q4G176 | 56 | GC16P089088 | 2.459382 | https://www.genecards.org/cgi-bin/carddisp.pl?gene=ACSF3 |
| MTR | 5-Methyltetrahydrofolate-Homocysteine Methyltransferase | Protein Coding | Q99707 | 61 | GC01P236795 | 2.440983 | https://www.genecards.org/cgi-bin/carddisp.pl?gene=MTR |
| RYR1 | Ryanodine Receptor 1 | Protein Coding | P21817 | 62 | GC19P142895 | 2.416108 | https://www.genecards.org/cgi-bin/carddisp.pl?gene=RYR1 |
| AMPD1 | Adenosine Monophosphate Deaminase 1 | Protein Coding | P23109 | 59 | GC01M114673 | 2.416108 | https://www.genecards.org/cgi-bin/carddisp.pl?gene=AMPD1 |
| LPIN1 | Lipin 1 | Protein Coding | Q14693 | 59 | GC02P011677 | 2.416108 | https://www.genecards.org/cgi-bin/carddisp.pl?gene=LPIN1 |
| ANO5 | Anoctamin 5 | Protein Coding | Q75V66 | 50 | GC11P021799 | 2.416108 | https://www.genecards.org/cgi-bin/carddisp.pl?gene=ANO5 |
| TYMP | Thymidine Phosphorylase | Protein Coding | P19971 | 60 | GC22M050525 | 2.403202 | https://www.genecards.org/cgi-bin/carddisp.pl?gene=TYMP |
| OAT | Ornithine Aminotransferase | Protein Coding | P04181 | 61 | GC10M124397 | 2.363292 | https://www.genecards.org/cgi-bin/carddisp.pl?gene=OAT |
| POLG | DNA Polymerase Gamma, Catalytic Subunit | Protein Coding | P54098 | 61 | GC15M159705 | 2.363292 | https://www.genecards.org/cgi-bin/carddisp.pl?gene=POLG |
| TYR | Tyrosinase | Protein Coding | P14679 | 61 | GC11P089177 | 2.363292 | https://www.genecards.org/cgi-bin/carddisp.pl?gene=TYR |
| GATM | Glycine Amidinotransferase | Protein Coding | P50440 | 59 | GC15M045361 | 2.363292 | https://www.genecards.org/cgi-bin/carddisp.pl?gene=GATM |
| IDUA | Alpha-L-Iduronidase | Protein Coding | P35475 | 58 | GC04P000986 | 2.363292 | https://www.genecards.org/cgi-bin/carddisp.pl?gene=IDUA |
| NAGA | Alpha-N-Acetylgalactosaminidase | Protein Coding | P17050 | 58 | GC22M042058 | 2.363292 | https://www.genecards.org/cgi-bin/carddisp.pl?gene=NAGA |
| NAGLU | N-Acetyl-Alpha-Glucosaminidase | Protein Coding | P54802 | 58 | GC17P144465 | 2.363292 | https://www.genecards.org/cgi-bin/carddisp.pl?gene=NAGLU |
| NDUFV1 | NADH:Ubiquinone Oxidoreductase Core Subunit V1 | Protein Coding | P49821 | 58 | GC11P103503 | 2.363292 | https://www.genecards.org/cgi-bin/carddisp.pl?gene=NDUFV1 |
| CAV3 | Caveolin 3 | Protein Coding | P56539 | 57 | GC03P008733 | 2.363292 | https://www.genecards.org/cgi-bin/carddisp.pl?gene=CAV3 |
| LAMA2 | Laminin Subunit Alpha 2 | Protein Coding | P24043 | 57 | GC06P174242 | 2.363292 | https://www.genecards.org/cgi-bin/carddisp.pl?gene=LAMA2 |
| PCBD1 | Pterin-4 Alpha-Carbinolamine Dehydratase 1 | Protein Coding | P61457 | 57 | GC10M070882 | 2.363292 | https://www.genecards.org/cgi-bin/carddisp.pl?gene=PCBD1 |
| CYP21A2 | Cytochrome P450 Family 21 Subfamily A Member 2 | Protein Coding | P08686 | 56 | GC06P173102 | 2.363292 | https://www.genecards.org/cgi-bin/carddisp.pl?gene=CYP21A2 |
| SLC46A1 | Solute Carrier Family 46 Member 1 | Protein Coding | Q96NT5 | 55 | GC17M092927 | 2.363292 | https://www.genecards.org/cgi-bin/carddisp.pl?gene=SLC46A1 |
| MYOT | Myotilin | Protein Coding | Q9UBF9 | 53 | GC05P137867 | 2.363292 | https://www.genecards.org/cgi-bin/carddisp.pl?gene=MYOT |
| SLC25A44 | Solute Carrier Family 25 Member 44 | Protein Coding | Q96H78 | 45 | GC01P173541 | 2.352186 | https://www.genecards.org/cgi-bin/carddisp.pl?gene=SLC25A44 |
| PPARD | Peroxisome Proliferator Activated Receptor Delta | Protein Coding | Q03181 | 59 | GC06P173188 | 2.351486 | https://www.genecards.org/cgi-bin/carddisp.pl?gene=PPARD |
| PPP1R16A | Protein Phosphatase 1 Regulatory Subunit 16A | Protein Coding | Q96I34 | 41 | GC08P144477 | 2.351486 | https://www.genecards.org/cgi-bin/carddisp.pl?gene=PPP1R16A |
| CD40 | CD40 Molecule | Protein Coding | P25942 | 63 | GC20P046118 | 2.320683 | https://www.genecards.org/cgi-bin/carddisp.pl?gene=CD40 |
| CTSA | Cathepsin A | Protein Coding | P10619 | 60 | GC20P045890 | 2.320683 | https://www.genecards.org/cgi-bin/carddisp.pl?gene=CTSA |
| HINT1 | Histidine Triad Nucleotide Binding Protein 1 | Protein Coding | P49773 | 59 | GC05M131159 | 2.320683 | https://www.genecards.org/cgi-bin/carddisp.pl?gene=HINT1 |
| MYH11 | Myosin Heavy Chain 11 | Protein Coding | P35749 | 59 | GC16M047678 | 2.320683 | https://www.genecards.org/cgi-bin/carddisp.pl?gene=MYH11 |
| COL4A4 | Collagen Type IV Alpha 4 Chain | Protein Coding | P53420 | 56 | GC02M226973 | 2.320683 | https://www.genecards.org/cgi-bin/carddisp.pl?gene=COL4A4 |
| USH2A | Usherin | Protein Coding | O75445 | 51 | GC01M215622 | 2.320683 | https://www.genecards.org/cgi-bin/carddisp.pl?gene=USH2A |
| CS | Citrate Synthase | Protein Coding | O75390 | 58 | GC12M056271 | 2.317115 | https://www.genecards.org/cgi-bin/carddisp.pl?gene=CS |
| SLC25A3 | Solute Carrier Family 25 Member 3 | Protein Coding | Q00325 | 58 | GC12P098593 | 2.306243 | https://www.genecards.org/cgi-bin/carddisp.pl?gene=SLC25A3 |
| PLA2G6 | Phospholipase A2 Group VI | Protein Coding | O60733 | 61 | GC22M085492 | 2.256774 | https://www.genecards.org/cgi-bin/carddisp.pl?gene=PLA2G6 |
| COQ5 | Coenzyme Q5, Methyltransferase | Protein Coding | Q5HYK3 | 54 | GC12M120503 | 2.249331 | https://www.genecards.org/cgi-bin/carddisp.pl?gene=COQ5 |
| COQ3 | Coenzyme Q3, Methyltransferase | Protein Coding | Q9NZJ6 | 47 | GC06M099369 | 2.249331 | https://www.genecards.org/cgi-bin/carddisp.pl?gene=COQ3 |
| TYW1 | TRNA-YW Synthesizing Protein 1 Homolog | Protein Coding | Q9NV66 | 47 | GC07P066995 | 2.249331 | https://www.genecards.org/cgi-bin/carddisp.pl?gene=TYW1 |
| ADSL | Adenylosuccinate Lyase | Protein Coding | P30566 | 60 | GC22P040346 | 2.240905 | https://www.genecards.org/cgi-bin/carddisp.pl?gene=ADSL |
| SUCLG1 | Succinate-CoA Ligase GDP/ADP-Forming Subunit Alpha | Protein Coding | P53597 | 58 | GC02M084423 | 2.240905 | https://www.genecards.org/cgi-bin/carddisp.pl?gene=SUCLG1 |
| EIF5A | Eukaryotic Translation Initiation Factor 5A | Protein Coding | P63241 | 56 | GC17P007306 | 2.206722 | https://www.genecards.org/cgi-bin/carddisp.pl?gene=EIF5A |
| AMD1 | Adenosylmethionine Decarboxylase 1 | Protein Coding | P17707 | 53 | GC06P110814 | 2.206722 | https://www.genecards.org/cgi-bin/carddisp.pl?gene=AMD1 |
| PCCA | Propionyl-CoA Carboxylase Subunit Alpha | Protein Coding | P05165 | 60 | GC13P100089 | 2.196092 | https://www.genecards.org/cgi-bin/carddisp.pl?gene=PCCA |
| COASY | Coenzyme A Synthase | Protein Coding | Q13057 | 57 | GC17P042561 | 2.196092 | https://www.genecards.org/cgi-bin/carddisp.pl?gene=COASY |
| CYCS | Cytochrome C, Somatic | Protein Coding | P99999 | 61 | GC07M025118 | 2.193975 | https://www.genecards.org/cgi-bin/carddisp.pl?gene=CYCS |
| MGAM | Maltase-Glucoamylase | Protein Coding | O43451 | 57 | GC07P165315 | 2.179991 | https://www.genecards.org/cgi-bin/carddisp.pl?gene=MGAM |
| CKM | Creatine Kinase, M-Type | Protein Coding | P06732 | 58 | GC19M045306 | 2.173453 | https://www.genecards.org/cgi-bin/carddisp.pl?gene=CKM |
| MB | Myoglobin | Protein Coding | P02144 | 58 | GC22M035606 | 2.173453 | https://www.genecards.org/cgi-bin/carddisp.pl?gene=MB |
| CKB | Creatine Kinase B | Protein Coding | P12277 | 57 | GC14M103519 | 2.173453 | https://www.genecards.org/cgi-bin/carddisp.pl?gene=CKB |
| COQ6 | Coenzyme Q6, Monooxygenase | Protein Coding | Q9Y2Z9 | 56 | GC14P073949 | 2.173453 | https://www.genecards.org/cgi-bin/carddisp.pl?gene=COQ6 |
| CKMT2 | Creatine Kinase, Mitochondrial 2 | Protein Coding | P17540 | 54 | GC05P081233 | 2.173453 | https://www.genecards.org/cgi-bin/carddisp.pl?gene=CKMT2 |
| COQ2 | Coenzyme Q2, Polyprenyltransferase | Protein Coding | Q96H96 | 53 | GC04M083261 | 2.173453 | https://www.genecards.org/cgi-bin/carddisp.pl?gene=COQ2 |
| CKMT1B | Creatine Kinase, Mitochondrial 1B | Protein Coding | P12532 | 49 | GC15P184191 | 2.173453 | https://www.genecards.org/cgi-bin/carddisp.pl?gene=CKMT1B |
| MT-CYB | Mitochondrially Encoded Cytochrome B | Protein Coding | P00156 | 45 | GCMTP014749 | 2.173453 | https://www.genecards.org/cgi-bin/carddisp.pl?gene=MT-CYB |
| SCO2 | Synthesis Of Cytochrome C Oxidase 2 | Protein Coding | O43819 | 58 | GC22M050523 | 2.145919 | https://www.genecards.org/cgi-bin/carddisp.pl?gene=SCO2 |
| SUOX | Sulfite Oxidase | Protein Coding | P51687 | 57 | GC12P055997 | 2.145919 | https://www.genecards.org/cgi-bin/carddisp.pl?gene=SUOX |
| NDUFS4 | NADH:Ubiquinone Oxidoreductase Subunit S4 | Protein Coding | O43181 | 56 | GC05P053560 | 2.145919 | https://www.genecards.org/cgi-bin/carddisp.pl?gene=NDUFS4 |
| MMADHC | Metabolism Of Cobalamin Associated D | Protein Coding | Q9H3L0 | 55 | GC02M149569 | 2.145919 | https://www.genecards.org/cgi-bin/carddisp.pl?gene=MMADHC |
| SLC39A4 | Solute Carrier Family 39 Member 4 | Protein Coding | Q6P5W5 | 54 | GC08M144409 | 2.145919 | https://www.genecards.org/cgi-bin/carddisp.pl?gene=SLC39A4 |
| TMEM70 | Transmembrane Protein 70 | Protein Coding | Q9BUB7 | 51 | GC08P073972 | 2.145919 | https://www.genecards.org/cgi-bin/carddisp.pl?gene=TMEM70 |
| GAA | Alpha Glucosidase | Protein Coding | P10253 | 64 | GC17P080101 | 2.133833 | https://www.genecards.org/cgi-bin/carddisp.pl?gene=GAA |
| DNMT3B | DNA Methyltransferase 3 Beta | Protein Coding | Q9UBC3 | 64 | GC20P032762 | 2.128309 | https://www.genecards.org/cgi-bin/carddisp.pl?gene=DNMT3B |
| GSS | Glutathione Synthetase | Protein Coding | P48637 | 59 | GC20M034928 | 2.128309 | https://www.genecards.org/cgi-bin/carddisp.pl?gene=GSS |
| MAT2A | Methionine Adenosyltransferase 2A | Protein Coding | P31153 | 59 | GC02P087165 | 2.128309 | https://www.genecards.org/cgi-bin/carddisp.pl?gene=MAT2A |
| MTRR | 5-Methyltetrahydrofolate-Homocysteine Methyltransferase Reductase | Protein Coding | Q9UBK8 | 57 | GC05P007851 | 2.128309 | https://www.genecards.org/cgi-bin/carddisp.pl?gene=MTRR |
| DLG4 | Discs Large MAGUK Scaffold Protein 4 | Protein Coding | P78352 | 61 | GC17M092409 | 2.128096 | https://www.genecards.org/cgi-bin/carddisp.pl?gene=DLG4 |
| SLC22A4 | Solute Carrier Family 22 Member 4 | Protein Coding | Q9H015 | 58 | GC05P132294 | 2.128096 | https://www.genecards.org/cgi-bin/carddisp.pl?gene=SLC22A4 |
| SP1 | Sp1 Transcription Factor | Protein Coding | P08047 | 60 | GC12P053380 | 2.11459 | https://www.genecards.org/cgi-bin/carddisp.pl?gene=SP1 |
| FOXA1 | Forkhead Box A1 | Protein Coding | P55317 | 57 | GC14M037589 | 2.11459 | https://www.genecards.org/cgi-bin/carddisp.pl?gene=FOXA1 |
| DGUOK | Deoxyguanosine Kinase | Protein Coding | Q16854 | 56 | GC02P073926 | 2.049569 | https://www.genecards.org/cgi-bin/carddisp.pl?gene=DGUOK |
| TMLHE | Trimethyllysine Hydroxylase, Epsilon | Protein Coding | Q9NVH6 | 54 | GC0XM155489 | 2.049569 | https://www.genecards.org/cgi-bin/carddisp.pl?gene=TMLHE |
| TBCK | TBC1 Domain Containing Kinase | Protein Coding | Q8TEA7 | 52 | GC04M106041 | 2.049569 | https://www.genecards.org/cgi-bin/carddisp.pl?gene=TBCK |
| FAH | Fumarylacetoacetate Hydrolase | Protein Coding | P16930 | 59 | GC15P080152 | 2.00444 | https://www.genecards.org/cgi-bin/carddisp.pl?gene=FAH |
| DVL2 | Dishevelled Segment Polarity Protein 2 | Protein Coding | O14641 | 59 | GC17M007225 | 1.970585 | https://www.genecards.org/cgi-bin/carddisp.pl?gene=DVL2 |
| DDC | Dopa Decarboxylase | Protein Coding | P20711 | 63 | GC07M050458 | 1.953087 | https://www.genecards.org/cgi-bin/carddisp.pl?gene=DDC |
| ABCC8 | ATP Binding Cassette Subfamily C Member 8 | Protein Coding | Q09428 | 59 | GC11M017392 | 1.953087 | https://www.genecards.org/cgi-bin/carddisp.pl?gene=ABCC8 |
| ASL | Argininosuccinate Lyase | Protein Coding | P04424 | 58 | GC07P066075 | 1.953087 | https://www.genecards.org/cgi-bin/carddisp.pl?gene=ASL |
| PANK2 | Pantothenate Kinase 2 | Protein Coding | Q9BZ23 | 58 | GC20P010259 | 1.953087 | https://www.genecards.org/cgi-bin/carddisp.pl?gene=PANK2 |
| PPARGC1A | PPARG Coactivator 1 Alpha | Protein Coding | Q9UBK2 | 60 | GC04M023755 | 1.93381 | https://www.genecards.org/cgi-bin/carddisp.pl?gene=PPARGC1A |
| CXCL10 | C-X-C Motif Chemokine Ligand 10 | Protein Coding | P02778 | 57 | GC04M076021 | 1.922938 | https://www.genecards.org/cgi-bin/carddisp.pl?gene=CXCL10 |
| CACNA1S | Calcium Voltage-Gated Channel Subunit Alpha1 S | Protein Coding | Q13698 | 62 | GC01M201008 | 1.908953 | https://www.genecards.org/cgi-bin/carddisp.pl?gene=CACNA1S |
| ENO3 | Enolase 3 | Protein Coding | P13929 | 61 | GC17P004948 | 1.908953 | https://www.genecards.org/cgi-bin/carddisp.pl?gene=ENO3 |
| HMGCR | 3-Hydroxy-3-Methylglutaryl-CoA Reductase | Protein Coding | P04035 | 61 | GC05P075336 | 1.908953 | https://www.genecards.org/cgi-bin/carddisp.pl?gene=HMGCR |
| CYP2D6 | Cytochrome P450 Family 2 Subfamily D Member 6 (Gene/Pseudogene) | Protein Coding | P10635 | 60 | GC22M042126 | 1.908953 | https://www.genecards.org/cgi-bin/carddisp.pl?gene=CYP2D6 |
| MYOD1 | Myogenic Differentiation 1 | Protein Coding | P15172 | 60 | GC11P017719 | 1.908953 | https://www.genecards.org/cgi-bin/carddisp.pl?gene=MYOD1 |
| PIK3C2A | Phosphatidylinositol-4-Phosphate 3-Kinase Catalytic Subunit Type 2 Alpha | Protein Coding | O00443 | 60 | GC11M018080 | 1.908953 | https://www.genecards.org/cgi-bin/carddisp.pl?gene=PIK3C2A |
| ALDH18A1 | Aldehyde Dehydrogenase 18 Family Member A1 | Protein Coding | P54886 | 59 | GC10M095605 | 1.908953 | https://www.genecards.org/cgi-bin/carddisp.pl?gene=ALDH18A1 |
| ARG2 | Arginase 2 | Protein Coding | P78540 | 59 | GC14P067619 | 1.908953 | https://www.genecards.org/cgi-bin/carddisp.pl?gene=ARG2 |
| CYP3A5 | Cytochrome P450 Family 3 Subfamily A Member 5 | Protein Coding | P20815 | 59 | GC07M099648 | 1.908953 | https://www.genecards.org/cgi-bin/carddisp.pl?gene=CYP3A5 |
| PYGL | Glycogen Phosphorylase L | Protein Coding | P06737 | 59 | GC14M050857 | 1.908953 | https://www.genecards.org/cgi-bin/carddisp.pl?gene=PYGL |
| ADH5 | Alcohol Dehydrogenase 5 (Class III), Chi Polypeptide | Protein Coding | P11766 | 58 | GC04M099070 | 1.908953 | https://www.genecards.org/cgi-bin/carddisp.pl?gene=ADH5 |
| SI | Sucrase-Isomaltase | Protein Coding | P14410 | 57 | GC03M164978 | 1.908953 | https://www.genecards.org/cgi-bin/carddisp.pl?gene=SI |
| PGAM2 | Phosphoglycerate Mutase 2 | Protein Coding | P15259 | 56 | GC07M044062 | 1.908953 | https://www.genecards.org/cgi-bin/carddisp.pl?gene=PGAM2 |
| TRPM6 | Transient Receptor Potential Cation Channel Subfamily M Member 6 | Protein Coding | Q9BX84 | 56 | GC09M074725 | 1.908953 | https://www.genecards.org/cgi-bin/carddisp.pl?gene=TRPM6 |
| CCS | Copper Chaperone For Superoxide Dismutase | Protein Coding | O14618 | 55 | GC11P066593 | 1.908953 | https://www.genecards.org/cgi-bin/carddisp.pl?gene=CCS |
| COX6A1 | Cytochrome C Oxidase Subunit 6A1 | Protein Coding | P12074 | 55 | GC12P120519 | 1.908953 | https://www.genecards.org/cgi-bin/carddisp.pl?gene=COX6A1 |
| HCCS | Holocytochrome C Synthase | Protein Coding | P53701 | 55 | GC0XP011111 | 1.908953 | https://www.genecards.org/cgi-bin/carddisp.pl?gene=HCCS |
| MTO1 | Mitochondrial TRNA Translation Optimization 1 | Protein Coding | Q9Y2Z2 | 55 | GC06P073461 | 1.908953 | https://www.genecards.org/cgi-bin/carddisp.pl?gene=MTO1 |
| PAX7 | Paired Box 7 | Protein Coding | P23759 | 55 | GC01P068595 | 1.908953 | https://www.genecards.org/cgi-bin/carddisp.pl?gene=PAX7 |
| SBDS | SBDS Ribosome Maturation Factor | Protein Coding | Q9Y3A5 | 55 | GC07M066987 | 1.908953 | https://www.genecards.org/cgi-bin/carddisp.pl?gene=SBDS |
| COQ9 | Coenzyme Q9 | Protein Coding | O75208 | 54 | GC16P057447 | 1.908953 | https://www.genecards.org/cgi-bin/carddisp.pl?gene=COQ9 |
| RRAGC | Ras Related GTP Binding C | Protein Coding | Q9HB90 | 54 | GC01M039032 | 1.908953 | https://www.genecards.org/cgi-bin/carddisp.pl?gene=RRAGC |
| RTEL1 | Regulator Of Telomere Elongation Helicase 1 | Protein Coding | Q9NZ71 | 54 | GC20P068672 | 1.908953 | https://www.genecards.org/cgi-bin/carddisp.pl?gene=RTEL1 |
| EXOSC9 | Exosome Component 9 | Protein Coding | Q06265 | 53 | GC04P121801 | 1.908953 | https://www.genecards.org/cgi-bin/carddisp.pl?gene=EXOSC9 |
| FBXO32 | F-Box Protein 32 | Protein Coding | Q969P5 | 53 | GC08M123631 | 1.908953 | https://www.genecards.org/cgi-bin/carddisp.pl?gene=FBXO32 |
| GFM2 | GTP Dependent Ribosome Recycling Factor Mitochondrial 2 | Protein Coding | Q969S9 | 53 | GC05M074721 | 1.908953 | https://www.genecards.org/cgi-bin/carddisp.pl?gene=GFM2 |
| NDUFAF1 | NADH:Ubiquinone Oxidoreductase Complex Assembly Factor 1 | Protein Coding | Q9Y375 | 53 | GC15M041387 | 1.908953 | https://www.genecards.org/cgi-bin/carddisp.pl?gene=NDUFAF1 |
| PPCDC | Phosphopantothenoylcysteine Decarboxylase | Protein Coding | Q96CD2 | 53 | GC15P075023 | 1.908953 | https://www.genecards.org/cgi-bin/carddisp.pl?gene=PPCDC |
| UTRN | Utrophin | Protein Coding | P46939 | 53 | GC06P144285 | 1.908953 | https://www.genecards.org/cgi-bin/carddisp.pl?gene=UTRN |
| DNAL1 | Dynein Axonemal Light Chain 1 | Protein Coding | Q4LDG9 | 52 | GC14P073644 | 1.908953 | https://www.genecards.org/cgi-bin/carddisp.pl?gene=DNAL1 |
| SMN2 | Survival Of Motor Neuron 2, Centromeric | Protein Coding | Q16637 | 52 | GC05P070049 | 1.908953 | https://www.genecards.org/cgi-bin/carddisp.pl?gene=SMN2 |
| SYNE2 | Spectrin Repeat Containing Nuclear Envelope Protein 2 | Protein Coding | Q8WXH0 | 52 | GC14P063761 | 1.908953 | https://www.genecards.org/cgi-bin/carddisp.pl?gene=SYNE2 |
| TBX6 | T-Box Transcription Factor 6 | Protein Coding | O95947 | 52 | GC16M030085 | 1.908953 | https://www.genecards.org/cgi-bin/carddisp.pl?gene=TBX6 |
| AP1M1 | Adaptor Related Protein Complex 1 Subunit Mu 1 | Protein Coding | Q9BXS5 | 51 | GC19P142496 | 1.908953 | https://www.genecards.org/cgi-bin/carddisp.pl?gene=AP1M1 |
| ECSIT | ECSIT Signaling Integrator | Protein Coding | Q9BQ95 | 50 | GC19M011505 | 1.908953 | https://www.genecards.org/cgi-bin/carddisp.pl?gene=ECSIT |
| TBC1D23 | TBC1 Domain Family Member 23 | Protein Coding | Q9NUY8 | 50 | GC03P100260 | 1.908953 | https://www.genecards.org/cgi-bin/carddisp.pl?gene=TBC1D23 |
| TMEM126B | Transmembrane Protein 126B | Protein Coding | Q8IUX1 | 50 | GC11P085628 | 1.908953 | https://www.genecards.org/cgi-bin/carddisp.pl?gene=TMEM126B |
| KCNG4 | Potassium Voltage-Gated Channel Modifier Subfamily G Member 4 | Protein Coding | Q8TDN1 | 49 | GC16M084255 | 1.908953 | https://www.genecards.org/cgi-bin/carddisp.pl?gene=KCNG4 |
| MRRF | Mitochondrial Ribosome Recycling Factor | Protein Coding | Q96E11 | 49 | GC09P122264 | 1.908953 | https://www.genecards.org/cgi-bin/carddisp.pl?gene=MRRF |
| SERAC1 | Serine Active Site Containing 1 | Protein Coding | Q96JX3 | 49 | GC06M158109 | 1.908953 | https://www.genecards.org/cgi-bin/carddisp.pl?gene=SERAC1 |
| SQOR | Sulfide Quinone Oxidoreductase | Protein Coding | Q9Y6N5 | 49 | GC15P184238 | 1.908953 | https://www.genecards.org/cgi-bin/carddisp.pl?gene=SQOR |
| LILRB5 | Leukocyte Immunoglobulin Like Receptor B5 | Protein Coding | O75023 | 48 | GC19M105484 | 1.908953 | https://www.genecards.org/cgi-bin/carddisp.pl?gene=LILRB5 |
| SHROOM4 | Shroom Family Member 4 | Protein Coding | Q9ULL8 | 48 | GC0XM050576 | 1.908953 | https://www.genecards.org/cgi-bin/carddisp.pl?gene=SHROOM4 |
| ACAD10 | Acyl-CoA Dehydrogenase Family Member 10 | Protein Coding | Q6JQN1 | 47 | GC12P111686 | 1.908953 | https://www.genecards.org/cgi-bin/carddisp.pl?gene=ACAD10 |
| AHDC1 | AT-Hook DNA Binding Motif Containing 1 | Protein Coding | Q5TGY3 | 47 | GC01M027534 | 1.908953 | https://www.genecards.org/cgi-bin/carddisp.pl?gene=AHDC1 |
| CEP89 | Centrosomal Protein 89 | Protein Coding | Q96ST8 | 47 | GC19M104852 | 1.908953 | https://www.genecards.org/cgi-bin/carddisp.pl?gene=CEP89 |
| TMTC3 | Transmembrane O-Mannosyltransferase Targeting Cadherins 3 | Protein Coding | Q6ZXV5 | 47 | GC12P088142 | 1.908953 | https://www.genecards.org/cgi-bin/carddisp.pl?gene=TMTC3 |
| ACAD11 | Acyl-CoA Dehydrogenase Family Member 11 | Protein Coding | Q709F0 | 46 | GC03M132559 | 1.908953 | https://www.genecards.org/cgi-bin/carddisp.pl?gene=ACAD11 |
| TAF1A | TATA-Box Binding Protein Associated Factor, RNA Polymerase I Subunit A | Protein Coding | Q15573 | 46 | GC01M227252 | 1.908953 | https://www.genecards.org/cgi-bin/carddisp.pl?gene=TAF1A |
| ALDH16A1 | Aldehyde Dehydrogenase 16 Family Member A1 | Protein Coding | Q8IZ83 | 45 | GC19P049453 | 1.908953 | https://www.genecards.org/cgi-bin/carddisp.pl?gene=ALDH16A1 |
| CFAP44 | Cilia And Flagella Associated Protein 44 | Protein Coding | Q96MT7 | 45 | GC03M113286 | 1.908953 | https://www.genecards.org/cgi-bin/carddisp.pl?gene=CFAP44 |
| PRR12 | Proline Rich 12 | Protein Coding | Q9ULL5 | 43 | GC19P049591 | 1.908953 | https://www.genecards.org/cgi-bin/carddisp.pl?gene=PRR12 |
| ZCCHC12 | Zinc Finger CCHC-Type Containing 12 | Protein Coding | Q6PEW1 | 43 | GC0XP118823 | 1.908953 | https://www.genecards.org/cgi-bin/carddisp.pl?gene=ZCCHC12 |
| ZCCHC13 | Zinc Finger CCHC-Type Containing 13 | Protein Coding | Q8WW36 | 43 | GC0XP074304 | 1.908953 | https://www.genecards.org/cgi-bin/carddisp.pl?gene=ZCCHC13 |
| NAA11 | N-Alpha-Acetyltransferase 11, NatA Catalytic Subunit | Protein Coding | Q9BSU3 | 42 | GC04M079155 | 1.908953 | https://www.genecards.org/cgi-bin/carddisp.pl?gene=NAA11 |
| MSGN1 | Mesogenin 1 | Protein Coding | A6NI15 | 41 | GC02P017817 | 1.908953 | https://www.genecards.org/cgi-bin/carddisp.pl?gene=MSGN1 |
| FADS6 | Fatty Acid Desaturase 6 | Protein Coding | Q8N9I5 | 40 | GC17M094251 | 1.908953 | https://www.genecards.org/cgi-bin/carddisp.pl?gene=FADS6 |
| ABCA1 | ATP Binding Cassette Subfamily A Member 1 | Protein Coding | O95477 | 61 | GC09M104781 | 1.892172 | https://www.genecards.org/cgi-bin/carddisp.pl?gene=ABCA1 |
| MYBPC3 | Myosin Binding Protein C3 | Protein Coding | Q14896 | 60 | GC11M136236 | 1.892172 | https://www.genecards.org/cgi-bin/carddisp.pl?gene=MYBPC3 |
| SLC17A5 | Solute Carrier Family 17 Member 5 | Protein Coding | Q9NRA2 | 58 | GC06M073593 | 1.892172 | https://www.genecards.org/cgi-bin/carddisp.pl?gene=SLC17A5 |
| SLC25A15 | Solute Carrier Family 25 Member 15 | Protein Coding | Q9Y619 | 57 | GC13P040789 | 1.892172 | https://www.genecards.org/cgi-bin/carddisp.pl?gene=SLC25A15 |
| HBA1 | Hemoglobin Subunit Alpha 1 | Protein Coding | P69905 | 56 | GC16P112211 | 1.878417 | https://www.genecards.org/cgi-bin/carddisp.pl?gene=HBA1 |
| HBA2 | Hemoglobin Subunit Alpha 2 | Protein Coding | P69905 | 53 | GC16P112212 | 1.878417 | https://www.genecards.org/cgi-bin/carddisp.pl?gene=HBA2 |
| ADA | Adenosine Deaminase | Protein Coding | P00813 | 64 | GC20M044620 | 1.812787 | https://www.genecards.org/cgi-bin/carddisp.pl?gene=ADA |
| LPL | Lipoprotein Lipase | Protein Coding | P06858 | 63 | GC08P019901 | 1.812787 | https://www.genecards.org/cgi-bin/carddisp.pl?gene=LPL |
| ASS1 | Argininosuccinate Synthase 1 | Protein Coding | P00966 | 62 | GC09P130444 | 1.812787 | https://www.genecards.org/cgi-bin/carddisp.pl?gene=ASS1 |
| GLDC | Glycine Decarboxylase | Protein Coding | P23378 | 60 | GC09M006522 | 1.812787 | https://www.genecards.org/cgi-bin/carddisp.pl?gene=GLDC |
| PDP1 | Pyruvate Dehydrogenase Phosphatase Catalytic Subunit 1 | Protein Coding | Q9P0J1 | 60 | GC08P093857 | 1.812787 | https://www.genecards.org/cgi-bin/carddisp.pl?gene=PDP1 |
| MYH6 | Myosin Heavy Chain 6 | Protein Coding | P13533 | 59 | GC14M023381 | 1.812787 | https://www.genecards.org/cgi-bin/carddisp.pl?gene=MYH6 |
| MTTP | Microsomal Triglyceride Transfer Protein | Protein Coding | P55157 | 57 | GC04P099563 | 1.812787 | https://www.genecards.org/cgi-bin/carddisp.pl?gene=MTTP |
| CYP17A1 | Cytochrome P450 Family 17 Subfamily A Member 1 | Protein Coding | P05093 | 63 | GC10M102830 | 1.801882 | https://www.genecards.org/cgi-bin/carddisp.pl?gene=CYP17A1 |
| CYP11A1 | Cytochrome P450 Family 11 Subfamily A Member 1 | Protein Coding | P05108 | 61 | GC15M074337 | 1.801882 | https://www.genecards.org/cgi-bin/carddisp.pl?gene=CYP11A1 |
| PFKM | Phosphofructokinase, Muscle | Protein Coding | P08237 | 61 | GC12P048105 | 1.801882 | https://www.genecards.org/cgi-bin/carddisp.pl?gene=PFKM |
| POR | Cytochrome P450 Oxidoreductase | Protein Coding | P16435 | 61 | GC07P075899 | 1.801882 | https://www.genecards.org/cgi-bin/carddisp.pl?gene=POR |
| PYGM | Glycogen Phosphorylase, Muscle Associated | Protein Coding | P11217 | 60 | GC11M064746 | 1.801882 | https://www.genecards.org/cgi-bin/carddisp.pl?gene=PYGM |
| AGL | Amylo-Alpha-1,6-Glucosidase And 4-Alpha-Glucanotransferase | Protein Coding | P35573 | 59 | GC01P099850 | 1.801882 | https://www.genecards.org/cgi-bin/carddisp.pl?gene=AGL |
| DMD | Dystrophin | Protein Coding | P11532 | 58 | GC0XM031097 | 1.801882 | https://www.genecards.org/cgi-bin/carddisp.pl?gene=DMD |
| HSD3B2 | Hydroxy-Delta-5-Steroid Dehydrogenase, 3 Beta- And Steroid Delta-Isomerase 2 | Protein Coding | P26439 | 58 | GC01P119414 | 1.801882 | https://www.genecards.org/cgi-bin/carddisp.pl?gene=HSD3B2 |
| STAR | Steroidogenic Acute Regulatory Protein | Protein Coding | P49675 | 58 | GC08M038792 | 1.801882 | https://www.genecards.org/cgi-bin/carddisp.pl?gene=STAR |
| DYSF | Dysferlin | Protein Coding | O75923 | 56 | GC02P071453 | 1.801882 | https://www.genecards.org/cgi-bin/carddisp.pl?gene=DYSF |
| FKRP | Fukutin Related Protein | Protein Coding | Q9H9S5 | 52 | GC19P143178 | 1.801882 | https://www.genecards.org/cgi-bin/carddisp.pl?gene=FKRP |
| DHFR | Dihydrofolate Reductase | Protein Coding | P00374 | 61 | GC05M080626 | 1.742157 | https://www.genecards.org/cgi-bin/carddisp.pl?gene=DHFR |
| SDHA | Succinate Dehydrogenase Complex Flavoprotein Subunit A | Protein Coding | P31040 | 61 | GC05P000238 | 1.742157 | https://www.genecards.org/cgi-bin/carddisp.pl?gene=SDHA |
| NDUFS3 | NADH:Ubiquinone Oxidoreductase Core Subunit S3 | Protein Coding | O75489 | 59 | GC11P048705 | 1.742157 | https://www.genecards.org/cgi-bin/carddisp.pl?gene=NDUFS3 |
| AARS2 | Alanyl-TRNA Synthetase 2, Mitochondrial | Protein Coding | Q5JTZ9 | 57 | GC06M103592 | 1.742157 | https://www.genecards.org/cgi-bin/carddisp.pl?gene=AARS2 |
| ACY1 | Aminoacylase 1 | Protein Coding | Q03154 | 57 | GC03P051983 | 1.742157 | https://www.genecards.org/cgi-bin/carddisp.pl?gene=ACY1 |
| BCKDK | Branched Chain Keto Acid Dehydrogenase Kinase | Protein Coding | O14874 | 57 | GC16P113202 | 1.742157 | https://www.genecards.org/cgi-bin/carddisp.pl?gene=BCKDK |
| NDUFS2 | NADH:Ubiquinone Oxidoreductase Core Subunit S2 | Protein Coding | O75306 | 57 | GC01P161197 | 1.742157 | https://www.genecards.org/cgi-bin/carddisp.pl?gene=NDUFS2 |
| PRODH | Proline Dehydrogenase 1 | Protein Coding | O43272 | 57 | GC22M018912 | 1.742157 | https://www.genecards.org/cgi-bin/carddisp.pl?gene=PRODH |
| APTX | Aprataxin | Protein Coding | Q7Z2E3 | 56 | GC09M032886 | 1.742157 | https://www.genecards.org/cgi-bin/carddisp.pl?gene=APTX |
| COQ7 | Coenzyme Q7, Hydroxylase | Protein Coding | Q99807 | 56 | GC16P019067 | 1.742157 | https://www.genecards.org/cgi-bin/carddisp.pl?gene=COQ7 |
| COQ8A | Coenzyme Q8A | Protein Coding | Q8NI60 | 56 | GC01P226942 | 1.742157 | https://www.genecards.org/cgi-bin/carddisp.pl?gene=COQ8A |
| D2HGDH | D-2-Hydroxyglutarate Dehydrogenase | Protein Coding | Q8N465 | 56 | GC02P241734 | 1.742157 | https://www.genecards.org/cgi-bin/carddisp.pl?gene=D2HGDH |
| NDUFA9 | NADH:Ubiquinone Oxidoreductase Subunit A9 | Protein Coding | Q16795 | 56 | GC12P004649 | 1.742157 | https://www.genecards.org/cgi-bin/carddisp.pl?gene=NDUFA9 |
| NDUFV2 | NADH:Ubiquinone Oxidoreductase Core Subunit V2 | Protein Coding | P19404 | 56 | GC18P009114 | 1.742157 | https://www.genecards.org/cgi-bin/carddisp.pl?gene=NDUFV2 |
| SARDH | Sarcosine Dehydrogenase | Protein Coding | Q9UL12 | 56 | GC09M133663 | 1.742157 | https://www.genecards.org/cgi-bin/carddisp.pl?gene=SARDH |
| EHHADH | Enoyl-CoA Hydratase And 3-Hydroxyacyl CoA Dehydrogenase | Protein Coding | Q08426 | 55 | GC03M185190 | 1.742157 | https://www.genecards.org/cgi-bin/carddisp.pl?gene=EHHADH |
| ACAA2 | Acetyl-CoA Acyltransferase 2 | Protein Coding | P42765 | 54 | GC18M049782 | 1.742157 | https://www.genecards.org/cgi-bin/carddisp.pl?gene=ACAA2 |
| PDSS1 | Decaprenyl Diphosphate Synthase Subunit 1 | Protein Coding | Q5T2R2 | 54 | GC10P026697 | 1.742157 | https://www.genecards.org/cgi-bin/carddisp.pl?gene=PDSS1 |
| PDSS2 | Decaprenyl Diphosphate Synthase Subunit 2 | Protein Coding | Q86YH6 | 54 | GC06M107152 | 1.742157 | https://www.genecards.org/cgi-bin/carddisp.pl?gene=PDSS2 |
| SLC6A12 | Solute Carrier Family 6 Member 12 | Protein Coding | P48065 | 54 | GC12M000183 | 1.742157 | https://www.genecards.org/cgi-bin/carddisp.pl?gene=SLC6A12 |
| COQ8B | Coenzyme Q8B | Protein Coding | Q96D53 | 53 | GC19M104976 | 1.742157 | https://www.genecards.org/cgi-bin/carddisp.pl?gene=COQ8B |
| DMGDH | Dimethylglycine Dehydrogenase | Protein Coding | Q9UI17 | 53 | GC05M078997 | 1.742157 | https://www.genecards.org/cgi-bin/carddisp.pl?gene=DMGDH |
| NDUFA5 | NADH:Ubiquinone Oxidoreductase Subunit A5 | Protein Coding | Q16718 | 53 | GC07M123536 | 1.742157 | https://www.genecards.org/cgi-bin/carddisp.pl?gene=NDUFA5 |
| NDUFB11 | NADH:Ubiquinone Oxidoreductase Subunit B11 | Protein Coding | Q9NX14 | 53 | GC0XM047142 | 1.742157 | https://www.genecards.org/cgi-bin/carddisp.pl?gene=NDUFB11 |
| PDP2 | Pyruvate Dehydrogenase Phosphatase Catalytic Subunit 2 | Protein Coding | Q9P2J9 | 53 | GC16P113936 | 1.742157 | https://www.genecards.org/cgi-bin/carddisp.pl?gene=PDP2 |
| CBY1 | Chibby 1, Beta Catenin Antagonist | Protein Coding | Q9Y3M2 | 52 | GC22P038656 | 1.742157 | https://www.genecards.org/cgi-bin/carddisp.pl?gene=CBY1 |
| COQ4 | Coenzyme Q4 | Protein Coding | Q9Y3A0 | 52 | GC09P128322 | 1.742157 | https://www.genecards.org/cgi-bin/carddisp.pl?gene=COQ4 |
| UROC1 | Urocanate Hydratase 1 | Protein Coding | Q96N76 | 52 | GC03M126481 | 1.742157 | https://www.genecards.org/cgi-bin/carddisp.pl?gene=UROC1 |
| DNAH8 | Dynein Axonemal Heavy Chain 8 | Protein Coding | Q96JB1 | 50 | GC06P173224 | 1.742157 | https://www.genecards.org/cgi-bin/carddisp.pl?gene=DNAH8 |
| SLC25A42 | Solute Carrier Family 25 Member 42 | Protein Coding | Q86VD7 | 50 | GC19P019063 | 1.742157 | https://www.genecards.org/cgi-bin/carddisp.pl?gene=SLC25A42 |
| SUGCT | Succinyl-CoA:Glutarate-CoA Transferase | Protein Coding | Q9HAC7 | 50 | GC07P041295 | 1.742157 | https://www.genecards.org/cgi-bin/carddisp.pl?gene=SUGCT |
| ACOT11 | Acyl-CoA Thioesterase 11 | Protein Coding | Q8WXI4 | 49 | GC01P054542 | 1.742157 | https://www.genecards.org/cgi-bin/carddisp.pl?gene=ACOT11 |
| CIR1 | Corepressor Interacting With RBPJ, CIR1 | Protein Coding | Q86X95 | 48 | GC02M174348 | 1.742157 | https://www.genecards.org/cgi-bin/carddisp.pl?gene=CIR1 |
| COQ10A | Coenzyme Q10A | Protein Coding | Q96MF6 | 47 | GC12P056266 | 1.742157 | https://www.genecards.org/cgi-bin/carddisp.pl?gene=COQ10A |
| SDHAF1 | Succinate Dehydrogenase Complex Assembly Factor 1 | Protein Coding | A6NFY7 | 47 | GC19P035995 | 1.742157 | https://www.genecards.org/cgi-bin/carddisp.pl?gene=SDHAF1 |
| ZFAND6 | Zinc Finger AN1-Type Containing 6 | Protein Coding | Q6FIF0 | 47 | GC15P184875 | 1.742157 | https://www.genecards.org/cgi-bin/carddisp.pl?gene=ZFAND6 |
| DHFR2 | Dihydrofolate Reductase 2 | Protein Coding | Q86XF0 | 46 | GC03M094048 | 1.742157 | https://www.genecards.org/cgi-bin/carddisp.pl?gene=DHFR2 |
| MT-ND1 | Mitochondrially Encoded NADH:Ubiquinone Oxidoreductase Core Subunit 1 | Protein Coding | P03886 | 46 | GCMTP003309 | 1.742157 | https://www.genecards.org/cgi-bin/carddisp.pl?gene=MT-ND1 |
| MT-CO3 | Mitochondrially Encoded Cytochrome C Oxidase III | Protein Coding | P00414 | 43 | GCMTP009209 | 1.742157 | https://www.genecards.org/cgi-bin/carddisp.pl?gene=MT-CO3 |
| ETFBKMT | Electron Transfer Flavoprotein Subunit Beta Lysine Methyltransferase | Protein Coding | Q8IXQ9 | 42 | GC12P068994 | 1.742157 | https://www.genecards.org/cgi-bin/carddisp.pl?gene=ETFBKMT |
| POLR1F | RNA Polymerase I Subunit F | Protein Coding | Q3B726 | 42 | GC07M019695 | 1.742157 | https://www.genecards.org/cgi-bin/carddisp.pl?gene=POLR1F |
| ETFRF1 | Electron Transfer Flavoprotein Regulatory Factor 1 | Protein Coding | Q6IPR1 | 41 | GC12P068779 | 1.742157 | https://www.genecards.org/cgi-bin/carddisp.pl?gene=ETFRF1 |
| KTI12 | KTI12 Chromatin Associated Homolog | Protein Coding | Q96EK9 | 40 | GC01M052032 | 1.742157 | https://www.genecards.org/cgi-bin/carddisp.pl?gene=KTI12 |
| SLC25A53 | Solute Carrier Family 25 Member 53 | Protein Coding | Q5H9E4 | 39 | GC0XM104099 | 1.742157 | https://www.genecards.org/cgi-bin/carddisp.pl?gene=SLC25A53 |
| SMIM11 | Small Integral Membrane Protein 11 | Protein Coding | P58511 | 39 | GC21P034375 | 1.742157 | https://www.genecards.org/cgi-bin/carddisp.pl?gene=SMIM11 |
| NBPF4 | NBPF Member 4 | Protein Coding | Q96M43 | 36 | GC01M108223 | 1.742157 | https://www.genecards.org/cgi-bin/carddisp.pl?gene=NBPF4 |
| ANKRD61 | Ankyrin Repeat Domain 61 | Protein Coding | A6NGH8 | 35 | GC07P006031 | 1.742157 | https://www.genecards.org/cgi-bin/carddisp.pl?gene=ANKRD61 |
| LOC102723553 | Small Integral Membrane Protein 11B | Protein Coding | | 11 | GC21P007744 | 1.742157 | https://www.genecards.org/cgi-bin/carddisp.pl?gene=LOC102723553 |
| RRM1 | Ribonucleotide Reductase Catalytic Subunit M1 | Protein Coding | P23921 | 63 | GC11P015892 | 1.699548 | https://www.genecards.org/cgi-bin/carddisp.pl?gene=RRM1 |
| ABCC1 | ATP Binding Cassette Subfamily C Member 1 (ABCC1 Blood Group) | Protein Coding | P33527 | 61 | GC16P015949 | 1.699548 | https://www.genecards.org/cgi-bin/carddisp.pl?gene=ABCC1 |
| NFATC1 | Nuclear Factor Of Activated T Cells 1 | Protein Coding | O95644 | 61 | GC18P079395 | 1.699548 | https://www.genecards.org/cgi-bin/carddisp.pl?gene=NFATC1 |
| SLC12A5 | Solute Carrier Family 12 Member 5 | Protein Coding | Q9H2X9 | 61 | GC20P046021 | 1.699548 | https://www.genecards.org/cgi-bin/carddisp.pl?gene=SLC12A5 |
| ABCC6 | ATP Binding Cassette Subfamily C Member 6 | Protein Coding | O95255 | 60 | GC16M047689 | 1.699548 | https://www.genecards.org/cgi-bin/carddisp.pl?gene=ABCC6 |
| CA12 | Carbonic Anhydrase 12 | Protein Coding | O43570 | 60 | GC15M063321 | 1.699548 | https://www.genecards.org/cgi-bin/carddisp.pl?gene=CA12 |
| CDK9 | Cyclin Dependent Kinase 9 | Protein Coding | P50750 | 60 | GC09P149740 | 1.699548 | https://www.genecards.org/cgi-bin/carddisp.pl?gene=CDK9 |
| FECH | Ferrochelatase | Protein Coding | P22830 | 60 | GC18M057544 | 1.699548 | https://www.genecards.org/cgi-bin/carddisp.pl?gene=FECH |
| IRF1 | Interferon Regulatory Factor 1 | Protein Coding | P10914 | 60 | GC05M132440 | 1.699548 | https://www.genecards.org/cgi-bin/carddisp.pl?gene=IRF1 |
| MAPKAPK2 | MAPK Activated Protein Kinase 2 | Protein Coding | P49137 | 60 | GC01P206684 | 1.699548 | https://www.genecards.org/cgi-bin/carddisp.pl?gene=MAPKAPK2 |
| CSF2 | Colony Stimulating Factor 2 | Protein Coding | P04141 | 59 | GC05P132073 | 1.699548 | https://www.genecards.org/cgi-bin/carddisp.pl?gene=CSF2 |
| PPP2R2A | Protein Phosphatase 2 Regulatory Subunit Balpha | Protein Coding | P63151 | 59 | GC08P026292 | 1.699548 | https://www.genecards.org/cgi-bin/carddisp.pl?gene=PPP2R2A |
| IKBKE | Inhibitor Of Nuclear Factor Kappa B Kinase Subunit Epsilon | Protein Coding | Q14164 | 58 | GC01P206470 | 1.699548 | https://www.genecards.org/cgi-bin/carddisp.pl?gene=IKBKE |
| CDK19 | Cyclin Dependent Kinase 19 | Protein Coding | Q9BWU1 | 57 | GC06M110609 | 1.699548 | https://www.genecards.org/cgi-bin/carddisp.pl?gene=CDK19 |
| CELF2 | CUGBP Elav-Like Family Member 2 | Protein Coding | O95319 | 57 | GC10P010462 | 1.699548 | https://www.genecards.org/cgi-bin/carddisp.pl?gene=CELF2 |
| FZD3 | Frizzled Class Receptor 3 | Protein Coding | Q9NPG1 | 57 | GC08P028494 | 1.699548 | https://www.genecards.org/cgi-bin/carddisp.pl?gene=FZD3 |
| KDM5B | Lysine Demethylase 5B | Protein Coding | Q9UGL1 | 57 | GC01M202696 | 1.699548 | https://www.genecards.org/cgi-bin/carddisp.pl?gene=KDM5B |
| NARS1 | Asparaginyl-TRNA Synthetase 1 | Protein Coding | O43776 | 57 | GC18M057601 | 1.699548 | https://www.genecards.org/cgi-bin/carddisp.pl?gene=NARS1 |
| P4HA2 | Prolyl 4-Hydroxylase Subunit Alpha 2 | Protein Coding | O15460 | 57 | GC05M132193 | 1.699548 | https://www.genecards.org/cgi-bin/carddisp.pl?gene=P4HA2 |
| PLTP | Phospholipid Transfer Protein | Protein Coding | P55058 | 57 | GC20M045898 | 1.699548 | https://www.genecards.org/cgi-bin/carddisp.pl?gene=PLTP |
| PPID | Peptidylprolyl Isomerase D | Protein Coding | Q08752 | 57 | GC04M158709 | 1.699548 | https://www.genecards.org/cgi-bin/carddisp.pl?gene=PPID |
| SOX5 | SRY-Box Transcription Factor 5 | Protein Coding | P35711 | 57 | GC12M023529 | 1.699548 | https://www.genecards.org/cgi-bin/carddisp.pl?gene=SOX5 |
| USP1 | Ubiquitin Specific Peptidase 1 | Protein Coding | O94782 | 57 | GC01P062436 | 1.699548 | https://www.genecards.org/cgi-bin/carddisp.pl?gene=USP1 |
| HRH4 | Histamine Receptor H4 | Protein Coding | Q9H3N8 | 56 | GC18P024460 | 1.699548 | https://www.genecards.org/cgi-bin/carddisp.pl?gene=HRH4 |
| NFIA | Nuclear Factor I A | Protein Coding | Q12857 | 56 | GC01P060865 | 1.699548 | https://www.genecards.org/cgi-bin/carddisp.pl?gene=NFIA |
| PKD2L1 | Polycystin 2 Like 1, Transient Receptor Potential Cation Channel | Protein Coding | Q9P0L9 | 56 | GC10M100288 | 1.699548 | https://www.genecards.org/cgi-bin/carddisp.pl?gene=PKD2L1 |
| PPP1R3A | Protein Phosphatase 1 Regulatory Subunit 3A | Protein Coding | Q16821 | 56 | GC07M113876 | 1.699548 | https://www.genecards.org/cgi-bin/carddisp.pl?gene=PPP1R3A |
| SLC1A7 | Solute Carrier Family 1 Member 7 | Protein Coding | O00341 | 56 | GC01M053087 | 1.699548 | https://www.genecards.org/cgi-bin/carddisp.pl?gene=SLC1A7 |
| UBE2C | Ubiquitin Conjugating Enzyme E2 C | Protein Coding | O00762 | 56 | GC20P045812 | 1.699548 | https://www.genecards.org/cgi-bin/carddisp.pl?gene=UBE2C |
| CACNA2D2 | Calcium Voltage-Gated Channel Auxiliary Subunit Alpha2delta 2 | Protein Coding | Q9NY47 | 55 | GC03M054579 | 1.699548 | https://www.genecards.org/cgi-bin/carddisp.pl?gene=CACNA2D2 |
| PDGFD | Platelet Derived Growth Factor D | Protein Coding | Q9GZP0 | 55 | GC11M103907 | 1.699548 | https://www.genecards.org/cgi-bin/carddisp.pl?gene=PDGFD |
| ACOT7 | Acyl-CoA Thioesterase 7 | Protein Coding | O00154 | 54 | GC01M020776 | 1.699548 | https://www.genecards.org/cgi-bin/carddisp.pl?gene=ACOT7 |
| GPAM | Glycerol-3-Phosphate Acyltransferase, Mitochondrial | Protein Coding | Q9HCL2 | 54 | GC10M112148 | 1.699548 | https://www.genecards.org/cgi-bin/carddisp.pl?gene=GPAM |
| NDE1 | NudE Neurodevelopment Protein 1 | Protein Coding | Q9NXR1 | 54 | GC16P112598 | 1.699548 | https://www.genecards.org/cgi-bin/carddisp.pl?gene=NDE1 |
| PIGR | Polymeric Immunoglobulin Receptor | Protein Coding | P01833 | 54 | GC01M206928 | 1.699548 | https://www.genecards.org/cgi-bin/carddisp.pl?gene=PIGR |
| PTPRT | Protein Tyrosine Phosphatase Receptor Type T | Protein Coding | O14522 | 54 | GC20M042377 | 1.699548 | https://www.genecards.org/cgi-bin/carddisp.pl?gene=PTPRT |
| RAMP1 | Receptor Activity Modifying Protein 1 | Protein Coding | O60894 | 54 | GC02P237858 | 1.699548 | https://www.genecards.org/cgi-bin/carddisp.pl?gene=RAMP1 |
| IL24 | Interleukin 24 | Protein Coding | Q13007 | 53 | GC01P206897 | 1.699548 | https://www.genecards.org/cgi-bin/carddisp.pl?gene=IL24 |
| PIGT | Phosphatidylinositol Glycan Anchor Biosynthesis Class T | Protein Coding | Q969N2 | 53 | GC20P045416 | 1.699548 | https://www.genecards.org/cgi-bin/carddisp.pl?gene=PIGT |
| PREX1 | Phosphatidylinositol-3,4,5-Trisphosphate Dependent Rac Exchange Factor 1 | Protein Coding | Q8TCU6 | 53 | GC20M048624 | 1.699548 | https://www.genecards.org/cgi-bin/carddisp.pl?gene=PREX1 |
| SLC6A11 | Solute Carrier Family 6 Member 11 | Protein Coding | P48066 | 53 | GC03P025582 | 1.699548 | https://www.genecards.org/cgi-bin/carddisp.pl?gene=SLC6A11 |
| TP53RK | TP53 Regulating Kinase | Protein Coding | Q96S44 | 53 | GC20M046684 | 1.699548 | https://www.genecards.org/cgi-bin/carddisp.pl?gene=TP53RK |
| WNT8B | Wnt Family Member 8B | Protein Coding | Q93098 | 53 | GC10P100463 | 1.699548 | https://www.genecards.org/cgi-bin/carddisp.pl?gene=WNT8B |
| ELF3 | E74 Like ETS Transcription Factor 3 | Protein Coding | P78545 | 52 | GC01P202007 | 1.699548 | https://www.genecards.org/cgi-bin/carddisp.pl?gene=ELF3 |
| GPR37L1 | G Protein-Coupled Receptor 37 Like 1 | Protein Coding | O60883 | 52 | GC01P202122 | 1.699548 | https://www.genecards.org/cgi-bin/carddisp.pl?gene=GPR37L1 |
| HERC1 | HECT And RLD Domain Containing E3 Ubiquitin Protein Ligase Family Member 1 | Protein Coding | Q15751 | 52 | GC15M063608 | 1.699548 | https://www.genecards.org/cgi-bin/carddisp.pl?gene=HERC1 |
| IL19 | Interleukin 19 | Protein Coding | Q9UHD0 | 52 | GC01P206770 | 1.699548 | https://www.genecards.org/cgi-bin/carddisp.pl?gene=IL19 |
| LIN7A | Lin-7 Homolog A, Crumbs Cell Polarity Complex Component | Protein Coding | O14910 | 52 | GC12M080792 | 1.699548 | https://www.genecards.org/cgi-bin/carddisp.pl?gene=LIN7A |
| RNPEP | Arginyl Aminopeptidase | Protein Coding | Q9H4A4 | 52 | GC01P201982 | 1.699548 | https://www.genecards.org/cgi-bin/carddisp.pl?gene=RNPEP |
| CADM2 | Cell Adhesion Molecule 2 | Protein Coding | Q8N3J6 | 51 | GC03P084979 | 1.699548 | https://www.genecards.org/cgi-bin/carddisp.pl?gene=CADM2 |
| CCN3 | Cellular Communication Network Factor 3 | Protein Coding | P48745 | 51 | GC08P119416 | 1.699548 | https://www.genecards.org/cgi-bin/carddisp.pl?gene=CCN3 |
| RARRES2 | Retinoic Acid Receptor Responder 2 | Protein Coding | Q99969 | 51 | GC07M150333 | 1.699548 | https://www.genecards.org/cgi-bin/carddisp.pl?gene=RARRES2 |
| SULF2 | Sulfatase 2 | Protein Coding | Q8IWU5 | 51 | GC20M047656 | 1.699548 | https://www.genecards.org/cgi-bin/carddisp.pl?gene=SULF2 |
| DDO | D-Aspartate Oxidase | Protein Coding | Q99489 | 50 | GC06M110391 | 1.699548 | https://www.genecards.org/cgi-bin/carddisp.pl?gene=DDO |
| IL20 | Interleukin 20 | Protein Coding | Q9NYY1 | 50 | GC01P206866 | 1.699548 | https://www.genecards.org/cgi-bin/carddisp.pl?gene=IL20 |
| MYRF | Myelin Regulatory Factor | Protein Coding | Q9Y2G1 | 50 | GC11P103021 | 1.699548 | https://www.genecards.org/cgi-bin/carddisp.pl?gene=MYRF |
| TNNC2 | Troponin C2, Fast Skeletal Type | Protein Coding | P02585 | 50 | GC20M045823 | 1.699548 | https://www.genecards.org/cgi-bin/carddisp.pl?gene=TNNC2 |
| USP3 | Ubiquitin Specific Peptidase 3 | Protein Coding | Q9Y6I4 | 50 | GC15P063504 | 1.699548 | https://www.genecards.org/cgi-bin/carddisp.pl?gene=USP3 |
| ZNF335 | Zinc Finger Protein 335 | Protein Coding | Q9H4Z2 | 50 | GC20M045948 | 1.699548 | https://www.genecards.org/cgi-bin/carddisp.pl?gene=ZNF335 |
| PHYKPL | 5-Phosphohydroxy-L-Lysine Phospho-Lyase | Protein Coding | Q8IUZ5 | 49 | GC05M178207 | 1.699548 | https://www.genecards.org/cgi-bin/carddisp.pl?gene=PHYKPL |
| RASSF5 | Ras Association Domain Family Member 5 | Protein Coding | Q8WWW0 | 49 | GC01P206507 | 1.699548 | https://www.genecards.org/cgi-bin/carddisp.pl?gene=RASSF5 |
| SLC16A9 | Solute Carrier Family 16 Member 9 | Protein Coding | Q7RTY1 | 49 | GC10M059650 | 1.699548 | https://www.genecards.org/cgi-bin/carddisp.pl?gene=SLC16A9 |
| THEM4 | Thioesterase Superfamily Member 4 | Protein Coding | Q5T1C6 | 49 | GC01M151870 | 1.699548 | https://www.genecards.org/cgi-bin/carddisp.pl?gene=THEM4 |
| UBE2F | Ubiquitin Conjugating Enzyme E2 F (Putative) | Protein Coding | Q969M7 | 49 | GC02P238037 | 1.699548 | https://www.genecards.org/cgi-bin/carddisp.pl?gene=UBE2F |
| ACOT8 | Acyl-CoA Thioesterase 8 | Protein Coding | O14734 | 48 | GC20M045841 | 1.699548 | https://www.genecards.org/cgi-bin/carddisp.pl?gene=ACOT8 |
| AFAP1L2 | Actin Filament Associated Protein 1 Like 2 | Protein Coding | Q8N4X5 | 48 | GC10M114281 | 1.699548 | https://www.genecards.org/cgi-bin/carddisp.pl?gene=AFAP1L2 |
| SEMA4G | Semaphorin 4G | Protein Coding | Q9NTN9 | 48 | GC10P100969 | 1.699548 | https://www.genecards.org/cgi-bin/carddisp.pl?gene=SEMA4G |
| SLC35C2 | Solute Carrier Family 35 Member C2 | Protein Coding | Q9NQQ7 | 48 | GC20M046345 | 1.699548 | https://www.genecards.org/cgi-bin/carddisp.pl?gene=SLC35C2 |
| ZNF746 | Zinc Finger Protein 746 | Protein Coding | Q6NUN9 | 48 | GC07M149472 | 1.699548 | https://www.genecards.org/cgi-bin/carddisp.pl?gene=ZNF746 |
| CHSY3 | Chondroitin Sulfate Synthase 3 | Protein Coding | Q70JA7 | 47 | GC05P129904 | 1.699548 | https://www.genecards.org/cgi-bin/carddisp.pl?gene=CHSY3 |
| EIF2D | Eukaryotic Translation Initiation Factor 2D | Protein Coding | P41214 | 47 | GC01M206571 | 1.699548 | https://www.genecards.org/cgi-bin/carddisp.pl?gene=EIF2D |
| MACROD2 | Mono-ADP Ribosylhydrolase 2 | Protein Coding | A1Z1Q3 | 47 | GC20P013995 | 1.699548 | https://www.genecards.org/cgi-bin/carddisp.pl?gene=MACROD2 |
| MYO16 | Myosin XVI | Protein Coding | Q9Y6X6 | 47 | GC13P108495 | 1.699548 | https://www.genecards.org/cgi-bin/carddisp.pl?gene=MYO16 |
| SLC22A16 | Solute Carrier Family 22 Member 16 | Protein Coding | Q86VW1 | 47 | GC06M110424 | 1.699548 | https://www.genecards.org/cgi-bin/carddisp.pl?gene=SLC22A16 |
| TM2D1 | TM2 Domain Containing 1 | Protein Coding | Q9BX74 | 47 | GC01M061681 | 1.699548 | https://www.genecards.org/cgi-bin/carddisp.pl?gene=TM2D1 |
| VWA2 | Von Willebrand Factor A Domain Containing 2 | Protein Coding | Q5GFL6 | 47 | GC10P114239 | 1.699548 | https://www.genecards.org/cgi-bin/carddisp.pl?gene=VWA2 |
| ZNF521 | Zinc Finger Protein 521 | Protein Coding | Q96K83 | 47 | GC18M025061 | 1.699548 | https://www.genecards.org/cgi-bin/carddisp.pl?gene=ZNF521 |
| BLOC1S2 | Biogenesis Of Lysosomal Organelles Complex 1 Subunit 2 | Protein Coding | Q6QNY1 | 46 | GC10M100273 | 1.699548 | https://www.genecards.org/cgi-bin/carddisp.pl?gene=BLOC1S2 |
| FCMR | Fc Mu Receptor | Protein Coding | O60667 | 46 | GC01M206904 | 1.699548 | https://www.genecards.org/cgi-bin/carddisp.pl?gene=FCMR |
| MARCHF8 | Membrane Associated Ring-CH-Type Finger 8 | Protein Coding | Q5T0T0 | 46 | GC10M049085 | 1.699548 | https://www.genecards.org/cgi-bin/carddisp.pl?gene=MARCHF8 |
| PRP4K | Pre-MRNA Processing Factor Kinase PRP4K | Protein Coding | Q13523 | 46 | GC06P004021 | 1.699548 | https://www.genecards.org/cgi-bin/carddisp.pl?gene=PRP4K |
| RPF2 | Ribosome Production Factor 2 Homolog | Protein Coding | Q9H7B2 | 46 | GC06P110982 | 1.699548 | https://www.genecards.org/cgi-bin/carddisp.pl?gene=RPF2 |
| SLC22A23 | Solute Carrier Family 22 Member 23 | Protein Coding | A1A5C7 | 46 | GC06M004085 | 1.699548 | https://www.genecards.org/cgi-bin/carddisp.pl?gene=SLC22A23 |
| SLC35B1 | Solute Carrier Family 35 Member B1 | Protein Coding | P78383 | 46 | GC17M049700 | 1.699548 | https://www.genecards.org/cgi-bin/carddisp.pl?gene=SLC35B1 |
| TDRD1 | Tudor Domain Containing 1 | Protein Coding | Q9BXT4 | 46 | GC10P120816 | 1.699548 | https://www.genecards.org/cgi-bin/carddisp.pl?gene=TDRD1 |
| TOMM34 | Translocase Of Outer Mitochondrial Membrane 34 | Protein Coding | Q15785 | 46 | GC20M044942 | 1.699548 | https://www.genecards.org/cgi-bin/carddisp.pl?gene=TOMM34 |
| CDC42SE2 | CDC42 Small Effector 2 | Protein Coding | Q9NRR3 | 45 | GC05P131212 | 1.699548 | https://www.genecards.org/cgi-bin/carddisp.pl?gene=CDC42SE2 |
| FCAMR | Fc Alpha And Mu Receptor | Protein Coding | Q8WWV6 | 45 | GC01M206957 | 1.699548 | https://www.genecards.org/cgi-bin/carddisp.pl?gene=FCAMR |
| NAA30 | N-Alpha-Acetyltransferase 30, NatC Catalytic Subunit | Protein Coding | Q147X3 | 45 | GC14P057390 | 1.699548 | https://www.genecards.org/cgi-bin/carddisp.pl?gene=NAA30 |
| STON2 | Stonin 2 | Protein Coding | Q8WXE9 | 45 | GC14M081260 | 1.699548 | https://www.genecards.org/cgi-bin/carddisp.pl?gene=STON2 |
| USP6NL | USP6 N-Terminal Like | Protein Coding | Q92738 | 45 | GC10M011453 | 1.699548 | https://www.genecards.org/cgi-bin/carddisp.pl?gene=USP6NL |
| ABLIM1 | Actin Binding LIM Protein 1 | Protein Coding | O14639 | 44 | GC10M114564 | 1.699548 | https://www.genecards.org/cgi-bin/carddisp.pl?gene=ABLIM1 |
| CEP20 | Centrosomal Protein 20 | Protein Coding | Q96NB1 | 44 | GC16M047684 | 1.699548 | https://www.genecards.org/cgi-bin/carddisp.pl?gene=CEP20 |
| COL23A1 | Collagen Type XXIII Alpha 1 Chain | Protein Coding | Q86Y22 | 44 | GC05M178237 | 1.699548 | https://www.genecards.org/cgi-bin/carddisp.pl?gene=COL23A1 |
| FAM13C | Family With Sequence Similarity 13 Member C | Protein Coding | Q8NE31 | 44 | GC10M059246 | 1.699548 | https://www.genecards.org/cgi-bin/carddisp.pl?gene=FAM13C |
| LUZP2 | Leucine Zipper Protein 2 | Protein Coding | Q86TE4 | 44 | GC11P024518 | 1.699548 | https://www.genecards.org/cgi-bin/carddisp.pl?gene=LUZP2 |
| NCOA5 | Nuclear Receptor Coactivator 5 | Protein Coding | Q9HCD5 | 44 | GC20M046060 | 1.699548 | https://www.genecards.org/cgi-bin/carddisp.pl?gene=NCOA5 |
| NEURL2 | Neuralized E3 Ubiquitin Protein Ligase 2 | Protein Coding | Q9BR09 | 44 | GC20M045888 | 1.699548 | https://www.genecards.org/cgi-bin/carddisp.pl?gene=NEURL2 |
| WFDC3 | WAP Four-Disulfide Core Domain 3 | Protein Coding | Q8IUB2 | 44 | GC20M045747 | 1.699548 | https://www.genecards.org/cgi-bin/carddisp.pl?gene=WFDC3 |
| DNTTIP1 | Deoxynucleotidyltransferase Terminal Interacting Protein 1 | Protein Coding | Q9H147 | 43 | GC20P045791 | 1.699548 | https://www.genecards.org/cgi-bin/carddisp.pl?gene=DNTTIP1 |
| FBXL22 | F-Box And Leucine Rich Repeat Protein 22 | Protein Coding | Q6P050 | 43 | GC15P063597 | 1.699548 | https://www.genecards.org/cgi-bin/carddisp.pl?gene=FBXL22 |
| GTF2A1 | General Transcription Factor IIA Subunit 1 | Protein Coding | P52655 | 43 | GC14M081175 | 1.699548 | https://www.genecards.org/cgi-bin/carddisp.pl?gene=GTF2A1 |
| RAPGEF6 | Rap Guanine Nucleotide Exchange Factor 6 | Protein Coding | Q8TEU7 | 43 | GC05M131423 | 1.699548 | https://www.genecards.org/cgi-bin/carddisp.pl?gene=RAPGEF6 |
| STOML3 | Stomatin Like 3 | Protein Coding | Q8TAV4 | 43 | GC13M038965 | 1.699548 | https://www.genecards.org/cgi-bin/carddisp.pl?gene=STOML3 |
| PSMG4 | Proteasome Assembly Chaperone 4 | Protein Coding | Q5JS54 | 42 | GC06P003231 | 1.699548 | https://www.genecards.org/cgi-bin/carddisp.pl?gene=PSMG4 |
| RHBDD1 | Rhomboid Domain Containing 1 | Protein Coding | Q8TEB9 | 42 | GC02P226896 | 1.699548 | https://www.genecards.org/cgi-bin/carddisp.pl?gene=RHBDD1 |
| TMEM258 | Transmembrane Protein 258 | Protein Coding | P61165 | 42 | GC11M061768 | 1.699548 | https://www.genecards.org/cgi-bin/carddisp.pl?gene=TMEM258 |
| UBXN2A | UBX Domain Protein 2A | Protein Coding | P68543 | 42 | GC02P023927 | 1.699548 | https://www.genecards.org/cgi-bin/carddisp.pl?gene=UBXN2A |
| GTF3C6 | General Transcription Factor IIIC Subunit 6 | Protein Coding | Q969F1 | 41 | GC06P110958 | 1.699548 | https://www.genecards.org/cgi-bin/carddisp.pl?gene=GTF3C6 |
| KLHL29 | Kelch Like Family Member 29 | Protein Coding | Q96CT2 | 41 | GC02P023385 | 1.699548 | https://www.genecards.org/cgi-bin/carddisp.pl?gene=KLHL29 |
| MAL2 | Mal, T Cell Differentiation Protein 2 | Protein Coding | Q969L2 | 41 | GC08P119165 | 1.699548 | https://www.genecards.org/cgi-bin/carddisp.pl?gene=MAL2 |
| PCIF1 | Phosphorylated CTD Interacting Factor 1 | Protein Coding | Q9H4Z3 | 41 | GC20P045934 | 1.699548 | https://www.genecards.org/cgi-bin/carddisp.pl?gene=PCIF1 |
| BMERB1 | BMERB Domain Containing 1 | Protein Coding | Q96MC5 | 40 | GC16P015434 | 1.699548 | https://www.genecards.org/cgi-bin/carddisp.pl?gene=BMERB1 |
| KRBA1 | KRAB-A Domain Containing 1 | Protein Coding | A5PL33 | 40 | GC07P149714 | 1.699548 | https://www.genecards.org/cgi-bin/carddisp.pl?gene=KRBA1 |
| TDRP | Testis Development Related Protein | Protein Coding | Q86YL5 | 40 | GC08M002905 | 1.699548 | https://www.genecards.org/cgi-bin/carddisp.pl?gene=TDRP |
| SNX21 | Sorting Nexin Family Member 21 | Protein Coding | Q969T3 | 39 | GC20P045833 | 1.699548 | https://www.genecards.org/cgi-bin/carddisp.pl?gene=SNX21 |
| ZNF267 | Zinc Finger Protein 267 | Protein Coding | Q14586 | 39 | GC16P113254 | 1.699548 | https://www.genecards.org/cgi-bin/carddisp.pl?gene=ZNF267 |
| CCDC198 | Coiled-Coil Domain Containing 198 | Protein Coding | Q9NVL8 | 38 | GC14M057470 | 1.699548 | https://www.genecards.org/cgi-bin/carddisp.pl?gene=CCDC198 |
| ERICH1 | Glutamate Rich 1 | Protein Coding | Q86X53 | 38 | GC08M000614 | 1.699548 | https://www.genecards.org/cgi-bin/carddisp.pl?gene=ERICH1 |
| METTL24 | Methyltransferase Like 24 | Protein Coding | Q5JXM2 | 38 | GC06M110245 | 1.699548 | https://www.genecards.org/cgi-bin/carddisp.pl?gene=METTL24 |
| RSBN1L | Round Spermatid Basic Protein 1 Like | Protein Coding | Q6PCB5 | 38 | GC07P077696 | 1.699548 | https://www.genecards.org/cgi-bin/carddisp.pl?gene=RSBN1L |
| SYS1 | SYS1 Golgi Trafficking Protein | Protein Coding | Q8N2H4 | 38 | GC20P045361 | 1.699548 | https://www.genecards.org/cgi-bin/carddisp.pl?gene=SYS1 |
| ZSWIM3 | Zinc Finger SWIM-Type Containing 3 | Protein Coding | Q96MP5 | 38 | GC20P045857 | 1.699548 | https://www.genecards.org/cgi-bin/carddisp.pl?gene=ZSWIM3 |
| SPATA25 | Spermatogenesis Associated 25 | Protein Coding | Q9BR10 | 37 | GC20M045886 | 1.699548 | https://www.genecards.org/cgi-bin/carddisp.pl?gene=SPATA25 |
| PXDC1 | PX Domain Containing 1 | Protein Coding | Q5TGL8 | 36 | GC06M004044 | 1.699548 | https://www.genecards.org/cgi-bin/carddisp.pl?gene=PXDC1 |
| MEIKIN | Meiotic Kinetochore Factor | Protein Coding | A0A087WXM9 | 34 | GC05M131806 | 1.699548 | https://www.genecards.org/cgi-bin/carddisp.pl?gene=MEIKIN |
| OR11G2 | Olfactory Receptor Family 11 Subfamily G Member 2 | Protein Coding | Q8NGC1 | 33 | GC14P054122 | 1.699548 | https://www.genecards.org/cgi-bin/carddisp.pl?gene=OR11G2 |
| SMIM30 | Small Integral Membrane Protein 30 | Protein Coding | A4D0T7 | 27 | GC07M113117 | 1.699548 | https://www.genecards.org/cgi-bin/carddisp.pl?gene=SMIM30 |
| SLCO1B3-SLCO1B7 | SLCO1B3-SLCO1B7 Readthrough | Protein Coding | F5H094 | 23 | GC12P068702 | 1.699548 | https://www.genecards.org/cgi-bin/carddisp.pl?gene=SLCO1B3-SLCO1B7 |
| SSU72L5 | SSU72 Like 5 | Protein Coding | A0A1W2PQ64 | 15 | GC11P004233 | 1.699548 | https://www.genecards.org/cgi-bin/carddisp.pl?gene=SSU72L5 |
| ENSG00000283782 | Novel Protein | Protein Coding | | 10 | GC05P132608 | 1.699548 | https://www.genecards.org/cgi-bin/carddisp.pl?gene=ENSG00000283782 |
| LOC124904333 | Octapeptide-Repeat Protein T2 | Protein Coding | | 7 | GC18P079419 | 1.699548 | https://www.genecards.org/cgi-bin/carddisp.pl?gene=LOC124904333 |
| LOC124902894 | Putative Solute Carrier Organic Anion Transporter Family Member 1B7 | Protein Coding | | 5 | GC12P071245 | 1.699548 | https://www.genecards.org/cgi-bin/carddisp.pl?gene=LOC124902894 |
| HIBCH | 3-Hydroxyisobutyryl-CoA Hydrolase | Protein Coding | Q6NVY1 | 56 | GC02M190189 | 1.663904 | https://www.genecards.org/cgi-bin/carddisp.pl?gene=HIBCH |
| BRAF | B-Raf Proto-Oncogene, Serine/Threonine Kinase | Protein Coding | P15056 | 67 | GC07M140788 | 1.621135 | https://www.genecards.org/cgi-bin/carddisp.pl?gene=BRAF |
| BTK | Bruton Tyrosine Kinase | Protein Coding | Q06187 | 67 | GC0XM101349 | 1.621135 | https://www.genecards.org/cgi-bin/carddisp.pl?gene=BTK |
| IKBKB | Inhibitor Of Nuclear Factor Kappa B Kinase Subunit Beta | Protein Coding | O14920 | 67 | GC08P042271 | 1.621135 | https://www.genecards.org/cgi-bin/carddisp.pl?gene=IKBKB |
| MAP2K1 | Mitogen-Activated Protein Kinase Kinase 1 | Protein Coding | Q02750 | 67 | GC15P066386 | 1.621135 | https://www.genecards.org/cgi-bin/carddisp.pl?gene=MAP2K1 |
| MAP2K2 | Mitogen-Activated Protein Kinase Kinase 2 | Protein Coding | P36507 | 67 | GC19M004090 | 1.621135 | https://www.genecards.org/cgi-bin/carddisp.pl?gene=MAP2K2 |
| PTPN11 | Protein Tyrosine Phosphatase Non-Receptor Type 11 | Protein Coding | Q06124 | 67 | GC12P112418 | 1.621135 | https://www.genecards.org/cgi-bin/carddisp.pl?gene=PTPN11 |
| RAF1 | Raf-1 Proto-Oncogene, Serine/Threonine Kinase | Protein Coding | P04049 | 67 | GC03M012583 | 1.621135 | https://www.genecards.org/cgi-bin/carddisp.pl?gene=RAF1 |
| SMAD3 | SMAD Family Member 3 | Protein Coding | P84022 | 67 | GC15P067063 | 1.621135 | https://www.genecards.org/cgi-bin/carddisp.pl?gene=SMAD3 |
| TGFBR1 | Transforming Growth Factor Beta Receptor 1 | Protein Coding | P36897 | 67 | GC09P105622 | 1.621135 | https://www.genecards.org/cgi-bin/carddisp.pl?gene=TGFBR1 |
| ATM | ATM Serine/Threonine Kinase | Protein Coding | Q13315 | 66 | GC11P108223 | 1.621135 | https://www.genecards.org/cgi-bin/carddisp.pl?gene=ATM |
| CFTR | CF Transmembrane Conductance Regulator | Protein Coding | P13569 | 66 | GC07P117287 | 1.621135 | https://www.genecards.org/cgi-bin/carddisp.pl?gene=CFTR |
| CTSD | Cathepsin D | Protein Coding | P07339 | 66 | GC11M001752 | 1.621135 | https://www.genecards.org/cgi-bin/carddisp.pl?gene=CTSD |
| KRAS | KRAS Proto-Oncogene, GTPase | Protein Coding | P01116 | 66 | GC12M034710 | 1.621135 | https://www.genecards.org/cgi-bin/carddisp.pl?gene=KRAS |
| NOTCH1 | Notch Receptor 1 | Protein Coding | P46531 | 66 | GC09M139117 | 1.621135 | https://www.genecards.org/cgi-bin/carddisp.pl?gene=NOTCH1 |
| RPS6KA3 | Ribosomal Protein S6 Kinase A3 | Protein Coding | P51812 | 66 | GC0XM020149 | 1.621135 | https://www.genecards.org/cgi-bin/carddisp.pl?gene=RPS6KA3 |
| SMAD2 | SMAD Family Member 2 | Protein Coding | Q15796 | 66 | GC18M047809 | 1.621135 | https://www.genecards.org/cgi-bin/carddisp.pl?gene=SMAD2 |
| SMAD4 | SMAD Family Member 4 | Protein Coding | Q13485 | 66 | GC18P051028 | 1.621135 | https://www.genecards.org/cgi-bin/carddisp.pl?gene=SMAD4 |
| HRAS | HRas Proto-Oncogene, GTPase | Protein Coding | P01112 | 65 | GC11M013956 | 1.621135 | https://www.genecards.org/cgi-bin/carddisp.pl?gene=HRAS |
| JAK3 | Janus Kinase 3 | Protein Coding | P52333 | 65 | GC19M017824 | 1.621135 | https://www.genecards.org/cgi-bin/carddisp.pl?gene=JAK3 |
| LCK | LCK Proto-Oncogene, Src Family Tyrosine Kinase | Protein Coding | P06239 | 65 | GC01P032251 | 1.621135 | https://www.genecards.org/cgi-bin/carddisp.pl?gene=LCK |
| MYLK | Myosin Light Chain Kinase | Protein Coding | Q15746 | 65 | GC03M123610 | 1.621135 | https://www.genecards.org/cgi-bin/carddisp.pl?gene=MYLK |
| PTPRC | Protein Tyrosine Phosphatase Receptor Type C | Protein Coding | P08575 | 65 | GC01P198607 | 1.621135 | https://www.genecards.org/cgi-bin/carddisp.pl?gene=PTPRC |
| TGFBR2 | Transforming Growth Factor Beta Receptor 2 | Protein Coding | P37173 | 65 | GC03P030608 | 1.621135 | https://www.genecards.org/cgi-bin/carddisp.pl?gene=TGFBR2 |
| CBL | Cbl Proto-Oncogene | Protein Coding | P22681 | 64 | GC11P119206 | 1.621135 | https://www.genecards.org/cgi-bin/carddisp.pl?gene=CBL |
| DPYD | Dihydropyrimidine Dehydrogenase | Protein Coding | Q12882 | 64 | GC01M097015 | 1.621135 | https://www.genecards.org/cgi-bin/carddisp.pl?gene=DPYD |
| LDLR | Low Density Lipoprotein Receptor | Protein Coding | P01130 | 64 | GC19P142248 | 1.621135 | https://www.genecards.org/cgi-bin/carddisp.pl?gene=LDLR |
| NRAS | NRAS Proto-Oncogene, GTPase | Protein Coding | P01111 | 64 | GC01M114704 | 1.621135 | https://www.genecards.org/cgi-bin/carddisp.pl?gene=NRAS |
| SOS1 | SOS Ras/Rac Guanine Nucleotide Exchange Factor 1 | Protein Coding | Q07889 | 64 | GC02M039417 | 1.621135 | https://www.genecards.org/cgi-bin/carddisp.pl?gene=SOS1 |
| TF | Transferrin | Protein Coding | P02787 | 64 | GC03P142020 | 1.621135 | https://www.genecards.org/cgi-bin/carddisp.pl?gene=TF |
| TGFB2 | Transforming Growth Factor Beta 2 | Protein Coding | P61812 | 64 | GC01P218345 | 1.621135 | https://www.genecards.org/cgi-bin/carddisp.pl?gene=TGFB2 |
| ZAP70 | Zeta Chain Of T Cell Receptor Associated Protein Kinase 70 | Protein Coding | P43403 | 64 | GC02P099091 | 1.621135 | https://www.genecards.org/cgi-bin/carddisp.pl?gene=ZAP70 |
| ALPL | Alkaline Phosphatase, Biomineralization Associated | Protein Coding | P05186 | 63 | GC01P021508 | 1.621135 | https://www.genecards.org/cgi-bin/carddisp.pl?gene=ALPL |
| APOA1 | Apolipoprotein A1 | Protein Coding | P02647 | 63 | GC11M116835 | 1.621135 | https://www.genecards.org/cgi-bin/carddisp.pl?gene=APOA1 |
| ARG1 | Arginase 1 | Protein Coding | P05089 | 63 | GC06P174283 | 1.621135 | https://www.genecards.org/cgi-bin/carddisp.pl?gene=ARG1 |
| BCHE | Butyrylcholinesterase | Protein Coding | P06276 | 63 | GC03M165772 | 1.621135 | https://www.genecards.org/cgi-bin/carddisp.pl?gene=BCHE |
| BLM | BLM RecQ Like Helicase | Protein Coding | P54132 | 63 | GC15P090717 | 1.621135 | https://www.genecards.org/cgi-bin/carddisp.pl?gene=BLM |
| CD40LG | CD40 Ligand | Protein Coding | P29965 | 63 | GC0XP136649 | 1.621135 | https://www.genecards.org/cgi-bin/carddisp.pl?gene=CD40LG |
| CTSK | Cathepsin K | Protein Coding | P43235 | 63 | GC01M166075 | 1.621135 | https://www.genecards.org/cgi-bin/carddisp.pl?gene=CTSK |
| DLD | Dihydrolipoamide Dehydrogenase | Protein Coding | P09622 | 63 | GC07P109995 | 1.621135 | https://www.genecards.org/cgi-bin/carddisp.pl?gene=DLD |
| DSP | Desmoplakin | Protein Coding | P15924 | 63 | GC06P007541 | 1.621135 | https://www.genecards.org/cgi-bin/carddisp.pl?gene=DSP |
| GBA1 | Glucosylceramidase Beta 1 | Protein Coding | P04062 | 63 | GC01M166343 | 1.621135 | https://www.genecards.org/cgi-bin/carddisp.pl?gene=GBA1 |
| GLA | Galactosidase Alpha | Protein Coding | P06280 | 63 | GC0XM101393 | 1.621135 | https://www.genecards.org/cgi-bin/carddisp.pl?gene=GLA |
| IL2RA | Interleukin 2 Receptor Subunit Alpha | Protein Coding | P01589 | 63 | GC10M006010 | 1.621135 | https://www.genecards.org/cgi-bin/carddisp.pl?gene=IL2RA |
| JAG1 | Jagged Canonical Notch Ligand 1 | Protein Coding | P78504 | 63 | GC20M010637 | 1.621135 | https://www.genecards.org/cgi-bin/carddisp.pl?gene=JAG1 |
| KCNH2 | Potassium Voltage-Gated Channel Subfamily H Member 2 | Protein Coding | Q12809 | 63 | GC07M150944 | 1.621135 | https://www.genecards.org/cgi-bin/carddisp.pl?gene=KCNH2 |
| KCNQ1 | Potassium Voltage-Gated Channel Subfamily Q Member 1 | Protein Coding | P51787 | 63 | GC11P002444 | 1.621135 | https://www.genecards.org/cgi-bin/carddisp.pl?gene=KCNQ1 |
| LMNA | Lamin A/C | Protein Coding | P02545 | 63 | GC01P156082 | 1.621135 | https://www.genecards.org/cgi-bin/carddisp.pl?gene=LMNA |
| NTRK1 | Neurotrophic Receptor Tyrosine Kinase 1 | Protein Coding | P04629 | 63 | GC01P156815 | 1.621135 | https://www.genecards.org/cgi-bin/carddisp.pl?gene=NTRK1 |
| PCSK9 | Proprotein Convertase Subtilisin/Kexin Type 9 | Protein Coding | Q8NBP7 | 63 | GC01P055039 | 1.621135 | https://www.genecards.org/cgi-bin/carddisp.pl?gene=PCSK9 |
| PHGDH | Phosphoglycerate Dehydrogenase | Protein Coding | O43175 | 63 | GC01P119692 | 1.621135 | https://www.genecards.org/cgi-bin/carddisp.pl?gene=PHGDH |
| PRKAG2 | Protein Kinase AMP-Activated Non-Catalytic Subunit Gamma 2 | Protein Coding | Q9UGJ0 | 63 | GC07M151556 | 1.621135 | https://www.genecards.org/cgi-bin/carddisp.pl?gene=PRKAG2 |
| PRKDC | Protein Kinase, DNA-Activated, Catalytic Subunit | Protein Coding | P78527 | 63 | GC08M047773 | 1.621135 | https://www.genecards.org/cgi-bin/carddisp.pl?gene=PRKDC |
| PRKG1 | Protein Kinase CGMP-Dependent 1 | Protein Coding | Q13976 | 63 | GC10P052081 | 1.621135 | https://www.genecards.org/cgi-bin/carddisp.pl?gene=PRKG1 |
| SCN5A | Sodium Voltage-Gated Channel Alpha Subunit 5 | Protein Coding | Q14524 | 63 | GC03M038812 | 1.621135 | https://www.genecards.org/cgi-bin/carddisp.pl?gene=SCN5A |
| TH | Tyrosine Hydroxylase | Protein Coding | P07101 | 63 | GC11M002163 | 1.621135 | https://www.genecards.org/cgi-bin/carddisp.pl?gene=TH |
| ABCA3 | ATP Binding Cassette Subfamily A Member 3 | Protein Coding | Q99758 | 62 | GC16M002275 | 1.621135 | https://www.genecards.org/cgi-bin/carddisp.pl?gene=ABCA3 |
| BRIP1 | BRCA1 Interacting DNA Helicase 1 | Protein Coding | Q9BX63 | 62 | GC17M061679 | 1.621135 | https://www.genecards.org/cgi-bin/carddisp.pl?gene=BRIP1 |
| CACNA1C | Calcium Voltage-Gated Channel Subunit Alpha1 C | Protein Coding | Q13936 | 62 | GC12P001970 | 1.621135 | https://www.genecards.org/cgi-bin/carddisp.pl?gene=CACNA1C |
| CAD | Carbamoyl-Phosphate Synthetase 2, Aspartate Transcarbamylase, And Dihydroorotase | Protein Coding | P27708 | 62 | GC02P027217 | 1.621135 | https://www.genecards.org/cgi-bin/carddisp.pl?gene=CAD |
| CD247 | CD247 Molecule | Protein Coding | P20963 | 62 | GC01M167399 | 1.621135 | https://www.genecards.org/cgi-bin/carddisp.pl?gene=CD247 |
| CP | Ceruloplasmin | Protein Coding | P00450 | 62 | GC03M149162 | 1.621135 | https://www.genecards.org/cgi-bin/carddisp.pl?gene=CP |
| CTSF | Cathepsin F | Protein Coding | Q9UBX1 | 62 | GC11M136630 | 1.621135 | https://www.genecards.org/cgi-bin/carddisp.pl?gene=CTSF |
| FANCA | FA Complementation Group A | Protein Coding | O15360 | 62 | GC16M090272 | 1.621135 | https://www.genecards.org/cgi-bin/carddisp.pl?gene=FANCA |
| FLNA | Filamin A | Protein Coding | P21333 | 62 | GC0XM154348 | 1.621135 | https://www.genecards.org/cgi-bin/carddisp.pl?gene=FLNA |
| GATA4 | GATA Binding Protein 4 | Protein Coding | P43694 | 62 | GC08P011676 | 1.621135 | https://www.genecards.org/cgi-bin/carddisp.pl?gene=GATA4 |
| GLB1 | Galactosidase Beta 1 | Protein Coding | P16278 | 62 | GC03M032963 | 1.621135 | https://www.genecards.org/cgi-bin/carddisp.pl?gene=GLB1 |
| GUSB | Glucuronidase Beta | Protein Coding | P08236 | 62 | GC07M065960 | 1.621135 | https://www.genecards.org/cgi-bin/carddisp.pl?gene=GUSB |
| LIPA | Lipase A, Lysosomal Acid Type | Protein Coding | P38571 | 62 | GC10M089213 | 1.621135 | https://www.genecards.org/cgi-bin/carddisp.pl?gene=LIPA |
| NPC1 | NPC Intracellular Cholesterol Transporter 1 | Protein Coding | O15118 | 62 | GC18M023506 | 1.621135 | https://www.genecards.org/cgi-bin/carddisp.pl?gene=NPC1 |
| PNP | Purine Nucleoside Phosphorylase | Protein Coding | P00491 | 62 | GC14P054161 | 1.621135 | https://www.genecards.org/cgi-bin/carddisp.pl?gene=PNP |
| PPIB | Peptidylprolyl Isomerase B | Protein Coding | P23284 | 62 | GC15M064155 | 1.621135 | https://www.genecards.org/cgi-bin/carddisp.pl?gene=PPIB |
| SCN10A | Sodium Voltage-Gated Channel Alpha Subunit 10 | Protein Coding | Q9Y5Y9 | 62 | GC03M038813 | 1.621135 | https://www.genecards.org/cgi-bin/carddisp.pl?gene=SCN10A |
| TNFSF11 | TNF Superfamily Member 11 | Protein Coding | O14788 | 62 | GC13P042562 | 1.621135 | https://www.genecards.org/cgi-bin/carddisp.pl?gene=TNFSF11 |
| ACTA2 | Actin Alpha 2, Smooth Muscle | Protein Coding | P62736 | 61 | GC10M088935 | 1.621135 | https://www.genecards.org/cgi-bin/carddisp.pl?gene=ACTA2 |
| ATP1B1 | ATPase Na+/K+ Transporting Subunit Beta 1 | Protein Coding | P05026 | 61 | GC01P169105 | 1.621135 | https://www.genecards.org/cgi-bin/carddisp.pl?gene=ATP1B1 |
| ATP7B | ATPase Copper Transporting Beta | Protein Coding | P35670 | 61 | GC13M051930 | 1.621135 | https://www.genecards.org/cgi-bin/carddisp.pl?gene=ATP7B |
| CAPN3 | Calpain 3 | Protein Coding | P20807 | 61 | GC15P042359 | 1.621135 | https://www.genecards.org/cgi-bin/carddisp.pl?gene=CAPN3 |
| CD8A | CD8 Subunit Alpha | Protein Coding | P01732 | 61 | GC02M086784 | 1.621135 | https://www.genecards.org/cgi-bin/carddisp.pl?gene=CD8A |
| COL3A1 | Collagen Type III Alpha 1 Chain | Protein Coding | P02461 | 61 | GC02P188974 | 1.621135 | https://www.genecards.org/cgi-bin/carddisp.pl?gene=COL3A1 |
| CPS1 | Carbamoyl-Phosphate Synthase 1 | Protein Coding | P31327 | 61 | GC02P210477 | 1.621135 | https://www.genecards.org/cgi-bin/carddisp.pl?gene=CPS1 |
| CYBB | Cytochrome B-245 Beta Chain | Protein Coding | P04839 | 61 | GC0XP037780 | 1.621135 | https://www.genecards.org/cgi-bin/carddisp.pl?gene=CYBB |
| CYP1B1 | Cytochrome P450 Family 1 Subfamily B Member 1 | Protein Coding | Q16678 | 61 | GC02M038066 | 1.621135 | https://www.genecards.org/cgi-bin/carddisp.pl?gene=CYP1B1 |
| DES | Desmin | Protein Coding | P17661 | 61 | GC02P219418 | 1.621135 | https://www.genecards.org/cgi-bin/carddisp.pl?gene=DES |
| DHCR7 | 7-Dehydrocholesterol Reductase | Protein Coding | Q9UBM7 | 61 | GC11M136756 | 1.621135 | https://www.genecards.org/cgi-bin/carddisp.pl?gene=DHCR7 |
| ERCC2 | ERCC Excision Repair 2, TFIIH Core Complex Helicase Subunit | Protein Coding | P18074 | 61 | GC19M045349 | 1.621135 | https://www.genecards.org/cgi-bin/carddisp.pl?gene=ERCC2 |
| ERCC3 | ERCC Excision Repair 3, TFIIH Core Complex Helicase Subunit | Protein Coding | P19447 | 61 | GC02M127257 | 1.621135 | https://www.genecards.org/cgi-bin/carddisp.pl?gene=ERCC3 |
| FUCA1 | Alpha-L-Fucosidase 1 | Protein Coding | P04066 | 61 | GC01M023845 | 1.621135 | https://www.genecards.org/cgi-bin/carddisp.pl?gene=FUCA1 |
| GALNS | Galactosamine (N-Acetyl)-6-Sulfatase | Protein Coding | P34059 | 61 | GC16M088813 | 1.621135 | https://www.genecards.org/cgi-bin/carddisp.pl?gene=GALNS |
| GPHN | Gephyrin | Protein Coding | Q9NQX3 | 61 | GC14P066507 | 1.621135 | https://www.genecards.org/cgi-bin/carddisp.pl?gene=GPHN |
| HCN4 | Hyperpolarization Activated Cyclic Nucleotide Gated Potassium Channel 4 | Protein Coding | Q9Y3Q4 | 61 | GC15M073319 | 1.621135 | https://www.genecards.org/cgi-bin/carddisp.pl?gene=HCN4 |
| IL2RG | Interleukin 2 Receptor Subunit Gamma | Protein Coding | P31785 | 61 | GC0XM071118 | 1.621135 | https://www.genecards.org/cgi-bin/carddisp.pl?gene=IL2RG |
| JUP | Junction Plakoglobin | Protein Coding | P14923 | 61 | GC17M041754 | 1.621135 | https://www.genecards.org/cgi-bin/carddisp.pl?gene=JUP |
| KCNJ1 | Potassium Inwardly Rectifying Channel Subfamily J Member 1 | Protein Coding | P48048 | 61 | GC11M137616 | 1.621135 | https://www.genecards.org/cgi-bin/carddisp.pl?gene=KCNJ1 |
| KCNJ2 | Potassium Inwardly Rectifying Channel Subfamily J Member 2 | Protein Coding | P63252 | 61 | GC17P070168 | 1.621135 | https://www.genecards.org/cgi-bin/carddisp.pl?gene=KCNJ2 |
| KCNJ5 | Potassium Inwardly Rectifying Channel Subfamily J Member 5 | Protein Coding | P48544 | 61 | GC11P128891 | 1.621135 | https://www.genecards.org/cgi-bin/carddisp.pl?gene=KCNJ5 |
| LRP2 | LDL Receptor Related Protein 2 | Protein Coding | P98164 | 61 | GC02M169127 | 1.621135 | https://www.genecards.org/cgi-bin/carddisp.pl?gene=LRP2 |
| MYL2 | Myosin Light Chain 2 | Protein Coding | P10916 | 61 | GC12M111940 | 1.621135 | https://www.genecards.org/cgi-bin/carddisp.pl?gene=MYL2 |
| NBN | Nibrin | Protein Coding | O60934 | 61 | GC08M089933 | 1.621135 | https://www.genecards.org/cgi-bin/carddisp.pl?gene=NBN |
| NCF2 | Neutrophil Cytosolic Factor 2 | Protein Coding | P19878 | 61 | GC01M186633 | 1.621135 | https://www.genecards.org/cgi-bin/carddisp.pl?gene=NCF2 |
| NF1 | Neurofibromin 1 | Protein Coding | P21359 | 61 | GC17P031094 | 1.621135 | https://www.genecards.org/cgi-bin/carddisp.pl?gene=NF1 |
| NR2F2 | Nuclear Receptor Subfamily 2 Group F Member 2 | Protein Coding | P24468 | 61 | GC15P096325 | 1.621135 | https://www.genecards.org/cgi-bin/carddisp.pl?gene=NR2F2 |
| PSAP | Prosaposin | Protein Coding | P07602 | 61 | GC10M071816 | 1.621135 | https://www.genecards.org/cgi-bin/carddisp.pl?gene=PSAP |
| SLC12A1 | Solute Carrier Family 12 Member 1 | Protein Coding | Q13621 | 61 | GC15P184282 | 1.621135 | https://www.genecards.org/cgi-bin/carddisp.pl?gene=SLC12A1 |
| SLC25A4 | Solute Carrier Family 25 Member 4 | Protein Coding | P12235 | 61 | GC04P185143 | 1.621135 | https://www.genecards.org/cgi-bin/carddisp.pl?gene=SLC25A4 |
| SMPD1 | Sphingomyelin Phosphodiesterase 1 | Protein Coding | P17405 | 61 | GC11P006390 | 1.621135 | https://www.genecards.org/cgi-bin/carddisp.pl?gene=SMPD1 |
| STK4 | Serine/Threonine Kinase 4 | Protein Coding | Q13043 | 61 | GC20P044966 | 1.621135 | https://www.genecards.org/cgi-bin/carddisp.pl?gene=STK4 |
| TGFB3 | Transforming Growth Factor Beta 3 | Protein Coding | P10600 | 61 | GC14M075958 | 1.621135 | https://www.genecards.org/cgi-bin/carddisp.pl?gene=TGFB3 |
| TNNI3 | Troponin I3, Cardiac Type | Protein Coding | P19429 | 61 | GC19M055151 | 1.621135 | https://www.genecards.org/cgi-bin/carddisp.pl?gene=TNNI3 |
| TNNT2 | Troponin T2, Cardiac Type | Protein Coding | P45379 | 61 | GC01M201359 | 1.621135 | https://www.genecards.org/cgi-bin/carddisp.pl?gene=TNNT2 |
| TPM1 | Tropomyosin 1 | Protein Coding | P09493 | 61 | GC15P192130 | 1.621135 | https://www.genecards.org/cgi-bin/carddisp.pl?gene=TPM1 |
| TTN | Titin | Protein Coding | Q8WZ42 | 61 | GC02M178525 | 1.621135 | https://www.genecards.org/cgi-bin/carddisp.pl?gene=TTN |
| TTR | Transthyretin | Protein Coding | P02766 | 61 | GC18P031557 | 1.621135 | https://www.genecards.org/cgi-bin/carddisp.pl?gene=TTR |
| VLDLR | Very Low Density Lipoprotein Receptor | Protein Coding | P98155 | 61 | GC09P002611 | 1.621135 | https://www.genecards.org/cgi-bin/carddisp.pl?gene=VLDLR |
| WAS | WASP Actin Nucleation Promoting Factor | Protein Coding | P42768 | 61 | GC0XP048676 | 1.621135 | https://www.genecards.org/cgi-bin/carddisp.pl?gene=WAS |
| ABCB11 | ATP Binding Cassette Subfamily B Member 11 | Protein Coding | O95342 | 60 | GC02M168922 | 1.621135 | https://www.genecards.org/cgi-bin/carddisp.pl?gene=ABCB11 |
| ABCB4 | ATP Binding Cassette Subfamily B Member 4 | Protein Coding | P21439 | 60 | GC07M087365 | 1.621135 | https://www.genecards.org/cgi-bin/carddisp.pl?gene=ABCB4 |
| ACTN2 | Actinin Alpha 2 | Protein Coding | P35609 | 60 | GC01P236686 | 1.621135 | https://www.genecards.org/cgi-bin/carddisp.pl?gene=ACTN2 |
| AK2 | Adenylate Kinase 2 | Protein Coding | P54819 | 60 | GC01M033007 | 1.621135 | https://www.genecards.org/cgi-bin/carddisp.pl?gene=AK2 |
| ALDH7A1 | Aldehyde Dehydrogenase 7 Family Member A1 | Protein Coding | P49419 | 60 | GC05M126541 | 1.621135 | https://www.genecards.org/cgi-bin/carddisp.pl?gene=ALDH7A1 |
| ALDOB | Aldolase, Fructose-Bisphosphate B | Protein Coding | P05062 | 60 | GC09M101420 | 1.621135 | https://www.genecards.org/cgi-bin/carddisp.pl?gene=ALDOB |
| ARSA | Arylsulfatase A | Protein Coding | P15289 | 60 | GC22M050622 | 1.621135 | https://www.genecards.org/cgi-bin/carddisp.pl?gene=ARSA |
| ARSB | Arylsulfatase B | Protein Coding | P15848 | 60 | GC05M078777 | 1.621135 | https://www.genecards.org/cgi-bin/carddisp.pl?gene=ARSB |
| AVPR2 | Arginine Vasopressin Receptor 2 | Protein Coding | P30518 | 60 | GC0XP153902 | 1.621135 | https://www.genecards.org/cgi-bin/carddisp.pl?gene=AVPR2 |
| BMP1 | Bone Morphogenetic Protein 1 | Protein Coding | P13497 | 60 | GC08P022165 | 1.621135 | https://www.genecards.org/cgi-bin/carddisp.pl?gene=BMP1 |
| CD3E | CD3 Epsilon Subunit Of T-Cell Receptor Complex | Protein Coding | P07766 | 60 | GC11P118304 | 1.621135 | https://www.genecards.org/cgi-bin/carddisp.pl?gene=CD3E |
| COL5A1 | Collagen Type V Alpha 1 Chain | Protein Coding | P20908 | 60 | GC09P134641 | 1.621135 | https://www.genecards.org/cgi-bin/carddisp.pl?gene=COL5A1 |
| CYBA | Cytochrome B-245 Alpha Chain | Protein Coding | P13498 | 60 | GC16M088643 | 1.621135 | https://www.genecards.org/cgi-bin/carddisp.pl?gene=CYBA |
| CYP27A1 | Cytochrome P450 Family 27 Subfamily A Member 1 | Protein Coding | Q02318 | 60 | GC02P218781 | 1.621135 | https://www.genecards.org/cgi-bin/carddisp.pl?gene=CYP27A1 |
| ERCC6 | ERCC Excision Repair 6, Chromatin Remodeling Factor | Protein Coding | Q03468 | 60 | GC10M049454 | 1.621135 | https://www.genecards.org/cgi-bin/carddisp.pl?gene=ERCC6 |
| F9 | Coagulation Factor IX | Protein Coding | P00740 | 60 | GC0XP139530 | 1.621135 | https://www.genecards.org/cgi-bin/carddisp.pl?gene=F9 |
| FANCC | FA Complementation Group C | Protein Coding | Q00597 | 60 | GC09M095099 | 1.621135 | https://www.genecards.org/cgi-bin/carddisp.pl?gene=FANCC |
| FANCD2 | FA Complementation Group D2 | Protein Coding | Q9BXW9 | 60 | GC03P010026 | 1.621135 | https://www.genecards.org/cgi-bin/carddisp.pl?gene=FANCD2 |
| FBN1 | Fibrillin 1 | Protein Coding | P35555 | 60 | GC15M048408 | 1.621135 | https://www.genecards.org/cgi-bin/carddisp.pl?gene=FBN1 |
| FOXP3 | Forkhead Box P3 | Protein Coding | Q9BZS1 | 60 | GC0XM049250 | 1.621135 | https://www.genecards.org/cgi-bin/carddisp.pl?gene=FOXP3 |
| GALK1 | Galactokinase 1 | Protein Coding | P51570 | 60 | GC17M075751 | 1.621135 | https://www.genecards.org/cgi-bin/carddisp.pl?gene=GALK1 |
| GATA6 | GATA Binding Protein 6 | Protein Coding | Q92908 | 60 | GC18P022169 | 1.621135 | https://www.genecards.org/cgi-bin/carddisp.pl?gene=GATA6 |
| GFPT1 | Glutamine--Fructose-6-Phosphate Transaminase 1 | Protein Coding | Q06210 | 60 | GC02M069319 | 1.621135 | https://www.genecards.org/cgi-bin/carddisp.pl?gene=GFPT1 |
| HEXA | Hexosaminidase Subunit Alpha | Protein Coding | P06865 | 60 | GC15M072340 | 1.621135 | https://www.genecards.org/cgi-bin/carddisp.pl?gene=HEXA |
| HEXB | Hexosaminidase Subunit Beta | Protein Coding | P07686 | 60 | GC05P074640 | 1.621135 | https://www.genecards.org/cgi-bin/carddisp.pl?gene=HEXB |
| HYAL1 | Hyaluronidase 1 | Protein Coding | Q12794 | 60 | GC03M050299 | 1.621135 | https://www.genecards.org/cgi-bin/carddisp.pl?gene=HYAL1 |
| IDS | Iduronate 2-Sulfatase | Protein Coding | P22304 | 60 | GC0XM149476 | 1.621135 | https://www.genecards.org/cgi-bin/carddisp.pl?gene=IDS |
| ITPA | Inosine Triphosphatase | Protein Coding | Q9BY32 | 60 | GC20P010242 | 1.621135 | https://www.genecards.org/cgi-bin/carddisp.pl?gene=ITPA |
| KCND3 | Potassium Voltage-Gated Channel Subfamily D Member 3 | Protein Coding | Q9UK17 | 60 | GC01M111770 | 1.621135 | https://www.genecards.org/cgi-bin/carddisp.pl?gene=KCND3 |
| L1CAM | L1 Cell Adhesion Molecule | Protein Coding | P32004 | 60 | GC0XM153864 | 1.621135 | https://www.genecards.org/cgi-bin/carddisp.pl?gene=L1CAM |
| LAMC2 | Laminin Subunit Gamma 2 | Protein Coding | Q13753 | 60 | GC01P183186 | 1.621135 | https://www.genecards.org/cgi-bin/carddisp.pl?gene=LAMC2 |
| LIFR | LIF Receptor Subunit Alpha | Protein Coding | P42702 | 60 | GC05M038741 | 1.621135 | https://www.genecards.org/cgi-bin/carddisp.pl?gene=LIFR |
| LIG4 | DNA Ligase 4 | Protein Coding | P49917 | 60 | GC13M108207 | 1.621135 | https://www.genecards.org/cgi-bin/carddisp.pl?gene=LIG4 |
| LOX | Lysyl Oxidase | Protein Coding | P28300 | 60 | GC05M122063 | 1.621135 | https://www.genecards.org/cgi-bin/carddisp.pl?gene=LOX |
| MALT1 | MALT1 Paracaspase | Protein Coding | Q9UDY8 | 60 | GC18P058671 | 1.621135 | https://www.genecards.org/cgi-bin/carddisp.pl?gene=MALT1 |
| MPL | MPL Proto-Oncogene, Thrombopoietin Receptor | Protein Coding | P40238 | 60 | GC01P043337 | 1.621135 | https://www.genecards.org/cgi-bin/carddisp.pl?gene=MPL |
| MVK | Mevalonate Kinase | Protein Coding | Q03426 | 60 | GC12P109573 | 1.621135 | https://www.genecards.org/cgi-bin/carddisp.pl?gene=MVK |
| MYH7 | Myosin Heavy Chain 7 | Protein Coding | P12883 | 60 | GC14M023412 | 1.621135 | https://www.genecards.org/cgi-bin/carddisp.pl?gene=MYH7 |
| NCF4 | Neutrophil Cytosolic Factor 4 | Protein Coding | Q15080 | 60 | GC22P036860 | 1.621135 | https://www.genecards.org/cgi-bin/carddisp.pl?gene=NCF4 |
| NPHS1 | NPHS1 Adhesion Molecule, Nephrin | Protein Coding | O60500 | 60 | GC19M035825 | 1.621135 | https://www.genecards.org/cgi-bin/carddisp.pl?gene=NPHS1 |
| PDHA1 | Pyruvate Dehydrogenase E1 Subunit Alpha 1 | Protein Coding | P08559 | 60 | GC0XP019343 | 1.621135 | https://www.genecards.org/cgi-bin/carddisp.pl?gene=PDHA1 |
| PDHB | Pyruvate Dehydrogenase E1 Subunit Beta | Protein Coding | P11177 | 60 | GC03M058475 | 1.621135 | https://www.genecards.org/cgi-bin/carddisp.pl?gene=PDHB |
| PPT1 | Palmitoyl-Protein Thioesterase 1 | Protein Coding | P50897 | 60 | GC01M040249 | 1.621135 | https://www.genecards.org/cgi-bin/carddisp.pl?gene=PPT1 |
| PRF1 | Perforin 1 | Protein Coding | P14222 | 60 | GC10M070597 | 1.621135 | https://www.genecards.org/cgi-bin/carddisp.pl?gene=PRF1 |
| RYR2 | Ryanodine Receptor 2 | Protein Coding | Q92736 | 60 | GC01P237042 | 1.621135 | https://www.genecards.org/cgi-bin/carddisp.pl?gene=RYR2 |
| SGSH | N-Sulfoglucosamine Sulfohydrolase | Protein Coding | P51688 | 60 | GC17M094409 | 1.621135 | https://www.genecards.org/cgi-bin/carddisp.pl?gene=SGSH |
| SLC6A8 | Solute Carrier Family 6 Member 8 | Protein Coding | P48029 | 60 | GC0XP153774 | 1.621135 | https://www.genecards.org/cgi-bin/carddisp.pl?gene=SLC6A8 |
| SMAD6 | SMAD Family Member 6 | Protein Coding | O43541 | 60 | GC15P066702 | 1.621135 | https://www.genecards.org/cgi-bin/carddisp.pl?gene=SMAD6 |
| VCL | Vinculin | Protein Coding | P18206 | 60 | GC10P073995 | 1.621135 | https://www.genecards.org/cgi-bin/carddisp.pl?gene=VCL |
| VRK1 | VRK Serine/Threonine Kinase 1 | Protein Coding | Q99986 | 60 | GC14P096797 | 1.621135 | https://www.genecards.org/cgi-bin/carddisp.pl?gene=VRK1 |
| WNT1 | Wnt Family Member 1 | Protein Coding | P04628 | 60 | GC12P069479 | 1.621135 | https://www.genecards.org/cgi-bin/carddisp.pl?gene=WNT1 |
| WRN | WRN RecQ Like Helicase | Protein Coding | Q14191 | 60 | GC08P031033 | 1.621135 | https://www.genecards.org/cgi-bin/carddisp.pl?gene=WRN |
| ABCD1 | ATP Binding Cassette Subfamily D Member 1 | Protein Coding | P33897 | 59 | GC0XP153724 | 1.621135 | https://www.genecards.org/cgi-bin/carddisp.pl?gene=ABCD1 |
| ACOX1 | Acyl-CoA Oxidase 1 | Protein Coding | Q15067 | 59 | GC17M075941 | 1.621135 | https://www.genecards.org/cgi-bin/carddisp.pl?gene=ACOX1 |
| AGA | Aspartylglucosaminidase | Protein Coding | P20933 | 59 | GC04M177430 | 1.621135 | https://www.genecards.org/cgi-bin/carddisp.pl?gene=AGA |
| AGXT | Alanine--Glyoxylate Aminotransferase | Protein Coding | P21549 | 59 | GC02P240868 | 1.621135 | https://www.genecards.org/cgi-bin/carddisp.pl?gene=AGXT |
| ALDH3A2 | Aldehyde Dehydrogenase 3 Family Member A2 | Protein Coding | P51648 | 59 | GC17P019648 | 1.621135 | https://www.genecards.org/cgi-bin/carddisp.pl?gene=ALDH3A2 |
| AMPD2 | Adenosine Monophosphate Deaminase 2 | Protein Coding | Q01433 | 59 | GC01P109616 | 1.621135 | https://www.genecards.org/cgi-bin/carddisp.pl?gene=AMPD2 |
| ASNS | Asparagine Synthetase (Glutamine-Hydrolyzing) | Protein Coding | P08243 | 59 | GC07M097854 | 1.621135 | https://www.genecards.org/cgi-bin/carddisp.pl?gene=ASNS |
| ATP6V1E1 | ATPase H+ Transporting V1 Subunit E1 | Protein Coding | P36543 | 59 | GC22M017592 | 1.621135 | https://www.genecards.org/cgi-bin/carddisp.pl?gene=ATP6V1E1 |
| ATP7A | ATPase Copper Transporting Alpha | Protein Coding | Q04656 | 59 | GC0XP078240 | 1.621135 | https://www.genecards.org/cgi-bin/carddisp.pl?gene=ATP7A |
| ATRX | ATRX Chromatin Remodeler | Protein Coding | P46100 | 59 | GC0XM077504 | 1.621135 | https://www.genecards.org/cgi-bin/carddisp.pl?gene=ATRX |
| CACNA2D1 | Calcium Voltage-Gated Channel Auxiliary Subunit Alpha2delta 1 | Protein Coding | P54289 | 59 | GC07M081946 | 1.621135 | https://www.genecards.org/cgi-bin/carddisp.pl?gene=CACNA2D1 |
| CACNB2 | Calcium Voltage-Gated Channel Auxiliary Subunit Beta 2 | Protein Coding | Q08289 | 59 | GC10P018141 | 1.621135 | https://www.genecards.org/cgi-bin/carddisp.pl?gene=CACNB2 |
| CALM1 | Calmodulin 1 | Protein Coding | P0DP23 | 59 | GC14P090396 | 1.621135 | https://www.genecards.org/cgi-bin/carddisp.pl?gene=CALM1 |
| CD3D | CD3 Delta Subunit Of T-Cell Receptor Complex | Protein Coding | P04234 | 59 | GC11M137418 | 1.621135 | https://www.genecards.org/cgi-bin/carddisp.pl?gene=CD3D |
| CD3G | CD3 Gamma Subunit Of T-Cell Receptor Complex | Protein Coding | P09693 | 59 | GC11P118344 | 1.621135 | https://www.genecards.org/cgi-bin/carddisp.pl?gene=CD3G |
| CRYAB | Crystallin Alpha B | Protein Coding | P02511 | 59 | GC11M111908 | 1.621135 | https://www.genecards.org/cgi-bin/carddisp.pl?gene=CRYAB |
| CYP11B1 | Cytochrome P450 Family 11 Subfamily B Member 1 | Protein Coding | P15538 | 59 | GC08M142872 | 1.621135 | https://www.genecards.org/cgi-bin/carddisp.pl?gene=CYP11B1 |
| CYP27B1 | Cytochrome P450 Family 27 Subfamily B Member 1 | Protein Coding | O15528 | 59 | GC12M060216 | 1.621135 | https://www.genecards.org/cgi-bin/carddisp.pl?gene=CYP27B1 |
| CYP7B1 | Cytochrome P450 Family 7 Subfamily B Member 1 | Protein Coding | O75881 | 59 | GC08M064587 | 1.621135 | https://www.genecards.org/cgi-bin/carddisp.pl?gene=CYP7B1 |
| DDB2 | Damage Specific DNA Binding Protein 2 | Protein Coding | Q92466 | 59 | GC11P048687 | 1.621135 | https://www.genecards.org/cgi-bin/carddisp.pl?gene=DDB2 |
| DOCK8 | Dedicator Of Cytokinesis 8 | Protein Coding | Q8NF50 | 59 | GC09P000322 | 1.621135 | https://www.genecards.org/cgi-bin/carddisp.pl?gene=DOCK8 |
| ERCC4 | ERCC Excision Repair 4, Endonuclease Catalytic Subunit | Protein Coding | Q92889 | 59 | GC16P013920 | 1.621135 | https://www.genecards.org/cgi-bin/carddisp.pl?gene=ERCC4 |
| FANCL | FA Complementation Group L | Protein Coding | Q9NW38 | 59 | GC02M058127 | 1.621135 | https://www.genecards.org/cgi-bin/carddisp.pl?gene=FANCL |
| FHL1 | Four And A Half LIM Domains 1 | Protein Coding | Q13642 | 59 | GC0XP136146 | 1.621135 | https://www.genecards.org/cgi-bin/carddisp.pl?gene=FHL1 |
| FMO3 | Flavin Containing Dimethylaniline Monoxygenase 3 | Protein Coding | P31513 | 59 | GC01P171090 | 1.621135 | https://www.genecards.org/cgi-bin/carddisp.pl?gene=FMO3 |
| GALT | Galactose-1-Phosphate Uridylyltransferase | Protein Coding | P07902 | 59 | GC09P061647 | 1.621135 | https://www.genecards.org/cgi-bin/carddisp.pl?gene=GALT |
| GJB2 | Gap Junction Protein Beta 2 | Protein Coding | P29033 | 59 | GC13M020187 | 1.621135 | https://www.genecards.org/cgi-bin/carddisp.pl?gene=GJB2 |
| HCFC1 | Host Cell Factor C1 | Protein Coding | P51610 | 59 | GC0XM153947 | 1.621135 | https://www.genecards.org/cgi-bin/carddisp.pl?gene=HCFC1 |
| HPD | 4-Hydroxyphenylpyruvate Dioxygenase | Protein Coding | P32754 | 59 | GC12M121839 | 1.621135 | https://www.genecards.org/cgi-bin/carddisp.pl?gene=HPD |
| IL7R | Interleukin 7 Receptor | Protein Coding | P16871 | 59 | GC05P035852 | 1.621135 | https://www.genecards.org/cgi-bin/carddisp.pl?gene=IL7R |
| LAMB3 | Laminin Subunit Beta 3 | Protein Coding | Q13751 | 59 | GC01M209614 | 1.621135 | https://www.genecards.org/cgi-bin/carddisp.pl?gene=LAMB3 |
| MTHFD1 | Methylenetetrahydrofolate Dehydrogenase, Cyclohydrolase And Formyltetrahydrofolate Synthetase 1 | Protein Coding | P11586 | 59 | GC14P064388 | 1.621135 | https://www.genecards.org/cgi-bin/carddisp.pl?gene=MTHFD1 |
| MYL3 | Myosin Light Chain 3 | Protein Coding | P08590 | 59 | GC03M046836 | 1.621135 | https://www.genecards.org/cgi-bin/carddisp.pl?gene=MYL3 |
| MYLK2 | Myosin Light Chain Kinase 2 | Protein Coding | Q9H1R3 | 59 | GC20P031819 | 1.621135 | https://www.genecards.org/cgi-bin/carddisp.pl?gene=MYLK2 |
| NDUFS7 | NADH:Ubiquinone Oxidoreductase Core Subunit S7 | Protein Coding | O75251 | 59 | GC19P141840 | 1.621135 | https://www.genecards.org/cgi-bin/carddisp.pl?gene=NDUFS7 |
| NEU1 | Neuraminidase 1 | Protein Coding | Q99519 | 59 | GC06M031857 | 1.621135 | https://www.genecards.org/cgi-bin/carddisp.pl?gene=NEU1 |
| NR0B1 | Nuclear Receptor Subfamily 0 Group B Member 1 | Protein Coding | P51843 | 59 | GC0XM030304 | 1.621135 | https://www.genecards.org/cgi-bin/carddisp.pl?gene=NR0B1 |
| PDHX | Pyruvate Dehydrogenase Complex Component X | Protein Coding | O00330 | 59 | GC11P034894 | 1.621135 | https://www.genecards.org/cgi-bin/carddisp.pl?gene=PDHX |
| PLOD1 | Procollagen-Lysine,2-Oxoglutarate 5-Dioxygenase 1 | Protein Coding | Q02809 | 59 | GC01P011934 | 1.621135 | https://www.genecards.org/cgi-bin/carddisp.pl?gene=PLOD1 |
| PMM2 | Phosphomannomutase 2 | Protein Coding | O15305 | 59 | GC16P008788 | 1.621135 | https://www.genecards.org/cgi-bin/carddisp.pl?gene=PMM2 |
| PNPO | Pyridoxamine 5'-Phosphate Oxidase | Protein Coding | Q9NVS9 | 59 | GC17P047941 | 1.621135 | https://www.genecards.org/cgi-bin/carddisp.pl?gene=PNPO |
| POLH | DNA Polymerase Eta | Protein Coding | Q9Y253 | 59 | GC06P043576 | 1.621135 | https://www.genecards.org/cgi-bin/carddisp.pl?gene=POLH |
| PPP1CB | Protein Phosphatase 1 Catalytic Subunit Beta | Protein Coding | P62140 | 59 | GC02P029413 | 1.621135 | https://www.genecards.org/cgi-bin/carddisp.pl?gene=PPP1CB |
| PTS | 6-Pyruvoyltetrahydropterin Synthase | Protein Coding | Q03393 | 59 | GC11P112226 | 1.621135 | https://www.genecards.org/cgi-bin/carddisp.pl?gene=PTS |
| RIT1 | Ras Like Without CAAX 1 | Protein Coding | Q92963 | 59 | GC01M155897 | 1.621135 | https://www.genecards.org/cgi-bin/carddisp.pl?gene=RIT1 |
| SCN2B | Sodium Voltage-Gated Channel Beta Subunit 2 | Protein Coding | O60939 | 59 | GC11M137407 | 1.621135 | https://www.genecards.org/cgi-bin/carddisp.pl?gene=SCN2B |
| SLC12A6 | Solute Carrier Family 12 Member 6 | Protein Coding | Q9UHW9 | 59 | GC15M034229 | 1.621135 | https://www.genecards.org/cgi-bin/carddisp.pl?gene=SLC12A6 |
| TGM1 | Transglutaminase 1 | Protein Coding | P22735 | 59 | GC14M024249 | 1.621135 | https://www.genecards.org/cgi-bin/carddisp.pl?gene=TGM1 |
| TMPO | Thymopoietin | Protein Coding | P42166 | 59 | GC12P098515 | 1.621135 | https://www.genecards.org/cgi-bin/carddisp.pl?gene=TMPO |
| TPP1 | Tripeptidyl Peptidase 1 | Protein Coding | O14773 | 59 | GC11M014225 | 1.621135 | https://www.genecards.org/cgi-bin/carddisp.pl?gene=TPP1 |
| UNG | Uracil DNA Glycosylase | Protein Coding | P13051 | 59 | GC12P109097 | 1.621135 | https://www.genecards.org/cgi-bin/carddisp.pl?gene=UNG |
| XPC | XPC Complex Subunit, DNA Damage Recognition And Repair Factor | Protein Coding | Q01831 | 59 | GC03M028072 | 1.621135 | https://www.genecards.org/cgi-bin/carddisp.pl?gene=XPC |
| ABCG5 | ATP Binding Cassette Subfamily G Member 5 | Protein Coding | Q9H222 | 58 | GC02M043806 | 1.621135 | https://www.genecards.org/cgi-bin/carddisp.pl?gene=ABCG5 |
| ACTC1 | Actin Alpha Cardiac Muscle 1 | Protein Coding | P68032 | 58 | GC15M034790 | 1.621135 | https://www.genecards.org/cgi-bin/carddisp.pl?gene=ACTC1 |
| ALG1 | ALG1 Chitobiosyldiphosphodolichol Beta-Mannosyltransferase | Protein Coding | Q9BT22 | 58 | GC16P005033 | 1.621135 | https://www.genecards.org/cgi-bin/carddisp.pl?gene=ALG1 |
| ANTXR2 | ANTXR Cell Adhesion Molecule 2 | Protein Coding | P58335 | 58 | GC04M079901 | 1.621135 | https://www.genecards.org/cgi-bin/carddisp.pl?gene=ANTXR2 |
| APOC3 | Apolipoprotein C3 | Protein Coding | P02656 | 58 | GC11P116829 | 1.621135 | https://www.genecards.org/cgi-bin/carddisp.pl?gene=APOC3 |
| CASQ2 | Calsequestrin 2 | Protein Coding | O14958 | 58 | GC01M115700 | 1.621135 | https://www.genecards.org/cgi-bin/carddisp.pl?gene=CASQ2 |
| CHRNE | Cholinergic Receptor Nicotinic Epsilon Subunit | Protein Coding | Q04844 | 58 | GC17M004897 | 1.621135 | https://www.genecards.org/cgi-bin/carddisp.pl?gene=CHRNE |
| CNTNAP2 | Contactin Associated Protein 2 | Protein Coding | Q9UHC6 | 58 | GC07P146116 | 1.621135 | https://www.genecards.org/cgi-bin/carddisp.pl?gene=CNTNAP2 |
| COL4A3 | Collagen Type IV Alpha 3 Chain | Protein Coding | Q01955 | 58 | GC02P227164 | 1.621135 | https://www.genecards.org/cgi-bin/carddisp.pl?gene=COL4A3 |
| DSC2 | Desmocollin 2 | Protein Coding | Q02487 | 58 | GC18M031058 | 1.621135 | https://www.genecards.org/cgi-bin/carddisp.pl?gene=DSC2 |
| EMD | Emerin | Protein Coding | P50402 | 58 | GC0XP154379 | 1.621135 | https://www.genecards.org/cgi-bin/carddisp.pl?gene=EMD |
| ERCC5 | ERCC Excision Repair 5, Endonuclease | Protein Coding | P28715 | 58 | GC13P102845 | 1.621135 | https://www.genecards.org/cgi-bin/carddisp.pl?gene=ERCC5 |
| FA2H | Fatty Acid 2-Hydroxylase | Protein Coding | Q7L5A8 | 58 | GC16M074712 | 1.621135 | https://www.genecards.org/cgi-bin/carddisp.pl?gene=FA2H |
| GALC | Galactosylceramidase | Protein Coding | P54803 | 58 | GC14M087837 | 1.621135 | https://www.genecards.org/cgi-bin/carddisp.pl?gene=GALC |
| GJA5 | Gap Junction Protein Alpha 5 | Protein Coding | P36382 | 58 | GC01M147756 | 1.621135 | https://www.genecards.org/cgi-bin/carddisp.pl?gene=GJA5 |
| GNPAT | Glyceronephosphate O-Acyltransferase | Protein Coding | O15228 | 58 | GC01P231241 | 1.621135 | https://www.genecards.org/cgi-bin/carddisp.pl?gene=GNPAT |
| GRHPR | Glyoxylate And Hydroxypyruvate Reductase | Protein Coding | Q9UBQ7 | 58 | GC09P061841 | 1.621135 | https://www.genecards.org/cgi-bin/carddisp.pl?gene=GRHPR |
| HELLS | Helicase, Lymphoid Specific | Protein Coding | Q9NRZ9 | 58 | GC10P120190 | 1.621135 | https://www.genecards.org/cgi-bin/carddisp.pl?gene=HELLS |
| KCNA5 | Potassium Voltage-Gated Channel Subfamily A Member 5 | Protein Coding | P22460 | 58 | GC12P005043 | 1.621135 | https://www.genecards.org/cgi-bin/carddisp.pl?gene=KCNA5 |
| KCNE1 | Potassium Voltage-Gated Channel Subfamily E Regulatory Subunit 1 | Protein Coding | P15382 | 58 | GC21M034446 | 1.621135 | https://www.genecards.org/cgi-bin/carddisp.pl?gene=KCNE1 |
| LIG3 | DNA Ligase 3 | Protein Coding | P49916 | 58 | GC17P034980 | 1.621135 | https://www.genecards.org/cgi-bin/carddisp.pl?gene=LIG3 |
| NONO | Non-POU Domain Containing Octamer Binding | Protein Coding | Q15233 | 58 | GC0XP071274 | 1.621135 | https://www.genecards.org/cgi-bin/carddisp.pl?gene=NONO |
| PHKG2 | Phosphorylase Kinase Catalytic Subunit Gamma 2 | Protein Coding | P15735 | 58 | GC16P113182 | 1.621135 | https://www.genecards.org/cgi-bin/carddisp.pl?gene=PHKG2 |
| PLOD2 | Procollagen-Lysine,2-Oxoglutarate 5-Dioxygenase 2 | Protein Coding | O00469 | 58 | GC03M146035 | 1.621135 | https://www.genecards.org/cgi-bin/carddisp.pl?gene=PLOD2 |
| POMT1 | Protein O-Mannosyltransferase 1 | Protein Coding | Q9Y6A1 | 58 | GC09P131502 | 1.621135 | https://www.genecards.org/cgi-bin/carddisp.pl?gene=POMT1 |
| PRDM16 | PR/SET Domain 16 | Protein Coding | Q9HAZ2 | 58 | GC01P067821 | 1.621135 | https://www.genecards.org/cgi-bin/carddisp.pl?gene=PRDM16 |
| RPE65 | Retinoid Isomerohydrolase RPE65 | Protein Coding | Q16518 | 58 | GC01M068428 | 1.621135 | https://www.genecards.org/cgi-bin/carddisp.pl?gene=RPE65 |
| SCN1B | Sodium Voltage-Gated Channel Beta Subunit 1 | Protein Coding | Q07699 | 58 | GC19P035030 | 1.621135 | https://www.genecards.org/cgi-bin/carddisp.pl?gene=SCN1B |
| SERPINF1 | Serpin Family F Member 1 | Protein Coding | P36955 | 58 | GC17P143092 | 1.621135 | https://www.genecards.org/cgi-bin/carddisp.pl?gene=SERPINF1 |
| SMARCAL1 | SNF2 Related Chromatin Remodeling Annealing Helicase 1 | Protein Coding | Q9NZC9 | 58 | GC02P216412 | 1.621135 | https://www.genecards.org/cgi-bin/carddisp.pl?gene=SMARCAL1 |
| SNTA1 | Syntrophin Alpha 1 | Protein Coding | Q13424 | 58 | GC20M033407 | 1.621135 | https://www.genecards.org/cgi-bin/carddisp.pl?gene=SNTA1 |
| TAT | Tyrosine Aminotransferase | Protein Coding | P17735 | 58 | GC16M071565 | 1.621135 | https://www.genecards.org/cgi-bin/carddisp.pl?gene=TAT |
| XPA | XPA, DNA Damage Recognition And Repair Factor | Protein Coding | P23025 | 58 | GC09M097654 | 1.621135 | https://www.genecards.org/cgi-bin/carddisp.pl?gene=XPA |
| ABCC9 | ATP Binding Cassette Subfamily C Member 9 | Protein Coding | O60706 | 57 | GC12M021797 | 1.621135 | https://www.genecards.org/cgi-bin/carddisp.pl?gene=ABCC9 |
| AGPAT2 | 1-Acylglycerol-3-Phosphate O-Acyltransferase 2 | Protein Coding | O15120 | 57 | GC09M136673 | 1.621135 | https://www.genecards.org/cgi-bin/carddisp.pl?gene=AGPAT2 |
| AICDA | Activation Induced Cytidine Deaminase | Protein Coding | Q9GZX7 | 57 | GC12M008602 | 1.621135 | https://www.genecards.org/cgi-bin/carddisp.pl?gene=AICDA |
| AIRE | Autoimmune Regulator | Protein Coding | O43918 | 57 | GC21P044285 | 1.621135 | https://www.genecards.org/cgi-bin/carddisp.pl?gene=AIRE |
| AP3B1 | Adaptor Related Protein Complex 3 Subunit Beta 1 | Protein Coding | O00203 | 57 | GC05M081096 | 1.621135 | https://www.genecards.org/cgi-bin/carddisp.pl?gene=AP3B1 |
| ASPA | Aspartoacylase | Protein Coding | P45381 | 57 | GC17P003472 | 1.621135 | https://www.genecards.org/cgi-bin/carddisp.pl?gene=ASPA |
| ATP13A2 | ATPase Cation Transporting 13A2 | Protein Coding | Q9NQ11 | 57 | GC01M016985 | 1.621135 | https://www.genecards.org/cgi-bin/carddisp.pl?gene=ATP13A2 |
| BGN | Biglycan | Protein Coding | P21810 | 57 | GC0XP153494 | 1.621135 | https://www.genecards.org/cgi-bin/carddisp.pl?gene=BGN |
| CDH23 | Cadherin Related 23 | Protein Coding | Q9H251 | 57 | GC10P071396 | 1.621135 | https://www.genecards.org/cgi-bin/carddisp.pl?gene=CDH23 |
| CHD7 | Chromodomain Helicase DNA Binding Protein 7 | Protein Coding | Q9P2D1 | 57 | GC08P060678 | 1.621135 | https://www.genecards.org/cgi-bin/carddisp.pl?gene=CHD7 |
| CIITA | Class II Major Histocompatibility Complex Transactivator | Protein Coding | P33076 | 57 | GC16P112480 | 1.621135 | https://www.genecards.org/cgi-bin/carddisp.pl?gene=CIITA |
| CLCN5 | Chloride Voltage-Gated Channel 5 | Protein Coding | P51795 | 57 | GC0XP049922 | 1.621135 | https://www.genecards.org/cgi-bin/carddisp.pl?gene=CLCN5 |
| CNGA1 | Cyclic Nucleotide Gated Channel Subunit Alpha 1 | Protein Coding | P29973 | 57 | GC04M047935 | 1.621135 | https://www.genecards.org/cgi-bin/carddisp.pl?gene=CNGA1 |
| COL4A5 | Collagen Type IV Alpha 5 Chain | Protein Coding | P29400 | 57 | GC0XP108439 | 1.621135 | https://www.genecards.org/cgi-bin/carddisp.pl?gene=COL4A5 |
| COL5A2 | Collagen Type V Alpha 2 Chain | Protein Coding | P05997 | 57 | GC02M189031 | 1.621135 | https://www.genecards.org/cgi-bin/carddisp.pl?gene=COL5A2 |
| COL7A1 | Collagen Type VII Alpha 1 Chain | Protein Coding | Q02388 | 57 | GC03M048564 | 1.621135 | https://www.genecards.org/cgi-bin/carddisp.pl?gene=COL7A1 |
| CTNS | Cystinosin, Lysosomal Cystine Transporter | Protein Coding | O60931 | 57 | GC17P003636 | 1.621135 | https://www.genecards.org/cgi-bin/carddisp.pl?gene=CTNS |
| DSG2 | Desmoglein 2 | Protein Coding | Q14126 | 57 | GC18P031498 | 1.621135 | https://www.genecards.org/cgi-bin/carddisp.pl?gene=DSG2 |
| EFEMP2 | EGF Containing Fibulin Extracellular Matrix Protein 2 | Protein Coding | O95967 | 57 | GC11M136594 | 1.621135 | https://www.genecards.org/cgi-bin/carddisp.pl?gene=EFEMP2 |
| EIF2B2 | Eukaryotic Translation Initiation Factor 2B Subunit Beta | Protein Coding | P49770 | 57 | GC14P075002 | 1.621135 | https://www.genecards.org/cgi-bin/carddisp.pl?gene=EIF2B2 |
| EIF2B4 | Eukaryotic Translation Initiation Factor 2B Subunit Delta | Protein Coding | Q9UI10 | 57 | GC02M027364 | 1.621135 | https://www.genecards.org/cgi-bin/carddisp.pl?gene=EIF2B4 |
| FANCG | FA Complementation Group G | Protein Coding | O15287 | 57 | GC09M035073 | 1.621135 | https://www.genecards.org/cgi-bin/carddisp.pl?gene=FANCG |
| FLNC | Filamin C | Protein Coding | Q14315 | 57 | GC07P128830 | 1.621135 | https://www.genecards.org/cgi-bin/carddisp.pl?gene=FLNC |
| FMR1 | Fragile X Messenger Ribonucleoprotein 1 | Protein Coding | Q06787 | 57 | GC0XP148000 | 1.621135 | https://www.genecards.org/cgi-bin/carddisp.pl?gene=FMR1 |
| G6PC1 | Glucose-6-Phosphatase Catalytic Subunit 1 | Protein Coding | P35575 | 57 | GC17P145863 | 1.621135 | https://www.genecards.org/cgi-bin/carddisp.pl?gene=G6PC1 |
| GBE1 | 1,4-Alpha-Glucan Branching Enzyme 1 | Protein Coding | Q04446 | 57 | GC03M081489 | 1.621135 | https://www.genecards.org/cgi-bin/carddisp.pl?gene=GBE1 |
| GFM1 | G Elongation Factor Mitochondrial 1 | Protein Coding | Q96RP9 | 57 | GC03P158644 | 1.621135 | https://www.genecards.org/cgi-bin/carddisp.pl?gene=GFM1 |
| GNS | Glucosamine (N-Acetyl)-6-Sulfatase | Protein Coding | P15586 | 57 | GC12M064713 | 1.621135 | https://www.genecards.org/cgi-bin/carddisp.pl?gene=GNS |
| KCND2 | Potassium Voltage-Gated Channel Subfamily D Member 2 | Protein Coding | Q9NZV8 | 57 | GC07P120273 | 1.621135 | https://www.genecards.org/cgi-bin/carddisp.pl?gene=KCND2 |
| KCNE3 | Potassium Voltage-Gated Channel Subfamily E Regulatory Subunit 3 | Protein Coding | Q9Y6H6 | 57 | GC11M074454 | 1.621135 | https://www.genecards.org/cgi-bin/carddisp.pl?gene=KCNE3 |
| LAMA3 | Laminin Subunit Alpha 3 | Protein Coding | Q16787 | 57 | GC18P023689 | 1.621135 | https://www.genecards.org/cgi-bin/carddisp.pl?gene=LAMA3 |
| LAMA4 | Laminin Subunit Alpha 4 | Protein Coding | Q16363 | 57 | GC06M112107 | 1.621135 | https://www.genecards.org/cgi-bin/carddisp.pl?gene=LAMA4 |
| LAMP2 | Lysosomal Associated Membrane Protein 2 | Protein Coding | P13473 | 57 | GC0XM120426 | 1.621135 | https://www.genecards.org/cgi-bin/carddisp.pl?gene=LAMP2 |
| LARS1 | Leucyl-TRNA Synthetase 1 | Protein Coding | Q9P2J5 | 57 | GC05M146114 | 1.621135 | https://www.genecards.org/cgi-bin/carddisp.pl?gene=LARS1 |
| MAN2B1 | Mannosidase Alpha Class 2B Member 1 | Protein Coding | O00754 | 57 | GC19M104434 | 1.621135 | https://www.genecards.org/cgi-bin/carddisp.pl?gene=MAN2B1 |
| MED12 | Mediator Complex Subunit 12 | Protein Coding | Q93074 | 57 | GC0XP071118 | 1.621135 | https://www.genecards.org/cgi-bin/carddisp.pl?gene=MED12 |
| MEFV | MEFV Innate Immunity Regulator, Pyrin | Protein Coding | O15553 | 57 | GC16M046510 | 1.621135 | https://www.genecards.org/cgi-bin/carddisp.pl?gene=MEFV |
| MRAS | Muscle RAS Oncogene Homolog | Protein Coding | O14807 | 57 | GC03P138347 | 1.621135 | https://www.genecards.org/cgi-bin/carddisp.pl?gene=MRAS |
| NKX2-5 | NK2 Homeobox 5 | Protein Coding | P52952 | 57 | GC05M173232 | 1.621135 | https://www.genecards.org/cgi-bin/carddisp.pl?gene=NKX2-5 |
| NPPA | Natriuretic Peptide A | Protein Coding | P01160 | 57 | GC01M020958 | 1.621135 | https://www.genecards.org/cgi-bin/carddisp.pl?gene=NPPA |
| OCA2 | OCA2 Melanosomal Transmembrane Protein | Protein Coding | Q04671 | 57 | GC15M045184 | 1.621135 | https://www.genecards.org/cgi-bin/carddisp.pl?gene=OCA2 |
| OCRL | OCRL Inositol Polyphosphate-5-Phosphatase | Protein Coding | Q01968 | 57 | GC0XP129539 | 1.621135 | https://www.genecards.org/cgi-bin/carddisp.pl?gene=OCRL |
| PEX2 | Peroxisomal Biogenesis Factor 2 | Protein Coding | P28328 | 57 | GC08M076980 | 1.621135 | https://www.genecards.org/cgi-bin/carddisp.pl?gene=PEX2 |
| PEX5 | Peroxisomal Biogenesis Factor 5 | Protein Coding | P50542 | 57 | GC12P068301 | 1.621135 | https://www.genecards.org/cgi-bin/carddisp.pl?gene=PEX5 |
| PLN | Phospholamban | Protein Coding | P26678 | 57 | GC06P118548 | 1.621135 | https://www.genecards.org/cgi-bin/carddisp.pl?gene=PLN |
| POMGNT1 | Protein O-Linked Mannose N-Acetylglucosaminyltransferase 1 (Beta 1,2-) | Protein Coding | Q8WZA1 | 57 | GC01M046188 | 1.621135 | https://www.genecards.org/cgi-bin/carddisp.pl?gene=POMGNT1 |
| PYCR1 | Pyrroline-5-Carboxylate Reductase 1 | Protein Coding | P32322 | 57 | GC17M081932 | 1.621135 | https://www.genecards.org/cgi-bin/carddisp.pl?gene=PYCR1 |
| RDH12 | Retinol Dehydrogenase 12 | Protein Coding | Q96NR8 | 57 | GC14P067701 | 1.621135 | https://www.genecards.org/cgi-bin/carddisp.pl?gene=RDH12 |
| RLBP1 | Retinaldehyde Binding Protein 1 | Protein Coding | P12271 | 57 | GC15M089209 | 1.621135 | https://www.genecards.org/cgi-bin/carddisp.pl?gene=RLBP1 |
| RNASEH2A | Ribonuclease H2 Subunit A | Protein Coding | O75792 | 57 | GC19P142366 | 1.621135 | https://www.genecards.org/cgi-bin/carddisp.pl?gene=RNASEH2A |
| SCN3B | Sodium Voltage-Gated Channel Beta Subunit 3 | Protein Coding | Q9NY72 | 57 | GC11M123629 | 1.621135 | https://www.genecards.org/cgi-bin/carddisp.pl?gene=SCN3B |
| SCN4B | Sodium Voltage-Gated Channel Beta Subunit 4 | Protein Coding | Q8IWT1 | 57 | GC11M137404 | 1.621135 | https://www.genecards.org/cgi-bin/carddisp.pl?gene=SCN4B |
| SGCD | Sarcoglycan Delta | Protein Coding | Q92629 | 57 | GC05P157436 | 1.621135 | https://www.genecards.org/cgi-bin/carddisp.pl?gene=SGCD |
| SLC7A7 | Solute Carrier Family 7 Member 7 | Protein Coding | Q9UM01 | 57 | GC14M022773 | 1.621135 | https://www.genecards.org/cgi-bin/carddisp.pl?gene=SLC7A7 |
| SOS2 | SOS Ras/Rho Guanine Nucleotide Exchange Factor 2 | Protein Coding | Q07890 | 57 | GC14M050117 | 1.621135 | https://www.genecards.org/cgi-bin/carddisp.pl?gene=SOS2 |
| STXBP2 | Syntaxin Binding Protein 2 | Protein Coding | Q15833 | 57 | GC19P142077 | 1.621135 | https://www.genecards.org/cgi-bin/carddisp.pl?gene=STXBP2 |
| TBX5 | T-Box Transcription Factor 5 | Protein Coding | Q99593 | 57 | GC12M114353 | 1.621135 | https://www.genecards.org/cgi-bin/carddisp.pl?gene=TBX5 |
| TCIRG1 | T Cell Immune Regulator 1, ATPase H+ Transporting V0 Subunit A3 | Protein Coding | Q13488 | 57 | GC11P103525 | 1.621135 | https://www.genecards.org/cgi-bin/carddisp.pl?gene=TCIRG1 |
| UNC13D | Unc-13 Homolog D | Protein Coding | Q70J99 | 57 | GC17M075827 | 1.621135 | https://www.genecards.org/cgi-bin/carddisp.pl?gene=UNC13D |
| AGPS | Alkylglycerone Phosphate Synthase | Protein Coding | O00116 | 56 | GC02P177392 | 1.621135 | https://www.genecards.org/cgi-bin/carddisp.pl?gene=AGPS |
| ALOX12B | Arachidonate 12-Lipoxygenase, 12R Type | Protein Coding | O75342 | 56 | GC17M092460 | 1.621135 | https://www.genecards.org/cgi-bin/carddisp.pl?gene=ALOX12B |
| AMT | Aminomethyltransferase | Protein Coding | P48728 | 56 | GC03M054511 | 1.621135 | https://www.genecards.org/cgi-bin/carddisp.pl?gene=AMT |
| APOA5 | Apolipoprotein A5 | Protein Coding | Q6Q788 | 56 | GC11M116789 | 1.621135 | https://www.genecards.org/cgi-bin/carddisp.pl?gene=APOA5 |
| ARX | Aristaless Related Homeobox | Protein Coding | Q96QS3 | 56 | GC0XM025003 | 1.621135 | https://www.genecards.org/cgi-bin/carddisp.pl?gene=ARX |
| ATP6V0A2 | ATPase H+ Transporting V0 Subunit A2 | Protein Coding | Q9Y487 | 56 | GC12P123712 | 1.621135 | https://www.genecards.org/cgi-bin/carddisp.pl?gene=ATP6V0A2 |
| ATP6V1B1 | ATPase H+ Transporting V1 Subunit B1 | Protein Coding | P15313 | 56 | GC02P070935 | 1.621135 | https://www.genecards.org/cgi-bin/carddisp.pl?gene=ATP6V1B1 |
| BAG3 | BAG Cochaperone 3 | Protein Coding | O95817 | 56 | GC10P119651 | 1.621135 | https://www.genecards.org/cgi-bin/carddisp.pl?gene=BAG3 |
| CALM2 | Calmodulin 2 | Protein Coding | P0DP24 | 56 | GC02M047160 | 1.621135 | https://www.genecards.org/cgi-bin/carddisp.pl?gene=CALM2 |
| CLCN1 | Chloride Voltage-Gated Channel 1 | Protein Coding | P35523 | 56 | GC07P143316 | 1.621135 | https://www.genecards.org/cgi-bin/carddisp.pl?gene=CLCN1 |
| CLN3 | CLN3 Lysosomal/Endosomal Transmembrane Protein, Battenin | Protein Coding | Q13286 | 56 | GC16M028466 | 1.621135 | https://www.genecards.org/cgi-bin/carddisp.pl?gene=CLN3 |
| CORO1A | Coronin 1A | Protein Coding | P31146 | 56 | GC16P113132 | 1.621135 | https://www.genecards.org/cgi-bin/carddisp.pl?gene=CORO1A |
| CRLF1 | Cytokine Receptor Like Factor 1 | Protein Coding | O75462 | 56 | GC19M018572 | 1.621135 | https://www.genecards.org/cgi-bin/carddisp.pl?gene=CRLF1 |
| DCLRE1C | DNA Cross-Link Repair 1C | Protein Coding | Q96SD1 | 56 | GC10M014897 | 1.621135 | https://www.genecards.org/cgi-bin/carddisp.pl?gene=DCLRE1C |
| DTNA | Dystrobrevin Alpha | Protein Coding | Q9Y4J8 | 56 | GC18P034493 | 1.621135 | https://www.genecards.org/cgi-bin/carddisp.pl?gene=DTNA |
| DTNBP1 | Dystrobrevin Binding Protein 1 | Protein Coding | Q96EV8 | 56 | GC06M015620 | 1.621135 | https://www.genecards.org/cgi-bin/carddisp.pl?gene=DTNBP1 |
| EIF2B1 | Eukaryotic Translation Initiation Factor 2B Subunit Alpha | Protein Coding | Q14232 | 56 | GC12M123620 | 1.621135 | https://www.genecards.org/cgi-bin/carddisp.pl?gene=EIF2B1 |
| EIF2B5 | Eukaryotic Translation Initiation Factor 2B Subunit Epsilon | Protein Coding | Q13144 | 56 | GC03P184135 | 1.621135 | https://www.genecards.org/cgi-bin/carddisp.pl?gene=EIF2B5 |
| ELN | Elastin | Protein Coding | P15502 | 56 | GC07P074027 | 1.621135 | https://www.genecards.org/cgi-bin/carddisp.pl?gene=ELN |
| ELP1 | Elongator Acetyltransferase Complex Subunit 1 | Protein Coding | O95163 | 56 | GC09M120172 | 1.621135 | https://www.genecards.org/cgi-bin/carddisp.pl?gene=ELP1 |
| ERCC8 | ERCC Excision Repair 8, CSA Ubiquitin Ligase Complex Subunit | Protein Coding | Q13216 | 56 | GC05M060919 | 1.621135 | https://www.genecards.org/cgi-bin/carddisp.pl?gene=ERCC8 |
| FBN2 | Fibrillin 2 | Protein Coding | P35556 | 56 | GC05M128257 | 1.621135 | https://www.genecards.org/cgi-bin/carddisp.pl?gene=FBN2 |
| GALE | UDP-Galactose-4-Epimerase | Protein Coding | Q14376 | 56 | GC01M023795 | 1.621135 | https://www.genecards.org/cgi-bin/carddisp.pl?gene=GALE |
| GRIP1 | Glutamate Receptor Interacting Protein 1 | Protein Coding | Q9Y3R0 | 56 | GC12M066347 | 1.621135 | https://www.genecards.org/cgi-bin/carddisp.pl?gene=GRIP1 |
| HAMP | Hepcidin Antimicrobial Peptide | Protein Coding | P81172 | 56 | GC19P142820 | 1.621135 | https://www.genecards.org/cgi-bin/carddisp.pl?gene=HAMP |
| HJV | Hemojuvelin BMP Co-Receptor | Protein Coding | Q6ZVN8 | 56 | GC01M165818 | 1.621135 | https://www.genecards.org/cgi-bin/carddisp.pl?gene=HJV |
| KCNJ8 | Potassium Inwardly Rectifying Channel Subfamily J Member 8 | Protein Coding | Q15842 | 56 | GC12M021764 | 1.621135 | https://www.genecards.org/cgi-bin/carddisp.pl?gene=KCNJ8 |
| KIF23 | Kinesin Family Member 23 | Protein Coding | Q02241 | 56 | GC15P069414 | 1.621135 | https://www.genecards.org/cgi-bin/carddisp.pl?gene=KIF23 |
| LRAT | Lecithin Retinol Acyltransferase | Protein Coding | O95237 | 56 | GC04P154626 | 1.621135 | https://www.genecards.org/cgi-bin/carddisp.pl?gene=LRAT |
| MCOLN1 | Mucolipin TRP Cation Channel 1 | Protein Coding | Q9GZU1 | 56 | GC19P142070 | 1.621135 | https://www.genecards.org/cgi-bin/carddisp.pl?gene=MCOLN1 |
| MFAP5 | Microfibril Associated Protein 5 | Protein Coding | Q13361 | 56 | GC12M008637 | 1.621135 | https://www.genecards.org/cgi-bin/carddisp.pl?gene=MFAP5 |
| MID1 | Midline 1 | Protein Coding | O15344 | 56 | GC0XM010445 | 1.621135 | https://www.genecards.org/cgi-bin/carddisp.pl?gene=MID1 |
| MPI | Mannose Phosphate Isomerase | Protein Coding | P34949 | 56 | GC15P074890 | 1.621135 | https://www.genecards.org/cgi-bin/carddisp.pl?gene=MPI |
| MTM1 | Myotubularin 1 | Protein Coding | Q13496 | 56 | GC0XP150562 | 1.621135 | https://www.genecards.org/cgi-bin/carddisp.pl?gene=MTM1 |
| MYO7A | Myosin VIIA | Protein Coding | Q13402 | 56 | GC11P077128 | 1.621135 | https://www.genecards.org/cgi-bin/carddisp.pl?gene=MYO7A |
| NDUFS6 | NADH:Ubiquinone Oxidoreductase Subunit S6 | Protein Coding | O75380 | 56 | GC05P001801 | 1.621135 | https://www.genecards.org/cgi-bin/carddisp.pl?gene=NDUFS6 |
| NGLY1 | N-Glycanase 1 | Protein Coding | Q96IV0 | 56 | GC03M025718 | 1.621135 | https://www.genecards.org/cgi-bin/carddisp.pl?gene=NGLY1 |
| NPC2 | NPC Intracellular Cholesterol Transporter 2 | Protein Coding | P61916 | 56 | GC14M074476 | 1.621135 | https://www.genecards.org/cgi-bin/carddisp.pl?gene=NPC2 |
| PEX14 | Peroxisomal Biogenesis Factor 14 | Protein Coding | O75381 | 56 | GC01P010472 | 1.621135 | https://www.genecards.org/cgi-bin/carddisp.pl?gene=PEX14 |
| PEX19 | Peroxisomal Biogenesis Factor 19 | Protein Coding | P40855 | 56 | GC01M160276 | 1.621135 | https://www.genecards.org/cgi-bin/carddisp.pl?gene=PEX19 |
| PEX7 | Peroxisomal Biogenesis Factor 7 | Protein Coding | O00628 | 56 | GC06P136822 | 1.621135 | https://www.genecards.org/cgi-bin/carddisp.pl?gene=PEX7 |
| PHKA2 | Phosphorylase Kinase Regulatory Subunit Alpha 2 | Protein Coding | P46019 | 56 | GC0XM018892 | 1.621135 | https://www.genecards.org/cgi-bin/carddisp.pl?gene=PHKA2 |
| PHKB | Phosphorylase Kinase Regulatory Subunit Beta | Protein Coding | Q93100 | 56 | GC16P113489 | 1.621135 | https://www.genecards.org/cgi-bin/carddisp.pl?gene=PHKB |
| PKP2 | Plakophilin 2 | Protein Coding | Q99959 | 56 | GC12M032790 | 1.621135 | https://www.genecards.org/cgi-bin/carddisp.pl?gene=PKP2 |
| PLP1 | Proteolipid Protein 1 | Protein Coding | P60201 | 56 | GC0XP103773 | 1.621135 | https://www.genecards.org/cgi-bin/carddisp.pl?gene=PLP1 |
| POMT2 | Protein O-Mannosyltransferase 2 | Protein Coding | Q9UKY4 | 56 | GC14M077274 | 1.621135 | https://www.genecards.org/cgi-bin/carddisp.pl?gene=POMT2 |
| PON2 | Paraoxonase 2 | Protein Coding | Q15165 | 56 | GC07M095404 | 1.621135 | https://www.genecards.org/cgi-bin/carddisp.pl?gene=PON2 |
| PUS1 | Pseudouridine Synthase 1 | Protein Coding | Q9Y606 | 56 | GC12P131929 | 1.621135 | https://www.genecards.org/cgi-bin/carddisp.pl?gene=PUS1 |
| RAG1 | Recombination Activating 1 | Protein Coding | P15918 | 56 | GC11P036554 | 1.621135 | https://www.genecards.org/cgi-bin/carddisp.pl?gene=RAG1 |
| SEPSECS | Sep (O-Phosphoserine) TRNA:Sec (Selenocysteine) TRNA Synthase | Protein Coding | Q9HD40 | 56 | GC04M025121 | 1.621135 | https://www.genecards.org/cgi-bin/carddisp.pl?gene=SEPSECS |
| SLC16A2 | Solute Carrier Family 16 Member 2 | Protein Coding | P36021 | 56 | GC0XP075074 | 1.621135 | https://www.genecards.org/cgi-bin/carddisp.pl?gene=SLC16A2 |
| SLC26A2 | Solute Carrier Family 26 Member 2 | Protein Coding | P50443 | 56 | GC05P157260 | 1.621135 | https://www.genecards.org/cgi-bin/carddisp.pl?gene=SLC26A2 |
| SLC26A4 | Solute Carrier Family 26 Member 4 | Protein Coding | O43511 | 56 | GC07P107660 | 1.621135 | https://www.genecards.org/cgi-bin/carddisp.pl?gene=SLC26A4 |
| SLC27A4 | Solute Carrier Family 27 Member 4 | Protein Coding | Q6P1M0 | 56 | GC09P128340 | 1.621135 | https://www.genecards.org/cgi-bin/carddisp.pl?gene=SLC27A4 |
| TNXB | Tenascin XB | Protein Coding | P22105 | 56 | GC06M103361 | 1.621135 | https://www.genecards.org/cgi-bin/carddisp.pl?gene=TNXB |
| TRIM32 | Tripartite Motif Containing 32 | Protein Coding | Q13049 | 56 | GC09P116687 | 1.621135 | https://www.genecards.org/cgi-bin/carddisp.pl?gene=TRIM32 |
| TRPM4 | Transient Receptor Potential Cation Channel Subfamily M Member 4 | Protein Coding | Q8TD43 | 56 | GC19P049157 | 1.621135 | https://www.genecards.org/cgi-bin/carddisp.pl?gene=TRPM4 |
| ADAMTS2 | ADAM Metallopeptidase With Thrombospondin Type 1 Motif 2 | Protein Coding | O95450 | 55 | GC05M179110 | 1.621135 | https://www.genecards.org/cgi-bin/carddisp.pl?gene=ADAMTS2 |
| ADGRG1 | Adhesion G Protein-Coupled Receptor G1 | Protein Coding | Q9Y653 | 55 | GC16P057610 | 1.621135 | https://www.genecards.org/cgi-bin/carddisp.pl?gene=ADGRG1 |
| ATP6V0A4 | ATPase H+ Transporting V0 Subunit A4 | Protein Coding | Q9HBG4 | 55 | GC07M138783 | 1.621135 | https://www.genecards.org/cgi-bin/carddisp.pl?gene=ATP6V0A4 |
| CALM3 | Calmodulin 3 | Protein Coding | P0DP25 | 55 | GC19P046601 | 1.621135 | https://www.genecards.org/cgi-bin/carddisp.pl?gene=CALM3 |
| CHRNG | Cholinergic Receptor Nicotinic Gamma Subunit | Protein Coding | P07510 | 55 | GC02P232539 | 1.621135 | https://www.genecards.org/cgi-bin/carddisp.pl?gene=CHRNG |
| CNGB1 | Cyclic Nucleotide Gated Channel Subunit Beta 1 | Protein Coding | Q14028 | 55 | GC16M057884 | 1.621135 | https://www.genecards.org/cgi-bin/carddisp.pl?gene=CNGB1 |
| DBT | Dihydrolipoamide Branched Chain Transacylase E2 | Protein Coding | P11182 | 55 | GC01M100186 | 1.621135 | https://www.genecards.org/cgi-bin/carddisp.pl?gene=DBT |
| EXOSC3 | Exosome Component 3 | Protein Coding | Q9NQT5 | 55 | GC09M037772 | 1.621135 | https://www.genecards.org/cgi-bin/carddisp.pl?gene=EXOSC3 |
| GNE | Glucosamine (UDP-N-Acetyl)-2-Epimerase/N-Acetylmannosamine Kinase | Protein Coding | Q9Y223 | 55 | GC09M036214 | 1.621135 | https://www.genecards.org/cgi-bin/carddisp.pl?gene=GNE |
| HAX1 | HCLS1 Associated Protein X-1 | Protein Coding | O00165 | 55 | GC01P173375 | 1.621135 | https://www.genecards.org/cgi-bin/carddisp.pl?gene=HAX1 |
| JPH2 | Junctophilin 2 | Protein Coding | Q9BR39 | 55 | GC20M044111 | 1.621135 | https://www.genecards.org/cgi-bin/carddisp.pl?gene=JPH2 |
| KCNE2 | Potassium Voltage-Gated Channel Subfamily E Regulatory Subunit 2 | Protein Coding | Q9Y6J6 | 55 | GC21P034304 | 1.621135 | https://www.genecards.org/cgi-bin/carddisp.pl?gene=KCNE2 |
| LHX3 | LIM Homeobox 3 | Protein Coding | Q9UBR4 | 55 | GC09M136196 | 1.621135 | https://www.genecards.org/cgi-bin/carddisp.pl?gene=LHX3 |
| LTBP4 | Latent Transforming Growth Factor Beta Binding Protein 4 | Protein Coding | Q8N2S1 | 55 | GC19P040592 | 1.621135 | https://www.genecards.org/cgi-bin/carddisp.pl?gene=LTBP4 |
| LZTR1 | Leucine Zipper Like Post Translational Regulator 1 | Protein Coding | Q8N653 | 55 | GC22P086356 | 1.621135 | https://www.genecards.org/cgi-bin/carddisp.pl?gene=LZTR1 |
| MAK | Male Germ Cell Associated Kinase | Protein Coding | P20794 | 55 | GC06M010762 | 1.621135 | https://www.genecards.org/cgi-bin/carddisp.pl?gene=MAK |
| MANBA | Mannosidase Beta | Protein Coding | O00462 | 55 | GC04M102631 | 1.621135 | https://www.genecards.org/cgi-bin/carddisp.pl?gene=MANBA |
| MOCS2 | Molybdenum Cofactor Synthesis 2 | Protein Coding | O96033 | 55 | GC05M053095 | 1.621135 | https://www.genecards.org/cgi-bin/carddisp.pl?gene=MOCS2 |
| NHEJ1 | Non-Homologous End Joining Factor 1 | Protein Coding | Q9H9Q4 | 55 | GC02M219188 | 1.621135 | https://www.genecards.org/cgi-bin/carddisp.pl?gene=NHEJ1 |
| NPHP1 | Nephrocystin 1 | Protein Coding | O15259 | 55 | GC02M110122 | 1.621135 | https://www.genecards.org/cgi-bin/carddisp.pl?gene=NPHP1 |
| PGM3 | Phosphoglucomutase 3 | Protein Coding | O95394 | 55 | GC06M103994 | 1.621135 | https://www.genecards.org/cgi-bin/carddisp.pl?gene=PGM3 |
| RAB23 | RAB23, Member RAS Oncogene Family | Protein Coding | Q9ULC3 | 55 | GC06M103738 | 1.621135 | https://www.genecards.org/cgi-bin/carddisp.pl?gene=RAB23 |
| RARS2 | Arginyl-TRNA Synthetase 2, Mitochondrial | Protein Coding | Q5T160 | 55 | GC06M104065 | 1.621135 | https://www.genecards.org/cgi-bin/carddisp.pl?gene=RARS2 |
| SAMHD1 | SAM And HD Domain Containing Deoxynucleoside Triphosphate Triphosphohydrolase 1 | Protein Coding | Q9Y3Z3 | 55 | GC20M036890 | 1.621135 | https://www.genecards.org/cgi-bin/carddisp.pl?gene=SAMHD1 |
| SGCG | Sarcoglycan Gamma | Protein Coding | Q13326 | 55 | GC13P023160 | 1.621135 | https://www.genecards.org/cgi-bin/carddisp.pl?gene=SGCG |
| SLC2A10 | Solute Carrier Family 2 Member 10 | Protein Coding | O95528 | 55 | GC20P051509 | 1.621135 | https://www.genecards.org/cgi-bin/carddisp.pl?gene=SLC2A10 |
| TBX1 | T-Box Transcription Factor 1 | Protein Coding | O43435 | 55 | GC22P086289 | 1.621135 | https://www.genecards.org/cgi-bin/carddisp.pl?gene=TBX1 |
| TMEM43 | Transmembrane Protein 43 | Protein Coding | Q9BTV4 | 55 | GC03P025644 | 1.621135 | https://www.genecards.org/cgi-bin/carddisp.pl?gene=TMEM43 |
| TSFM | Ts Translation Elongation Factor, Mitochondrial | Protein Coding | P43897 | 55 | GC12P057782 | 1.621135 | https://www.genecards.org/cgi-bin/carddisp.pl?gene=TSFM |
| TTPA | Alpha Tocopherol Transfer Protein | Protein Coding | P49638 | 55 | GC08M063048 | 1.621135 | https://www.genecards.org/cgi-bin/carddisp.pl?gene=TTPA |
| ABCG8 | ATP Binding Cassette Subfamily G Member 8 | Protein Coding | Q9H221 | 54 | GC02P044543 | 1.621135 | https://www.genecards.org/cgi-bin/carddisp.pl?gene=ABCG8 |
| AKAP9 | A-Kinase Anchoring Protein 9 | Protein Coding | Q99996 | 54 | GC07P091940 | 1.621135 | https://www.genecards.org/cgi-bin/carddisp.pl?gene=AKAP9 |
| ALG6 | ALG6 Alpha-1,3-Glucosyltransferase | Protein Coding | Q9Y672 | 54 | GC01P063367 | 1.621135 | https://www.genecards.org/cgi-bin/carddisp.pl?gene=ALG6 |
| ANK2 | Ankyrin 2 | Protein Coding | Q01484 | 54 | GC04P112784 | 1.621135 | https://www.genecards.org/cgi-bin/carddisp.pl?gene=ANK2 |
| ATP8B1 | ATPase Phospholipid Transporting 8B1 | Protein Coding | O43520 | 54 | GC18M057646 | 1.621135 | https://www.genecards.org/cgi-bin/carddisp.pl?gene=ATP8B1 |
| BBS4 | Bardet-Biedl Syndrome 4 | Protein Coding | Q96RK4 | 54 | GC15P072686 | 1.621135 | https://www.genecards.org/cgi-bin/carddisp.pl?gene=BBS4 |
| CAVIN1 | Caveolae Associated Protein 1 | Protein Coding | Q6NZI2 | 54 | GC17M093442 | 1.621135 | https://www.genecards.org/cgi-bin/carddisp.pl?gene=CAVIN1 |
| CEP290 | Centrosomal Protein 290 | Protein Coding | O15078 | 54 | GC12M088049 | 1.621135 | https://www.genecards.org/cgi-bin/carddisp.pl?gene=CEP290 |
| CLCF1 | Cardiotrophin Like Cytokine Factor 1 | Protein Coding | Q9UBD9 | 54 | GC11M067364 | 1.621135 | https://www.genecards.org/cgi-bin/carddisp.pl?gene=CLCF1 |
| CLP1 | Cleavage Factor Polyribonucleotide Kinase Subunit 1 | Protein Coding | Q92989 | 54 | GC11P057648 | 1.621135 | https://www.genecards.org/cgi-bin/carddisp.pl?gene=CLP1 |
| CRTAP | Cartilage Associated Protein | Protein Coding | O75718 | 54 | GC03P033835 | 1.621135 | https://www.genecards.org/cgi-bin/carddisp.pl?gene=CRTAP |
| CSRP3 | Cysteine And Glycine Rich Protein 3 | Protein Coding | P50461 | 54 | GC11M019160 | 1.621135 | https://www.genecards.org/cgi-bin/carddisp.pl?gene=CSRP3 |
| CTNNA3 | Catenin Alpha 3 | Protein Coding | Q9UI47 | 54 | GC10M065912 | 1.621135 | https://www.genecards.org/cgi-bin/carddisp.pl?gene=CTNNA3 |
| DHDDS | Dehydrodolichyl Diphosphate Synthase Subunit | Protein Coding | Q86SQ9 | 54 | GC01P026432 | 1.621135 | https://www.genecards.org/cgi-bin/carddisp.pl?gene=DHDDS |
| EDA | Ectodysplasin A | Protein Coding | Q92838 | 54 | GC0XP069618 | 1.621135 | https://www.genecards.org/cgi-bin/carddisp.pl?gene=EDA |
| EIF2B3 | Eukaryotic Translation Initiation Factor 2B Subunit Gamma | Protein Coding | Q9NR50 | 54 | GC01M044850 | 1.621135 | https://www.genecards.org/cgi-bin/carddisp.pl?gene=EIF2B3 |
| EYA4 | EYA Transcriptional Coactivator And Phosphatase 4 | Protein Coding | O95677 | 54 | GC06P133240 | 1.621135 | https://www.genecards.org/cgi-bin/carddisp.pl?gene=EYA4 |
| FANCE | FA Complementation Group E | Protein Coding | Q9HB96 | 54 | GC06P173191 | 1.621135 | https://www.genecards.org/cgi-bin/carddisp.pl?gene=FANCE |
| FANCI | FA Complementation Group I | Protein Coding | Q9NVI1 | 54 | GC15P089243 | 1.621135 | https://www.genecards.org/cgi-bin/carddisp.pl?gene=FANCI |
| FKBP10 | FKBP Prolyl Isomerase 10 | Protein Coding | Q96AY3 | 54 | GC17P041812 | 1.621135 | https://www.genecards.org/cgi-bin/carddisp.pl?gene=FKBP10 |
| GNPTAB | N-Acetylglucosamine-1-Phosphate Transferase Subunits Alpha And Beta | Protein Coding | Q3T906 | 54 | GC12M101745 | 1.621135 | https://www.genecards.org/cgi-bin/carddisp.pl?gene=GNPTAB |
| GPD1L | Glycerol-3-Phosphate Dehydrogenase 1 Like | Protein Coding | Q8N335 | 54 | GC03P032156 | 1.621135 | https://www.genecards.org/cgi-bin/carddisp.pl?gene=GPD1L |
| LDB3 | LIM Domain Binding 3 | Protein Coding | O75112 | 54 | GC10P086666 | 1.621135 | https://www.genecards.org/cgi-bin/carddisp.pl?gene=LDB3 |
| LRPPRC | Leucine Rich Pentatricopeptide Repeat Containing | Protein Coding | P42704 | 54 | GC02M043886 | 1.621135 | https://www.genecards.org/cgi-bin/carddisp.pl?gene=LRPPRC |
| MYPN | Myopalladin | Protein Coding | Q86TC9 | 54 | GC10P068106 | 1.621135 | https://www.genecards.org/cgi-bin/carddisp.pl?gene=MYPN |
| NDUFAF2 | NADH:Ubiquinone Oxidoreductase Complex Assembly Factor 2 | Protein Coding | Q8N183 | 54 | GC05P060945 | 1.621135 | https://www.genecards.org/cgi-bin/carddisp.pl?gene=NDUFAF2 |
| NPHS2 | NPHS2 Stomatin Family Member, Podocin | Protein Coding | Q9NP85 | 54 | GC01M179554 | 1.621135 | https://www.genecards.org/cgi-bin/carddisp.pl?gene=NPHS2 |
| PCDH15 | Protocadherin Related 15 | Protein Coding | Q96QU1 | 54 | GC10M053802 | 1.621135 | https://www.genecards.org/cgi-bin/carddisp.pl?gene=PCDH15 |
| PEX3 | Peroxisomal Biogenesis Factor 3 | Protein Coding | P56589 | 54 | GC06P143450 | 1.621135 | https://www.genecards.org/cgi-bin/carddisp.pl?gene=PEX3 |
| PHKA1 | Phosphorylase Kinase Regulatory Subunit Alpha 1 | Protein Coding | P46020 | 54 | GC0XM072578 | 1.621135 | https://www.genecards.org/cgi-bin/carddisp.pl?gene=PHKA1 |
| RAPSN | Receptor Associated Protein Of The Synapse | Protein Coding | Q13702 | 54 | GC11M136239 | 1.621135 | https://www.genecards.org/cgi-bin/carddisp.pl?gene=RAPSN |
| RPGR | Retinitis Pigmentosa GTPase Regulator | Protein Coding | Q92834 | 54 | GC0XM038269 | 1.621135 | https://www.genecards.org/cgi-bin/carddisp.pl?gene=RPGR |
| RPGRIP1L | RPGRIP1 Like | Protein Coding | Q68CZ1 | 54 | GC16M053786 | 1.621135 | https://www.genecards.org/cgi-bin/carddisp.pl?gene=RPGRIP1L |
| RRAS | RAS Related | Protein Coding | P10301 | 54 | GC19M049635 | 1.621135 | https://www.genecards.org/cgi-bin/carddisp.pl?gene=RRAS |
| SGCA | Sarcoglycan Alpha | Protein Coding | Q16586 | 54 | GC17P050164 | 1.621135 | https://www.genecards.org/cgi-bin/carddisp.pl?gene=SGCA |
| SKIC2 | SKI2 Subunit Of Superkiller Complex | Protein Coding | Q15477 | 54 | GC06P181637 | 1.621135 | https://www.genecards.org/cgi-bin/carddisp.pl?gene=SKIC2 |
| SLC37A4 | Solute Carrier Family 37 Member 4 | Protein Coding | O43826 | 54 | GC11M137443 | 1.621135 | https://www.genecards.org/cgi-bin/carddisp.pl?gene=SLC37A4 |
| SMN1 | Survival Of Motor Neuron 1, Telomeric | Protein Coding | Q16637 | 54 | GC05P070924 | 1.621135 | https://www.genecards.org/cgi-bin/carddisp.pl?gene=SMN1 |
| SPRED1 | Sprouty Related EVH1 Domain Containing 1 | Protein Coding | Q7Z699 | 54 | GC15P038252 | 1.621135 | https://www.genecards.org/cgi-bin/carddisp.pl?gene=SPRED1 |
| STX11 | Syntaxin 11 | Protein Coding | O75558 | 54 | GC06P174647 | 1.621135 | https://www.genecards.org/cgi-bin/carddisp.pl?gene=STX11 |
| SUMF1 | Sulfatase Modifying Factor 1 | Protein Coding | Q8NBK3 | 54 | GC03M003700 | 1.621135 | https://www.genecards.org/cgi-bin/carddisp.pl?gene=SUMF1 |
| TCAP | Titin-Cap | Protein Coding | O15273 | 54 | GC17P144370 | 1.621135 | https://www.genecards.org/cgi-bin/carddisp.pl?gene=TCAP |
| TK2 | Thymidine Kinase 2 | Protein Coding | O00142 | 54 | GC16M066508 | 1.621135 | https://www.genecards.org/cgi-bin/carddisp.pl?gene=TK2 |
| TNNC1 | Troponin C1, Slow Skeletal And Cardiac Type | Protein Coding | P63316 | 54 | GC03M054648 | 1.621135 | https://www.genecards.org/cgi-bin/carddisp.pl?gene=TNNC1 |
| TRIM63 | Tripartite Motif Containing 63 | Protein Coding | Q969Q1 | 54 | GC01M032764 | 1.621135 | https://www.genecards.org/cgi-bin/carddisp.pl?gene=TRIM63 |
| TSEN2 | TRNA Splicing Endonuclease Subunit 2 | Protein Coding | Q8NCE0 | 54 | GC03P025603 | 1.621135 | https://www.genecards.org/cgi-bin/carddisp.pl?gene=TSEN2 |
| TULP1 | TUB Like Protein 1 | Protein Coding | O00294 | 54 | GC06M103466 | 1.621135 | https://www.genecards.org/cgi-bin/carddisp.pl?gene=TULP1 |
| USH1C | USH1 Protein Network Component Harmonin | Protein Coding | Q9Y6N9 | 54 | GC11M018092 | 1.621135 | https://www.genecards.org/cgi-bin/carddisp.pl?gene=USH1C |
| ABCA12 | ATP Binding Cassette Subfamily A Member 12 | Protein Coding | Q86UK0 | 53 | GC02M214931 | 1.621135 | https://www.genecards.org/cgi-bin/carddisp.pl?gene=ABCA12 |
| ADGRV1 | Adhesion G Protein-Coupled Receptor V1 | Protein Coding | Q8WXG9 | 53 | GC05P090529 | 1.621135 | https://www.genecards.org/cgi-bin/carddisp.pl?gene=ADGRV1 |
| AHI1 | Abelson Helper Integration Site 1 | Protein Coding | Q8N157 | 53 | GC06M135283 | 1.621135 | https://www.genecards.org/cgi-bin/carddisp.pl?gene=AHI1 |
| AIPL1 | Aryl Hydrocarbon Receptor Interacting Protein Like 1 | Protein Coding | Q9NZN9 | 53 | GC17M006393 | 1.621135 | https://www.genecards.org/cgi-bin/carddisp.pl?gene=AIPL1 |
| ALOXE3 | Arachidonate Epidermal Lipoxygenase 3 | Protein Coding | Q9BYJ1 | 53 | GC17M092458 | 1.621135 | https://www.genecards.org/cgi-bin/carddisp.pl?gene=ALOXE3 |
| ANKRD1 | Ankyrin Repeat Domain 1 | Protein Coding | Q15327 | 53 | GC10M090912 | 1.621135 | https://www.genecards.org/cgi-bin/carddisp.pl?gene=ANKRD1 |
| BBS2 | Bardet-Biedl Syndrome 2 | Protein Coding | Q9BXC9 | 53 | GC16M056467 | 1.621135 | https://www.genecards.org/cgi-bin/carddisp.pl?gene=BBS2 |
| BBS9 | Bardet-Biedl Syndrome 9 | Protein Coding | Q3SYG4 | 53 | GC07P034104 | 1.621135 | https://www.genecards.org/cgi-bin/carddisp.pl?gene=BBS9 |
| BSND | Barttin CLCNK Type Accessory Subunit Beta | Protein Coding | Q8WZ55 | 53 | GC01P054998 | 1.621135 | https://www.genecards.org/cgi-bin/carddisp.pl?gene=BSND |
| CELSR1 | Cadherin EGF LAG Seven-Pass G-Type Receptor 1 | Protein Coding | Q9NYQ6 | 53 | GC22M046360 | 1.621135 | https://www.genecards.org/cgi-bin/carddisp.pl?gene=CELSR1 |
| CERS3 | Ceramide Synthase 3 | Protein Coding | Q8IU89 | 53 | GC15M159819 | 1.621135 | https://www.genecards.org/cgi-bin/carddisp.pl?gene=CERS3 |
| CHMP1A | Charged Multivesicular Body Protein 1A | Protein Coding | Q9HD42 | 53 | GC16M089644 | 1.621135 | https://www.genecards.org/cgi-bin/carddisp.pl?gene=CHMP1A |
| CHST6 | Carbohydrate Sulfotransferase 6 | Protein Coding | Q9GZX3 | 53 | GC16M075472 | 1.621135 | https://www.genecards.org/cgi-bin/carddisp.pl?gene=CHST6 |
| CLN5 | CLN5 Intracellular Trafficking Protein | Protein Coding | O75503 | 53 | GC13P076990 | 1.621135 | https://www.genecards.org/cgi-bin/carddisp.pl?gene=CLN5 |
| CLN8 | CLN8 Transmembrane ER And ERGIC Protein | Protein Coding | Q9UBY8 | 53 | GC08P001755 | 1.621135 | https://www.genecards.org/cgi-bin/carddisp.pl?gene=CLN8 |
| CNGB3 | Cyclic Nucleotide Gated Channel Subunit Beta 3 | Protein Coding | Q9NQW8 | 53 | GC08M086553 | 1.621135 | https://www.genecards.org/cgi-bin/carddisp.pl?gene=CNGB3 |
| DNAJC19 | DnaJ Heat Shock Protein Family (Hsp40) Member C19 | Protein Coding | Q96DA6 | 53 | GC03M180983 | 1.621135 | https://www.genecards.org/cgi-bin/carddisp.pl?gene=DNAJC19 |
| FANCB | FA Complementation Group B | Protein Coding | Q8NB91 | 53 | GC0XM016024 | 1.621135 | https://www.genecards.org/cgi-bin/carddisp.pl?gene=FANCB |
| FANCF | FA Complementation Group F | Protein Coding | Q9NPI8 | 53 | GC11M022600 | 1.621135 | https://www.genecards.org/cgi-bin/carddisp.pl?gene=FANCF |
| FOXN1 | Forkhead Box N1 | Protein Coding | O15353 | 53 | GC17P028506 | 1.621135 | https://www.genecards.org/cgi-bin/carddisp.pl?gene=FOXN1 |
| FOXRED1 | FAD Dependent Oxidoreductase Domain Containing 1 | Protein Coding | Q96CU9 | 53 | GC11P126269 | 1.621135 | https://www.genecards.org/cgi-bin/carddisp.pl?gene=FOXRED1 |
| GLE1 | GLE1 RNA Export Mediator | Protein Coding | Q53GS7 | 53 | GC09P128504 | 1.621135 | https://www.genecards.org/cgi-bin/carddisp.pl?gene=GLE1 |
| HAND1 | Heart And Neural Crest Derivatives Expressed 1 | Protein Coding | O96004 | 53 | GC05M154475 | 1.621135 | https://www.genecards.org/cgi-bin/carddisp.pl?gene=HAND1 |
| HPS1 | HPS1 Biogenesis Of Lysosomal Organelles Complex 3 Subunit 1 | Protein Coding | Q92902 | 53 | GC10M098410 | 1.621135 | https://www.genecards.org/cgi-bin/carddisp.pl?gene=HPS1 |
| INPP5E | Inositol Polyphosphate-5-Phosphatase E | Protein Coding | Q9NRR6 | 53 | GC09M136428 | 1.621135 | https://www.genecards.org/cgi-bin/carddisp.pl?gene=INPP5E |
| KIF14 | Kinesin Family Member 14 | Protein Coding | Q15058 | 53 | GC01M200551 | 1.621135 | https://www.genecards.org/cgi-bin/carddisp.pl?gene=KIF14 |
| LDLRAP1 | Low Density Lipoprotein Receptor Adaptor Protein 1 | Protein Coding | Q5SW96 | 53 | GC01P025543 | 1.621135 | https://www.genecards.org/cgi-bin/carddisp.pl?gene=LDLRAP1 |
| MCPH1 | Microcephalin 1 | Protein Coding | Q8NEM0 | 53 | GC08P006406 | 1.621135 | https://www.genecards.org/cgi-bin/carddisp.pl?gene=MCPH1 |
| MKS1 | MKS Transition Zone Complex Subunit 1 | Protein Coding | Q9NXB0 | 53 | GC17M058205 | 1.621135 | https://www.genecards.org/cgi-bin/carddisp.pl?gene=MKS1 |
| MLC1 | Modulator Of VRAC Current 1 | Protein Coding | Q15049 | 53 | GC22M050059 | 1.621135 | https://www.genecards.org/cgi-bin/carddisp.pl?gene=MLC1 |
| NBAS | NBAS Subunit Of NRZ Tethering Complex | Protein Coding | A2RRP1 | 53 | GC02M014802 | 1.621135 | https://www.genecards.org/cgi-bin/carddisp.pl?gene=NBAS |
| NEXN | Nexilin F-Actin Binding Protein | Protein Coding | Q0ZGT2 | 53 | GC01P078122 | 1.621135 | https://www.genecards.org/cgi-bin/carddisp.pl?gene=NEXN |
| OSTM1 | Osteoclastogenesis Associated Transmembrane Protein 1 | Protein Coding | Q86WC4 | 53 | GC06M108463 | 1.621135 | https://www.genecards.org/cgi-bin/carddisp.pl?gene=OSTM1 |
| OTOF | Otoferlin | Protein Coding | Q9HC10 | 53 | GC02M026659 | 1.621135 | https://www.genecards.org/cgi-bin/carddisp.pl?gene=OTOF |
| P3H1 | Prolyl 3-Hydroxylase 1 | Protein Coding | Q32P28 | 53 | GC01M042746 | 1.621135 | https://www.genecards.org/cgi-bin/carddisp.pl?gene=P3H1 |
| PEX10 | Peroxisomal Biogenesis Factor 10 | Protein Coding | O60683 | 53 | GC01M002403 | 1.621135 | https://www.genecards.org/cgi-bin/carddisp.pl?gene=PEX10 |
| PEX26 | Peroxisomal Biogenesis Factor 26 | Protein Coding | Q7Z412 | 53 | GC22P086254 | 1.621135 | https://www.genecards.org/cgi-bin/carddisp.pl?gene=PEX26 |
| PEX6 | Peroxisomal Biogenesis Factor 6 | Protein Coding | Q13608 | 53 | GC06M042963 | 1.621135 | https://www.genecards.org/cgi-bin/carddisp.pl?gene=PEX6 |
| RAG2 | Recombination Activating 2 | Protein Coding | P55895 | 53 | GC11M036575 | 1.621135 | https://www.genecards.org/cgi-bin/carddisp.pl?gene=RAG2 |
| RFX5 | Regulatory Factor X5 | Protein Coding | P48382 | 53 | GC01M151340 | 1.621135 | https://www.genecards.org/cgi-bin/carddisp.pl?gene=RFX5 |
| RP2 | RP2 Activator Of ARL3 GTPase | Protein Coding | O75695 | 53 | GC0XP046837 | 1.621135 | https://www.genecards.org/cgi-bin/carddisp.pl?gene=RP2 |
| RPGRIP1 | RPGR Interacting Protein 1 | Protein Coding | Q96KN7 | 53 | GC14P054459 | 1.621135 | https://www.genecards.org/cgi-bin/carddisp.pl?gene=RPGRIP1 |
| RS1 | Retinoschisin 1 | Protein Coding | O15537 | 53 | GC0XM018639 | 1.621135 | https://www.genecards.org/cgi-bin/carddisp.pl?gene=RS1 |
| SDCCAG8 | SHH Signaling And Ciliogenesis Regulator SDCCAG8 | Protein Coding | Q86SQ7 | 53 | GC01P243255 | 1.621135 | https://www.genecards.org/cgi-bin/carddisp.pl?gene=SDCCAG8 |
| SHOC2 | SHOC2 Leucine Rich Repeat Scaffold Protein | Protein Coding | Q9UQ13 | 53 | GC10P110919 | 1.621135 | https://www.genecards.org/cgi-bin/carddisp.pl?gene=SHOC2 |
| TBX20 | T-Box Transcription Factor 20 | Protein Coding | Q9UMR3 | 53 | GC07M035237 | 1.621135 | https://www.genecards.org/cgi-bin/carddisp.pl?gene=TBX20 |
| TMEM38B | Transmembrane Protein 38B | Protein Coding | Q9NVV0 | 53 | GC09P105694 | 1.621135 | https://www.genecards.org/cgi-bin/carddisp.pl?gene=TMEM38B |
| TRDN | Triadin | Protein Coding | Q13061 | 53 | GC06M123198 | 1.621135 | https://www.genecards.org/cgi-bin/carddisp.pl?gene=TRDN |
| VPS13A | Vacuolar Protein Sorting 13 Homolog A | Protein Coding | Q96RL7 | 53 | GC09P077177 | 1.621135 | https://www.genecards.org/cgi-bin/carddisp.pl?gene=VPS13A |
| VPS13B | Vacuolar Protein Sorting 13 Homolog B | Protein Coding | Q7Z7G8 | 53 | GC08P099154 | 1.621135 | https://www.genecards.org/cgi-bin/carddisp.pl?gene=VPS13B |
| VPS53 | VPS53 Subunit Of GARP Complex | Protein Coding | Q5VIR6 | 53 | GC17M000508 | 1.621135 | https://www.genecards.org/cgi-bin/carddisp.pl?gene=VPS53 |
| AAAS | Aladin WD Repeat Nucleoporin | Protein Coding | Q9NRG9 | 52 | GC12M053307 | 1.621135 | https://www.genecards.org/cgi-bin/carddisp.pl?gene=AAAS |
| ALMS1 | ALMS1 Centrosome And Basal Body Associated Protein | Protein Coding | Q8TCU4 | 52 | GC02P073385 | 1.621135 | https://www.genecards.org/cgi-bin/carddisp.pl?gene=ALMS1 |
| ARL6 | ARF Like GTPase 6 | Protein Coding | Q9H0F7 | 52 | GC03P097764 | 1.621135 | https://www.genecards.org/cgi-bin/carddisp.pl?gene=ARL6 |
| BBS1 | Bardet-Biedl Syndrome 1 | Protein Coding | Q8NFJ9 | 52 | GC11P103443 | 1.621135 | https://www.genecards.org/cgi-bin/carddisp.pl?gene=BBS1 |
| BBS10 | Bardet-Biedl Syndrome 10 | Protein Coding | Q8TAM1 | 52 | GC12M076344 | 1.621135 | https://www.genecards.org/cgi-bin/carddisp.pl?gene=BBS10 |
| CC2D2A | Coiled-Coil And C2 Domain Containing 2A | Protein Coding | Q9P2K1 | 52 | GC04P028372 | 1.621135 | https://www.genecards.org/cgi-bin/carddisp.pl?gene=CC2D2A |
| CCDC88C | Coiled-Coil Domain Containing 88C | Protein Coding | Q9P219 | 52 | GC14M091271 | 1.621135 | https://www.genecards.org/cgi-bin/carddisp.pl?gene=CCDC88C |
| CCN6 | Cellular Communication Network Factor 6 | Protein Coding | O95389 | 52 | GC06P174010 | 1.621135 | https://www.genecards.org/cgi-bin/carddisp.pl?gene=CCN6 |
| CDCA7 | Cell Division Cycle Associated 7 | Protein Coding | Q9BWT1 | 52 | GC02P173354 | 1.621135 | https://www.genecards.org/cgi-bin/carddisp.pl?gene=CDCA7 |
| CIB2 | Calcium And Integrin Binding Family Member 2 | Protein Coding | O75838 | 52 | GC15M078104 | 1.621135 | https://www.genecards.org/cgi-bin/carddisp.pl?gene=CIB2 |
| COLQ | Collagen Like Tail Subunit Of Asymmetric Acetylcholinesterase | Protein Coding | Q9Y215 | 52 | GC03M028105 | 1.621135 | https://www.genecards.org/cgi-bin/carddisp.pl?gene=COLQ |
| CTF1 | Cardiotrophin 1 | Protein Coding | Q16619 | 52 | GC16P113186 | 1.621135 | https://www.genecards.org/cgi-bin/carddisp.pl?gene=CTF1 |
| CUL7 | Cullin 7 | Protein Coding | Q14999 | 52 | GC06M043037 | 1.621135 | https://www.genecards.org/cgi-bin/carddisp.pl?gene=CUL7 |
| DOLK | Dolichol Kinase | Protein Coding | Q9UPQ8 | 52 | GC09M128945 | 1.621135 | https://www.genecards.org/cgi-bin/carddisp.pl?gene=DOLK |
| DYNC2H1 | Dynein Cytoplasmic 2 Heavy Chain 1 | Protein Coding | Q8NCM8 | 52 | GC11P103109 | 1.621135 | https://www.genecards.org/cgi-bin/carddisp.pl?gene=DYNC2H1 |
| ESCO2 | Establishment Of Sister Chromatid Cohesion N-Acetyltransferase 2 | Protein Coding | Q56NI9 | 52 | GC08P027771 | 1.621135 | https://www.genecards.org/cgi-bin/carddisp.pl?gene=ESCO2 |
| FKTN | Fukutin | Protein Coding | O75072 | 52 | GC09P105558 | 1.621135 | https://www.genecards.org/cgi-bin/carddisp.pl?gene=FKTN |
| FOXH1 | Forkhead Box H1 | Protein Coding | O75593 | 52 | GC08M144473 | 1.621135 | https://www.genecards.org/cgi-bin/carddisp.pl?gene=FOXH1 |
| HOGA1 | 4-Hydroxy-2-Oxoglutarate Aldolase 1 | Protein Coding | Q86XE5 | 52 | GC10P120254 | 1.621135 | https://www.genecards.org/cgi-bin/carddisp.pl?gene=HOGA1 |
| HPS4 | HPS4 Biogenesis Of Lysosomal Organelles Complex 3 Subunit 2 | Protein Coding | Q9NQG7 | 52 | GC22M026443 | 1.621135 | https://www.genecards.org/cgi-bin/carddisp.pl?gene=HPS4 |
| IFT140 | Intraflagellar Transport 140 | Protein Coding | Q96RY7 | 52 | GC16M046339 | 1.621135 | https://www.genecards.org/cgi-bin/carddisp.pl?gene=IFT140 |
| MED17 | Mediator Complex Subunit 17 | Protein Coding | Q9NVC6 | 52 | GC11P093784 | 1.621135 | https://www.genecards.org/cgi-bin/carddisp.pl?gene=MED17 |
| MYOM1 | Myomesin 1 | Protein Coding | P52179 | 52 | GC18M003066 | 1.621135 | https://www.genecards.org/cgi-bin/carddisp.pl?gene=MYOM1 |
| NEB | Nebulin | Protein Coding | P20929 | 52 | GC02M151485 | 1.621135 | https://www.genecards.org/cgi-bin/carddisp.pl?gene=NEB |
| NPHP3 | Nephrocystin 3 | Protein Coding | Q7Z494 | 52 | GC03M132683 | 1.621135 | https://www.genecards.org/cgi-bin/carddisp.pl?gene=NPHP3 |
| PEX11B | Peroxisomal Biogenesis Factor 11 Beta | Protein Coding | O96011 | 52 | GC01M145911 | 1.621135 | https://www.genecards.org/cgi-bin/carddisp.pl?gene=PEX11B |
| PEX13 | Peroxisomal Biogenesis Factor 13 | Protein Coding | Q92968 | 52 | GC02P061017 | 1.621135 | https://www.genecards.org/cgi-bin/carddisp.pl?gene=PEX13 |
| PLEKHG5 | Pleckstrin Homology And RhoGEF Domain Containing G5 | Protein Coding | O94827 | 52 | GC01M020796 | 1.621135 | https://www.genecards.org/cgi-bin/carddisp.pl?gene=PLEKHG5 |
| RASA2 | RAS P21 Protein Activator 2 | Protein Coding | Q15283 | 52 | GC03P141487 | 1.621135 | https://www.genecards.org/cgi-bin/carddisp.pl?gene=RASA2 |
| RNASEH2C | Ribonuclease H2 Subunit C | Protein Coding | Q8TDP1 | 52 | GC11M065714 | 1.621135 | https://www.genecards.org/cgi-bin/carddisp.pl?gene=RNASEH2C |
| SGCB | Sarcoglycan Beta | Protein Coding | Q16585 | 52 | GC04M052019 | 1.621135 | https://www.genecards.org/cgi-bin/carddisp.pl?gene=SGCB |
| SKIC3 | SKI3 Subunit Of Superkiller Complex | Protein Coding | Q6PGP7 | 52 | GC05M095461 | 1.621135 | https://www.genecards.org/cgi-bin/carddisp.pl?gene=SKIC3 |
| SLC35A3 | Solute Carrier Family 35 Member A3 | Protein Coding | Q9Y2D2 | 52 | GC01P100087 | 1.621135 | https://www.genecards.org/cgi-bin/carddisp.pl?gene=SLC35A3 |
| SLC4A11 | Solute Carrier Family 4 Member 11 | Protein Coding | Q8NBS3 | 52 | GC20M004311 | 1.621135 | https://www.genecards.org/cgi-bin/carddisp.pl?gene=SLC4A11 |
| SPG11 | SPG11 Vesicle Trafficking Associated, Spatacsin | Protein Coding | Q96JI7 | 52 | GC15M047072 | 1.621135 | https://www.genecards.org/cgi-bin/carddisp.pl?gene=SPG11 |
| TCTN2 | Tectonic Family Member 2 | Protein Coding | Q96GX1 | 52 | GC12P123671 | 1.621135 | https://www.genecards.org/cgi-bin/carddisp.pl?gene=TCTN2 |
| TCTN3 | Tectonic Family Member 3 | Protein Coding | Q6NUS6 | 52 | GC10M095663 | 1.621135 | https://www.genecards.org/cgi-bin/carddisp.pl?gene=TCTN3 |
| TMEM67 | Transmembrane Protein 67 | Protein Coding | Q5HYA8 | 52 | GC08P093754 | 1.621135 | https://www.genecards.org/cgi-bin/carddisp.pl?gene=TMEM67 |
| TTC8 | Tetratricopeptide Repeat Domain 8 | Protein Coding | Q8TAM2 | 52 | GC14P094379 | 1.621135 | https://www.genecards.org/cgi-bin/carddisp.pl?gene=TTC8 |
| VPS45 | Vacuolar Protein Sorting 45 Homolog | Protein Coding | Q9NRW7 | 52 | GC01P173149 | 1.621135 | https://www.genecards.org/cgi-bin/carddisp.pl?gene=VPS45 |
| ANO10 | Anoctamin 10 | Protein Coding | Q9NW15 | 51 | GC03M043355 | 1.621135 | https://www.genecards.org/cgi-bin/carddisp.pl?gene=ANO10 |
| CERKL | CERK Like Autophagy Regulator | Protein Coding | Q49MI3 | 51 | GC02M181536 | 1.621135 | https://www.genecards.org/cgi-bin/carddisp.pl?gene=CERKL |
| CWC27 | CWC27 Spliceosome Associated Cyclophilin | Protein Coding | Q6UX04 | 51 | GC05P064768 | 1.621135 | https://www.genecards.org/cgi-bin/carddisp.pl?gene=CWC27 |
| DCAF17 | DDB1 And CUL4 Associated Factor 17 | Protein Coding | Q5H9S7 | 51 | GC02P171434 | 1.621135 | https://www.genecards.org/cgi-bin/carddisp.pl?gene=DCAF17 |
| EVC2 | EvC Ciliary Complex Subunit 2 | Protein Coding | Q86UK5 | 51 | GC04M006130 | 1.621135 | https://www.genecards.org/cgi-bin/carddisp.pl?gene=EVC2 |
| FBXL4 | F-Box And Leucine Rich Repeat Protein 4 | Protein Coding | Q9UKA2 | 51 | GC06M098868 | 1.621135 | https://www.genecards.org/cgi-bin/carddisp.pl?gene=FBXL4 |
| HPS5 | HPS5 Biogenesis Of Lysosomal Organelles Complex 2 Subunit 2 | Protein Coding | Q9UPZ3 | 51 | GC11M018278 | 1.621135 | https://www.genecards.org/cgi-bin/carddisp.pl?gene=HPS5 |
| MKKS | MKKS Centrosomal Shuttling Protein | Protein Coding | Q9NPJ1 | 51 | GC20M010560 | 1.621135 | https://www.genecards.org/cgi-bin/carddisp.pl?gene=MKKS |
| MYOZ2 | Myozenin 2 | Protein Coding | Q9NPC6 | 51 | GC04P119135 | 1.621135 | https://www.genecards.org/cgi-bin/carddisp.pl?gene=MYOZ2 |
| NIPAL4 | NIPA Like Domain Containing 4 | Protein Coding | Q0D2K0 | 51 | GC05P157460 | 1.621135 | https://www.genecards.org/cgi-bin/carddisp.pl?gene=NIPAL4 |
| NOS1AP | Nitric Oxide Synthase 1 Adaptor Protein | Protein Coding | O75052 | 51 | GC01P162069 | 1.621135 | https://www.genecards.org/cgi-bin/carddisp.pl?gene=NOS1AP |
| OPA3 | Outer Mitochondrial Membrane Lipid Metabolism Regulator OPA3 | Protein Coding | Q9H6K4 | 51 | GC19M045527 | 1.621135 | https://www.genecards.org/cgi-bin/carddisp.pl?gene=OPA3 |
| PEX12 | Peroxisomal Biogenesis Factor 12 | Protein Coding | O00623 | 51 | GC17M035574 | 1.621135 | https://www.genecards.org/cgi-bin/carddisp.pl?gene=PEX12 |
| PROP1 | PROP Paired-Like Homeobox 1 | Protein Coding | O75360 | 51 | GC05M177992 | 1.621135 | https://www.genecards.org/cgi-bin/carddisp.pl?gene=PROP1 |
| SELENON | Selenoprotein N | Protein Coding | Q9NZV5 | 51 | GC01P025800 | 1.621135 | https://www.genecards.org/cgi-bin/carddisp.pl?gene=SELENON |
| SLMAP | Sarcolemma Associated Protein | Protein Coding | Q14BN4 | 51 | GC03P063439 | 1.621135 | https://www.genecards.org/cgi-bin/carddisp.pl?gene=SLMAP |
| STAP1 | Signal Transducing Adaptor Family Member 1 | Protein Coding | Q9ULZ2 | 51 | GC04P067558 | 1.621135 | https://www.genecards.org/cgi-bin/carddisp.pl?gene=STAP1 |
| TCTN1 | Tectonic Family Member 1 | Protein Coding | Q2MV58 | 51 | GC12P110614 | 1.621135 | https://www.genecards.org/cgi-bin/carddisp.pl?gene=TCTN1 |
| TSEN54 | TRNA Splicing Endonuclease Subunit 54 | Protein Coding | Q7Z6J9 | 51 | GC17P075515 | 1.621135 | https://www.genecards.org/cgi-bin/carddisp.pl?gene=TSEN54 |
| TTC7A | Tetratricopeptide Repeat Domain 7A | Protein Coding | Q9ULT0 | 51 | GC02P046925 | 1.621135 | https://www.genecards.org/cgi-bin/carddisp.pl?gene=TTC7A |
| ZFPM2 | Zinc Finger Protein, FOG Family Member 2 | Protein Coding | Q8WW38 | 51 | GC08P104590 | 1.621135 | https://www.genecards.org/cgi-bin/carddisp.pl?gene=ZFPM2 |
| ARL13B | ARF Like GTPase 13B | Protein Coding | Q3SXY8 | 50 | GC03P093980 | 1.621135 | https://www.genecards.org/cgi-bin/carddisp.pl?gene=ARL13B |
| B9D2 | B9 Domain Containing 2 | Protein Coding | Q9BPU9 | 50 | GC19M041354 | 1.621135 | https://www.genecards.org/cgi-bin/carddisp.pl?gene=B9D2 |
| BBS5 | Bardet-Biedl Syndrome 5 | Protein Coding | Q8N3I7 | 50 | GC02P169495 | 1.621135 | https://www.genecards.org/cgi-bin/carddisp.pl?gene=BBS5 |
| BBS7 | Bardet-Biedl Syndrome 7 | Protein Coding | Q8IWZ6 | 50 | GC04M121824 | 1.621135 | https://www.genecards.org/cgi-bin/carddisp.pl?gene=BBS7 |
| BLOC1S6 | Biogenesis Of Lysosomal Organelles Complex 1 Subunit 6 | Protein Coding | Q9UL45 | 50 | GC15P184236 | 1.621135 | https://www.genecards.org/cgi-bin/carddisp.pl?gene=BLOC1S6 |
| CEP104 | Centrosomal Protein 104 | Protein Coding | O60308 | 50 | GC01M003812 | 1.621135 | https://www.genecards.org/cgi-bin/carddisp.pl?gene=CEP104 |
| CLN6 | CLN6 Transmembrane ER Protein | Protein Coding | Q9NWW5 | 50 | GC15M068206 | 1.621135 | https://www.genecards.org/cgi-bin/carddisp.pl?gene=CLN6 |
| DOK7 | Docking Protein 7 | Protein Coding | Q18PE1 | 50 | GC04P009442 | 1.621135 | https://www.genecards.org/cgi-bin/carddisp.pl?gene=DOK7 |
| HPS3 | HPS3 Biogenesis Of Lysosomal Organelles Complex 2 Subunit 1 | Protein Coding | Q969F9 | 50 | GC03P149129 | 1.621135 | https://www.genecards.org/cgi-bin/carddisp.pl?gene=HPS3 |
| HYLS1 | HYLS1 Centriolar And Ciliogenesis Associated | Protein Coding | Q96M11 | 50 | GC11P125883 | 1.621135 | https://www.genecards.org/cgi-bin/carddisp.pl?gene=HYLS1 |
| KCTD7 | Potassium Channel Tetramerization Domain Containing 7 | Protein Coding | Q96MP8 | 50 | GC07P066628 | 1.621135 | https://www.genecards.org/cgi-bin/carddisp.pl?gene=KCTD7 |
| LCA5 | Lebercilin LCA5 | Protein Coding | Q86VQ0 | 50 | GC06M079484 | 1.621135 | https://www.genecards.org/cgi-bin/carddisp.pl?gene=LCA5 |
| LYST | Lysosomal Trafficking Regulator | Protein Coding | Q99698 | 50 | GC01M235661 | 1.621135 | https://www.genecards.org/cgi-bin/carddisp.pl?gene=LYST |
| NEBL | Nebulette | Protein Coding | O76041 | 50 | GC10M020779 | 1.621135 | https://www.genecards.org/cgi-bin/carddisp.pl?gene=NEBL |
| OBSL1 | Obscurin Like Cytoskeletal Adaptor 1 | Protein Coding | O75147 | 50 | GC02M219641 | 1.621135 | https://www.genecards.org/cgi-bin/carddisp.pl?gene=OBSL1 |
| PDLIM3 | PDZ And LIM Domain 3 | Protein Coding | Q53GG5 | 50 | GC04M185500 | 1.621135 | https://www.genecards.org/cgi-bin/carddisp.pl?gene=PDLIM3 |
| PEX16 | Peroxisomal Biogenesis Factor 16 | Protein Coding | Q9Y5Y5 | 50 | GC11M136213 | 1.621135 | https://www.genecards.org/cgi-bin/carddisp.pl?gene=PEX16 |
| PKHD1 | PKHD1 Ciliary IPT Domain Containing Fibrocystin/Polyductin | Protein Coding | P08F94 | 50 | GC06M103646 | 1.621135 | https://www.genecards.org/cgi-bin/carddisp.pl?gene=PKHD1 |
| POU3F4 | POU Class 3 Homeobox 4 | Protein Coding | P49335 | 50 | GC0XP083508 | 1.621135 | https://www.genecards.org/cgi-bin/carddisp.pl?gene=POU3F4 |
| RFXANK | Regulatory Factor X Associated Ankyrin Containing Protein | Protein Coding | O14593 | 50 | GC19P019192 | 1.621135 | https://www.genecards.org/cgi-bin/carddisp.pl?gene=RFXANK |
| SACS | Sacsin Molecular Chaperone | Protein Coding | Q9NZJ4 | 50 | GC13M023288 | 1.621135 | https://www.genecards.org/cgi-bin/carddisp.pl?gene=SACS |
| SP110 | SP110 Nuclear Body Protein | Protein Coding | Q9HB58 | 50 | GC02M230167 | 1.621135 | https://www.genecards.org/cgi-bin/carddisp.pl?gene=SP110 |
| SPG21 | SPG21 Abhydrolase Domain Containing, Maspardin | Protein Coding | Q9NZD8 | 50 | GC15M064963 | 1.621135 | https://www.genecards.org/cgi-bin/carddisp.pl?gene=SPG21 |
| TMEM138 | Transmembrane Protein 138 | Protein Coding | Q9NPI0 | 50 | GC11P102983 | 1.621135 | https://www.genecards.org/cgi-bin/carddisp.pl?gene=TMEM138 |
| TSEN34 | TRNA Splicing Endonuclease Subunit 34 | Protein Coding | Q9BSV6 | 50 | GC19P151281 | 1.621135 | https://www.genecards.org/cgi-bin/carddisp.pl?gene=TSEN34 |
| USH1G | USH1 Protein Network Component Sans | Protein Coding | Q495M9 | 50 | GC17M074916 | 1.621135 | https://www.genecards.org/cgi-bin/carddisp.pl?gene=USH1G |
| ZBTB24 | Zinc Finger And BTB Domain Containing 24 | Protein Coding | O43167 | 50 | GC06M109504 | 1.621135 | https://www.genecards.org/cgi-bin/carddisp.pl?gene=ZBTB24 |
| CTC1 | CST Telomere Replication Complex Component 1 | Protein Coding | Q2NKJ3 | 49 | GC17M092479 | 1.621135 | https://www.genecards.org/cgi-bin/carddisp.pl?gene=CTC1 |
| FRAS1 | Fraser Extracellular Matrix Complex Subunit 1 | Protein Coding | Q86XX4 | 49 | GC04P078057 | 1.621135 | https://www.genecards.org/cgi-bin/carddisp.pl?gene=FRAS1 |
| FREM2 | FRAS1 Related Extracellular Matrix 2 | Protein Coding | Q5SZK8 | 49 | GC13P038687 | 1.621135 | https://www.genecards.org/cgi-bin/carddisp.pl?gene=FREM2 |
| GDF1 | Growth Differentiation Factor 1 | Protein Coding | P27539 | 49 | GC19M104640 | 1.621135 | https://www.genecards.org/cgi-bin/carddisp.pl?gene=GDF1 |
| GNPTG | N-Acetylglucosamine-1-Phosphate Transferase Subunit Gamma | Protein Coding | Q9UJJ9 | 49 | GC16P001351 | 1.621135 | https://www.genecards.org/cgi-bin/carddisp.pl?gene=GNPTG |
| HGSNAT | Heparan-Alpha-Glucosaminide N-Acetyltransferase | Protein Coding | Q68CP4 | 49 | GC08P043140 | 1.621135 | https://www.genecards.org/cgi-bin/carddisp.pl?gene=HGSNAT |
| HPS6 | HPS6 Biogenesis Of Lysosomal Organelles Complex 2 Subunit 3 | Protein Coding | Q86YV9 | 49 | GC10P102065 | 1.621135 | https://www.genecards.org/cgi-bin/carddisp.pl?gene=HPS6 |
| LIPN | Lipase Family Member N | Protein Coding | Q5VXI9 | 49 | GC10P120083 | 1.621135 | https://www.genecards.org/cgi-bin/carddisp.pl?gene=LIPN |
| MFSD8 | Major Facilitator Superfamily Domain Containing 8 | Protein Coding | Q8NHS3 | 49 | GC04M127917 | 1.621135 | https://www.genecards.org/cgi-bin/carddisp.pl?gene=MFSD8 |
| NDUFAF5 | NADH:Ubiquinone Oxidoreductase Complex Assembly Factor 5 | Protein Coding | Q5TEU4 | 49 | GC20P014051 | 1.621135 | https://www.genecards.org/cgi-bin/carddisp.pl?gene=NDUFAF5 |
| RBM20 | RNA Binding Motif Protein 20 | Protein Coding | Q5T481 | 49 | GC10P120700 | 1.621135 | https://www.genecards.org/cgi-bin/carddisp.pl?gene=RBM20 |
| RNASEH2B | Ribonuclease H2 Subunit B | Protein Coding | Q5TBB1 | 49 | GC13P050909 | 1.621135 | https://www.genecards.org/cgi-bin/carddisp.pl?gene=RNASEH2B |
| SPATA7 | Spermatogenesis Associated 7 | Protein Coding | Q9P0W8 | 49 | GC14P088384 | 1.621135 | https://www.genecards.org/cgi-bin/carddisp.pl?gene=SPATA7 |
| TMEM231 | Transmembrane Protein 231 | Protein Coding | Q9H6L2 | 49 | GC16M075536 | 1.621135 | https://www.genecards.org/cgi-bin/carddisp.pl?gene=TMEM231 |
| TMEM237 | Transmembrane Protein 237 | Protein Coding | Q96Q45 | 49 | GC02M201620 | 1.621135 | https://www.genecards.org/cgi-bin/carddisp.pl?gene=TMEM237 |
| TRAPPC11 | Trafficking Protein Particle Complex Subunit 11 | Protein Coding | Q7Z392 | 49 | GC04P183659 | 1.621135 | https://www.genecards.org/cgi-bin/carddisp.pl?gene=TRAPPC11 |
| CALR3 | Calreticulin 3 | Protein Coding | Q96L12 | 48 | GC19M104566 | 1.621135 | https://www.genecards.org/cgi-bin/carddisp.pl?gene=CALR3 |
| CCDC8 | Coiled-Coil Domain Containing 8 | Protein Coding | Q9H0W5 | 48 | GC19M046410 | 1.621135 | https://www.genecards.org/cgi-bin/carddisp.pl?gene=CCDC8 |
| HYCC1 | Hyccin PI4KA Lipid Kinase Complex Subunit 1 | Protein Coding | Q9BYI3 | 48 | GC07M022944 | 1.621135 | https://www.genecards.org/cgi-bin/carddisp.pl?gene=HYCC1 |
| NKX2-6 | NK2 Homeobox 6 | Protein Coding | A6NCS4 | 48 | GC08M023702 | 1.621135 | https://www.genecards.org/cgi-bin/carddisp.pl?gene=NKX2-6 |
| RD3 | RD3 Regulator Of GUCY2D | Protein Coding | Q7Z3Z2 | 48 | GC01M211476 | 1.621135 | https://www.genecards.org/cgi-bin/carddisp.pl?gene=RD3 |
| ALPK3 | Alpha Kinase 3 | Protein Coding | Q96L96 | 47 | GC15P185061 | 1.621135 | https://www.genecards.org/cgi-bin/carddisp.pl?gene=ALPK3 |
| B9D1 | B9 Domain Containing 1 | Protein Coding | Q9UPM9 | 47 | GC17M019334 | 1.621135 | https://www.genecards.org/cgi-bin/carddisp.pl?gene=B9D1 |
| BLOC1S3 | Biogenesis Of Lysosomal Organelles Complex 1 Subunit 3 | Protein Coding | Q6QNY0 | 47 | GC19P045178 | 1.621135 | https://www.genecards.org/cgi-bin/carddisp.pl?gene=BLOC1S3 |
| C19orf12 | Chromosome 19 Open Reading Frame 12 | Protein Coding | Q9NSK7 | 47 | GC19M104809 | 1.621135 | https://www.genecards.org/cgi-bin/carddisp.pl?gene=C19orf12 |
| CLRN1 | Clarin 1 | Protein Coding | P58418 | 47 | GC03M150926 | 1.621135 | https://www.genecards.org/cgi-bin/carddisp.pl?gene=CLRN1 |
| CYP4F22 | Cytochrome P450 Family 4 Subfamily F Member 22 | Protein Coding | Q6NT55 | 47 | GC19P015508 | 1.621135 | https://www.genecards.org/cgi-bin/carddisp.pl?gene=CYP4F22 |
| PNPLA1 | Patatin Like Phospholipase Domain Containing 1 | Protein Coding | Q8N8W4 | 47 | GC06P173202 | 1.621135 | https://www.genecards.org/cgi-bin/carddisp.pl?gene=PNPLA1 |
| PRDM5 | PR/SET Domain 5 | Protein Coding | Q9NQX1 | 47 | GC04M121298 | 1.621135 | https://www.genecards.org/cgi-bin/carddisp.pl?gene=PRDM5 |
| SDR9C7 | Short Chain Dehydrogenase/Reductase Family 9C Member 7 | Protein Coding | Q8NEX9 | 47 | GC12M056923 | 1.621135 | https://www.genecards.org/cgi-bin/carddisp.pl?gene=SDR9C7 |
| TECPR2 | Tectonin Beta-Propeller Repeat Containing 2 | Protein Coding | O15040 | 47 | GC14P102362 | 1.621135 | https://www.genecards.org/cgi-bin/carddisp.pl?gene=TECPR2 |
| TMEM216 | Transmembrane Protein 216 | Protein Coding | Q9P0N5 | 47 | GC11P102979 | 1.621135 | https://www.genecards.org/cgi-bin/carddisp.pl?gene=TMEM216 |
| WHRN | Whirlin | Protein Coding | Q9P202 | 47 | GC09M120438 | 1.621135 | https://www.genecards.org/cgi-bin/carddisp.pl?gene=WHRN |
| BBS12 | Bardet-Biedl Syndrome 12 | Protein Coding | Q6ZW61 | 46 | GC04P122702 | 1.621135 | https://www.genecards.org/cgi-bin/carddisp.pl?gene=BBS12 |
| CPLANE1 | Ciliogenesis And Planar Polarity Effector Complex Subunit 1 | Protein Coding | Q9H799 | 46 | GC05M038689 | 1.621135 | https://www.genecards.org/cgi-bin/carddisp.pl?gene=CPLANE1 |
| EYS | Eyes Shut Homolog | Protein Coding | Q5T1H1 | 46 | GC06M063719 | 1.621135 | https://www.genecards.org/cgi-bin/carddisp.pl?gene=EYS |
| FAM161A | FAM161 Centrosomal Protein A | Protein Coding | Q3B820 | 46 | GC02M061792 | 1.621135 | https://www.genecards.org/cgi-bin/carddisp.pl?gene=FAM161A |
| KCNE5 | Potassium Voltage-Gated Channel Subfamily E Regulatory Subunit 5 | Protein Coding | Q9UJ90 | 46 | GC0XM109623 | 1.621135 | https://www.genecards.org/cgi-bin/carddisp.pl?gene=KCNE5 |
| LOXHD1 | Lipoxygenase Homology PLAT Domains 1 | Protein Coding | Q8IVV2 | 46 | GC18M046476 | 1.621135 | https://www.genecards.org/cgi-bin/carddisp.pl?gene=LOXHD1 |
| MESP2 | Mesoderm Posterior BHLH Transcription Factor 2 | Protein Coding | Q0VG99 | 46 | GC15P185173 | 1.621135 | https://www.genecards.org/cgi-bin/carddisp.pl?gene=MESP2 |
| RANGRF | RAN Guanine Nucleotide Release Factor | Protein Coding | Q9HD47 | 46 | GC17P008288 | 1.621135 | https://www.genecards.org/cgi-bin/carddisp.pl?gene=RANGRF |
| RFXAP | Regulatory Factor X Associated Protein | Protein Coding | O00287 | 46 | GC13P036819 | 1.621135 | https://www.genecards.org/cgi-bin/carddisp.pl?gene=RFXAP |
| TECRL | Trans-2,3-Enoyl-CoA Reductase Like | Protein Coding | Q5HYJ1 | 46 | GC04M064275 | 1.621135 | https://www.genecards.org/cgi-bin/carddisp.pl?gene=TECRL |
| SYNE4 | Spectrin Repeat Containing Nuclear Envelope Family Member 4 | Protein Coding | Q8N205 | 42 | GC19M036003 | 1.621135 | https://www.genecards.org/cgi-bin/carddisp.pl?gene=SYNE4 |
| ZNF469 | Zinc Finger Protein 469 | Protein Coding | Q96JG9 | 42 | GC16P114893 | 1.621135 | https://www.genecards.org/cgi-bin/carddisp.pl?gene=ZNF469 |
| PRCD | Photoreceptor Disc Component | Protein Coding | Q00LT1 | 40 | GC17P076527 | 1.621135 | https://www.genecards.org/cgi-bin/carddisp.pl?gene=PRCD |
| CFAP92 | Cilia And Flagella Associated Protein 92 (Putative) | Protein Coding | Q9ULG3 | 39 | GC03M132354 | 1.621135 | https://www.genecards.org/cgi-bin/carddisp.pl?gene=CFAP92 |
| CTH | Cystathionine Gamma-Lyase | Protein Coding | P32929 | 63 | GC01P070411 | 1.524784 | https://www.genecards.org/cgi-bin/carddisp.pl?gene=CTH |
| GRIA4 | Glutamate Ionotropic Receptor AMPA Type Subunit 4 | Protein Coding | P48058 | 62 | GC11P105609 | 1.524784 | https://www.genecards.org/cgi-bin/carddisp.pl?gene=GRIA4 |
| SCN4A | Sodium Voltage-Gated Channel Alpha Subunit 4 | Protein Coding | P35499 | 59 | GC17M063938 | 1.524784 | https://www.genecards.org/cgi-bin/carddisp.pl?gene=SCN4A |
| MOGS | Mannosyl-Oligosaccharide Glucosidase | Protein Coding | Q13724 | 57 | GC02M074461 | 1.524784 | https://www.genecards.org/cgi-bin/carddisp.pl?gene=MOGS |
| HAGH | Hydroxyacylglutathione Hydrolase | Protein Coding | Q16775 | 55 | GC16M001795 | 1.524784 | https://www.genecards.org/cgi-bin/carddisp.pl?gene=HAGH |
| PUF60 | Poly(U) Binding Splicing Factor 60 | Protein Coding | Q9UHX1 | 54 | GC08M143816 | 1.524784 | https://www.genecards.org/cgi-bin/carddisp.pl?gene=PUF60 |
| SLC30A9 | Solute Carrier Family 30 Member 9 | Protein Coding | Q6PML9 | 54 | GC04P041998 | 1.524784 | https://www.genecards.org/cgi-bin/carddisp.pl?gene=SLC30A9 |
| CARS2 | Cysteinyl-TRNA Synthetase 2, Mitochondrial | Protein Coding | Q9HA77 | 53 | GC13M110641 | 1.524784 | https://www.genecards.org/cgi-bin/carddisp.pl?gene=CARS2 |
| MPST | Mercaptopyruvate Sulfurtransferase | Protein Coding | P25325 | 53 | GC22P037019 | 1.524784 | https://www.genecards.org/cgi-bin/carddisp.pl?gene=MPST |
| SLC6A17 | Solute Carrier Family 6 Member 17 | Protein Coding | Q9H1V8 | 53 | GC01P110150 | 1.524784 | https://www.genecards.org/cgi-bin/carddisp.pl?gene=SLC6A17 |
| TST | Thiosulfate Sulfurtransferase | Protein Coding | Q16762 | 53 | GC22M037010 | 1.524784 | https://www.genecards.org/cgi-bin/carddisp.pl?gene=TST |
| PCGF2 | Polycomb Group Ring Finger 2 | Protein Coding | P35227 | 52 | GC17M038733 | 1.524784 | https://www.genecards.org/cgi-bin/carddisp.pl?gene=PCGF2 |
| SLC30A5 | Solute Carrier Family 30 Member 5 | Protein Coding | Q8TAD4 | 52 | GC05P069093 | 1.524784 | https://www.genecards.org/cgi-bin/carddisp.pl?gene=SLC30A5 |
| SLC39A13 | Solute Carrier Family 39 Member 13 | Protein Coding | Q96H72 | 52 | GC11P047407 | 1.524784 | https://www.genecards.org/cgi-bin/carddisp.pl?gene=SLC39A13 |
| CDO1 | Cysteine Dioxygenase Type 1 | Protein Coding | Q16878 | 51 | GC05M115804 | 1.524784 | https://www.genecards.org/cgi-bin/carddisp.pl?gene=CDO1 |
| PQBP1 | Polyglutamine Binding Protein 1 | Protein Coding | O60828 | 51 | GC0XP048890 | 1.524784 | https://www.genecards.org/cgi-bin/carddisp.pl?gene=PQBP1 |
| SLC39A10 | Solute Carrier Family 39 Member 10 | Protein Coding | Q9ULF5 | 51 | GC02P195575 | 1.524784 | https://www.genecards.org/cgi-bin/carddisp.pl?gene=SLC39A10 |
| SLC39A5 | Solute Carrier Family 39 Member 5 | Protein Coding | Q6ZMH5 | 51 | GC12P069900 | 1.524784 | https://www.genecards.org/cgi-bin/carddisp.pl?gene=SLC39A5 |
| BRAT1 | BRCA1 Associated ATM Activator 1 | Protein Coding | Q6PJG6 | 50 | GC07M003872 | 1.524784 | https://www.genecards.org/cgi-bin/carddisp.pl?gene=BRAT1 |
| KCTD3 | Potassium Channel Tetramerization Domain Containing 3 | Protein Coding | Q9Y597 | 50 | GC01P215567 | 1.524784 | https://www.genecards.org/cgi-bin/carddisp.pl?gene=KCTD3 |
| MOCS3 | Molybdenum Cofactor Synthesis 3 | Protein Coding | O95396 | 50 | GC20P050958 | 1.524784 | https://www.genecards.org/cgi-bin/carddisp.pl?gene=MOCS3 |
| SLC30A6 | Solute Carrier Family 30 Member 6 | Protein Coding | Q6NXT4 | 50 | GC02P032166 | 1.524784 | https://www.genecards.org/cgi-bin/carddisp.pl?gene=SLC30A6 |
| SLC30A7 | Solute Carrier Family 30 Member 7 | Protein Coding | Q8NEW0 | 50 | GC01P100896 | 1.524784 | https://www.genecards.org/cgi-bin/carddisp.pl?gene=SLC30A7 |
| CENPC | Centromere Protein C | Protein Coding | Q03188 | 49 | GC04M067512 | 1.524784 | https://www.genecards.org/cgi-bin/carddisp.pl?gene=CENPC |
| SAMD9L | Sterile Alpha Motif Domain Containing 9 Like | Protein Coding | Q8IVG5 | 49 | GC07M093130 | 1.524784 | https://www.genecards.org/cgi-bin/carddisp.pl?gene=SAMD9L |
| CENPT | Centromere Protein T | Protein Coding | Q96BT3 | 48 | GC16M067828 | 1.524784 | https://www.genecards.org/cgi-bin/carddisp.pl?gene=CENPT |
| CENPU | Centromere Protein U | Protein Coding | Q71F23 | 47 | GC04M184694 | 1.524784 | https://www.genecards.org/cgi-bin/carddisp.pl?gene=CENPU |
| SLC39A9 | Solute Carrier Family 39 Member 9 | Protein Coding | Q9NUM3 | 47 | GC14P069398 | 1.524784 | https://www.genecards.org/cgi-bin/carddisp.pl?gene=SLC39A9 |
| ZBTB11 | Zinc Finger And BTB Domain Containing 11 | Protein Coding | O95625 | 47 | GC03M101648 | 1.524784 | https://www.genecards.org/cgi-bin/carddisp.pl?gene=ZBTB11 |
| ZNHIT3 | Zinc Finger HIT-Type Containing 3 | Protein Coding | Q15649 | 47 | GC17P036486 | 1.524784 | https://www.genecards.org/cgi-bin/carddisp.pl?gene=ZNHIT3 |
| GPR179 | G Protein-Coupled Receptor 179 | Protein Coding | Q6PRD1 | 46 | GC17M093277 | 1.524784 | https://www.genecards.org/cgi-bin/carddisp.pl?gene=GPR179 |
| LIPT2 | Lipoyl(Octanoyl) Transferase 2 | Protein Coding | A6NK58 | 46 | GC11M136852 | 1.524784 | https://www.genecards.org/cgi-bin/carddisp.pl?gene=LIPT2 |
| SLC39A3 | Solute Carrier Family 39 Member 3 | Protein Coding | Q9BRY0 | 46 | GC19M002732 | 1.524784 | https://www.genecards.org/cgi-bin/carddisp.pl?gene=SLC39A3 |
| AASDH | Aminoadipate-Semialdehyde Dehydrogenase | Protein Coding | Q4L235 | 45 | GC04M056340 | 1.524784 | https://www.genecards.org/cgi-bin/carddisp.pl?gene=AASDH |
| CD302 | CD302 Molecule | Protein Coding | Q8IX05 | 45 | GC02M159769 | 1.524784 | https://www.genecards.org/cgi-bin/carddisp.pl?gene=CD302 |
| TSTD1 | Thiosulfate Sulfurtransferase Like Domain Containing 1 | Protein Coding | Q8NFU3 | 45 | GC01M161037 | 1.524784 | https://www.genecards.org/cgi-bin/carddisp.pl?gene=TSTD1 |
| ZNF12 | Zinc Finger Protein 12 | Protein Coding | P17014 | 45 | GC07M009752 | 1.524784 | https://www.genecards.org/cgi-bin/carddisp.pl?gene=ZNF12 |
| CENPQ | Centromere Protein Q | Protein Coding | Q7L2Z9 | 43 | GC06P173304 | 1.524784 | https://www.genecards.org/cgi-bin/carddisp.pl?gene=CENPQ |
| DSN1 | DSN1 Component Of MIS12 Kinetochore Complex | Protein Coding | Q9H410 | 42 | GC20M036751 | 1.524784 | https://www.genecards.org/cgi-bin/carddisp.pl?gene=DSN1 |
| SLC39A11 | Solute Carrier Family 39 Member 11 | Protein Coding | Q8N1S5 | 42 | GC17M072645 | 1.524784 | https://www.genecards.org/cgi-bin/carddisp.pl?gene=SLC39A11 |
| EBF4 | EBF Family Member 4 | Protein Coding | Q9BQW3 | 41 | GC20P010161 | 1.524784 | https://www.genecards.org/cgi-bin/carddisp.pl?gene=EBF4 |
| OR8J1 | Olfactory Receptor Family 8 Subfamily J Member 1 | Protein Coding | Q8NGP2 | 38 | GC11P056360 | 1.524784 | https://www.genecards.org/cgi-bin/carddisp.pl?gene=OR8J1 |
| DNMT1 | DNA Methyltransferase 1 | Protein Coding | P26358 | 66 | GC19M010133 | 1.507174 | https://www.genecards.org/cgi-bin/carddisp.pl?gene=DNMT1 |
| EZH2 | Enhancer Of Zeste 2 Polycomb Repressive Complex 2 Subunit | Protein Coding | Q15910 | 66 | GC07M148807 | 1.507174 | https://www.genecards.org/cgi-bin/carddisp.pl?gene=EZH2 |
| PSEN1 | Presenilin 1 | Protein Coding | P49768 | 66 | GC14P073136 | 1.507174 | https://www.genecards.org/cgi-bin/carddisp.pl?gene=PSEN1 |
| STAT1 | Signal Transducer And Activator Of Transcription 1 | Protein Coding | P42224 | 66 | GC02M190908 | 1.507174 | https://www.genecards.org/cgi-bin/carddisp.pl?gene=STAT1 |
| DNMT3A | DNA Methyltransferase 3 Alpha | Protein Coding | Q9Y6K1 | 65 | GC02M025228 | 1.507174 | https://www.genecards.org/cgi-bin/carddisp.pl?gene=DNMT3A |
| COMT | Catechol-O-Methyltransferase | Protein Coding | P21964 | 64 | GC22P019941 | 1.507174 | https://www.genecards.org/cgi-bin/carddisp.pl?gene=COMT |
| ADK | Adenosine Kinase | Protein Coding | P55263 | 63 | GC10P075599 | 1.507174 | https://www.genecards.org/cgi-bin/carddisp.pl?gene=ADK |
| P4HB | Prolyl 4-Hydroxylase Subunit Beta | Protein Coding | P07237 | 63 | GC17M081843 | 1.507174 | https://www.genecards.org/cgi-bin/carddisp.pl?gene=P4HB |
| MTAP | Methylthioadenosine Phosphorylase | Protein Coding | Q13126 | 62 | GC09P021925 | 1.507174 | https://www.genecards.org/cgi-bin/carddisp.pl?gene=MTAP |
| AHCY | Adenosylhomocysteinase | Protein Coding | P23526 | 61 | GC20M036643 | 1.507174 | https://www.genecards.org/cgi-bin/carddisp.pl?gene=AHCY |
| KMT2A | Lysine Methyltransferase 2A | Protein Coding | Q03164 | 61 | GC11P118436 | 1.507174 | https://www.genecards.org/cgi-bin/carddisp.pl?gene=KMT2A |
| ODC1 | Ornithine Decarboxylase 1 | Protein Coding | P11926 | 61 | GC02M010432 | 1.507174 | https://www.genecards.org/cgi-bin/carddisp.pl?gene=ODC1 |
| PRMT1 | Protein Arginine Methyltransferase 1 | Protein Coding | Q99873 | 61 | GC19P049675 | 1.507174 | https://www.genecards.org/cgi-bin/carddisp.pl?gene=PRMT1 |
| APRT | Adenine Phosphoribosyltransferase | Protein Coding | P07741 | 60 | GC16M088810 | 1.507174 | https://www.genecards.org/cgi-bin/carddisp.pl?gene=APRT |
| CARM1 | Coactivator Associated Arginine Methyltransferase 1 | Protein Coding | Q86X55 | 60 | GC19P010871 | 1.507174 | https://www.genecards.org/cgi-bin/carddisp.pl?gene=CARM1 |
| PRMT5 | Protein Arginine Methyltransferase 5 | Protein Coding | O14744 | 60 | GC14M022920 | 1.507174 | https://www.genecards.org/cgi-bin/carddisp.pl?gene=PRMT5 |
| SETD2 | SET Domain Containing 2, Histone Lysine Methyltransferase | Protein Coding | Q9BYW2 | 60 | GC03M047033 | 1.507174 | https://www.genecards.org/cgi-bin/carddisp.pl?gene=SETD2 |
| SLC19A1 | Solute Carrier Family 19 Member 1 | Protein Coding | P41440 | 60 | GC21M045493 | 1.507174 | https://www.genecards.org/cgi-bin/carddisp.pl?gene=SLC19A1 |
| CYP2E1 | Cytochrome P450 Family 2 Subfamily E Member 1 | Protein Coding | P05181 | 59 | GC10P133520 | 1.507174 | https://www.genecards.org/cgi-bin/carddisp.pl?gene=CYP2E1 |
| EHMT1 | Euchromatic Histone Lysine Methyltransferase 1 | Protein Coding | Q9H9B1 | 59 | GC09P137618 | 1.507174 | https://www.genecards.org/cgi-bin/carddisp.pl?gene=EHMT1 |
| SAT1 | Spermidine/Spermine N1-Acetyltransferase 1 | Protein Coding | P21673 | 59 | GC0XP023784 | 1.507174 | https://www.genecards.org/cgi-bin/carddisp.pl?gene=SAT1 |
| TPMT | Thiopurine S-Methyltransferase | Protein Coding | P51580 | 59 | GC06M018128 | 1.507174 | https://www.genecards.org/cgi-bin/carddisp.pl?gene=TPMT |
| EHMT2 | Euchromatic Histone Lysine Methyltransferase 2 | Protein Coding | Q96KQ7 | 58 | GC06M031879 | 1.507174 | https://www.genecards.org/cgi-bin/carddisp.pl?gene=EHMT2 |
| HNMT | Histamine N-Methyltransferase | Protein Coding | P50135 | 58 | GC02P137964 | 1.507174 | https://www.genecards.org/cgi-bin/carddisp.pl?gene=HNMT |
| NSD2 | Nuclear Receptor Binding SET Domain Protein 2 | Protein Coding | O96028 | 58 | GC04P009308 | 1.507174 | https://www.genecards.org/cgi-bin/carddisp.pl?gene=NSD2 |
| PTPA | Protein Phosphatase 2 Phosphatase Activator | Protein Coding | Q15257 | 58 | GC09P149801 | 1.507174 | https://www.genecards.org/cgi-bin/carddisp.pl?gene=PTPA |
| DOT1L | DOT1 Like Histone Lysine Methyltransferase | Protein Coding | Q8TEK3 | 57 | GC19P141872 | 1.507174 | https://www.genecards.org/cgi-bin/carddisp.pl?gene=DOT1L |
| EZH1 | Enhancer Of Zeste 1 Polycomb Repressive Complex 2 Subunit | Protein Coding | Q92800 | 57 | GC17M042700 | 1.507174 | https://www.genecards.org/cgi-bin/carddisp.pl?gene=EZH1 |
| GNMT | Glycine N-Methyltransferase | Protein Coding | Q14749 | 57 | GC06P042960 | 1.507174 | https://www.genecards.org/cgi-bin/carddisp.pl?gene=GNMT |
| KMT2D | Lysine Methyltransferase 2D | Protein Coding | O14686 | 57 | GC12M049018 | 1.507174 | https://www.genecards.org/cgi-bin/carddisp.pl?gene=KMT2D |
| NNMT | Nicotinamide N-Methyltransferase | Protein Coding | P40261 | 57 | GC11P114257 | 1.507174 | https://www.genecards.org/cgi-bin/carddisp.pl?gene=NNMT |
| NSD1 | Nuclear Receptor Binding SET Domain Protein 1 | Protein Coding | Q96L73 | 57 | GC05P189455 | 1.507174 | https://www.genecards.org/cgi-bin/carddisp.pl?gene=NSD1 |
| PNMT | Phenylethanolamine N-Methyltransferase | Protein Coding | P11086 | 57 | GC17P039667 | 1.507174 | https://www.genecards.org/cgi-bin/carddisp.pl?gene=PNMT |
| RELN | Reelin | Protein Coding | P78509 | 57 | GC07M103471 | 1.507174 | https://www.genecards.org/cgi-bin/carddisp.pl?gene=RELN |
| SETD7 | SET Domain Containing 7, Histone Lysine Methyltransferase | Protein Coding | Q8WTS6 | 57 | GC04M139495 | 1.507174 | https://www.genecards.org/cgi-bin/carddisp.pl?gene=SETD7 |
| SHMT1 | Serine Hydroxymethyltransferase 1 | Protein Coding | P34896 | 57 | GC17M092729 | 1.507174 | https://www.genecards.org/cgi-bin/carddisp.pl?gene=SHMT1 |
| SMS | Spermine Synthase | Protein Coding | P52788 | 57 | GC0XP021958 | 1.507174 | https://www.genecards.org/cgi-bin/carddisp.pl?gene=SMS |
| BHMT | Betaine--Homocysteine S-Methyltransferase | Protein Coding | Q93088 | 56 | GC05P079111 | 1.507174 | https://www.genecards.org/cgi-bin/carddisp.pl?gene=BHMT |
| FBL | Fibrillarin | Protein Coding | P22087 | 56 | GC19M039834 | 1.507174 | https://www.genecards.org/cgi-bin/carddisp.pl?gene=FBL |
| KMT2C | Lysine Methyltransferase 2C | Protein Coding | Q8NEZ4 | 56 | GC07M152134 | 1.507174 | https://www.genecards.org/cgi-bin/carddisp.pl?gene=KMT2C |
| LIAS | Lipoic Acid Synthetase | Protein Coding | O43766 | 56 | GC04P039682 | 1.507174 | https://www.genecards.org/cgi-bin/carddisp.pl?gene=LIAS |
| NSUN2 | NOP2/Sun RNA Methyltransferase 2 | Protein Coding | Q08J23 | 56 | GC05M006599 | 1.507174 | https://www.genecards.org/cgi-bin/carddisp.pl?gene=NSUN2 |
| RNMT | RNA Guanine-7 Methyltransferase | Protein Coding | O43148 | 56 | GC18P024189 | 1.507174 | https://www.genecards.org/cgi-bin/carddisp.pl?gene=RNMT |
| SETDB1 | SET Domain Bifurcated Histone Lysine Methyltransferase 1 | Protein Coding | Q15047 | 56 | GC01P150926 | 1.507174 | https://www.genecards.org/cgi-bin/carddisp.pl?gene=SETDB1 |
| SMYD2 | SET And MYND Domain Containing 2 | Protein Coding | Q9NRG4 | 56 | GC01P214281 | 1.507174 | https://www.genecards.org/cgi-bin/carddisp.pl?gene=SMYD2 |
| PRMT7 | Protein Arginine Methyltransferase 7 | Protein Coding | Q9NVM4 | 55 | GC16P114043 | 1.507174 | https://www.genecards.org/cgi-bin/carddisp.pl?gene=PRMT7 |
| SUV39H1 | SUV39H1 Histone Lysine Methyltransferase | Protein Coding | O43463 | 55 | GC0XP060118 | 1.507174 | https://www.genecards.org/cgi-bin/carddisp.pl?gene=SUV39H1 |
| AHCYL1 | Adenosylhomocysteinase Like 1 | Protein Coding | O43865 | 54 | GC01P109984 | 1.507174 | https://www.genecards.org/cgi-bin/carddisp.pl?gene=AHCYL1 |
| AOC1 | Amine Oxidase Copper Containing 1 | Protein Coding | P19801 | 54 | GC07P150824 | 1.507174 | https://www.genecards.org/cgi-bin/carddisp.pl?gene=AOC1 |
| DNMT3L | DNA Methyltransferase 3 Like | Protein Coding | Q9UJW3 | 54 | GC21M044246 | 1.507174 | https://www.genecards.org/cgi-bin/carddisp.pl?gene=DNMT3L |
| FTSJ1 | FtsJ RNA 2'-O-Methyltransferase 1 | Protein Coding | Q9UET6 | 54 | GC0XP048476 | 1.507174 | https://www.genecards.org/cgi-bin/carddisp.pl?gene=FTSJ1 |
| KMT5B | Lysine Methyltransferase 5B | Protein Coding | Q4FZB7 | 54 | GC11M136690 | 1.507174 | https://www.genecards.org/cgi-bin/carddisp.pl?gene=KMT5B |
| METTL3 | Methyltransferase 3, N6-Adenosine-Methyltransferase Complex Catalytic Subunit | Protein Coding | Q86U44 | 54 | GC14M021498 | 1.507174 | https://www.genecards.org/cgi-bin/carddisp.pl?gene=METTL3 |
| SETD1A | SET Domain Containing 1A, Histone Lysine Methyltransferase | Protein Coding | O15047 | 54 | GC16P113188 | 1.507174 | https://www.genecards.org/cgi-bin/carddisp.pl?gene=SETD1A |
| SUV39H2 | SUV39H2 Histone Lysine Methyltransferase | Protein Coding | Q9H5I1 | 54 | GC10P014878 | 1.507174 | https://www.genecards.org/cgi-bin/carddisp.pl?gene=SUV39H2 |
| ASH1L | ASH1 Like Histone Lysine Methyltransferase | Protein Coding | Q9NR48 | 53 | GC01M155335 | 1.507174 | https://www.genecards.org/cgi-bin/carddisp.pl?gene=ASH1L |
| ELP3 | Elongator Acetyltransferase Complex Subunit 3 | Protein Coding | Q9H9T3 | 53 | GC08P028089 | 1.507174 | https://www.genecards.org/cgi-bin/carddisp.pl?gene=ELP3 |
| EMG1 | EMG1 N1-Specific Pseudouridine Methyltransferase | Protein Coding | Q92979 | 53 | GC12P006970 | 1.507174 | https://www.genecards.org/cgi-bin/carddisp.pl?gene=EMG1 |
| KMT2E | Lysine Methyltransferase 2E (Inactive) | Protein Coding | Q8IZD2 | 53 | GC07P109894 | 1.507174 | https://www.genecards.org/cgi-bin/carddisp.pl?gene=KMT2E |
| KMT5A | Lysine Methyltransferase 5A | Protein Coding | Q9NQR1 | 53 | GC12P137522 | 1.507174 | https://www.genecards.org/cgi-bin/carddisp.pl?gene=KMT5A |
| MBD2 | Methyl-CpG Binding Domain Protein 2 | Protein Coding | Q9UBB5 | 53 | GC18M054151 | 1.507174 | https://www.genecards.org/cgi-bin/carddisp.pl?gene=MBD2 |
| NSD3 | Nuclear Receptor Binding SET Domain Protein 3 | Protein Coding | Q9BZ95 | 53 | GC08M038269 | 1.507174 | https://www.genecards.org/cgi-bin/carddisp.pl?gene=NSD3 |
| TFB1M | Transcription Factor B1, Mitochondrial | Protein Coding | Q8WVM0 | 53 | GC06M155247 | 1.507174 | https://www.genecards.org/cgi-bin/carddisp.pl?gene=TFB1M |
| TRDMT1 | TRNA Aspartic Acid Methyltransferase 1 | Protein Coding | O14717 | 53 | GC10M017138 | 1.507174 | https://www.genecards.org/cgi-bin/carddisp.pl?gene=TRDMT1 |
| TRMT1 | TRNA Methyltransferase 1 | Protein Coding | Q9NXH9 | 53 | GC19M013104 | 1.507174 | https://www.genecards.org/cgi-bin/carddisp.pl?gene=TRMT1 |
| AS3MT | Arsenite Methyltransferase | Protein Coding | Q9HBK9 | 52 | GC10P102869 | 1.507174 | https://www.genecards.org/cgi-bin/carddisp.pl?gene=AS3MT |
| ASMT | Acetylserotonin O-Methyltransferase | Protein Coding | P46597 | 52 | GC0XP002811 | 1.507174 | https://www.genecards.org/cgi-bin/carddisp.pl?gene=ASMT |
| CDKAL1 | CDK5 Regulatory Subunit Associated Protein 1 Like 1 | Protein Coding | Q5VV42 | 52 | GC06P020534 | 1.507174 | https://www.genecards.org/cgi-bin/carddisp.pl?gene=CDKAL1 |
| ICMT | Isoprenylcysteine Carboxyl Methyltransferase | Protein Coding | O60725 | 52 | GC01M020774 | 1.507174 | https://www.genecards.org/cgi-bin/carddisp.pl?gene=ICMT |
| KMT2B | Lysine Methyltransferase 2B | Protein Coding | Q9UMN6 | 52 | GC19P142831 | 1.507174 | https://www.genecards.org/cgi-bin/carddisp.pl?gene=KMT2B |
| METTL1 | Methyltransferase 1, TRNA Methylguanosine | Protein Coding | Q9UBP6 | 52 | GC12M057768 | 1.507174 | https://www.genecards.org/cgi-bin/carddisp.pl?gene=METTL1 |
| MRM2 | Mitochondrial RRNA Methyltransferase 2 | Protein Coding | Q9UI43 | 52 | GC07M002234 | 1.507174 | https://www.genecards.org/cgi-bin/carddisp.pl?gene=MRM2 |
| PEMT | Phosphatidylethanolamine N-Methyltransferase | Protein Coding | Q9UBM1 | 52 | GC17M092673 | 1.507174 | https://www.genecards.org/cgi-bin/carddisp.pl?gene=PEMT |
| PRDM2 | PR/SET Domain 2 | Protein Coding | Q13029 | 52 | GC01P013700 | 1.507174 | https://www.genecards.org/cgi-bin/carddisp.pl?gene=PRDM2 |
| PRMT2 | Protein Arginine Methyltransferase 2 | Protein Coding | P55345 | 52 | GC21P046635 | 1.507174 | https://www.genecards.org/cgi-bin/carddisp.pl?gene=PRMT2 |
| PRMT6 | Protein Arginine Methyltransferase 6 | Protein Coding | Q96LA8 | 52 | GC01P107056 | 1.507174 | https://www.genecards.org/cgi-bin/carddisp.pl?gene=PRMT6 |
| RSAD2 | Radical S-Adenosyl Methionine Domain Containing 2 | Protein Coding | Q8WXG1 | 52 | GC02P006865 | 1.507174 | https://www.genecards.org/cgi-bin/carddisp.pl?gene=RSAD2 |
| SETD1B | SET Domain Containing 1B, Histone Lysine Methyltransferase | Protein Coding | Q9UPS6 | 52 | GC12P137500 | 1.507174 | https://www.genecards.org/cgi-bin/carddisp.pl?gene=SETD1B |
| SETMAR | SET Domain And Mariner Transposase Fusion Gene | Protein Coding | Q53H47 | 52 | GC03P004303 | 1.507174 | https://www.genecards.org/cgi-bin/carddisp.pl?gene=SETMAR |
| SRM | Spermidine Synthase | Protein Coding | P19623 | 52 | GC01M011054 | 1.507174 | https://www.genecards.org/cgi-bin/carddisp.pl?gene=SRM |
| TRMT5 | TRNA Methyltransferase 5 | Protein Coding | Q32P41 | 52 | GC14M060971 | 1.507174 | https://www.genecards.org/cgi-bin/carddisp.pl?gene=TRMT5 |
| NOP2 | NOP2 Nucleolar Protein | Protein Coding | P46087 | 51 | GC12M006556 | 1.507174 | https://www.genecards.org/cgi-bin/carddisp.pl?gene=NOP2 |
| PCMT1 | Protein-L-Isoaspartate (D-Aspartate) O-Methyltransferase | Protein Coding | P22061 | 51 | GC06P149749 | 1.507174 | https://www.genecards.org/cgi-bin/carddisp.pl?gene=PCMT1 |
| MAT2B | Methionine Adenosyltransferase 2 Non-Catalytic Beta Subunit | Protein Coding | Q9NZL9 | 50 | GC05P163504 | 1.507174 | https://www.genecards.org/cgi-bin/carddisp.pl?gene=MAT2B |
| NSUN3 | NOP2/Sun RNA Methyltransferase 3 | Protein Coding | Q9H649 | 50 | GC03P094062 | 1.507174 | https://www.genecards.org/cgi-bin/carddisp.pl?gene=NSUN3 |
| SMYD3 | SET And MYND Domain Containing 3 | Protein Coding | Q9H7B4 | 50 | GC01M245749 | 1.507174 | https://www.genecards.org/cgi-bin/carddisp.pl?gene=SMYD3 |
| ALKBH8 | AlkB Homolog 8, TRNA Methyltransferase | Protein Coding | Q96BT7 | 49 | GC11M107502 | 1.507174 | https://www.genecards.org/cgi-bin/carddisp.pl?gene=ALKBH8 |
| CAMKMT | Calmodulin-Lysine N-Methyltransferase | Protein Coding | Q7Z624 | 49 | GC02P044361 | 1.507174 | https://www.genecards.org/cgi-bin/carddisp.pl?gene=CAMKMT |
| DIMT1 | DIM1 RRNA Methyltransferase And Ribosome Maturation Factor | Protein Coding | Q9UNQ2 | 49 | GC05M062387 | 1.507174 | https://www.genecards.org/cgi-bin/carddisp.pl?gene=DIMT1 |
| DPH5 | Diphthamide Biosynthesis 5 | Protein Coding | Q9H2P9 | 49 | GC01M100989 | 1.507174 | https://www.genecards.org/cgi-bin/carddisp.pl?gene=DPH5 |
| NSUN4 | NOP2/Sun RNA Methyltransferase 4 | Protein Coding | Q96CB9 | 49 | GC01P070159 | 1.507174 | https://www.genecards.org/cgi-bin/carddisp.pl?gene=NSUN4 |
[truncated: 46,593 more chars]
